# Chromosome-level reference genome for the medically important Arabian horned viper (*Cerastes gasperettii*)

**DOI:** 10.1093/gigascience/giaf030

**Published:** 2025-06-06

**Authors:** Gabriel Mochales-Riaño, Samuel R Hirst, Adrián Talavera, Bernat Burriel-Carranza, Viviana Pagone, Maria Estarellas, Theo Busschau, Stéphane Boissinot, Michael P Hogan, Jordi Tena-Garcés, Davinia Pla, Juan J Calvete, Johannes Els, Mark J Margres, Salvador Carranza

**Affiliations:** Institute of Evolutionary Biology (IBE), CSIC-Universitat Pompeu Fabra, 08003 Barcelona, Spain; Department of Integrative Biology, University of South Florida, Tampa, FL 33620, USA; Institute of Evolutionary Biology (IBE), CSIC-Universitat Pompeu Fabra, 08003 Barcelona, Spain; Institute of Evolutionary Biology (IBE), CSIC-Universitat Pompeu Fabra, 08003 Barcelona, Spain; Museu de Ciències Naturals de Barcelona, P° Picasso s/n, Parc Ciutadella, 08003 Barcelona, Spain; Institute of Evolutionary Biology (IBE), CSIC-Universitat Pompeu Fabra, 08003 Barcelona, Spain; Institute of Evolutionary Biology (IBE), CSIC-Universitat Pompeu Fabra, 08003 Barcelona, Spain; New York University Abu Dhabi, Abu Dhabi, United Arab Emirates; New York University Abu Dhabi, Abu Dhabi, United Arab Emirates; Department of Biological Sciences, Florida State University, Tallahassee, FL 33306, USA; Department of Ecology and Evolutionary Biology, University of Michigan, Ann Arbor, MI 48109-1085, USA; Evolutionary and Translational Venomics Laboratory, Instituto de Biomedicina de Valencia, Consejo Superior de Investigaciones Científicas (CSIC) 46010 Valencia, Spain; Evolutionary and Translational Venomics Laboratory, Instituto de Biomedicina de Valencia, Consejo Superior de Investigaciones Científicas (CSIC) 46010 Valencia, Spain; Evolutionary and Translational Venomics Laboratory, Instituto de Biomedicina de Valencia, Consejo Superior de Investigaciones Científicas (CSIC) 46010 Valencia, Spain; Breeding Centre for Endangered Arabian Wildlife, Environment and Protected Areas Authority, Sharjah, United Arab Emirates; Department of Integrative Biology, University of South Florida, Tampa, FL 33620, USA; Institute of Evolutionary Biology (IBE), CSIC-Universitat Pompeu Fabra, 08003 Barcelona, Spain

**Keywords:** toxin evolution, gene synteny, genomics, transcriptomics, venom

## Abstract

Venoms have traditionally been studied from a proteomic and/or transcriptomic perspective, often overlooking the true genetic complexity underlying venom production. The recent surge in genome-based venom research (sometimes called “venomics”) has proven to be instrumental in deepening our understanding of venom evolution at the molecular level, particularly through the identification and mapping of toxin-coding loci across the broader chromosomal architecture. Although venomous snakes are a model system in venom research, the number of high-quality reference genomes in the group remains limited. In this study, we present a chromosome-resolution reference genome for the Arabian horned viper *Cerastes gasperettii* (NCBI: txid110202), a venomous snake native to the Arabian Peninsula. Our highly contiguous genome (genome size: 1.63 Gbp; contig N50: 45.6 Mbp; BUSCO: 92.8%) allowed us to explore macrochromosomal rearrangements within the Viperidae family, as well as across squamates. We identified the main highly expressed toxin genes within the venom glands comprising the venom's core, in line with our proteomic results. We also compared microsyntenic changes in the main toxin gene clusters with those of other venomous snake species, highlighting the pivotal role of gene duplication and loss in the emergence and diversification of snake venom metalloproteinases and snake venom serine proteases for *C. gasperettii*. Using Illumina short-read sequencing data, we reconstructed the demographic history and genome-wide heterozigosity of the species, revealing how historical aridity likely drove population expansions. Finally, this study highlights the importance of using long-read sequencing as well as chromosome-level reference genomes to disentangle the origin and diversification of toxin gene families in venomous snake species.

## Background

The rise of genomics in non-model organisms has led to an increase in the number of high-quality reference genomes available in recent years [1–6]. Advances in sequencing technologies have catalyzed the study of several complex traits from a genomic perspective, such as coloration, domestication, or venom, among others [3, 7–10]. Among these, venom genomic research has been particularly important in enhancing our understanding of the origin, evolution, and dynamics of this medically relevant trait [11–14]. Venom is a potentially lethal cocktail rich in proteins and peptides (from now on referred to as “toxins”) which are actively secreted by specialized venom glands [11, 15]. Toxins can have different effects depending on their type, interactions with other molecules, and the organism in which they are introduced, with convergent outcomes in different taxa [15, 16]. Historically, venom research has primarily been conducted using proteomic and transcriptomic approaches (see [7] and references therein). The identification of venom toxins and the characterization of their evolution using reference genomes is a recent and novel field [17]. Previous works have shown that changes in gene regulation can result in the activation and deactivation of venom-coding genes at all taxonomic levels and within the same individual [2, 16, 18, 19]. This suggests that transcriptomic and proteomic data are critical for studying venoms in conjunction with well annotated reference genomes to disentangle the complete number and biochemical nature of the toxins an individual can potentially transcribe [7]. Ultimately, the study of venom genomics may yield evolutionary insights into antivenom or drug discovery, as it enables the identification of unexpressed toxin-coding genes. These genes, often overlooked by transcriptomic or proteomic approaches unless ontogeny analyses or in-depth venom expression studies are performed, may target unique physiological pathways. Such discoveries could lead to novel therapies for human illnesses including but not limited to cancer [11, 20–22]. Unexpressed toxin-coding genes are particularly noteworthy because they may represent evolutionary “reservoirs” of bioactive molecules. These genes could encode toxins with unique mechanisms of action, offering untapped potential for drug discovery or therapeutic innovation.

Venom has evolved independently in multiple groups including cnidarians, molluscs, arthropods, squamates, and even mammals [11, 15]. Venomous snakes are one of the most life-threatening animal groups to humans [23] and, therefore, a medically relevant model system in venom research. Venomous snakes are a diverse group with more than 600 species [24], in which venom has evolved with the objective of immobilizing and digesting their prey [25]. More than 370 species of venomous snake have been classified as of medically important by the World Health Organization (WHO) due to their potential severe effects on humans [26]. Snakebite is considered a neglected tropical disease, with annual mortality exceeding 100,000 victims worldwide [23, 27]. The most medically important venomous snake families are Elapidae, Viperidae, and Atractaspididae [28], although within Colubridae (*sensu lato*) there are certain medically important venomous species as well [29]. Envenomation by certain members of these families can result in a range of pathologies, spanning neurotoxic, hemotoxic, and/or cytotoxic effects depending on the number and composition of toxins. Neurotoxic venoms primarily target the central nervous system and are mainly composed of small proteins including three-finger toxins (3FTs), snake venom phospholipases A_2_ group I (SV-G^I^-PLA_2_), or dendrotoxins, and are usually associated with elapid snakes [30]. Conversely, hemotoxic and cytotoxic venoms generally are comprised of large enzymatic proteins and protein complexes, including snake venom metalloproteases (SVMPs), serine proteases (SPs) or snake venom phospholipases A_2_ group II (SV-G^II^-PLA_2_), and are typically associated with viperid snakes [28, 31, 32]. Although these historical classifications have proven to be somewhat useful for treating envenomations medically, recent studies have revealed that the presence of these toxins are not exclusive to specific snake families [33].

Vipers (family Viperidae) are a monophyletic lineage of venomous snakes found across Eurasia, Africa, and America [34], and have received extensive research attention primarily due to their medical relevance [35–39]. The majority of venom studies in this group have primarily been investigated using a proteomic approach, with early venom work being highly motivated by the medical field, with a limited number of studies employing genomic approaches (but see [2, 3, 5, 40–42]. Sequencing efforts to obtain high-quality reference genomes have mainly focused on pitvipers (Crotalinae subfamily, 11 reference genomes, NCBI last accessed 13 March 2024), especially within the *Crotalus* (*n* = 6) genus, and have focused on the study of venom evolution [2, 3, 5, 43, 44]. Other viperids have also been sequenced (although in lower numbers) from both Azemiopinae and Viperinae subfamilies (one and four, respectively) [41, 45, 46]. Currently, reference genomes are only available for 16 viper species out of the total 387 total species via the NCBI genomic database [24]. Vipers display extensive variation in venom composition between and within genera [47, 48] and even intraspecifically [49, 50]. Such differences are most likely due to the high diversity of venom genes and their different effects on prey, but are also, at least in some cases, the result of introgression with related species [19, 49, 50]. This provides an extraordinary opportunity to study trait evolution both at inter- and intraspecific levels.

Native to the Arabian Peninsula, the Arabian horned viper (*Cerastes gasperettii*, family Viperidae) is a venomous snake currently recognized within the highest medical importance category (WHO; accessed July, 2024). Extending from the Sinai Peninsula to southwestern Iran in the north and reaching as far as Yemen and Oman in the south, its distribution is widespread (Supplementary Fig. S1). Found mainly in sandy habitats, this arid-adapted ground-dwelling snake with generalist requirements [51–53] is one of the most common venomous snakes found in Arabia and is responsible for occasional snakebite envenomations [54–56].

In this study, we present a high-quality chromosome-level reference genome assembly for *C. gasperettii* (NCBI: txid110202), being one of the first within the Viperinae subfamily. Our highly contiguous genome showcases a high level of similarity at the chromosome level within the Viperidae family with some minor rearrangements with elapids. Moreover, combining genomics, transcriptomics, and proteomics, we characterized the main toxins found in its venom and the location of those toxins in the genome, comparing their evolutionary history and gene copy number variation with those of other venomous species. We deciphered its adequate levels of genetic diversity. Finally, we reconstructed the demographic history for the species, revealing how historical increases in aridity likely drove population expansions. Overall, the genomic resources generated in this study provide an essential reference resource for forthcoming studies on venom evolution.

## Methods

### Sampling

Three adult specimens (two females and one male) of *C. gasperettii gasperettii* were used for this study (Supplementary Table S1). Blood was extracted only from a single female individual (the heterogametic sex, sample CG1) to obtain high-molecular-weight (HMW) genomic DNA (gDNA). We anesthesized the individual, extracted blood from the heart, and stored this in ethanol and EDTA. For each of the 3 individuals, we extracted 12 different tissues, including the venom gland, which was stored in RNAlater until RNA extraction (Supplementary Table S1 and Supplementary Fig. S2). Before dissections, venom was extracted and snakes were allowed to recover for 4 days to maximize the venom gland transcription. We only extracted the left venom gland per individual because previous research within the same family has shown that both venom glands provide indistinguishable results [57].

### DNA extraction, library preparation and sequencing

We extracted gDNA from the blood of a female individual (CG1 in Supplementary Table S1) using the MagAttract HMW Kit (Qiagen, Germany) following the manufacturer’s protocols without modifications. Then, we sequenced a total of two 8 M SMRT HiFi cells in a Sequel II PacBio machine, aiming for a ∼30× of coverage, at the University of Leiden. Hi-C libraries were prepared using the Omni-C kit (Dovetail Genomics), following the manufacturer's protocol and using blood stored in EDTA, at the National Center for Genomic Analyses (CNAG), in Barcelona, Spain. The library was paired-end sequenced on a NovaSeq 6000 (2 × 150 bp) following the manufacturer’s protocol for dual indexing and aiming for a coverage of ∼60×. Finally, we sequenced short-read whole-genome data of the same individual using a NEB Ultra II FS DNA kit; the library was paired-end sequenced on a NovaSeq 6000 (2 × 150 bp) at the Core sequencing platform from the New York University of Abu Dhabi, aiming for ∼70× depth of coverage.

### RNA extraction, library preparationm and sequencing

We extracted RNA from the same 3 individuals described above (Supplementary Table S1 and Supplementary Fig. S2). RNA was isolated using a HighPurity Total RNA Extraction Kit (Canvax, Valladolid, Spain). We selected a total of 35 samples (Supplementary Table S2). RNA libraries were prepared with the VAHTS Universal V8 RNA-seq Library Prep Kit, and being strand-specific, were sequenced on a NovaSeq 6000 (2 × 150 bp) aiming for an average of 40 M read pairs per sample (Supplementary Table S2), but we first sequenced the reference individual and subsequently the other 2 samples. Moreover, we sequenced one 8 M SMRT HiFi cell on a Sequel II PacBio machine containing 2 Iso-seq HiFi libraries at the University of Leiden: one containing only the venom gland, and the second library being a pool of 8 high-quality tissues (brain, kidney, liver, gallbladder, spleen, tongue, pancreas, and ovary).

### Genome assembly and scaffolding

Quality control of HiFi and Illumina reads was performed using FastQC (RRID:SCR_014583) v.0.12.1 [58] and adapters were removed with cutadapt (RRID:SCR_011814) v.4.9 [59]. In order to initially explore the genome size, heterozygosity levels, and coverage data, we generated a *k*-mer profile with Meryl (RRID:SCR_026366) v.1.4.1 [60], using the raw HiFi reads and default parameters, and visualized it with GenomeScope2 (RRID:SCR_017014) v.2.0.1 [61]. Then, we assembled the genome following the VGP assembly pipeline v.2.0 [62]. PacBio HiFi reads were assembled into contigs using the software Hifiasm (RRID:SCR_021069) v.0.21.0 [63], producing primary and alternate assemblies. We used purge_dups (RRID:SCR_021173) [64] to remove haplotypic duplicates from the primary assembly and added them to the alternate assembly. Then, we scaffolded the resulting haplotypic assembly using the Hi-C data with SALSA2 (Salsa, RRID:SCR_022013) v.1 [65], with default parameters. Following the VGP assembly pipeline [62], manual curation was performed with Pretext (RRID:SCR_022024) v.0.2.5. Breaks were not manually created and we joint contings on gaps previously identified by SALSA2 (Salsa, RRID:SCR_022013). We used the ∼78× Illumina data to polish the assembly with one round of Pilon (RRID:SCR_014731) v.1.24 [66]. The mitochondrial genome was obtained with GetOrganelle (RRID:SCR_022963) v.1.7.7.1 [67], using the available mitochondrial genome of several *Echis* species (*E. coloratus, E. carinatus*, and *E. omanensis*) to seed the assembly (NCBI: SRX18902082, SRX18902083, SRX18902084, respectively).

### Genome assembly quality evaluation

Quality assessment and general metrics for the final assembly were estimated with both QUAST (Quast, RRID:SCR_011228) v.5.1.0 [68] and gfastats (RRID:SCR_026368) v.1.3.8 [69]. Possible contaminations were evaluated with BlobToolKit (Blobtools, RRID:SCR_017618) v.4.4.0 [70] using the NCBI taxdump database. We also used MitoFinder v.1.4.2 [71, 72] to confirm that the mitochondrial genome was absent in the assembled nuclear reference genome. Completeness of the genome assembly was assessed with BUSCO (Busco, RRID:SCR_015008) v.5.3.0. against the sauropsida_odb10 database (*n* = 7,480).

### Genome annotation

First, we identified repetitive elements using RepeatModeler (RRID:SCR_015027) v.2.0.3 [73] for *de novo* predictions of repeat families. To annotate genome-wide complex repeats, we used RepeatMasker (RRID:SCR_012954) v.4.1.3 [74] with default settings to identify known Tetrapoda repeats present in the curated Repbase database [75]. Then, we ran 3 iterative rounds of RepeatMasker to annotate the known and unknown elements identified by RepeatModeler in order to maximize the known elements at the expense of diminishing the unknown elements. Later, we soft-masked the genome for simple repeats. We used GeMoMa (GeMoMa, RRID:SCR_017646) v.1.9 [76] to annotate protein-coding genes, combining both the RNA-seq data generated in this study as described above (already mapped in to our new assembly) as well as annotations from 6 other squamate genomes already published: *Crotalus adamanteus* [2], *Crotalus tigris* [3], *Ophiophagus hannah* [17], *Naja naja* [6], *Crotalus ruber* [42] and *Crotalus viridis* [5]. We quality checked and removed the adapters of the RNA-seq data using fastp v.0.23.3 [77], as well as mapped the transcriptomic data to our new reference genome with Hisat2 (RRID:SCR_015530) v.2.2.1 [78]. Additionally, we also removed the adapters for the Iso-seq data with fastp (RRID:016962) v.0.23.3 [77] and mapped the long-read transcriptomic data to our new reference genome with pbmm2 (RRID:SCR_025549), collapsing mapped reads into unique isoforms with isoseq3 and annotating with GeneMarkS-T (GeneMark, RRID:SCR_011930) v.5.1 [79]. We combined both annotations (GeMoMa and GeneMarkS-T) with TSEBRA [80]. We BLASTp (blastp, RRID:SCR_001010) our predicted proteins to a Uniprot protein database for a total of 10 species (*C. gasperettii, C. vipera, C. cerastes, Anolis carolinensis, C. viridis, C. tigris, C. ruber, C. adamanteus, O. hannah* and *N. naja*). Simultaneously, we ran Interproscan v.5.72 [81] on our predicted proteins. Then, we combined both functional annotations with AGAT v.1.4.1 [82]. Finally, as toxin-coding gene families are known to occur in large tandem arrays and the number of paralogs can be underestimated in particular gene families [5], we performed additional annotation steps for toxin genes: Following [3], we used a combination of empirical annotation in FGENESH+ (FGENESH, RRID:SCR_011928) [83], as well as manual annotation using RNA-seq and Iso-seq alignments; the former identified all genes regardless of expression, whereas the latter was used to explicitly identify expressed toxins.

### Chromosome-level analyses

Chromosomal synteny was explored between our new chromosome-level reference genome for the Arabian horned viper together with the Eastern diamondback rattlesnake (*C. adamanteus*) [2], the Indian cobra (*N. naja*) [6], and the brown anole (*Anolis sagrei*) [84] using MCscan (RRID:SCR_017650) v.1.4.23 [85]. Protein sequences from each of the 3 venomous snakes were extracted using AGAT v.1.2.1 [82] and were pairwise aligned with LAST [86], implemented in the JCVI python module [87]. A first alignment was used between the 3 species to identify chromosomes assembled in the reverse complement, which were corrected using SAMtools faidx (samtools, RRID:SCR_002105) v.1.18.1 [88] using both options reverse-complement and mark-strand. Gene annotations for the new reference (with the corresponding reversed chromosomes) were annotated using GeMoMa v.1.9 [76], and MCscan was rerun. The last 4 scaffolds (14, 15, 16, and 17) from *A. sagrei* were removed because no orthologous groups were found.

### Transcriptomics

After adapter trimming and quality control using fastp v.0.23.3 [77], we mapped our RNA-seq reads to the reference genome of *C. gasperettii* using Hisat2 (RRID:SCR_015530) v.2.2.1 [78]. Gene expression raw counts per gene across all samples were calculated with StringTie (RRID:SCR_016323) [89]. Initial exploration of our transcriptomic data revealed a clear batch effect for one of the 3 samples (Supplementary Fig. S4), due to the low mapping of that sample to our reference genome. Therefore, we decided to remove individual CG1 from future RNA-seq analyses. Moreover, to avoid pseudoreplication, we also removed the accessory gland from individual CG009 due to its high similarity with the venom gland, suggesting that the venom gland rather than the accessory gland was sampled (Supplementary  Fig. S4). Differential expression analyses were carried out with the DESeq2 package (RRID:SCR_015687) v.1.42.0 [90] from R v.4.4.2 [91]. Prior to analysis, genes with <10 counts across all samples were filtered out. For comparisons, we defined 2 groups: venom glands versus all other tissues. DESeq2 uses a negative binomial generalized linear model to estimate differences in gene expression, and the *P*-values were adjusted for multiple testing using the Benjamini–Hochberg method to control the false discovery rate. Genes with an adjusted *P*-value <0.01 and a fold change (FC) >2 were considered significantly differentially expressed. Finally, we identified the highly expressed genes found in the venom gland as well as the toxins uniquely expressed in the venom gland (following [6]) which were defined as: (1) genes expressed in the venom gland (transcripts per million (TPM) > 500), (2) differential upregulated genes with a FC > 2 comparing venom glands with all other tissues, and (3) unique to venom glands (TPM < 500 in all other tissues).

### Proteomics

A bottom-up mass spectrometry (MS) strategy [92] was used to characterize the venom of *C. gasperettii*. Briefly, the venom proteome (pooled from individuals CN6134 and CN6135, both from the United Arab Emirates (UAE); Supplementary Table S1) was submitted to reverse-phase high-performance liquid chromatography decomplexation followed by SDS-PAGE analysis in 12% polyacrylamide gels run under non-reducing and reducing conditions. Protein bands were excised from Coomassie Brilliant Blue-stained gels and subjected to automated in-gel reduction and alkylation on a Genomics Solution ProGest Protein Digestion Workstation. Tryptic digests were submitted to MS/MS analysis on a nano-Acquity UltraPerformance LC (UPLC) equipped with a BEH130 C_18_ (100 µm × 100 mm, 1.7 µm particle size) column in-line with a Waters SYNAPT G2 high-definition mass spectrometer. Doubly and triply charged ions were selected for CID-MS/MS. Fragmentation spectra were matched against a customized database including the bony vertebrates taxonomy dataset of the NCBI nonredundant database (release 258, 15 October 2023) plus the species-specific venom gland transcriptomic and genomic protein sequences gathered in this work. Search parameters were as follows: enzyme: trypsin (two-missed cleavage allowed); MS/MS mass tolerance for monoisotopic ions: ±0.6 Da; carbamidomethyl cysteine and oxidation of methionine were selected as fixed and variable modifications, respectively. Assignments with significance protein score threshold of *P* < 0.05 (Mascot score > 43) were taken into consideration, and all associated peptide ion hits were manually validated. Unmatched MS/MS spectra were *de novo* sequenced and manually matched to homologous snake toxins available in the NCBI nonredundant protein sequences database using the default parameters of the BLASTP program.

### Local synteny analyses

To explore toxin genomic organization across (sub)families, we used BLASTn, incorporating both toxin and nontoxin paralogs to identify the genomic location of SVMPs, SVSPs, and PLA_2_ toxin families, across the genome of *C. gasperettii, C. adamanteus, N. naja* and *A. feae*. We excluded *A. feae* for SVSP and SVMP local synteny analyses because those families were not assembled onto a single contig in the *A. feae* genome. Then, we aligned those regions using Mafft [93]: for SVMPs in CHR8:16.506.135 to CHR8:17.374.029, for SVSPs in CHR9: 17.531.416 to CHR9:17.788.049 and for PLA_2_ in CHR17:7.882.542 to CHR17:7.916.827 Each species was annotated within the MSA using its own annotation as a reference in Geneious Prime 2023.0.4. Results were plotted using the gggenomes package [94] from R v.4.4.2 [91].

### Toxin phylogenies

We used phylogenetic inference to study the evolutionary history for the main groups of toxins (i.e., SVMPs and SVSPs), which were the most abundant in the proteome of *C. gasperettii*, as well as PLA_2_ because this family has been widely studied within the Viperidae family [12, 41]. For the 3 main toxin families, we selected available toxin genes as well as nontoxin paralogous genes from venomous species; we also included other nontoxin paralogous genes from nontoxic species (for details, see Supplementary Datasets for the 3 main toxins). When nuclear sequences were obtained, we translated CDS to protein sequence, and then protein sequences were aligned with Mafft (RRID:SCR_011811) v.7 [93]. Following [13], we built a phylogeny for each of the toxin groups with the translated CDS sequences, as explained above, using Phyml (RRID:SCR_014629) v.3.3 [95], implementing the Dayhoff substitution model and validating our inferred tree with aBayes support.

### Demographic history

We inferred the demographic history of *C. gasperettii* by implementing the Pairwise Sequential Markovian Coalescent (PSMC) software (RRID:SCR_017229) v.0.6.5 [96] on the short-read whole-genome data. Heterozygous positions were obtained from bam files with the Samtools v.1.9 mpileup function [97], and data were filtered for low mapping (<30) and base quality (<30). Minimum and maximum depths were set at a third (27×) and twice (156×) the average coverage. Only autosomal chromosomes were considered. We used a squamate mutation rate of 2.4 × 10^−9^ substitutions/site/generation and a generation time of 3 years, following [98, 99], respectively. A total of 10 bootstraps were calculated, plotting the final results with the psmc_plot.pl function from PSMC.

### Genomic diversity

We downloaded Illumina data for *Bothrops jararaca* (SRR13839751 from [40], *Crotalus viridis* (SRR19221440; [5]), *Naja kaouthia* (SRR8224383; [100]), *N. naja* (SRR10428156; [6]) and *Sistrurus tergeminus* (SRR12802282; [101]). Then, we filtered for quality (Phred score: 30) and removed adapters with fastp v.0.23.3 [77]. Trimming of poly-G/X tails and correction in overlapped regions were specified. All other parameters were set as default. Filtered sequences were visually explored with FastQC (fastQC, RRID:SCR_014583) v.0.12.1 [58] to ensure data quality and absence of adapters. *C. gasperettii* filtered reads were mapped against the new reference genome of *C. gasperettii* using the bwa mem algorithm (bwa, RRID:SCR_010910) v.0.7.17 [102]. *B. jararaca, C. viridis*, and *S. tergeminus* were mapped against the *C. viridis* [5] reference genome and *N. naja* and *N. kaouthia* were mapped against the *N. naja* reference genome [6]. Mapped reads were sorted with Samtools (RRID:SCR_002105) v,1.9 [97] and duplicated reads were marked and removed with PicardTools (Picard, RRID:SCR_006525) v,2.28.0 [103]. Reads with mapping quality <30 were discarded. SNP calling was carried out with HaplotypeCaller (GATK, RRID:SCR_001876) v.4.1.3.0 [104], with BP_resolution and split by chromosome. For each chromosome, individual genotypes were joined using CombineGVCFs with convert-to-base-pair-resolution, and the GenotypeGVCFs tool was then applied to include nonvariant sites. Finally, for each individual, the whole dataset split by chromosome was concatenated with bcftools concat (bcftools, RRID:SCR_ 005227) [88], keeping only the autosomes. Then, for each sample, we used the raw dataset to calculate average genome heterozygosity. We generated nonoverlapping sliding windows for each of the reference genomes and included only sites (both variant and invariant) with site quality >30 (QUAL field in a VCF file from GATK). Only windows containing more than 60,000 unfiltered sites were considered. Visualization was carried out with ggplot2 (RRID:SCR_ 014601) [105] in R v.4.4.2 [91].

## Results and Discussion

### Genome assembly and annotation

**Figure 1:**
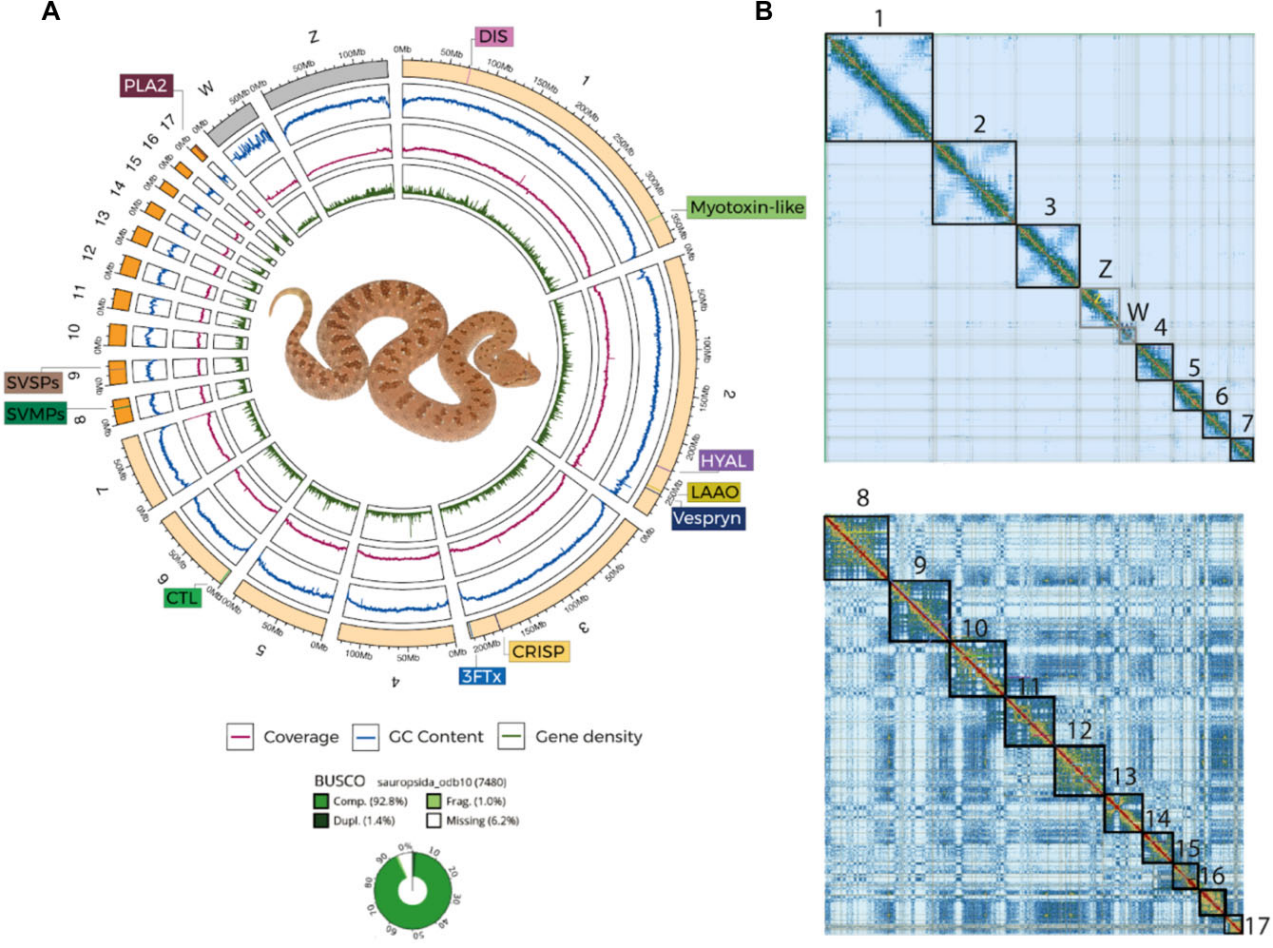
(A) Reference genome for *C. gasperettii*, including BUSCO score, GC content, coverage level, and the main toxins found within the genome. Macrochromosomes are shown in light orange; microchromosomes are shown in bright orange. Sex chromosomes are shown in gray. DIS: disintegrins; HYAL: hyaluronidases; LAAO: L-amino acid oxidase; CRISP: cysteine-rich secreted proteins; CTL: C-type lectins. (B) HiC contact map for the macrochromosomes (above), including the sex chromosomes (Z and W), and microchromosomes (below).

We generated a high-quality chromosome-level assembly for *C. gasperettii* by combining PacBio HiFi (65 Gbp of data), Hi-C (96 Gbp of data), and Illumina data (135 Gbp of data) (Fig. 1 and Supplementary Fig. S3). First, we *de novo* assembled the HiFi reads into 1,018 contigs (N50 = 45.7 Mbp; longest contig: 149.99 Mbp). Then, using the proximity ligation data (i.e., Hi-C), we scaffolded the genome into 319 scaffolds (N50 = 111.38 Mbp; largest scaffold: 345.38 Mbp). After manual curation, the scaffolding parameters of our genome were improved (N50 = 214.14 Mbp; largest scaffold: 361.99 Mbp), containing 99.44% of the genome present in 19 scaffolds or pseudochromosomes (7 macro-, 10 micro-, Z and W sex chromosomes; Table 1 and Fig. 1B). The total genome length was 1.63 Gb, similar to other venomous snakes [3, 5, 6, 17] (Table 1), with a contig N50 of 45.6 Mbp, ∼3.3 times more contiguous than the *N. naja* genome [6], ∼228 times more contiguous than the *A. sagrei* genome [84], but 0.67 times less contiguous than the recently published *C. adamanteus* genome [2], making it one of the most contiguous chromosomal squamate genomes assembled to date (Table 1). We assessed the completeness of the assembly using BUSCO [106] with the sauropsida gene set (*n* = 7,480). Upon evaluation, we successfully identified 92.8% of the genes (91.4% single-copy, 1.4% duplicated), while the remaining genes were fragmented (1%) or missing (6.2%; Fig. 1). For the *de novo* assembly, GC content and repeat content were 37.87% and 43.63%, respectively. The repetitive landscape was dominated by retroelements (30.25%), with a majority of LINEs (21.25%) (Supplementary Table S3). Finally, we annotated 27,158 different protein-coding genes within our assembly, with a total of 194 putative toxins or toxin-paralog genes. Toxin genes were found in both macro- and microchromosomes (Fig. 1), and were found on individual contigs. Finally, we also found a battery of 3FTxs and myotoxin-like genes, but they were not represented in our proteome and RNA-seq dataset (see below).

**Table 1:** Comparison of our new reference genome for *C. gasperettii* with other high-quality squamate genomes. Best value per category is shown in bold

	*C. gasperettii*	*C. adamanteus*	*N. naja*	*A. sagrei*
Genome size	1.63 Gbp	1.69 Gbp	1.79 Gbp	1.92 Gbp
Number of scaffolds	221	**27**	1,897	3,738
Scaffold N50	214.14 Mbp	208.9 Mbp	223.35 Mbp	**253.58** Mbp
Scaffold L50	**3**	**3**	**3**	4
Contig N50	45.6 Mbp	**67.5** Mbp	13.06 Mbp	0.2 Mbp

### Genomic architecture highly conserved among vipers

**Figure 2:**
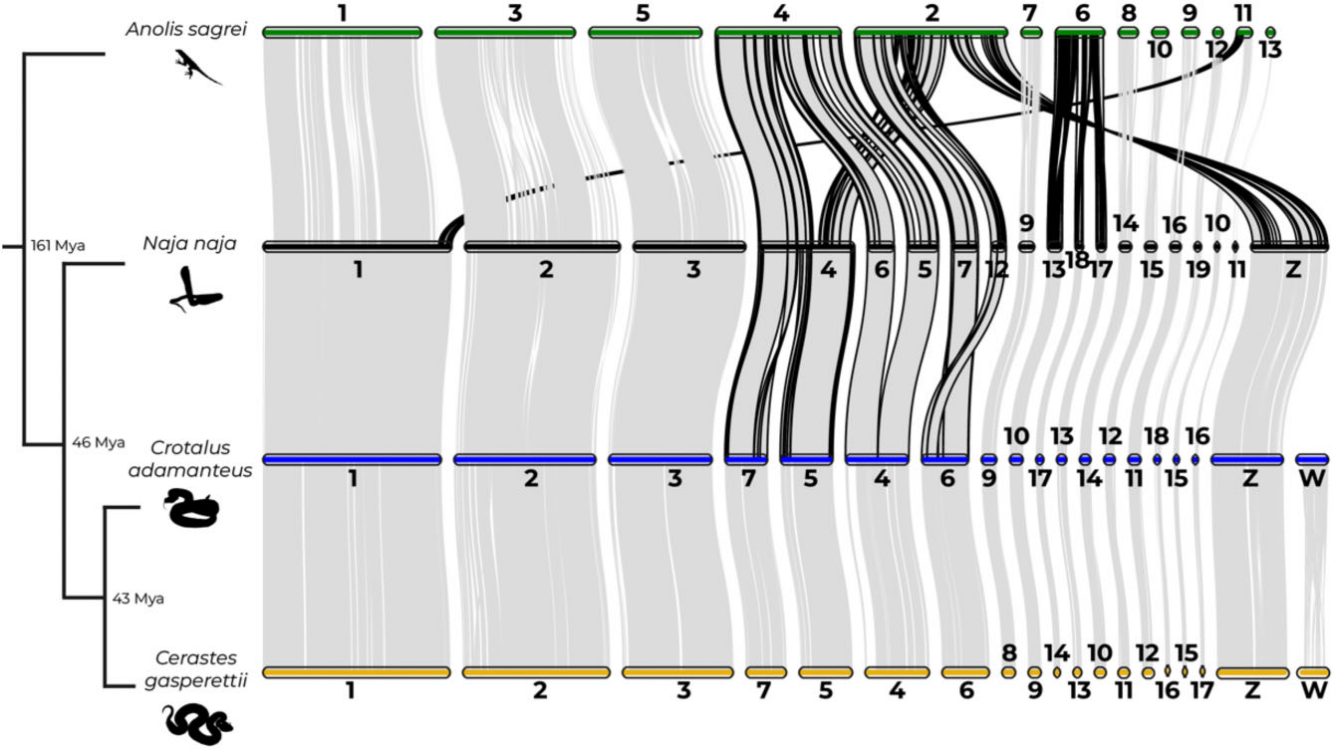
Chromosome-level analyses for one Elapidae (*N. naja*), one Crotalinae (*C. adamanteus*), and one Viperinae (*C. gasperettii*) species, with *A. sagrei* as the outgroup. The 4 smallest scaffolds (14, 15, 16, and 17) of *A. sagrei* were removed because no orthologous groups were found with other species. Borders of regions showing evidence for chromosomal rearrangements are shown in black. Estimates for branch times obtained from TimeTree.org based on divergence times between Iguania and Serpentes, Elapidae and Viperidae, and Crotalinae and Viperinae, respectively.

Whole-genome synteny comparisons showed similarity between *C. gasperettii* and *C. adamanteus*, with large syntenic blocks both within macro- and microchromosomes (Fig. 2). Some chromosomal rearrangements were observed between viperids and elapids, as previously discussed by [6], with a fission of chromosome 4 in *N. naja* to form chromosomes 5 and 7 in vipers, and a fusion of chromosomes 5 and 6 in *N. naja* to form chromosome 4 in vipers. Interestingly, several chromosomal rearrangements between lizards and snakes have occurred: we found several fission events in the *A. sagrei* genome, including one fission from chromosome 2 that originates the current Z chromosome in snakes (Fig. 2).

### Toxins uniquely expressed in the venom glands

**Figure 3:**
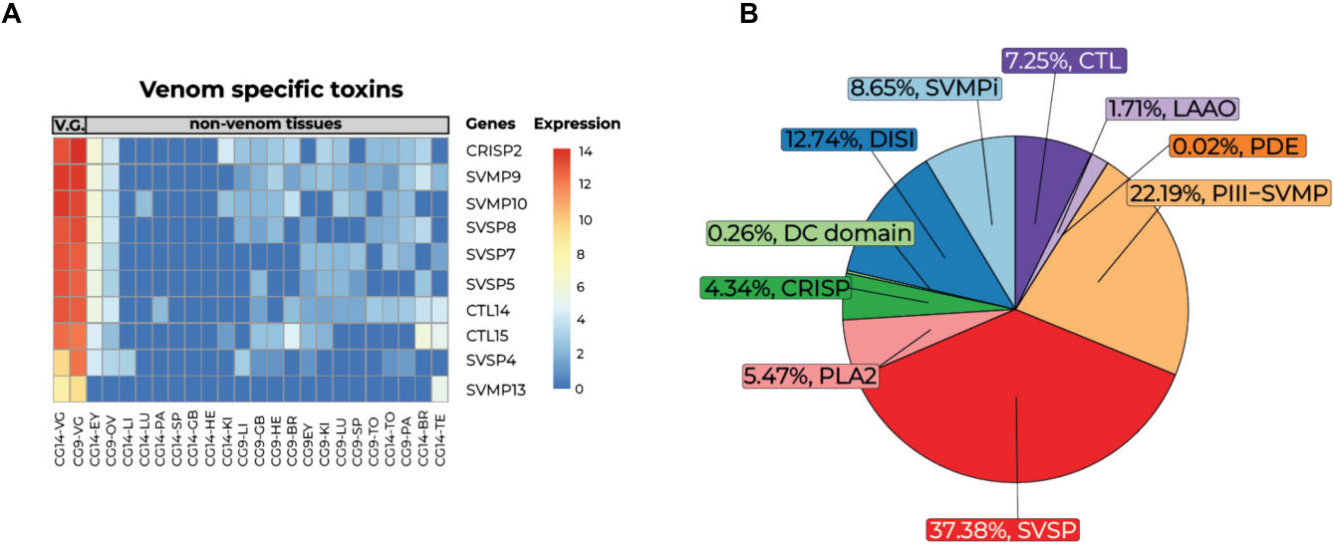
Main toxins found in both the transcriptome and proteome of *C. gasperettii*. (A) Transcriptomic results with genes upregulated and exclusively found in the venom gland for both individuals. Each column represents a different tissue type per sample. Rows show the different genes, and colors correspond to different expression levels. VG: venom gland; EY: eye; OV: ovary; LI: liver; LU: lung; PA: pancreas; SP: spleen; GB: gallbladder; HE: heart; KI: kidney; LI: liver; BR: brain; TO: tongue; TE: testis. (B) Proteomic results of venom composition for a pool of 2 individuals of *C. gasperettii*. The pie chart displays the relative abundances of the toxin families found in the proteome of the *C. gasperettii* venom. PDE, phosphodiesterases.

Our analyses of multitissue transcriptomic data (23 samples from 2 individuals covering 13 different tissues) reported a total of 23,178 expressed genes (TPM > 1). A heatmap of the 2,000 most variable genes reported unique upregulated genes for each of the analyzed tissues (Supplementary Fig. S5). The venom gland transcriptome contained a total of 7,237 genes expressed (TPM > 500), including a total of 65 putative toxin genes. From those, we did not detect any 3FTxs and/or myotoxin-like gene transcripts. Differential gene expression analyses revealed a total of 161 genes (33 putative toxin genes) that were differentially upregulated (FC > 2 and 1% false discovery rate) in venom glands compared to other tissues (Fig. 3A and Supplementary Figs S6 and S7). Finally, a total of 10 toxin genes (*CRISP2, SVMP9, SVMP10, SVSP8, SVSP7, SVSP5, CTL14, CTL15, SVSP4*, and *SVMP13*) were uniquely expressed in the venom gland, encoding for the minimal core venom effector (Fig. 3A) [6], and in line with the main toxins found within the proteome (Fig. 3B), although some differences were observed (such as the absence of PLA_2_ within the highly expressed genes), possibly due to individual venom differences. These 10 genes, together with other SVMPs, SVSPs, and C-type lectins (CTLs), were highly expressed in the venom gland and form the core toxic effector components of the venom. Targeting the core toxins together with other well-known modulators of venom may help manufacture of synthetic antivenom treatments and improve neutralization tests of current antivenoms [6]. However, more transcriptomic data should be incorporated to correct for potential ontogenetic and geographical variation in venom composition in *C. gasperettii* [18, 107].

### SVSPs and SVMPs as main toxins

Venom proteomics identified SVSPs and SVMPs as the most abundant toxin families within the venom of *C. gasperettii*, with 37.38% and 22.19% of the venom being composed by peptides from those 2 families, respectively (Fig. 3B); the dominance of these 2 toxin families is consistent with previous research on the same genus [108, 109]. Other toxin families identified were DISI (12.74%), CTL (7.25%), PLA_2_ (5.47%), cysteine-rich secretory proteins (CRISP; 4.34%) or L-amino acid oxidase (LAAO; 1.71%) (Fig. 3B). We did not detect any 3FTx or myotoxin-like peptides within the proteome.

### SVMPs

**Figure 4:**
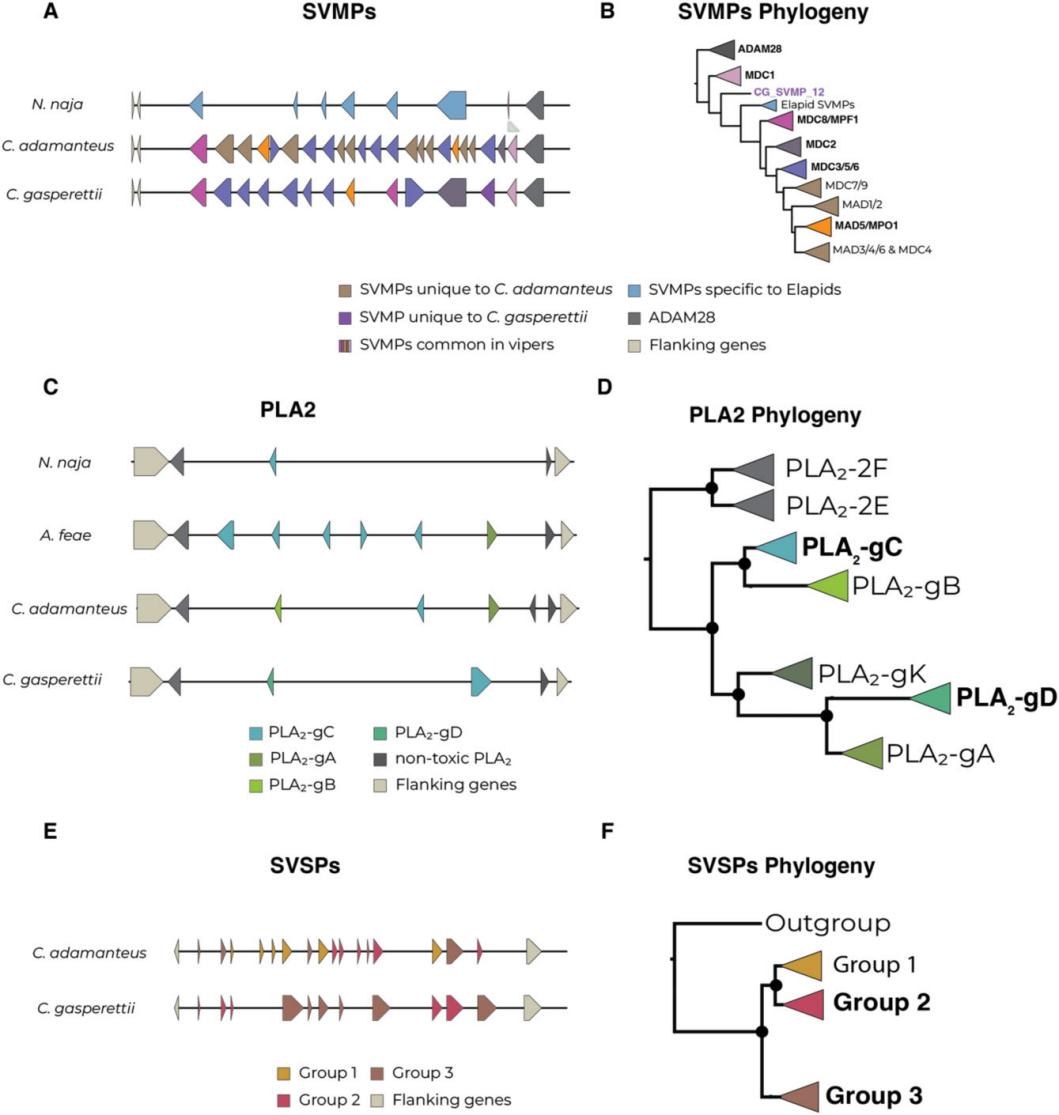
(A) Local synteny analyses for the SVMP toxin family in *N. naja, C. adamanteus*, and *C. gasperettii*. Different colors indicate orthologous genes unique to *C. gasperettii*, crotalids, true vipers, or elapids. *ADAM28* (right) as well as flanking genes (left) are also indicated. (B) Phylogeny of SVMPs. Bold type indicates groups that contained SVMPs from *C. gasperettii*; purple indicates the gene is exclusively found in *C. gasperettii*. (C) Local synteny analyses for PLA_2_ in *N. naja, A. feae, C. adamanteus*, and *C, gasperettii*. Nontoxic PLA_2_ and flanking genes are also shown. (D) Phylogeny of the PLA_2_ gene family, with 2 non-toxic PLA_2_s as outgroups. Some samples that did not fit in any category have been removed. For a complete phylogeny see Supplementary Fig. S9. Note that PLA_2_-gK is present in the phylogeny but not in the local synteny analyses, as any of the studied species contains it. (E) Local synteny analyses for SVSPs for *C. adamanteus* and *C. gasperettii*. Flanking genes are also shown. (F) Phylogeny for SVSPs with a nontoxic outgroup. For the 3 different phylogenies the groups that contained toxins from *C. gasperettii* are highlighted in bold.

We analyzed the evolution of venom of the most abundant venom toxin groups (i.e., SVMPs and SVSPs, as well as PLA_2_). After a thorough manual curation, we used comparative genomics to evaluate the number and position of those genes in comparison with the Indian cobra (*N. naja*), the Eastern diamondback rattlesnake (*C. adamanteus*), and Fea’s viper (*Azemiops feae*). We reported a total of 13 fully contiguous tandem array SVMPs for *C. gasperettii* (Fig. 4A), next to the nontoxic paralogous gene *ADAM28* and flanked by the *NEFL* and *NEFM* nontoxic genes. Microsyntenic analyses showed gene copy number variation between the studied species (Fig. 4A). Overall, we can see an expansion in the number of SVMPs within the Viperidae family, particurlarly in *C. adamanteus* (22 copies unique to vipers and 10 lineage-specific copies) but also in *C. gasperettii* (12 copies unique to vipers and 1 lineage-specific copy) (Fig. 4A). Then, we reconstructed the evolutionary history of this toxin family (Figs 4B and Supplementary Fig. S8). Phylogenetic analyses for this toxin group reported a highly supported clade comprising *ADAM28* peptides, the nontoxic paralogous gene. The second clade of orthologous toxin-peptides were found within both elapid and viperid families (including species from Crotalinae and Viperinae subfamilies in viperids; Supplementary Fig. S8) as well as 2 SVMPs from *A. feae*. Interestingly, we report a new toxin-coding gene within *C. gasperettii* with a different evolutionary history, as it did not share orthology with any other gene (Fig. 4B). This new gene likely arose from a duplication event of *SVMP13*, within the group of SVMP *MDC1* toxins (Supplementary Fig. S8). Our discovery of a novel SVMP gene in *C. gasperettii* adds to the growing body of work on the dynamic evolution of venom systems. Similar gene expansions and duplications have been observed in other species, such as PLA_2_ toxin-coding genes found in the venom of *A. feae* [41], highlighting the lineage-specific nature of venom evolution. The gene we identified, possibly arising from an *SVMP13* duplication, does not share orthology with genes in other species, suggesting the presence of hidden toxin diversity in venom systems. This discovery highlights the importance of using genomics in studying venom evolution, as this putatively toxic gene was not found to be differentially upregulated in the venom gland or recovered in the proteome (Fig. 3). More genomic data will indicate if *SVMP12* is unique for the Viperinae subfamily, the *Cerastes* genus, or if it is only found in *C. gasperettii*. All other clades were unique to viperids (and some exclusive only to crotalids), except for a clade composed by SVMPs unique to elapids, as previously discussed in [6]. Interestingly, one of the toxins (*SVMP8*) was not a class P-III SVMP, as it clusters within the MAD-4/5 clade (class P-II SVMP), contrary to the proteomic results where all SVMPs were categorized within the class P-III (Fig. 3B). Although there has been a clear expansion of the SVMP family within the *Crotalus* genus, our results suggest that the origin of that expansion was at the beginning of the Viperidae family, as most of the groups are also present within the Viperinae subfamily.

### PLA_2_

Regarding PLA_2_, we report 2 tandem repeat venom genes for *C. gasperettii* within the non-toxic *PLA_2_-g2E* and *PLA_2_-g2F* array (Fig. 4C), flanked by *OTUD3* and *MUL1* nontoxic genes, as previously reported in other species [3, 12, 41]. The number of venomous PLA_2_s in *C. gasperettii* was lower than in *A. feae* and *C. adamanteus*. Phylogenetic results for PLA_2_ genes showed a fully supported clade containing both nontoxic *PLA_2_-g2E* and *PLA_2_-g2F* as outgroups (Fig. 4D and Supplementary Fig. S9). We also found all other PLA_2_ groups reported in previous studies: *PLA_2_-gC, PLA_2_-gK, PLA_2_-gB, PLA_2_-gD*, and *PLA_2_-gA* [12, 41]. The 2 genes for our target species clustered in different groups (Fig. 4D and Supplementary Fig. S9). The first PLA_2_ was a *PLA_2_-gD*, which is a group of PLA_2_s exclusively found in true vipers (subfamily Viperinae). The second one was a *PLA_2_-gC* which is more ancestral as it is also found in other pitvipers and nonvenomous snakes such as pythons [12]. The genomic results are consistent with the proteomics, indicating that specific duplications of PLA_2_ toxin-coding genes have not occurred in *C. gasperettii*.

### SVSPs

We found 8 different SVSPs within the genome of *C. gasperettii*, flanked by *RBM42* and *GRAMD1A* nontoxic genes (Fig. 4E). For this toxin family, we were only able to compare the results with *C. adamanteus*. We were unable to confidently determine the location of SVSPs in the *N. naja* genome (several regions matched our venomous SVSP genes as well as the flanking genes). Moreover, *A. feae* was also not compared because SVSPs were not assembled in a single contig. Phylogenetic results showed 3 clades, with 2 containing *C. gasperettii* genes (Fig. 4F and Supplementary Fig. S10). Group 1 was mainly present within *Crotalus*, although there was the presence of some true vipers species, but not in *C. gasperettii* (Fig. S10). Group 2 contained 6 genes within *C. adamanteus* and only 2 for *C. gasperettii*. Interestingly, Group 3 was expanded in *C. gasperettii* (Fig. 4E) with a total of 6 copies, while 4 were found within *C. adamanteus*. Most of the toxins included in the analyses for true vipers were also found in Group 3 (Supplementary Fig. S10), indicating a possible expansion of this group of toxins in true vipers (or gene losses in pit vipers). Overall, our high-quality chromosome-level reference genome has shed light on the evolution of the main toxin-coding gene families, indicating a compelling correlation between the abundance of toxin-coding genes and the prevalence of these toxins in the venom of *C. gasperettii*.

### Glacial periods drove population expansions of *C. gasperettii*

**Figure 5:**
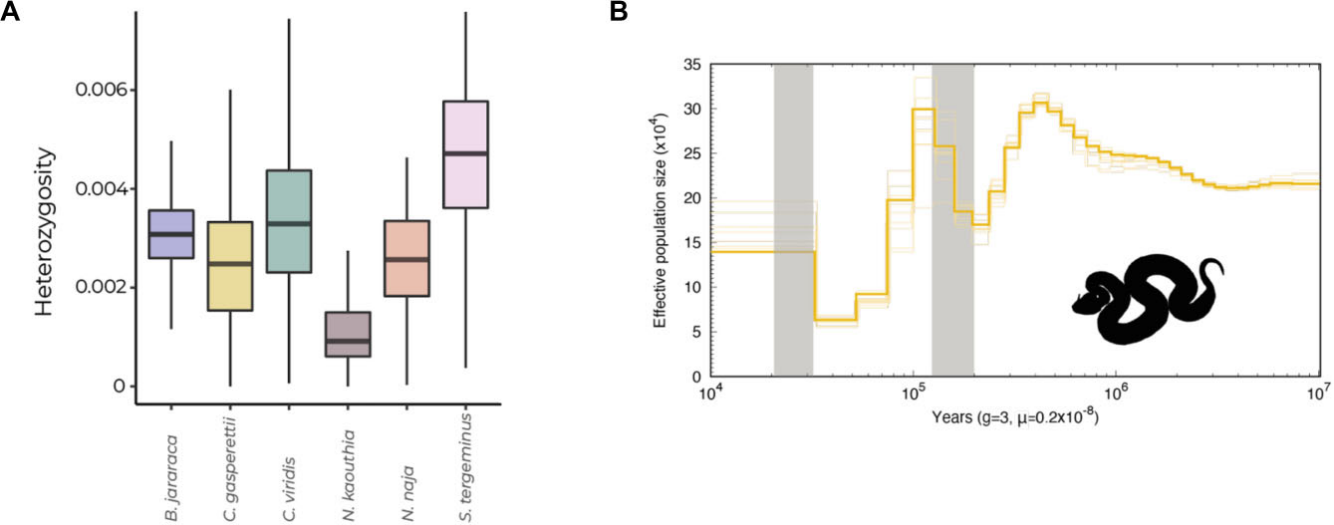
(A) Genome-wide diversity for 6 different venomous snakes: *B. jararaca, C. gasperettii, C. viridis, N. kaouthia, N. naja*, and *S. tergeminus*. (B) PSMC analysis recovering the ancient demographic history of *C. gasperettii*. Generation time was set to 3 years and the substitution rate to 2.4 × 10^−9^ per site per year. Shaded lines represent 10 bootstrap estimates. Two last glacial periods are shown with gray lines.

The Arabian horned viper (*C. gasperettii*) is a widespread species, categorized as “Least Concern” by the IUCN [109]. Genome-wide diversity was in line with its conservation status, as it showed similar heterozygosity levels compared to other venomous snakes (Fig. 5A). However, more individuals should be sampled along its distribution to verify that similar heterozygosity levels are found across its range. PSMC analyses showed several population expansions and contractions in the last 400 kya, whilst the effective population size of *C. gasperettii* remained relatively constant from 1 until 10 Mya (Fig. 5B). Interestingly, population expansions were coincident with the last glacial and penultimate glacial periods (gray lines on Fig. 5B), with a large population increase during the penultimate glacial period (1.94–1.35 mya) (Fig. 5B). In fact, during glacial periods, the global sea level dropped around 150 m, exposing the floor and the sand to the wind, which promoted aridification in the Arabian Peninsula and potentially increased habitat suitability for the species [110, 111]. PSMC results may vary depending on the generation time as well as the mutational rate specified. The absence of species-specific data for this analyses may bias our results, although it is a general consensus in the literature when inferring demographic analyses such as ours in snakes (e.g., [5]).

## Conclusions

Our high-quality chromosome-level reference genome for *C. gasperettii* showed that chromosomal architecture is highly conserved between Crotalinae and Viperinae subfamilies, and differs from elapid genomes by a small number of chromosomal rearrangements. We also found the genomic coordinates of the main toxin-encoding genes, highlighting gene duplication as the main driver in the evolution of SVMP and SVSP toxins. We identified a new SVMP toxin-coding gene, showcasing the importance of using high-quality reference genomes (combined with other -omic techniques) for thoroughly characterizing toxin-encoding genes. Finally, this is a new and important resource for a large clade with few reference genomes available. Future genomic studies focusing on Old World viper evolution will benefit greatly from this resource, which will help unveil the origin and diversification of venom and serve as an essential genomic tool for further venomic studies on the subfamily Viperinae.

## Supplementary Material

giaf030_Supplemental_File

giaf030_Authors_Response_To_Reviewer_Comments_original_submission

giaf030_Authors_Response_To_Reviewer_Comments_Revision_1

giaf030_GIGA-D-24-00269_original_submission

giaf030_GIGA-D-24-00269_Revision_1

giaf030_GIGA-D-24-00269_Revision_2

giaf030_Reviewer_1_Report_original_submission Jiatang Li -- 8/20/2024

giaf030_Reviewer_1_Report_Revision_1 Jiatang Li -- 12/18/2024

giaf030_Reviewer_2_Report_original_submission Blair Perry -- 9/9/2024

giaf030_Reviewer_2_Report_Revision_1 Blair Perry -- 12/27/2024

giaf030_Reviewer_3_Report_original_submission Hardip Patel -- 9/11/2024

giaf030_Reviewer_3_Report_Revision_1 Hardip Patel -- 12/21/2024

## Data Availability

Final assembly and raw reads files have been deposited in NCBI under bioproject no. PRJNA1068073. Proteomic data have been published at PRIDE [112, 113] under project accession numbers PXD060777 and PXD060783. All additional supporting data are available in the *GigaScience* repository, GigaDB [114].

## Additional Files

**Supplementary Fig. S1**. Distribution map for the studied species *C. gasperettii* with the location of our samples. Countries where the species is present are indicated. JO: Jordan; SA: Saudi Arabia; YE: Yemen; OM: Oman; UAE: United Arab Emirates; IQ: Iraq; IR: Iran; KU: Kuwait; QA: Qatar.

**Supplementary Fig. S2**. Drawing of an Arabian horned viper depicting all the tissues sampled for RNA-seq analyses.

**Supplementary Fig. S3**. Histogram from GenomeScope showing the frequency of reads in relation with their coverage.

**Supplementary Fig. S4**. Heatmap for the 2,000 most variable genes within our 3 samples, showing a clear batch effect of sample CG1 (possibly due to differences in sequencing time) as well as a high similarity between the putative accessory gland and the venom gland. Each column represents a different sampled tissue. The 3 different samples are depicted with different colors at the top of the heatmap. Abbreviations are as follows: G. bladder: gallbladder; V. gland: venom gland.

**Supplementary Fig. S5**. Heatmap for the 2,000 most variable genes for both samples, reporting highly expressed genes unique for each tissue type. Each column represents one tissue sampled per individual. Expression levels were normalized. Te: testis; Ov: ovary; G.b.: gallbladder; Sp: spleen; V.G.: venom gland.

**Supplementary Fig. S6**. Heatmap for the 161 upregulated genes found in the venom gland of *C. gasperettii* transcriptome including the 65 putative expressed toxins for both venom gland samples. Each column represents one tissue sampled per individual. VG: venom Ggland; Ki: kidney; GB: gall bladder; Lu: lung; Sp: spleen; He: heart; Li: liver; Pa: pancreas; To: tongue; Te: Testis; Ov: ovary.

**Supplementary Fig. S7**. Heatmap for the venom gland transcriptome for the 65 putative expressed toxins for both venom gland samples. Each column represents one tissue sampled per individual. VG: venom Ggland; Ki: kidney; GB: gall bladder; Lu: lung; Sp: spleen; He: heart; Li: liver; Pa: pancreas; To: tongue; Te: Testis; Ov: ovary.

**Supplementary Fig. S8**. Maximum-likelihood phylogeny for SVMP genes and its non-toxic paralog (ADAM28). Genes for *C. gasperettii* are highlighted in bold. Toxin groups are identified following previous categorizations. Asterisks indicate if *C. gasperettii* genes are present in that specific group. Branch support with aBayes values >90 are depicted as circles.

**Supplementary Fig. S9**. Maximum-likelihood phylogeny for PLA_2_, with the 2 nontoxic genes as outgroups (PLA_2_-2F and PLA_2_-2E). Asterisks in group labels indicate if *C. gasperettii* genes are present in that specific group. Branch support with aBayes values >90 are depicted as circles.

**Supplementary Fig. S10**. Maximum-likelihood phylogeny for SVSPs, with 1 sample from *Thamnophis elegans* as outgroup. Asterisks in group labels indicate if *C. gasperettii* genes are present in that specific group. Branch support with aBayes values >90 are depicted as circles.

**Supplementary Table S1**. Individuals sampled in this study with their sex, sampling coordinates, and data sequenced.

**Supplementary Table S2**. Id, tissue type and number of reads sequenced per sample.

**Supplementary Table S3**. Different types of repetitive elements masked within the genome.

**Supplementary Table S4**. Abundances for the different toxin families identified in the proteome of *C. gasperettii*.
