# Supplementary material for: Chromosome-level reference genome for the medically important Arabian horned viper (Cerastes gasperettii)
Source: Gigascience. 2025 Jun 6;14:giaf030. doi: 10.1093/gigascience/giaf030 (PMC12143202; doi:10.1093/gigascience/giaf030)
Supplement: giaf030_Supplemental_File [file giaf030_supplemental_file.pdf]

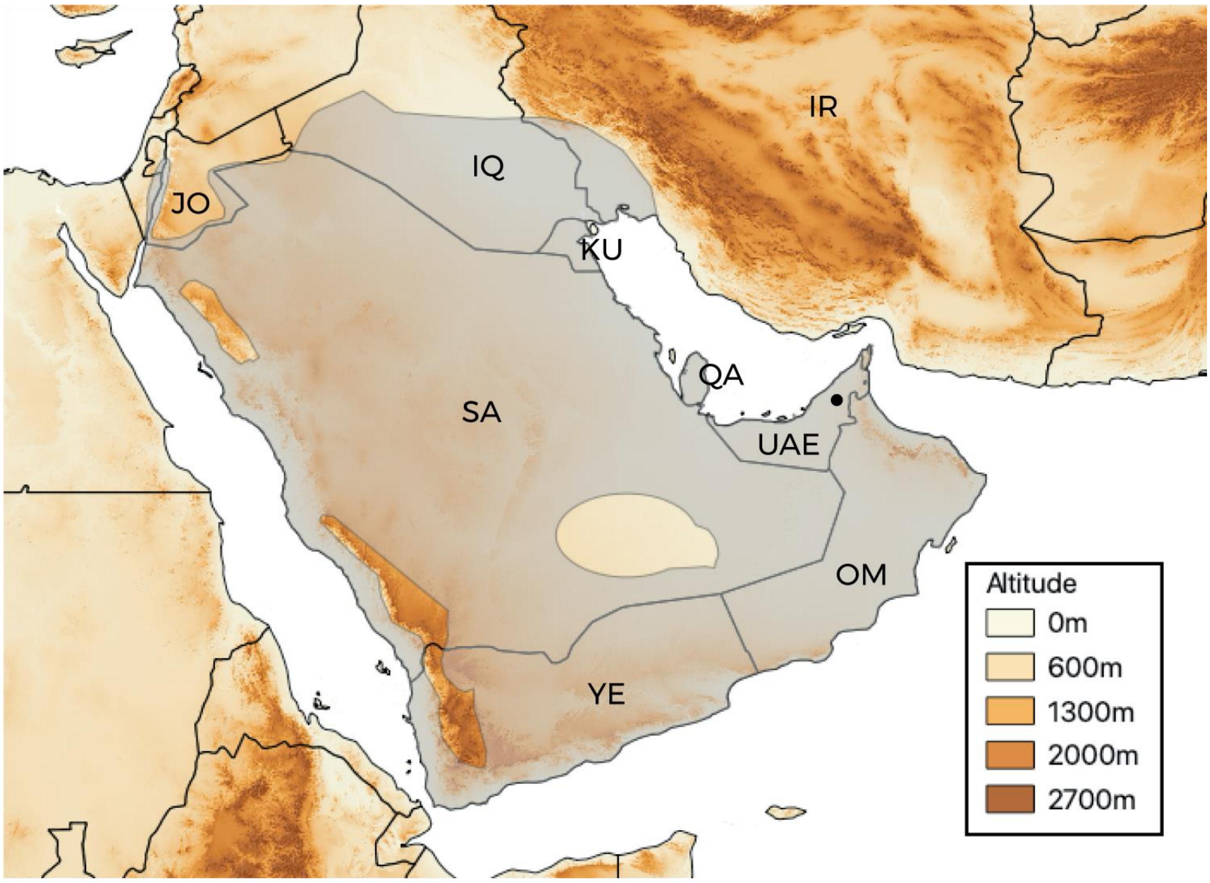

Fig. S1: Distribution map for the studied species *Cerastes gasperettii* with the location of our samples. Countries where the species is present are indicated. Abbreviations are as follows: JO, Jordania; SA, Saudi Arabia; YE, Yemen; OM, Oman; UAE, United Arab Emirates; IQ, Iraq; IR, Iran; KU, Kuwait, QA, Qatar.

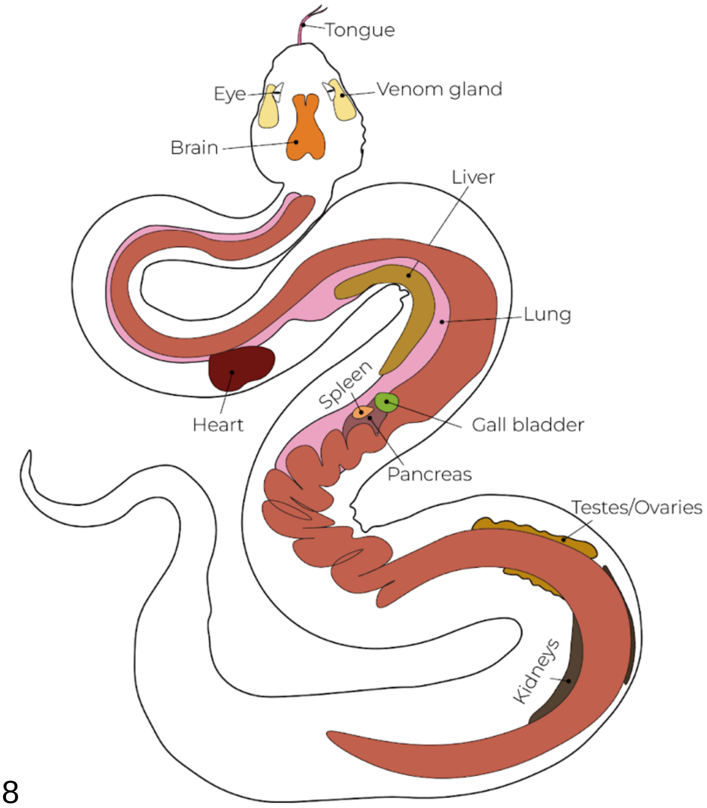

Fig. S2: Drawing of an Arabian horned viper depicting all the tissues sampled for RNA-seq analyses.

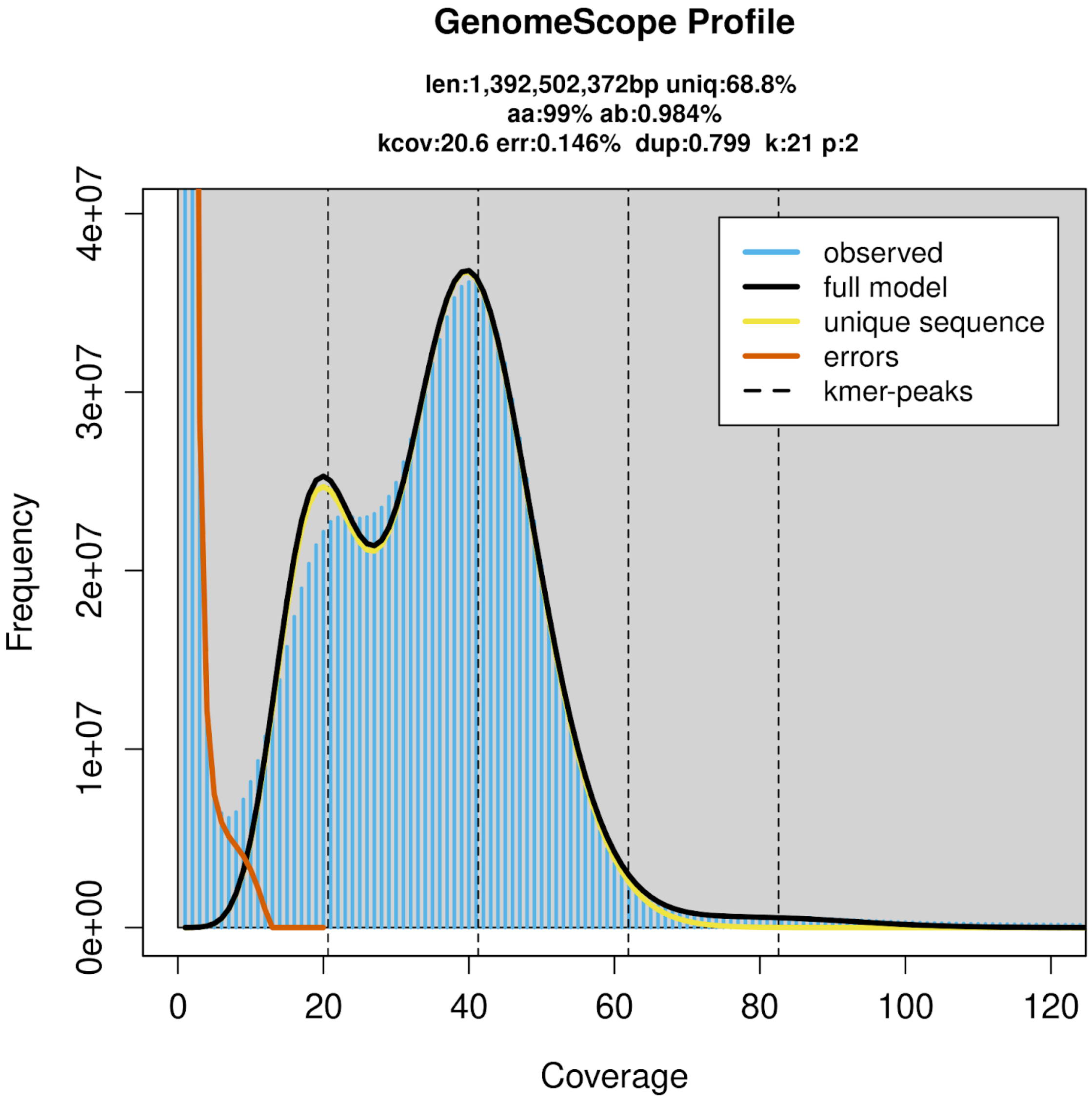

Fig. S3: Histogram from GenomeScope showing the frequency of reads in relation with their coverage.

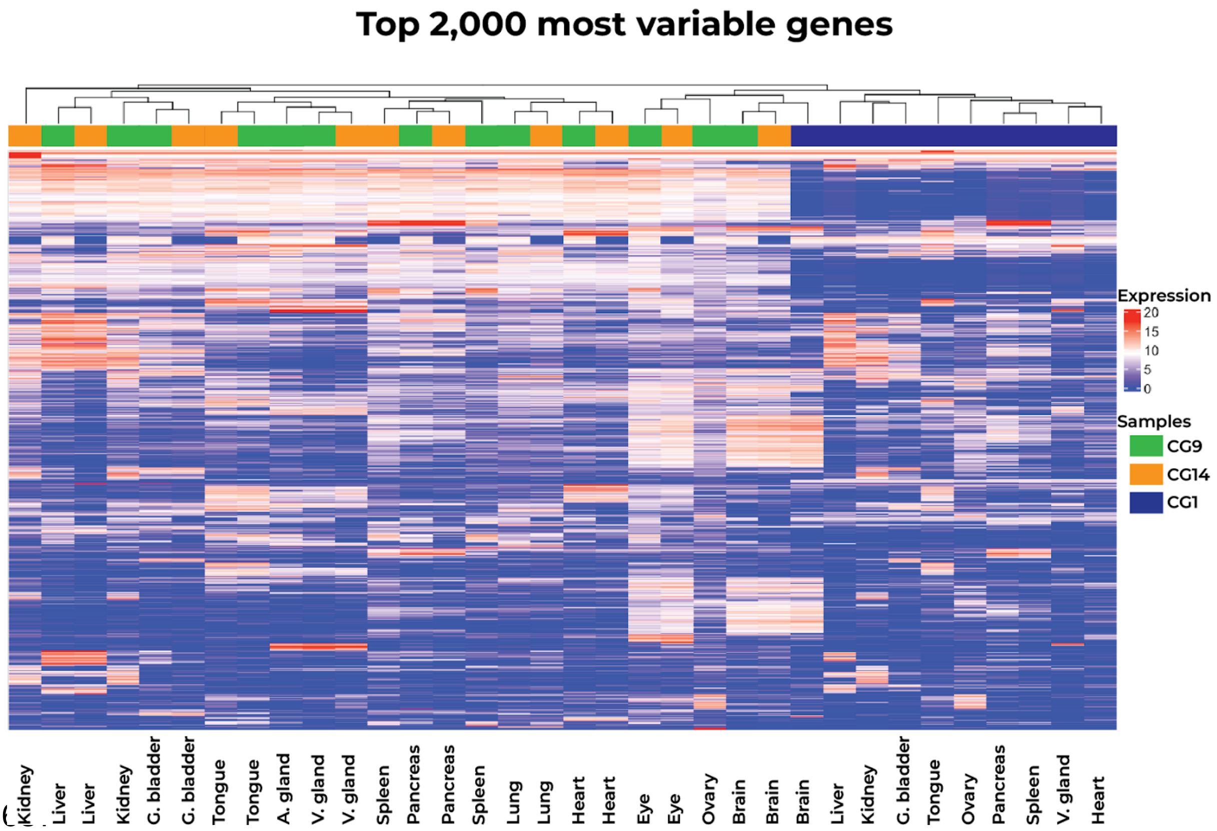

Fig. S4: Heatmap for the 2,000 most variable genes within our three samples, showing a clear batch effect of sample CG1 (possibly due to differences in sequencing time) as well as a high similarity between the putative accessory gland and the venom gland. Each column represents a different sampled tissue. The three different samples are depicted with different colors at the top of the heatmap. Abbreviations are as follows: G. bladder, gallbladder and V. gland, venom gland.

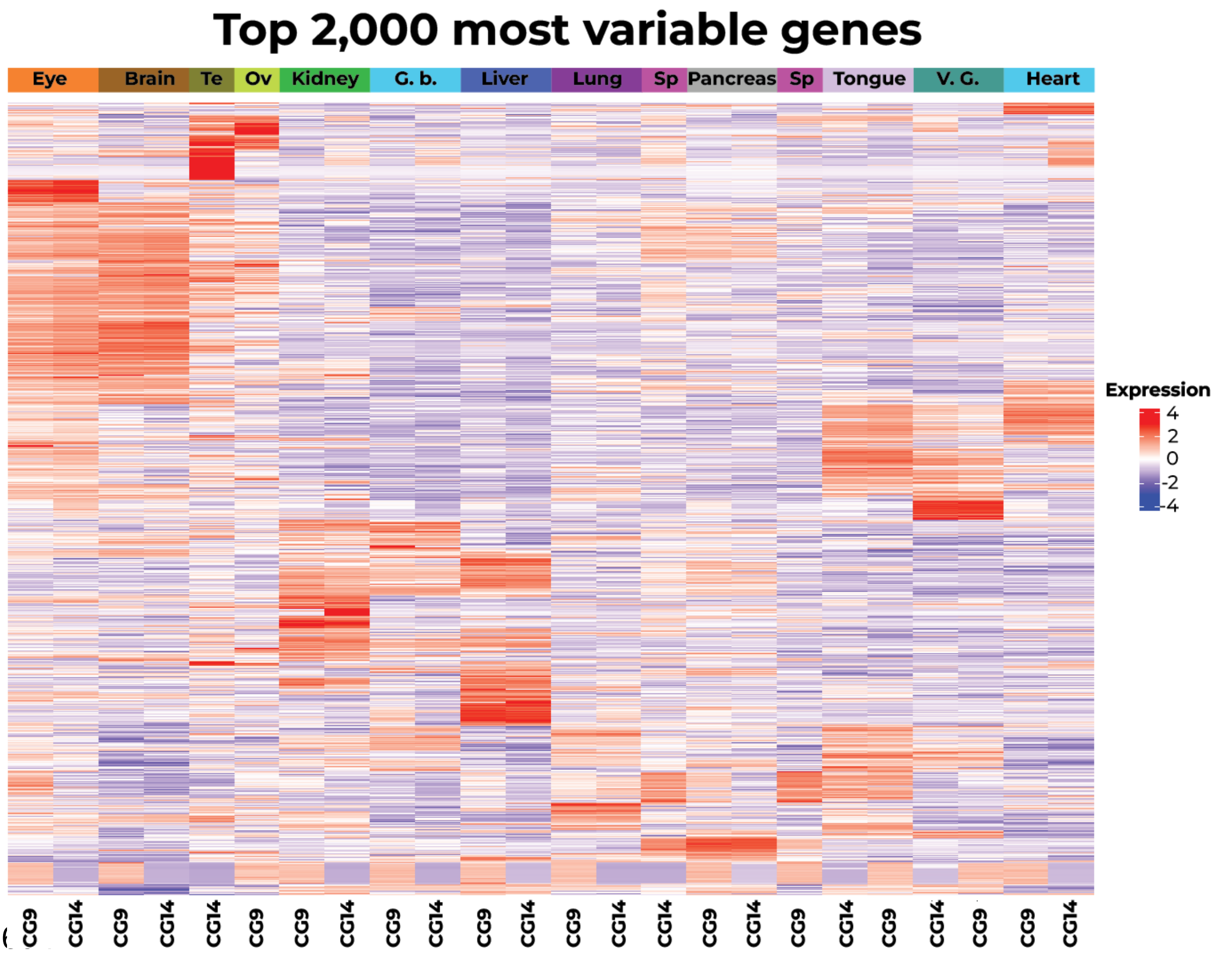

Fig. S5: Heatmap for the 2,000 most variable genes for both samples, reporting highly expressed genes unique for each tissue type. Each column represents one tissue sampled per individual. Expression levels were normalized. Abbreviations are as follows: Te, Testis; Ov, Ovary; G.b., gallbladder; Sp, Spleen and V.G., Venom gland.

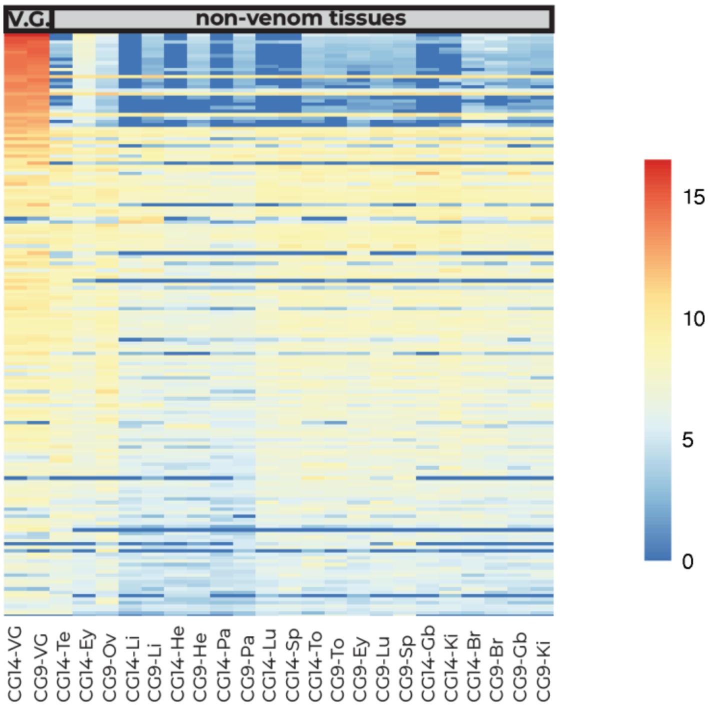

Fig. S6: Heatmap for the 161 upregulated genes found in the venom gland of *C. gasperettii* transcriptome including the 65 putative expressed toxins for both venom gland samples. Each column represents one tissue sampled per individual. Abbreviations are as follows: VG, Venom Gland; Ki, Kidney; GB, Gall Bladder; Lu; Lung; Sp, Spleen; He, Heart; Li, Liver; Pa, Pancreas; To, Tongue; Te, Testis; Ov, Ovary.

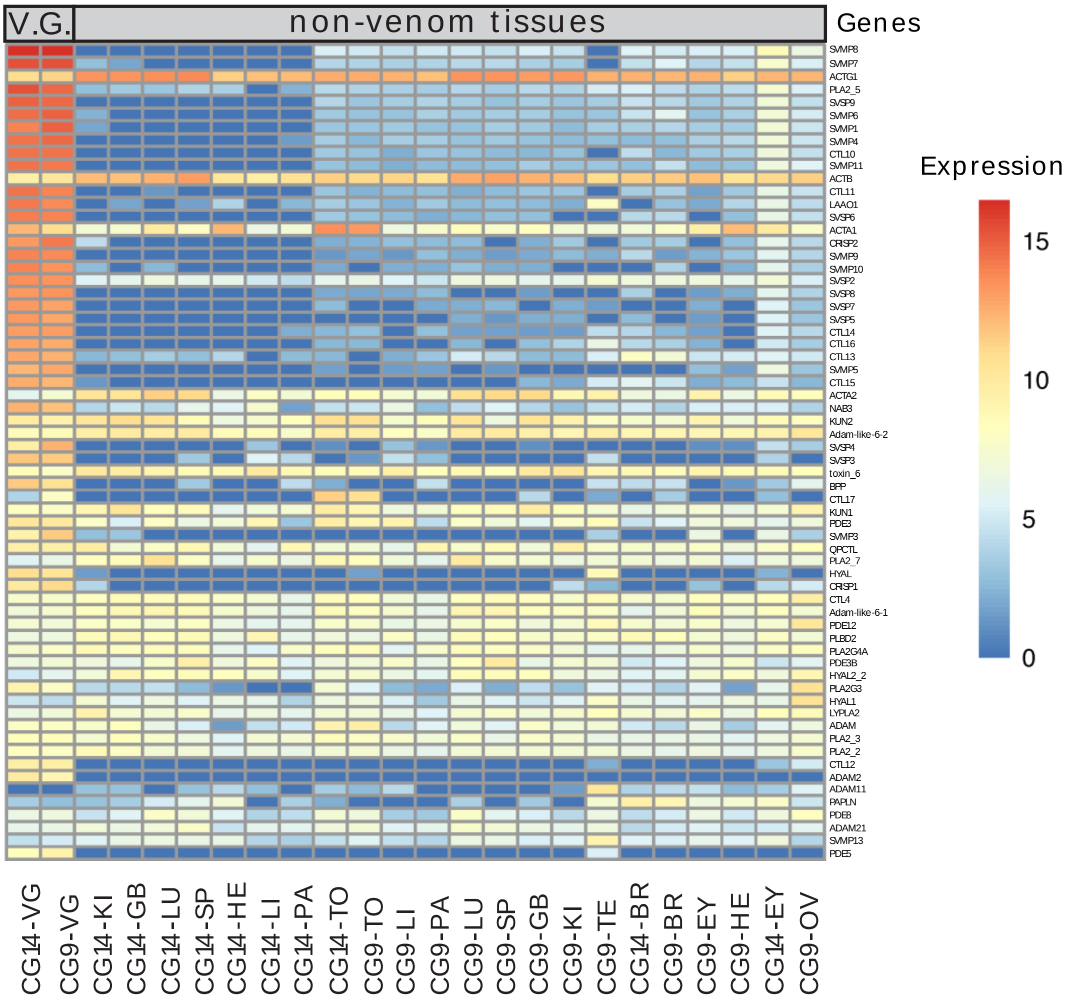

Fig. S7: Heatmap for the venom gland transcriptome for the 65 putative expressed toxins for both venom gland samples. Each column represents one tissue sampled per individual. Abbreviations are as follows: VG, Venom Gland; Ki, Kidney; GB, Gall Bladder; Lu; Lung; Sp, Spleen; He, Heart; Li, Liver; Pa, Pancreas; To, Tongue; Te, Testis; Ov, Ovary.

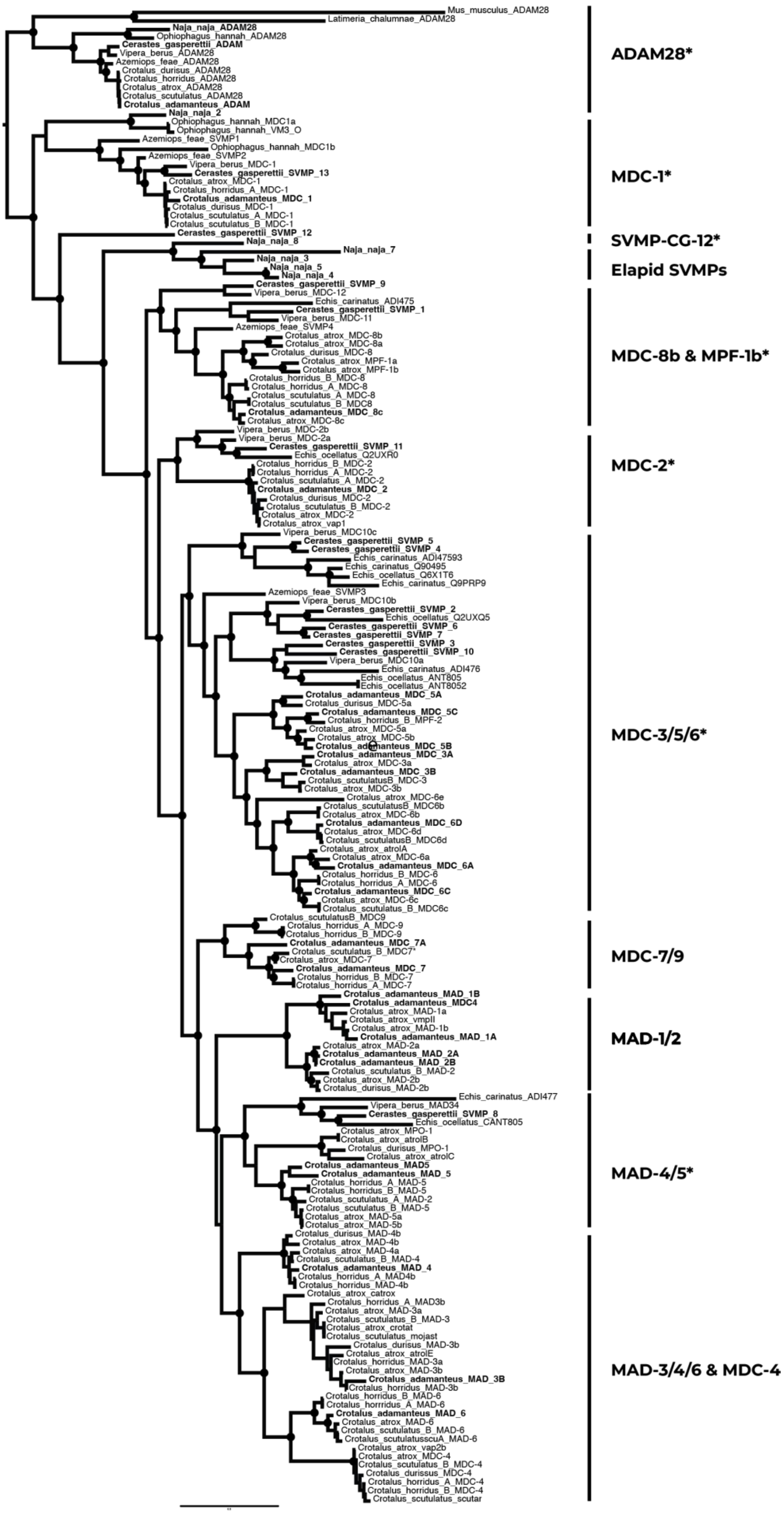

Fig. S8: Maximum

likelihood phylogeny for SVMP genes and its non-toxic paralog (ADAM28). Genes for *Cerastes gasperettii* are highlighted in bold. Toxin groups are identified following previous categorizations. Asterisks indicate if *Cerastes gasperettii* genes are present in that specific group. Branch support with aBayes values higher than 90 are depicted as circles.

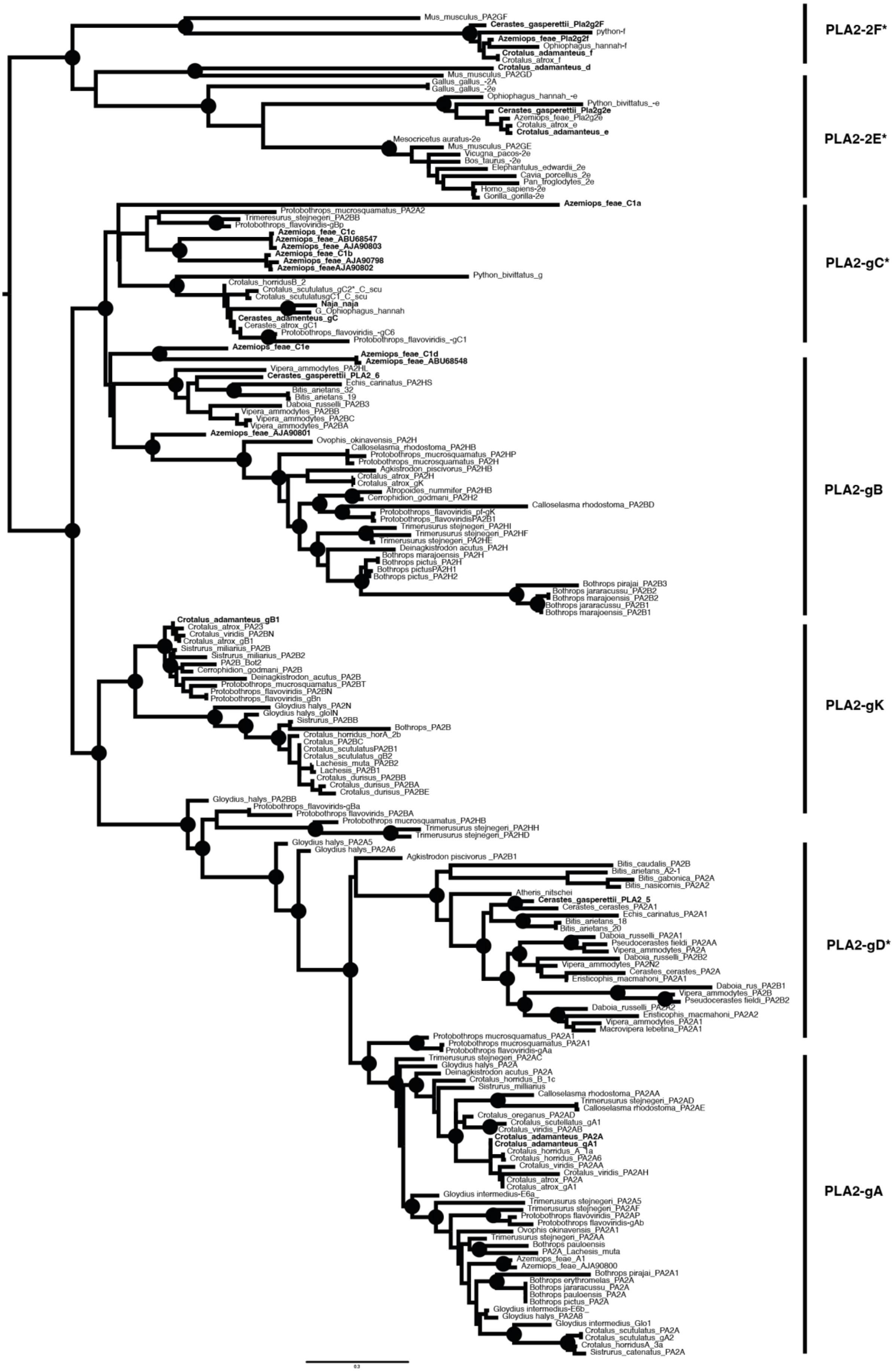

Fig. S9: Maximum likelihood phylogeny for PLA_2, with the two non-toxic genes as outgroups (PLA_2-

2F and PLA$_2$-2E). Asterisks in group labels indicate if *Cerastes gasperettii* genes are present in that specific group. Branch support with aBayes values higher than 90 are depicted as circles.

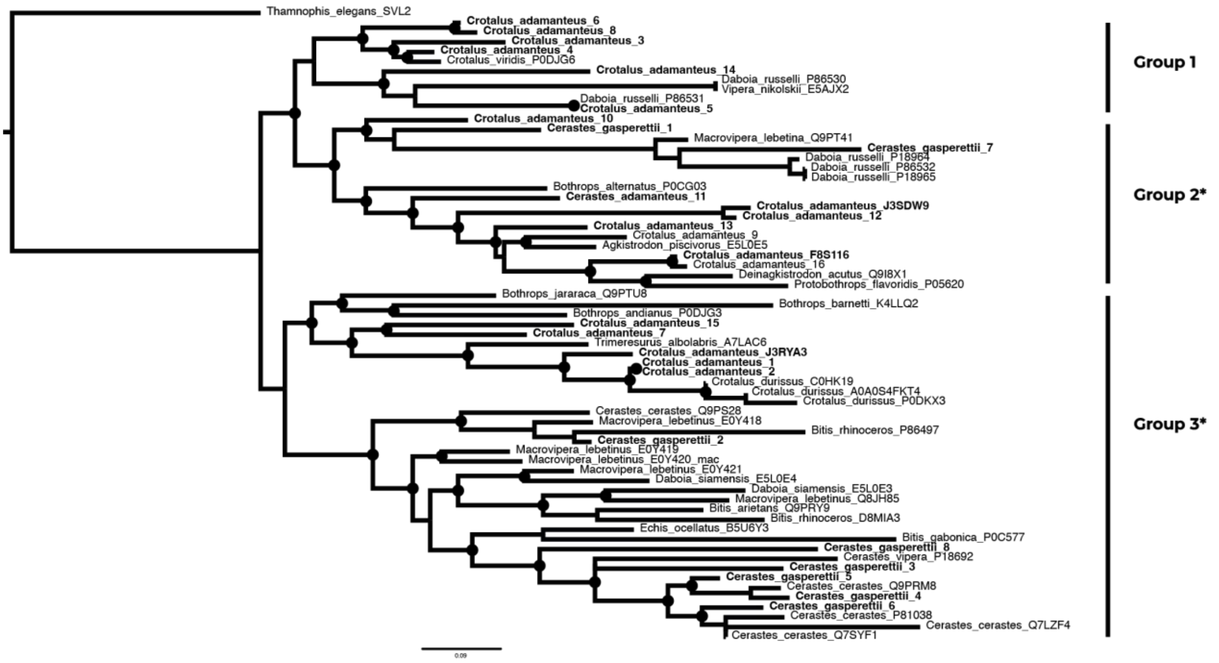

Fig. S10: Maximum likelihood phylogeny for SVSPs, with one sample from *Thamnophis elegans* as outgroup. Asterisks in group labels indicate if *Cerastes gasperettii* genes are present in that specific group. Branch support with aBayes values higher than 90 are depicted as circles.

Table S1: Individuals sampled in this study with their sex, sampling coordinates and data sequenced.

ID	Sex	Latitude	Longitude	Data sequenced
CG1	Female	25.284690	55.687860	HiFi, Omni-C, Illumina, RNA-seq, Iso-seq, Proteome
CG9	Female	25.284690	55.687860	RNA-seq
CG14	Male	25.284690	55.687860	RNA-seq

CN6134	-	UAE	UAE	Proteome
CN6135	-	UAE	UAE	Proteome

Table S2: Id, tissue type and number of reads sequenced per sample.

ID	Tissue	Reads
CG9	Tongue	44,672,733
CG9	Venom gland	41,124,132
CG9	Eye	41,951,109
CG9	Brain	42,800,966
CG9	Heart	40,715,947
CG9	Lung	42,518,938
CG9	Liver	42,251,137
CG9	Gallbladder	42,738,665
CG9	Spleen	40,909,550
CG9	Pancreas	40,527,010
CG9	Ovary	41,118,336
CG9	Kidney	40,620,023
CG9	Accessory gland	44,114,293
CG14	Tongue	41,455,346
CG14	Venom gland	41,035,764
CG14	Eye	40,753,220
CG14	Brain	43,413,973
CG14	Heart	42,338,980
CG14	Lung	42,068,410
CG14	Liver	21,549,210
CG14	Gallglabbder	50,571,941

CG14	Spleen	45,447,235
CG14	Pancreas	50,223,941
CG14	Testis	47,495,900
CG14	Kidney	45,945,776
CG1	Heart	47,362,067
CG1	Brain	45,740,571
CG1	Kidney	50,758,869
CG1	Gallbladder	40,546,711
CG1	Liver	48,058,958
CG1	Spleen	44,752,981
CG1	Tongue	46,837,490
CG1	Pancreas	45,023,783
CG1	Venom gland	49,775,424
CG1	Ovary	48,703,420

Table S3: Different types of repetitive elements masked within the genome:

Element	Number of elements	Length (bp)	Percentage
Retroelements	1524124	493932584	30.25 %
SINEs:	339152	55265721	3.38
Penelope	124778	19471740	1.19
LINEs:	988815	347028895	21.25
CRE/SLACS	0	0	0.00%
L2/CR1/Rex	480371	137654000	8.43
R1/LOA/Jockey	579	99034	0.01
R2/R4/NeSL	41793	10873028	0.67

RTE/Bov-B	128092	79663597	4.88
L1/CIN4	207974	95913575	5.87
LTR elements:	196157	91637968	5.61
BEL/Pao	16545	5263265	0.32
Ty1/Copia	25582	15088781	0.92
Gypsy/DIRS1	102598	63604234	3.90
Retroviral	50617	7642063	0.47
DNA transposons	707499	111444059	6.83
hobo-Activator	265944	30679712	1.88
Tc1-IS630-Pogo	227637	58877559	3.61
En-Spm	0	0	0.00%
MULE-MuDR	44	3962	0.00%
PiggyBac	138	6619	0.00%
Tourist/Harbinger	182161	18395721	1.13
Other	0	0	0.00%
Rolling-circles	2242	136656	0.01
Unclassified	205700	42385187	2.60
Total interspersed repeats	-	647761830	39.67
Small RNA	6134	652217	0.04
Satellites	35838	4217238	0.26
Simple repeats	765726	53044358	3.25
Low complexity	97863	6694649	0.41

Table S4: Abundances for the different toxin families identified in the proteome of *C. gasperettii*

Toxin family	Percentage
SVMPi	8.65%

DISI	12.74%
DC domain	0.26%
CRISP	4.34%
PLA2	5.47%
SVSP	37.38%
SVMP-III	22.19%
PDE	0.02%
LAAO	1.71%
CTL	7.25%

References

1. Dussex, N., van der Valk, T., Morales, H. E., Wheat, C. W., Díez-del-Molino, D., von Seth, J., Foster, Y., Kutschera, V. E., Guschanski, K., Rhie, A., Phillippy, A. M., Korlach, J., Howe, K., Chow, W., Pelan, S., Mendes Damas, J. D., Lewin, H. A., Hastie, A. R., Formenti, G., … Dalén, L. (2021). Population genomics of the critically endangered kākāpō. *Cell Genomics*, *1*(1), 100002. https://doi.org/10.1016/j.xgen.2021.100002
2. Hogan, M. P., Holding, M. L., Nystrom, G. S., Colston, T. J., Bartlett, D. A., Mason, A. J., Ellsworth, S. A., Rautsaw, R. M., Lawrence, K. C., Strickland, J. L., He, B., Fraser, P., Margres, M. J., Gilbert, D. M., Gibbs, H. L., Parkinson, C. L., & Rokyta, D. R. (2024). The genetic regulatory architecture and epigenomic basis for age-related changes in rattlesnake venom. *Proceedings of the National Academy of Sciences*, *121*(16), e2313440121. https://doi.org/10.1073/pnas.2313440121
3. Margres, M. J., Rautsaw, R. M., Strickland, J. L., Mason, A. J., Schramer, T. D., Hofmann, E. P., Stiers, E., Ellsworth, S. A., Nystrom, G. S., Hogan, M. P., Bartlett, D. A., Colston, T. J., Gilbert, D. M., Rokyta, D. R., & Parkinson, C. L. (2021). The Tiger Rattlesnake genome reveals a complex genotype underlying a simple venom phenotype. *Proceedings of the National Academy of Sciences*, *118*(4), e2014634118. https://doi.org/10.1073/pnas.2014634118
4. Pardos-Blas, J. R., Irisarri, I., Abalde, S., Afonso, C. M. L., Tenorio, M. J., & Zardoya, R. (2021). The genome of the venomous snail *Lautoconus ventricosus* sheds light on the origin of conotoxin diversity. *GigaScience*, *10*(5), giab037. https://doi.org/10.1093/gigascience/giab037
5. Schield, D. R., Card, D. C., Hales, N. R., Perry, B. W., Pasquesi, G. M., Blackmon, H., Adams, R. H., Corbin, A. B., Smith, C. F., Ramesh, B., Demuth, J. P., Betrán, E., Tollis, M., Meik, J. M., Mackessy, S. P., & Castoe, T. A. (2019). The origins and evolution of chromosomes, dosage compensation, and mechanisms underlying venom regulation in snakes. *Genome Research*, *29*(4), 590–601. https://doi.org/10.1101/gr.240952.118
