# Supplementary material for: Chromosome-level reference genome for the medically important Arabian horned viper (Cerastes gasperettii)
Source: Gigascience. 2025 Jun 6;14:giaf030. doi: 10.1093/gigascience/giaf030 (PMC12143202; doi:10.1093/gigascience/giaf030)
Supplement: giaf030_GIGA-D-24-00269_Revision_1 [file giaf030_giga-d-24-00269_revision_1.pdf]

GigaScience

Chromosome-level reference genome for the medically important Arabian horned viper (Cerastes gasperettii)

--Manuscript Draft--

**Manuscript Number:**	GIGA-D-24-00269R1
**Full Title:**	Chromosome-level reference genome for the medically important Arabian horned viper (Cerastes gasperettii)
**Article Type:**	Research
**Funding Information:**	Ministerio de Ciencia e Innovación (PID2021-128901NB-I00) — Dr. Salvador Carranza<br>Departament de recerca i Universitats (2021-SGR-00751) — Dr. Salvador Carranza
**Abstract:**	Venoms have traditionally been studied from a proteomic and/or transcriptomic perspective, often overlooking the true genetic complexity underlying venom production. The recent surge in genome-based venom research (sometimes called "venomics") has proven to be instrumental in deepening our molecular understanding of venom evolution, particularly through the identification and mapping of toxin-coding loci across the broader chromosomal architecture. Although venomous snakes are a model system in venom research, the number of high-quality reference genomes in the group remains limited. In this study, we present a chromosome-resolution reference genome for the Arabian horned viper (Cerastes gasperettii), a venomous snake native to the Arabian Peninsula. Our highly-contiguous genome allowed us to explore macrochromosomal rearrangements within the Viperidae family, as well as across squamates. We identified the main highly-expressed toxin genes compousing the venom's core, in line with our proteomic results. We also compared microsyntenic changes in the main toxin gene clusters with those of other venomous snake species, highlighting the pivotal role of gene duplication and loss in the emergence and diversification of Snake Venom Metalloproteinases (SVMPs) and Snake Venom Serine Proteases (SVSPs) for Cerastes gasperettii. Using Illumina short-read sequencing data, we reconstructed the demographic history and genome-wide diversity of the species, revealing how historical aridity likely drove population expansions. Finally, this study highlights the importance of using long-read sequencing as well as chromosome-level reference genomes to disentangle the origin and diversification of toxin gene families in venomous species.
**Corresponding Author:**	Gabriel Mochales Riaño<br>Institute of Evolutionary Biology: Institut de Biologia Evolutiva<br>barcelona, Barcelona SPAIN
**Corresponding Author Secondary Information:**	
**Corresponding Author's Institution:**	Institute of Evolutionary Biology: Institut de Biologia Evolutiva
**Corresponding Author's Secondary Institution:**	
**First Author:**	Gabriel Mochales Riaño
**First Author Secondary Information:**	
**Order of Authors:**	Gabriel Mochales Riaño<br>Samuel R. Hirst<br>Adrián Talavera<br>Bernat Burriel-Carranza<br>Viviana Pagone<br>Maria Estarellas<br>Theo Busschau

	Stéphane Boissinot
	Michael P. Hogan
	Jordi Tena-Garcés
	Davinia Pla
	Juan J. Calvete
	Johannes Els
	Mark J. Margres
	Salvador Carranza
**Order of Authors Secondary Information:**	
**Response to Reviewers:**	Reviewer #1: In the manuscript entitled 'Chromosome-level reference genome for the medically important Arabian horned viper (Cerastes gasperettii)', the authors assembled a high-quality chromosome-level reference genome for the Arabian horned viper (Cerastes gasperettii), a special Viperid species, which is an important data resource. Combined with multi omics data, the authors characterized the genome, conducted the analysis of toxin gene family, and identified a novel SVMP gene. The research is with great significance for the revelation of the origin and diversification of snake venom. Overall, I think the science and findings of the study are meaningful and merit publication, but in its current form, there are some issues should be noticed:<br><br>We thank reviewer 1 for his/her comments of our manuscript. We provide below a point-by-point answer to all his/her questions.<br><br>1. It should be noted that Fig. 1 and Fig. 2 both have unidentified border lines.<br><br>Sorry for that, we have now fixed it.<br><br>2. In all phylogenetic trees presented by the manuscript, it would be better for authors to indicate all species information.<br><br>The three toxin phylogenies have been modified and now all the tips include their species information.<br><br>3. I'm curious if the authors considered period differences in sampling, for example differences in venom glands after venom harvest or in the resting state, which could affect the analysis especially the transcriptome.<br><br>Before sampling the venom glands, venom was extracted, and snakes were allowed to recover for four days to maximize the venom gland transcription. This was absent from the text and now it has been added.<br><br>4. In the transcriptomics section, the author stated that the batch effect of CG1 was due to the low mapping of that sample to our reference genome. It is a misinterpretation to me as CG1 itself is the genome sequencing sample. The authors should further explain for this.<br><br>RNA-seq sequencing was not produced at the same time for the three samples, we first sequenced the rna-seq samples from the reference genome individual and later on the two other samples. We do not find any explanation to this, but both the low mapping as well as the different clustering of the tissues hampers the analyses of the three samples together. For that, we decided to exclude it from the analyses. We have clarified it in the text.<br><br>5. The authors need to ensure that all data generated by the manuscript is accessible and information about the data is not currently available.<br><br>All the NGS data was uploaded to ncbi and all the non-NGS data was uploaded to GigaScience’s internal repository. We will talk with the Editor to solve this issue, raised by several reviewers, we are sorry for that.

6. Please check the references to ensure that the formatting meets the publisher's requirements, e.g., some Latin names of species requiring italics.

This has been fixed now, thanks.

Reviewer #2: Mochales-Riaño et al. present a high-quality genome assembly for the Arabian horned viper and provide a suite of genomic analyses related to synteny, toxin gene evolution and expression, genomic diversity, and demographic history of this and related species. This species is a valuable addition to existing snake genome resources given its medical significance and the current underrepresentation of genomes for Viperidae. I also appreciate that the authors sequenced the heterogametic sex and successfully assembled both sex chromosomes. I do have a few questions and concerns about the manuscript in its current form that I highlight below. Most notably, I feel that the arguments throughout the manuscript about toxin gene copy number correlating with proteomic abundance to be poorly supported and generally problematic given the data and analyses that the authors present. I suggest that the authors reevaluate these claims, and either provide additional analyses in an effort to support these claims or otherwise remove them from the manuscript, as I don't think they are ultimately crucial to the value of this genome report.

We thank reviewer 2 for his/her comments of our manuscript. We provide below a point-by-point answer to all his/her questions.

Introduction:
I find the argument being made in the sentence beginning "Previous works have shown that changes in gene regulation" a bit confusing. Rather than this arguing that studying the expression of venom genes is "insufficient," I think that this instead argues that transcriptomic and proteomic data are critical for studying venom in conjunction with annotated genome sequence. You could for example have a species with 20 copies in a particular tandem array, but only two of them are ever expressed at biologically meaningful levels and thus contribute proteins to the excreted venom. Knowing both the total number of copies in the genome and the number that are actually contributing to the venom proteome are both valuable and necessary for understanding the evolution of that gene family, its role and significance in venom phenotypes, etc.

We agree with reviewer 2 about this point and we have accordingly changed this section of the introduction to highlight how the combination of these techniques are necessary to study the evolution of venom. Now the paragraph reads like this:
“Previous works have shown that changes in gene regulation can result in the activation and deactivation of venom-coding genes at all taxonomic levels and within the same individual (Avella et al., 2022; Hogan et al., 2024; Margres et al., 2021; Zancolli et al., 2022), suggesting that transcriptomic and proteomic data are critical for studying venom in conjunction with well annotated reference genomes to disentangle the complete number and biochemical nature of the toxins an individual can potentially transcribe (Drukewitz & Von Reumont, 2019). Ultimately, the study of venom genomics may yield insights into antivenom or drug discovery, as it can identify unexpressed toxin-coding genes that target specific physiological pathways, potentially leading to new therapies for human illnesses including but not limited to cancer (Casewell et al., 2013; King, 2011; L. Li et al., 2018).”

I'm also not sure I follow the logic of the next sentence. Why exactly would the identification of specifically "unexpressed" toxin genes be particularly notable for antivenom, drug discovery, therapeutics, etc.?

We have modified this sentence, now it reads like this:

“Ultimately, the study of venom genomics may yield insights into antivenom or drug discovery, as it enables the identification of unexpressed toxin-coding genes. These genes, often overlooked by transcriptomic or proteomic approaches unless ontogeny

analyses or in-depth venom expression studies are performed, may target unique physiological pathways. Such discoveries could lead to novel therapies for human illnesses including but not limited to cancer.”

"We deciphered numerous genomic attributes of this species including its genetic diversity and failed to find evidence of inbreeding" - lack of inbreeding is never discussed in the context of the heterozygosity results, but is pitched here as a major result of the paper. Did the authors have a priori expectations regarding inbreeding in this species?

It is true that we did not have a clear expectation of the genomic status. For this reason, we have now changed it to this:

“We deciphered numerous genomic attributes of this species, highlighting its adequate levels of genetic diversity”

Methods:
"Gene counts per gene…" - should this be "Gene expression counts per gene…"?

Reviewer 2 is right, we have included the word expression in this sentence, thank you for pointing it out.

Venom gland RNA-seq data was generated from three animals, but proteomic data was generated from a pool of two other animals. This is not ideal for linking gene expression to venom proteome composition, where you really would want venom collected from the same animals you are getting venom gland RNA from. This is especially true is there is intraspecific variation in venom phenotypes within this species. The latitude and longitude are not provided for the two proteome samples. Were these collected from the same latitude and longitude as the RNA-seq animals?

Intraspecific venom variation has not been described for this species, meaning that reviewer 2 is right and we should consider this. However, we were not able to extract venom from the reference individual and we do not have the exact coordinates for the two proteomic individuals. Although both individuals are from the same country as the reference genome, we have added that possible differences between transcriptomic and proteomic venom expression may rise due to geographical variation.

For analyses of heterozygosity, the authors map wgs data from diverse species against the cerastes reference and call variants. Why was this approach chosen over instead mapping the data for each species to either that species' reference (i.e., C. viridis and N. naja) or a more closely related species for those without a reference? Presumably that would reduce the potential influence of reference bias on these estimates of heterozygosity?

Yes, reviewer 2 is right and we may have reduced the heterozygosity levels of those species. We have reanalyzed each sample mapping it with the closest reference genome possible.

Results:
"Toxin genes usually found in venomous snakes (see proteome results below) were mainly found on macrochromosomes, although major toxin groups were found on microchromosomes (SVMPs, SVSPs and PLA2; Fig. 1)" this feels a bit contradictory. Maybe just can state that toxin genes were found on both macro and microchromosomes?

We have modified this section accordingly. Now it reads:

“Toxin genes were found in both macro- and microchromosomes (Fig. 1).”

"Finally, we also found a battery of 3FTxs and myotoxin-like genes, but they were not represented in our RNA-seq dataset (see below)." The authors do not further discuss

Powered by Editorial Manager® and ProduXion Manager® from Aries Systems Corporation

this result as implied by "(see below)," unless that was simply referring to subsequent discussion of RNA-seq data. From what I can tell, these are also not present in the proteomic data, correct?

Exactly, we did not detect neither transcripts nor proteomic data for those two gene groups. We have now clarified it in the text.

"The venom gland transcriptome contained a total of 7,237 genes expressed (TPM > 500), including a total of 65 putative toxin genes. Differential gene expression analyses revealed a total of 161 genes (33 putative toxin genes) that were differentially upregulated (FC > 2 and 1% FDR) in venom glands compared to other tissues (Fig. 3A)." Figure 3A only shows 10 toxin genes with "unique" expression in the venom gland, not the 161 upregulated toxin genes as implied here. The authors should add a heatmap with these 161 genes to the supplement, if not to Figure 3 (guessing it might not fit).

Thanks for pointing that out, we have added now two extra supplementary heatmaps (Fig. S6 and S7), one including the upregulated genes in the venom gland and a second one only focusing on the toxins.

Fig 3: The authors do not discuss the lack of unique/upregulated expression evidence for PLA2s and Disintegrins in Fig 3A, despite their contribution to protein composition in Fig 3B. For disintegrins in particular, they represent a higher proportion of the venom proteome than CTLs and CRISPs, yet there is no evidence presented for high expression in these genes. What do the authors think is going on here? Could this be a technical issue related to the processing of the RNAseq data, perhaps related to the small size of these genes? Alternatively, could this be indicative of a mismatch between venom phenotypes of the animals used to generate transcriptomic versus proteomic data?

We agree with the reviewer that we did not discuss some of the differences between RNA-seq and proteomic datasets, possibly (as suggested) due to individual differences. We have now included it along the text.

In the text, the authors state "These genes, together with other SVMPs, SVSPs, Disintegrins (DISI) and C-type lectins (CTL), were highly expressed in the venom gland and form the core toxic effector components of the venom" but again there is no presented evidence for DISI expression in particular. Are these genes included in the 161 upregulated genes in the venom gland?

This sentence was referring to the highly expressed genes in the venom gland, and as the reviewer mentions, DISI are not there. DISI has been removed from the sentence, thank you for pointing that out.

The authors only present proteomic data in the form of a pie chart of overall composition grouped by toxin family (Fig 3B). Does the proteomic data generated here provide individual gene-level proteomic abundance estimates? If so, this would be valuable to include, especially in support of the authors claims about gene copy number being correlated with protein abundance. For example in Figure 3, SVMP9 and SVMP10, and to a lesser extent SVMP13, are highly expressed and therefore possibly/likely the major contributors to SVMPs in the proteome. Is the SVMP section of the pie chart in Fig 3B dominated by proteins from these 3 genes?

We have now added one supplementary table (Table S4) with the values of figure 3B and a supplementary file including the proteomic abundance estimated at the gene-level. The claim about gene copy number being correlated with protein abundance is discussed below.

"We studied venom evolution within the most abundant toxin groups (i.e., SVMPs and SVSPs, as well as PLA2)." PLA2s are a relatively low proportion of the venom proteome in Fig 3B, and are not present in the expression heatmap in Fig 3A. Why were these chosen for further investigation over CTL, CRISP, DISI, etc.?

The reviewer is correct that the justification for including PLA2s in our comparative

	analyses is not very robust, as several other toxin families were found to be more abundant. However, genetic and genomic resources for PLA2s within snakes are more abundant than for other toxin families, paving the way to the study of this toxin family.<br><br>"The amplification of SVMP copy numbers is consistent with proteomic results, as SVMPs were the second most abundant component…". Related to my comment above, are all/many of these copies expressed in proteomic, or at least transcriptomic, data? As the data is currently presented, it appears that a small number of SVMPs are highly expressed and thus likely contributing to the proteome. This does not support, and might in fact contradict, the authors claim that there is an association with increased copy number and contribution to the proteome.<br><br>Regarding this fact, what we meant to say is that the toxin families that have undergone the highest number of gene duplication events throughout their evolution are also the most abundant in the proteome, regardless of whether all the genes in that toxin family are expressed or not. However, we agree with the reviewer that this statement could cause confusion, and therefore we have decided to remove it as well as other references for this claim along the text.<br><br>Related to this, and more generally, the authors do not present a convincing argument for the relationship between gene copy number and the resulting percentage of a given toxin gene family in the proteome. If copy number is directly related to the resulting amount of a toxin in the proteome, the authors would need to show that many/all of those copies are expressed in the transcriptomic data, and that proteins produced from those genes are present and contributing to the venom proteome (beyond just the total percentage for the family). Further, making any links between copy number and percent overall composition in the proteome is problematic, because it inherently is impacted by copy number variation and expression of all the other toxin genes. You could, in theory, have copy number expansion in a species where all the genes are expressed and contribute to the proteome, but no overall change in the percent of that toxin family in the proteome if other toxin families have<br>also expanded and/or are expressed more highly. Related to this, there is currently no obvious baseline to compare against in order to make these claims that expansion has resulted in higher venom proteome composition (i.e., a situation where we have fewer SVMP gene copies and a corresponding lower percentage of SVMP proteins in the venom proteome). This would potentially require comparison across species and/or populations with differing copy number, etc.<br><br>Thank you for this explanation, now it is clearer your specific point. We are aware that our data does not support this claim, and for this reason, we have decided to remove it from the manuscript.<br><br>My concerns above also apply to the interpretation of SVSP results: "The high number of SVSP genes found (although lower than in Crotalus adamanteus) were in line with the proteomic results, as SVSPs are the most abundant toxin in the proteome (Fig. 3B)." Further, C. adamanteus has a larger number of SVSP genes than C. gasperettii, yet a lower percent composition of SVSPs in the proteome (Margres et al. 2014), emphasizing my concerns about associating copy number and percent composition.<br><br>We agree with the reviewer and, as mentioned earlier, we have removed these claims from the manuscript. Thanks again for pointing it out.<br><br>Could the two large Group 2 SVSPs in Fig 4E be misannotations of multiple genes? Looking at the adamanteus genes above these, there genes starting and ending at roughly the same position the start and end of these large SVSPs, making me wonder if there are multiple cerastes genes that were annotated as one. In my own experience, I have seen similar situations where FGENESH+ was fed a large region containing multiple genes and annotated multiple genes together as one, so might just be worth double checking that that hasn't happened here. Alternatively, could these be gene fusions? If that's the case, that would presumably complicate the gene tree analyses, correct? i.e., these genes would probably need to excluded from those analyses.

The reviewer is right, thank you very much for spotting this. We have reannotated the region and we have identified the different genes. The different figures, as well as the annotation has been modified accordingly.

Reviewer #3: Dear Authors, thank you for compiling this resource and the manuscript. I apologise for the delay in my review. I have read the manuscript with great interest. I have some major concerns that need be addressed and a lot of minor concerns. Without line numbers, it was difficult to provide comments. I have chosen to write the part of the sentence that my comment refers to for you to consider for improvements.

We thank reviewer 3 for his/her comments of our manuscript. We are sorry for the lack of line numbers, they have been added now. We provide below a point-by-point answer to all his/her questions. We appreciate the thorough review provided, specially the minor concerns.

Major concerns:

- Abstract can include quantitative values for some key results such as the genome size, contiguity (e.g.N50, L90) and quality metrics (e.g. BUSCO) of the genome assembly among other result claims listed in the abstract.

We have now added quantitative metrics for the reference genome within the abstract.

- Venom as the keyword can perhaps be described/defined. Authors interchangeably use "venom", "toxin", "venom toxin", genes coding venom proteins. I strongly suggest the use of consistent terminologies that are well defined in the manuscript.

We have added venom as a keyword and we have carefully revised the manuscript regarding the use of the words venom, toxin, venom toxin and genes coding venom proteins, thanks for pointing that out.

- Methods need elaborate descriptions about reagents, procedures including for library preparations, sequencing machines, library kits and versions, etc. These are relevant for downstream analyses.

These details have been included, specially following the minor concerns suggested by the reviewer below. We hope it is more clear now.

- For all software, list parameters used, even if default, then explicitly state that "default parameters were used". For all software, list version numbers used for analyses.

We have incorporated parameters and version for the softwares.

- Authors are urged to change "macorsynteny" and "microsynteny" terms to chromosome level and local synteny analyses. This is to avoid confusion related to macro/microchromosomes.

We have accordingly modified the text with regard to both terms.

- "Genomic diversity" analyses use cross-species alignments and variant calling using software and methods developed for same species data. This can introduce significant bias in downstream interpretation and use of the variant data

(heterozygosity measure may be). I suggest removal of this section because of lack of accuracy.

This concern was also raised by other reviewers, and we have decided to analyze each species using the closest available reference genome. This approach aims to minimize, as much as possible, the expected reduction in heterozygosity levels associated with mapping to a more distantly related species.

- Discussion of new discovery is largely lacking. I would appreciate if authors contextualized their results with other discoveries in the field.

We have have increased that section, now it reads like this:
"Our discovery of a novel SVMP gene in C. gasperettii adds to the growing body of work on the dynamic evolution of venom systems. Similar gene expansions and duplications have been observed in other species, such as PLA2 toxin-coding genes found in the venom of Azemiops feae (Myers et al., 2022), highlighting the lineage-specific nature of venom evolution. The gene we identified, possibly arising from an SVMP13 duplication, do not share orthology with genes in other species, suggesting the presence of hidden toxin diversity in venom systems"
- Section headings in Results and Discussions can be changed to reflect main findings instead of "transcriptomics" or "genomic diversity".

Titles have been changed, thank you for the suggestion.

- One of the main findings is about SVMP gene family expansion. However, due to the lack of evidence about assembly accuracy in the region, accurate annotation of copies, and the effect of studying "primary assembly" instead of "haplotype assembly" at this region, I am not convinced of claims made in the paper. Appropriate justification is required for this section.

It may be that there was a misunderstanding regarding the used terms. We did work with haplotypic assemblies, as we obtain them directly from the assembler when incorporating the linkage data. When we mention primary assembly is in fact to remove duplicates present in the primary assembly to transfer them to the secondary assembly, but we do not work with an assembly that combines both haplotypes, this has been specified in the text.
Regarding the assembly accuracy of the region is as high as the current technologies allows it, and as proven by the metrics of the genome, our genome shows contiguity metrics similar to other high-quality reference genomes such as the recently published in PNAS by Hogan et al., (2024), with venom-coding genes assembled in single contigs. Regarding the accurate annotation of the copies, as stated in the manuscript we manually curated the venom regions combining both empirical and manual annotations.

- The nomenclature of SVMP genes is confusing. For example, In Figure 4A, they are all labelled as SVMPs with different colours, but then they are labelled as MDCs and MADs in Figure 4b and Supp Figure 6. Please label each gene in each species with consistent names that can reflect orthologous relationship. This is hard to discern, especially without appropriate species labels in Supp Figure 6.

We have modified the supplementary figures, highlighting in bold the genes involved in Fig. 4, so we hope this makes it easier to spot the orthology between groups. Moreover, now the supplementary figures contain appropriate species labels.

- Provide MSA files and trees used to infer evolutionary history. In the absence of the sequence alignments, and raw tree file, I am unable to evaluate this section of the manuscript. Please provide all required details for reviewers and readers.

All the NGS data was uploaded to ncbi and all the non-NGS data was uploaded to GigaScience's internal repository. We will talk with the Editor to solve this issue, raised by several reviewers, we are sorry for that.

Powered by Editorial Manager® and ProduXion Manager® from Aries Systems Corporation

??: It is not clear what authors mean by the word, term, phrase. Please correct them to convey accurate meaning using established and accepted scientific terminologies and English conventions.

Minor concerns:

Abstract:
- "compousing" ??
- "highly expressed toxin genes": in what tissues?
- "genome-wide diversity" ??
- "toxin gene families in venomous species" -> "toxin gene families in venomous snake species"

All these suggestions within the abstract have been modified, thanks.

Background:
- "Such advances in sequencing technologies": remove "Such"

We have removed it

- "depending on their type, interactions, and the organism": interactions with what?

The word interaction was changed.

- "proteomic (and transcriptomic) approaches": remove parenthesis

We have removed it.

- "to new therapies for human illnesses including but not": since the title contains "medically important", it would be great to include some specific examples here from the literature.

We have incorporated new literature citing examples about it.

- "However, venomous snakes are one": remove "However"

We have removed it.

- "therefore, the fundamental model system": change "fundamental" to "useful"

We have changed it.

- "of medical importance by the World Health Organization (WHO) due to their": provide citation

We provided a citation.

- "Within venomous snakes, the most medically": restructure the sentence for brevity and clarity.

We have modified the sentence.

- "cytotoxic effects (among others)": remove "(among others)"

We have removed it.

- "conducted using a proteomic approach": clarify what proteomic approach mean here.
- "Hirst et al., (in review);" : remove this citation

We have added it as citation as it is already available.

- "within the Viperidae family posses an available reference": change the word

Powered by Editorial Manager® and ProduXion Manager® from Aries Systems Corporation

	"posses" to something meaningful<br><br>We have changed it to contains.<br><br>- "Moreover, employing several -omics techniques": be specific about techniques<br><br>We have specified the techniques used.<br><br>- "We deciphered numerous genomic attributes": be specific<br><br>We have changed it to "We deciphered its adequate levels of genetic diversity."<br><br>Methods:<br>- Describe how blood was extracted from animals with all details including animal handling techniques, body part etc.<br><br>We describe it now.<br><br>- "was stored in RNAlater until RNA extraction": source for RNAlater<br><br>It is from a company, we have specified it<br><br>- "We extracted gDNA from the blood of a female individual": provide additional details such as the quantity of blood used, thawing process, qty of reagents, especially elution buffer etc. Manufacturer protocols may be suited best for mammalian blood (humans, mice) without nucleus in RBCs unlike snakes.<br><br>No modifications were added to the protocol, but we have clarified it.<br><br>- "Then, we sequenced a total of two 8M SMRT HiFi cells, aiming for a ~30x of coverage, at the University of Leiden": provide details of library preparation, sequencing machine etc.<br><br>We have provided additional information.<br><br>- "(including venom glands, tongue, liver and pancreas, among others": Either list all or refer to the table.<br><br>We refer now to the table.<br><br>- "RNA libraries were prepared with the VAHTS": Was the library and sequencing strand specific? Provide complete details on these processes.<br><br>It was strand specific, now it is specified.<br><br>- "8M SMRT HiFi cell containing two Iso-seq HiFi libraries": use correct names of these and also include sequencing machine details.<br><br>We have changed the sentence as well as indicate the sequencing details.<br><br>- "Quality control on HiFi and Illumina reads was assessed using FastQC": correct the phrasing of this sentence<br><br>This has been corrected.<br><br>- "To make an initial exploration of the genome, …..we generated a k-mer profile with Meryl": Explicitly state the purpose of this analysis.<br><br>We have specified it: "In order to initially explore the genome size, heterozygosity levels and coverage data".<br><br>- "Manual curation was performed with Pretext": cite Pretext properly. Explain decisions of this manual curation. i.e. what evidence was used to join or break contigs.

Powered by Editorial Manager® and ProduXion Manager® from Aries Systems Corporation

	Pretext does not have any scientific publication(or at least we did not find it), but we have provided the website.<br><br>- "Then, we ran three iterative rounds of RepeatMasker to annotate the known and unknown elements identified by RepeatModeler and soft-masked the genome for simple repeats": break this sentence into two and explain reasons for running RepeatMasker three times.<br><br>This has been specified.<br><br>- "We used GeMoMa v.1.9": Include all details about the annotations. This sentence is not sufficient for reproducibility. Were the RNAseq data assembled or provided as raw files to GeMoMa. How were they mapped to the genome assembly f<br><br>Details are included now.<br><br>- "published: Anolis carolinensis from Alföldi": Remove the word "from" here as citation is sufficient. Provide details of assembly versions, annotation version, database of annotations etc.<br><br>From was removed.<br><br>- "Crotalus ruber from Hirst et al., (in review)": remove this citation or list it as personal communication<br><br>As above, this has been included as the paper has been recently published.<br><br>- "We previously quality checked and removed the adapters of the RNA-seq data": remove "previously" and provide details on how adapters were removed from RNAseq data<br><br>We removed previously and provided information about how we removed the adapters with fastp.<br><br>- "also removed the adapters for the Iso-seq data": Explain how this was performed.<br><br>Same as above.<br><br>- "We blast our ..": Change all occurrence of "blast" to "BLAST" and specify parameters, if it was BLASTN or BLASTP or something else. This is not clear at all.<br><br>We have changed it and specified it.<br><br>- "we performed additional annotation steps for venom genes.": Details are not complete for reproducibility. State explicitly what decisions were made and how gene structure was determined. This is the main part of the paper and does require accurate details.<br><br>Details are following this sentence, we modified it and we hope now it is more clear.<br><br>- "Whole-genome synteny was explored between": synteny by definition refers to being on the same string/chromosome. Therefore whole-genome synteny as a term doesn't make sense given that genome is divided into chromosomes. Revise it to say "chromosomal synteny"<br><br>This has been changed.<br><br>- "chromosomes assembled in the reverse complement, which were corrected using SAMtools faidx": samtools faidx cannot do this. Explain how this was done.<br><br>We used samtools faidx with reverse-complement and mark-strand options.<br><br>- "After adapter trimming and quality control, we mapped our RNA-seq reads": how

	were adapters trimmed and QC implemented.<br>- "Gene counts per gene": change gene counts to read counts<br><br>This has been clarified now.<br><br>- "Differential expression analyses were carried out": requires additional details such as filters applied for the count, groups compared, statistical model, multiple testing correction methods.<br><br>This has been now added: "Prior to analysis, genes with fewer than 10 counts across all samples were filtered out. For comparisons, we defined two groups: venom glands versus all other tissues. DESeq2 employs a negative binomial generalized linear model to estimate differences in gene expression, and the p-values were adjusted for multiple testing using the Benjamini-Hochberg method to control the false discovery rate (FDR). Genes with an adjusted p-value < 0.01 and a fold change > 2 were considered significantly differentially expressed."<br><br>- "characterize the venom arsenal of Cerastes gasperettii": change the arsenal word.<br><br>Changed.<br><br>- "Fragmentation spectra were matched against a customized database including the bony vertebrates taxonomy dataset of the NCBI non-redundant database": revise for accuracy<br><br>This has been revised.<br><br>- "Unmatched MS/MS spectra were de novo sequenced": spectra were sequenced how??"we used blast, incorporating both toxin and non-toxin paralogs": change blast to BLAST and provide additional details about the tool used<br><br>This was chaned and we provided additional details about it.<br><br>- "Then, we aligned those regions using Mafft (Katoh": provide coordinates of these regions for future research in each assembly<br><br>Coordinates are now provided.<br><br>- "history for the main groups of toxins (i.e.,": parenthesis is not closed.<br><br>We have now closed it.<br><br>- "we also included other non-toxin paralogous genes from nontoxic species (for details about this see Supplementary Information": where do I look into the supplementary information? Be very clear. Provide coordinates of regions that were compared.<br><br>Supplementary information was provided to GigaScience. We already have told them that reviewers were not able to access the data.<br><br>- "When needed, we translated CDS": when was this needed? Explain.<br><br>Now it is explained: "When nuclear sequences were obtained".<br><br>- "built a phylogeny for each of the toxin groups using Phyml": I presume that this is done with translated CDS sequences in toxin genomic regions. Please clarify.<br><br>Yes, we have clarified it.<br><br>- "Heterozygous positions were obtained from bam files with Samtools v1.9": provide details as to how this was done. Samtools doesn't have features to operate at a site level and therefore I am confused.

We used the mpileup function of samtools, now it is clarified.

- "Filtered reads were mapped against the new reference genome of Cerastes gasperettii using the bwa mem algorithm": bwa mem is designed for same species comparisons. Here you have used it for cross-species. Provide justification and perhaps biases it may have introduced for distantly related species.

We have mapped the other species to closer reference genomes and every sampled was handled independently. This is now clarified.

- "SNP calling was carried out …": This is not appropriate as models assume same species data. You have used cross-species alignments, which can be highly biased.

Same as above, thanks for pointing it out.

Results and Discussion:
- "PacBio HiFi (~40x), Hi-C (~60x) and Illumina data (~78x)": change to number of base pairs. 40x for a genome of 2GB is 80GB data and for genome of 1GB size, it is 40GB data. Before sequencing and assembly, the genome size cannot be known.
This has been changed to: "PacBio HiFi (65 Gbp of data), Hi-C (96 Gbp of data) and Illumina data (135 Gbp of data)".

- "After manual curation, we enhanced the scaffolding parameters of our genome": what was done as manual curation. Please specify.

It is an intrinsic outcome of the manual curation, we have modified it for a better understanding.

- "~228 times more contiguous than the Anolis sagrei genome": how is 228 more measured. How is this useful as a metric without the known ground truth. Assemblies can and do have errors.

It is true that assemblies have errors and they are not complete, but the contiguity value is a comparable metric between genomes, so one genome can be 228x more contiguous than another one, although it is not complete.

- "27,158 different protein-coding genes within our assembly": this seems large compared to other species. Can you elaborate or compare these numbers with other species.

We have recently published a gecko reference genome (Burriel-Carranza et al., 2024 Mol Ecol) with a total of 39,360 protein-coding genes, compared to which other species it is a large number?

- "Toxin genes usually found in venomous snakes (see proteome results below) were mainly found on macrochromosomes, although major toxin groups were found on microchromosomes (SVMPs, SVSPs and PLA2; Fig. 1)." : please revise this statement. Two part of the sentence are saying opposite things. Second provide coordinates of these genes as GFF/BED file as supplementary file with their exon structure annotations for others to reuse this information.

This was modified, as also indicated by another reviewer, coordinates are also provided in a previous comment of the same reviewer.

- "showed a great level of similarity between Cerastes gasperettii and Crotalus adamanteus": provide quantitative metrics for "great" level of similarity.

This sentence has been now modified.

- "we found several fission events in the A. sagrei genome,": Since A. sagrei genome is not contiguous and chromosome scale, you cannot infer fissions as it may be artefact of non-contiguous assembly. If that is not the case, provide evidence of this.

Anolis sagrei genome is at chromosome level as indicated in the publication

https://www.nature.com/articles/s42003-022-04074-5

- "The last four…": Belongs in methods

This has been now moved to methods.

- "Macrosyntenic differences between lizards and snakes": this is very superficial discussion point. Please remove it or strengthen it with evidence.

We removed it, as it is not the point of the analyses or the paper.

- "Heatmap analyses with the most 2,000": Revise this statement. It doesn't make sense. E.g. Heatmap is a visualisation technique and not analyses method.

Thanks, we have removed it.

- "We studied venom evolution within the most abundant toxin groups": rewrite the sentence for clarity and brevity.

We have modified the sentence.

- "After a thorough manual curation": Explain what was this manual curation process clearly and the purpose of it.

This was explained in materials and methods

- "contiguous tandem repeat SVMPs for": Change "repeat" to "array" because tandem repeat has a different meaning in genomics research context.

Array is more acurate, thanks for the suggestion.

- "flanked by the NEFL and NEFM": Unclear if they are both 5' or 3' of toxin genes. Clarify

This has been clarified.

- "Microsyntenic analyses showed": change to local synteny

This has been chaned along the text.

- "gene copy number variation between": Since these are duplicate copies, clearly state how gene copies were identified. Include details of open reading frames, exon structures, pseudogene status, etc

Gene copies were identified through phylogeny methods, this has been now clarified in the text.

- "we can see an expansion in": Describe number of new copies, their status as intact or not, and sequence similarity between copies. Provide evidence that there is no false duplication due to heterozygous allele collapse in the assembly.

We provide details about it now: “Crotalus adamanteus (22 copies unique to vipers and 10 lineage-specific copies) but also in Cerastes gasperettii (12 copies unique to vipers and one lineage-specific copy)”
Regarding the false duplication due to heterozygous alleles, this was already discussed above. During the assembly process, we removed duplicates from the primary assembly.

- "More genomic data will indicate if SVMP12": Did you mean SVMP13?
No, we mean SVMP12, as it is the new discovered gene.

- "This difference may be expected, as PLA2 only represents around 5% of the proteome for Cerastes gasperettii": This is not true. Proteome doesn't equal to genome in some cases and superficial inference such as this is not warranted.

	We agree with the reviewer this sentence has been removed.<br><br>- For PSMC analyses, please discuss the effect of mutation rate and generation time.<br><br>We have added this sentence: “PSMC results may vary depending on the generation time as well as the mutational rate specified. The absence of species-specific data for this analyses may bias our results, although it is a general consensus in the literature when inferring these demographyc analyses in snakes (e.g. Schield et al., 2019).”<br><br><br>Figures:<br>- Figure 1: Add y-axis scales to the circos plot.<br><br>This has been added now.<br><br>- Figure 1b legend says it is a linkage map, but looks more like HiC contact map. Please edit.<br><br>We have now changed it, thanks.<br><br>- Figure 1b legend also says "including the sex chromosomes", which is not consistent with the circos plot.<br><br>During the HiC contact map, chromosomes were sorted by size, because it was not until the end that we identified the sexual chromosomes and in the final assembly they were moved to the end, for this reason there is this inconsistency.<br><br>- Figure 3A refers to transcriptome and 3b to proteome. Please make this very clear.<br>We have clarified it, thanks.<br><br>- Figure 4A, C and E, label genes consistent with the phylogenetic trees in supplementary figures so readers can know their genomic arrangements.<br><br>We have underscored the genes used in Figure 4A, C and E within the supplementary figures, because Figure 4 is already complex and labelling them would make it even more complex.<br><br>- Figure S4: Discuss why CG1 sample separates from rest of the samples. Seems like a batch effect.<br><br>We have added that information, thank you.
**Additional Information:**	
**Question**	**Response**
Are you submitting this manuscript to a special series or article collection?	No
**Experimental design and statistics**<br><br><br>Full details of the experimental design and statistical methods used should be given in the Methods section, as detailed in our Minimum Standards Reporting Checklist. Information essential to interpreting the data presented should be made available in the figure legends.	Yes

Have you included all the information requested in your manuscript?	
**Resources**<br><br>A description of all resources used, including antibodies, cell lines, animals and software tools, with enough information to allow them to be uniquely identified, should be included in the Methods section. Authors are strongly encouraged to cite Research Resource Identifiers (RRIDs) for antibodies, model organisms and tools, where possible.<br><br>Have you included the information requested as detailed in our Minimum Standards Reporting Checklist?	Yes
**Availability of data and materials**<br><br>All datasets and code on which the conclusions of the paper rely must be either included in your submission or deposited in publicly available repositories (where available and ethically appropriate), referencing such data using a unique identifier in the references and in the "Availability of Data and Materials" section of your manuscript.<br><br>Have you have met the above requirement as detailed in our Minimum Standards Reporting Checklist?	Yes

Powered by Editorial Manager® and ProduXion Manager® from Aries Systems Corporation

Manuscript Click here to access/download;Manuscript;manuscript.docx

Chromosome-level reference genome for the medically important Arabian horned viper (*Cerastes gasperettii*)

Gabriel Mochales-Riaño[1], Samuel R. Hirst[2], Adrián Talavera[1], Bernat Burriel-Carranza[1,3], Viviana Pagone[1], Maria Estarellas[1], Theo Busschau[4], Stéphane Boissinot[4], Michael P. Hogan[5,6], Jordi Tena-Garcés[7], Davinia Pla[7], Juan J. Calvete[7], Johannes Els[8], Mark J. Margres[2], Salvador Carranza[1]

[1] IBE, Institute of Evolutionary Biology (CSIC-Universitat Pompeu Fabra)

[2] Department of Integrative Biology, University of South Florida, Tampa, FL 33620, USA

[3] Museu de Ciències Naturals de Barcelona, P° Picasso s/n, Parc Ciutadella, 08003 Barcelona, Spain

[4] New York University Abu Dhabi, Abu Dhabi, United Arab Emirates,

[5] Department of Biological Sciences, Florida State University, Tallahassee, FL 33306 USA

[6] University of Michigan, Department of Ecology and Evolutionary Biology, Ann Arbor, MI (48109-1085) USA

[7] Evolutionary and Translational Venomics Laboratory, Consejo Superior de Investigaciones Científicas (CSIC) 46010 Valencia, Spain

[8] Breeding Centre for Endangered Arabian Wildlife, Environment and Protected Areas Authority, Sharjah, United Arab Emirates

Corresponding: gabriel.mochales@csic.es

Abstract

Venoms have traditionally been studied from a proteomic and/or transcriptomic perspective, often overlooking the true genetic complexity underlying venom production. The recent surge in genome-based venom research (sometimes called "venomics") has proven to be instrumental in deepening our understanding of venom evolution at the molecular level, particularly through the identification and mapping of toxin-coding loci across the broader chromosomal architecture. Although venomous snakes are a model system in venom research, the number of high-quality reference genomes in the group remains limited. In this study, we present a chromosome-resolution reference genome for the Arabian

horned viper (*Cerastes gasperettii*), a venomous snake native to the Arabian Peninsula. Our highly-contiguous genome (genome size: 1.63 Gbp, contig N50: 45.6 Mbp and BUSCO: 92.8%) allowed us to explore macrochromosomal rearrangements within the Viperidae family, as well as across squamates. We identified the main highly-expressed toxin genes within the venom glands comprising the venom's core, in line with our proteomic results. We also compared microsyntenic changes in the main toxin gene clusters with those of other venomous snake species, highlighting the pivotal role of gene duplication and loss in the emergence and diversification of Snake Venom Metalloproteinases (SVMPs) and Snake Venom Serine Proteases (SVSPs) for *Cerastes gasperettii*. Using Illumina short-read sequencing data, we reconstructed the demographic history and genome-wide heterozigosity of the species, revealing how historical aridity likely drove population expansions. Finally, this study highlights the importance of using long-read sequencing as well as chromosome-level reference genomes to disentangle the origin and diversification of toxin gene families in venomous snake species.

Keywords: Toxin evolution; gene synteny; genomics; transcriptomics; venom

Background

The rise of genomics in non-model organisms has led to an increase in the number of high-quality reference genomes available in recent years (Dussex et al., 2021; Hogan et al., 2024; Margres et al., 2021a; Pardos-Blas et al., 2021; Schield et al., 2019; Suryamohan et al., 2020). Advances in sequencing technologies have catalyzed the study of several complex traits from a genomic perspective, such as coloration, domestication, or venom, among others (Drukewitz & Von Reumont, 2019; Frantz et al., 2020; Margres et al., 2021a; Orteu & Jiggins, 2020; San-Jose & Roulin, 2017). Among these, venom genomic research has been particularly important in enhancing our understanding of the origin, evolution and dynamics of this medically relevant trait (Casewell et al., 2013; Dowell et al., 2016; Giorgianni et al., 2020; Werren et al., 2010). Venom is a potentially lethal cocktail rich in proteins and peptides (from now on referred to as "toxins") which are actively secreted by specialized venom glands (Casewell et al., 2013; Fry et al., 2009). Toxins can have different effects depending on their type, interactions with other molecules, and the organism in which they are introduced, with convergent

outcomes in different taxa (Fry et al., 2009; Zancolli et al., 2022). Historically, venom research has primarily been conducted using proteomic and transcriptomic approaches (see Drukewitz & Von Reumont (2019) and references therein). The identification of venom toxins and the characterization of their evolution using reference genomes is a recent and novel field (Vonk et al., 2013). Previous works have shown that changes in gene regulation can result in the activation and deactivation of venom-coding genes at all taxonomic levels and within the same individual (Avella et al., 2022; Hogan et al., 2024; Margres et al., 2021; Zancolli et al., 2022). This suggests that transcriptomic and proteomic data are critical for studying venom in conjunction with well annotated reference genomes to disentangle the complete number and biochemical nature of the toxins an individual can potentially transcribe (Drukewitz & Von Reumont, 2019). Ultimately, the study of venom genomics may yield evolutionary insights into antivenom or drug discovery, as it enables the identification of unexpressed toxin-coding genes. These genes, often overlooked by transcriptomic or proteomic approaches unless ontogeny analyses or in-depth venom expression studies are performed, may target unique physiological pathways. Such discoveries could lead to novel therapies for human illnesses including but not limited to cancer (Casewell et al., 2013; King, 2011; Li et al., 2018; Vyas et al., 2013). Unexpressed toxin-coding genes are particularly noteworthy as they may represent evolutionary 'reservoirs' of bioactive molecules. These genes could encode toxins with unique mechanisms of action, offering untapped potential for drug discovery or therapeutic innovation.

Venom has evolved independently in multiple groups including cnidarians, molluscs, arthropods, squamates and even mammals (Casewell et al., 2013; Fry et al., 2009). Venomous snakes are one of the most life-threatening animal groups to humans (Williams et al., 2019) and, therefore, a medically relevant model system in venom research. Venomous snakes are a diverse group with more than 600 species (Uetz, 2021), where venom has evolved with the objective of immobilizing and digesting their prey (Fry & Wüster, 2004). From those, more than 370 species have been classified as of medically important by the World Health Organization (WHO) due to their potential severe effects on humans (Gutiérrez et al., 2013). Snakebite is considered a neglected tropical disease, with annual mortality exceeding 100,000 victims worldwide (Gutiérrez et al., 2017; Williams et al., 2019). The most medically important venomous snake families are Elapidae, Viperidae and Atractaspididae (Tasoulis &

Isbister, 2017), although within Colubridae (*sensu lato*) there are certain medically important venomous species as well (Weinstein et al., 2013). Envenomation by certain members of these families can result in a range of pathologies, spanning neurotoxic, hemotoxic, and/or cytotoxic effects depending on the number and composition of toxins. Neurotoxic venoms primarily target the central nervous system and are mainly composed of small proteins including three-finger toxins (3FTs), snake venom phospholipases A_2 group I (SV-G^{I}-PLA_2) or dendrotoxins, and are usually associated with elapid snakes (Ferraz et al., 2019). Conversely, hemotoxic and cytotoxic venoms generally are comprised of large enzymatic proteins and protein complexes, including snake venom metalloproteases (SVMP), serine proteases (SP) or snake venom phospholipases A_2 group II (SV-G^{II}-PLA_2), and are typically associated with viperid snakes (Fry, 2015; Fry et al., 2008; Tasoulis & Isbister, 2017). While these historical classifications have proven to be somewhat useful for treating envenomations medically, recent studies have revealed that the presence of these toxins are not exclusive to specific snake families (Osipov & Utkin, 2023).

Vipers (family Viperidae) are a monophyletic lineage of venomous snakes found across Eurasia, Africa and America (Vitt & Caldwell, 2014), receiving extensive research attention primarily due to their medical relevance (Arnold et al., 2009; Casewell et al., 2009; Pook et al., 2009; Šmíd & Tolley, 2019; Wüster et al., 2008). The majority of venom studies in this group have primarily been investigated using a proteomic approach, with early venom work being highly motivated by the medical field, with a limited number of studies employing genomic approaches (but see Almeida et al., (2021); Margres et al., (2021a); Myers et al., (2022); Schield et al., (2019); Hirst et al., (2024); Hogan et al., (2024)). Sequencing efforts to obtain high-quality reference genomes have mainly focused on pitvipers (Crotalinae subfamily, 11 reference genomes, NCBI last accessed 13 March 2024), especially within the *Crotalus* (*n*=6) genus, and have focused on the study of venom evolution (Gilbert et al., 2014; Hogan et al., 2024; Margres et al., 2021a; Schield et al., 2019; Westeen et al., 2023). Other viperids have also been sequenced (although in lower numbers) from both Azemiopinae and Viperinae subfamilies (one and four, respectively) (Myers et al., 2022; Saethang et al., 2022; Talavera et al., in review). Currently, reference genomes are only available for 16 viper species out of the total 387 total species via the NCBI genomic database (Uetz, 2021). Vipers display extensive variation in venom composition between and

within genera (Ali et al., 2015; Mackessy, 2010) and even intraspecifically (Jan et al., 2002; Zancolli et al., 2019). Such differences are most likely due to the high diversity of venom genes and their different effects on prey but also, at least in some cases, the result of introgression with related species (Jan et al., 2002; Margres et al., 2021b; Smith et al., 2023). This provides an extraordinary opportunity to study trait evolution both at inter- and intraspecific levels.

Native to the Arabian Peninsula, the Arabian horned viper (*Cerastes gasperettii*, family Viperidae) is a venomous snake currently recognized within the highest medical importance category (WHO; accessed July, 2024). Extending from the Sinai Peninsula to southwestern Iran in the north and reaching as far as Yemen and Oman in the south, its distribution is widespread (Fig. S1). Found mainly in sandy habitats, this arid-adapted ground-dwelling snake with generalist requirements (Carranza et al., 2021; Mochales-Riaño et al., 2024; Russell & Campbell, 2015) is one of the most common venomous snakes found in Arabia and is responsible for occasional snakebite envenomations (Al-Sadoon & Paray, 2016; Amr et al., 2020; Schneemann et al., 2004).

In this study, we present a high-quality chromosome-level reference genome assembly for the Arabian horned viper (*Cerastes gasperettii*, NCBI: txid110202), being one of the first within the Viperinae subfamily. Our highly-contiguous genome showcases a high level of similarity at the chromosome level within the Viperidae family with some minor rearrangements with elapids. Moreover, combining genomics, transcriptomics, and proteomics, we characterized the main toxins found in its venom and the location of those toxins in the genome, comparing their evolutionary history and gene copy number variation with other venomous species. We deciphered its adequate levels of genetic diversity. Finally, we reconstructed the demographic history for the species, revealing how historical increases in aridity likely drove population expansions. Overall, the genomic resources generated in this study provide an essential reference resource for forthcoming studies on venom evolution.

Methods

Sampling

Three adult specimens (two females and one male) of *Cerastes gasperettii gasperettii* were used for this study (Table S1). Blood was extracted only from a single female individual (the heterogametic sex, sample CG1) to obtain High-molecular-weight (HMW) genomic DNA (gDNA). We anesthesized the individual, extracted blood from the heart and stored in ethanol and EDTA. For each of the three individuals, we extracted twelve different tissues, including the venom gland, which was stored in RNAlater™ until RNA extraction (Table S1 and Fig. S2). Before dissections, venom was extracted and snakes were allowed to recover for four days to maximize the venom gland transcription. We only extracted the left venom gland per individual, as previous research within the same family has shown that both venom glands provide indistinguishable results (Rokyta et al., 2017).

DNA extraction, library preparation and sequencing

We extracted gDNA from the blood of a female individual (CG1 in Table S1) using the MagAttract HMW Kit (Qiagen) following manufacturer's protocols without modifications. Then, we sequenced a total of two 8M SMRT HiFi cells in a Sequel II PacBio machine, aiming for a ∼30x of coverage, at the University of Leiden. Hi-C libraries were prepared using the Omni-C kit (Dovetail Genomics), following the manufacturer's protocol and using blood stored in EDTA, at the National Center for Genomic Analyses (CNAG), in Barcelona, Spain. The library was paired-end sequenced on a NovaSeq 6000 (2 × 150 bp) following the manufacturer's protocol for dual indexing and aiming for a coverage of ~60x. Finally, we sequenced short-read whole-genome data of the same individual using a NEB Ultra II FS DNA kit; the library was paired-end sequenced on a NovaSeq 6000 (2 × 150 bp) at the Core sequencing platform from the New York University of Abu Dhabi, aiming for ∼70x depth of coverage.

RNA extraction, library preparation and sequencing

We extracted RNA from the same three individuals described above (Table S1 and Fig. S2). RNA was isolated using the HighPurity™ Total RNA Extraction Kit (Canvax, Valladolid, Spain). We selected a total of 35 samples (Table S2). RNA libraries were prepared with the VAHTS Universal V8 RNA-seq Library Prep Kit, being strand-specific and were sequenced on a NovaSeq 6000 (2 × 150 bp) aiming for an average of 40M read pairs per sample (Table S2), but we first sequenced the reference individual and later on the other two samples. Moreover, we sequenced one 8M SMRT HiFi cell on a Sequel II

PacBio machine containing two Iso-seq HiFi libraries at University of Leiden: one containing only the venom gland, and the second library being a pool of eight high-quality tissues (brain, kidney, liver, gallbladder, spleen, tongue, pancreas and ovary).

Genome assembly and scaffolding

Quality control of HiFi and Illumina reads was performed using FastQC v0.12.1 (Andrews, 2010) and adapters were removed with cutadapt v4.9 (Martin, 2011). In order to initially explore the genome size, heterozygosity levels and coverage data, we generated a k-mer profile with Meryl v1.4.1 (Rhie, Walenz, et al., 2020), using the raw HiFi reads and default parameters, and visualized it with GenomeScope2 v2.0.1 (Ranallo-Benavidez et al., 2020). Then, we assembled the genome following the VGP assembly pipeline v2.0 (Rhie et al., 2020). PacBio HiFi reads were assembled into contigs using the software Hifiasm v0.21.0 (Cheng et al., 2021), producing primary and alternate assemblies. We used *purge_dups* (Guan et al., 2020) to remove haplotypic duplicates from the primary assembly and added them to the alternate assembly. Then, we scaffolded the resulting haplotypic assembly using the Hi-C data with SALSA2 v1 (Ghurye et al., 2019), with default parameters. Following the VGP assembly pipeline (Rhie et al., 2020), manual curation was performed with Pretext v0.2.5 (https://pipelines.tol.sanger.ac.uk/curationpretext). Breaks were not mannualy created and we joint contings on gaps previously identified by SALSA2. We used the ~78x Illumina data to polish the assembly with one round of Pilon v1.24 (Walker et al., 2014). The mitochondrial genome was obtained with GetOrganelle v1.7.7.1 (Jin et al., 2020), using the available mitochondrial genome of several *Echis* species (*E. coloratus*, *E. carinatus* and *E. omanensis*) to seed the assembly (NCBI accession numbers: SRX18902082, SRX18902083, SRX18902084, respectively).

Genome assembly quality evaluation

Quality assessment and general metrics for the final assembly were estimated with both QUAST v.5.1.0 (Gurevich et al., 2013) and gfastats v1.3.8 (Formenti et al., 2022). Possible contaminations were evaluated with BlobToolKit v.4.4.0 (Challis et al., 2020) using the NCBI taxdump database. We also used MitoFinder v.1.4.2 (Allio et al., 2020; Li et al., 2016) to confirm that the mitochondrial genome

was absent in the assembled nuclear reference genome. Completeness of the genome assembly was assessed with BUSCO v5.3.0. against the sauropsida_odb10 database (*n*=7,480).

Genome annotation

First, we identified repetitive elements using RepeatModeler v.2.0.3 (Flynn et al., 2020) for *de novo* predictions of repeat families. To annotate genome-wide complex repeats, we used RepeatMasker v.4.1.3 (Tempel, 2012) with default settings to identify known Tetrapoda repeats present in the curated Repbase database (Bao et al., 2015). Then, we ran three iterative rounds of RepeatMasker to annotate the known and unknown elements identified by RepeatModeler in order to maximize the known elements at the expense of diminishing the unknown elements. Later, we soft-masked the genome for simple repeats. We used GeMoMa v.1.9 (Keilwagen et al., 2019) to annotate protein-coding genes, combining both the RNA-seq data generated in this study as described above (already mapped in to our new assembly) as well as annotations from seven other squamate genomes already published: *Anolis carolinensis* (Alföldi et al., 2011), *Crotalus adamanteus* (Hogan et al., 2024), *Crotalus tigris* (Margres et al., 2021a), *Ophiophagus hannah* (Vonk et al., 2013), *Naja naja* (Suryamohan et al., (2020), *Crotalus ruber* (Hirst et al., 2024) and *Crotalus viridis* (Schield et al., 2019). We quality checked and removed the adapters of the RNA-seq data using fastp v0.23.3 (Chen et al., 2018), as well as mapped the transcriptomic data to our new reference genome with Hisat2 v2.2.1 (Kim et al., 2019). Additionally, we also removed the adapters for the Iso-seq data with fastp v0.23.3 (Chen et al., 2018) and mapped the long-read transcriptomic data to our new reference genome with pbmm2, collapsing mapped reads into unique isoforms with isoseq3 and annotated with GeneMarkS-T v5.1 (Tang et al., 2015). We combined both annotations (GeMoMa and GeneMarkS-T) with TSEBRA (Gabriel et al., 2021). We BLASTp our predicted proteins to a Uniprot protein database for a total of ten species (*C. gasperettii*, *C. vipera*, *C. cerastes*, *Anolis carolinensis*, *Crotalus viridis*, *Crotalus tigris*, *Crotalus ruber*, *Crotalus adamanteus*, *Ophiophagus hannah* and *Naja naja*). Simultaneously, we ran Interproscan v5.72 (Jones et al., 2014) on our predicted proteins. Then, we combined both functional annotations with AGAT v1.4.1 (Dainat et al., 2023). Finally, as toxin-coding gene families are known to occur in large tandem arrays and the number of paralogs can be underestimated in particular gene families (Schield et al.,

2019), we performed additional annotation steps for toxin genes: Following Margres et al., (2021a), we used a combination of empirical annotation in FGENESH+ (Solovyev et al., 2006), as well as manual annotation using RNA-seq and Iso-seq alignments; the former identified all genes regardless of expression, whereas the latter was used to explicitly identify expressed toxins.

Chromosome-level analyses

Chromosomal synteny was explored between our new chromosome-level reference genome for the Arabian horned viper together with the Eastern diamondback rattlesnake (*Crotalus adamanteus*) (Hogan et al., 2024), the Indian cobra (*Naja naja*) (Suryamohan et al., 2020) and the Brown anole (*Anolis sagrei*) (Geneva et al., 2022) using Mcscan v1.4.23 (H. Tang et al., 2008). Protein sequences from each of the three venomous snakes were extracted using AGAT v1.2.1 (Dainat et al., 2023) and were pairwise aligned with LAST (Kiełbasa et al., 2011), implemented in the JCVI python module (Tang et al., 2017). A first alignment was used between the three species to identify chromosomes assembled in the reverse complement, which were corrected using SAMtools faidx v1.18.1 (Danecek et al., 2021) using both options reverse-complement and mark-strand. Gene annotations for the new reference (with the corresponding reversed chromosomes) were annotated using GeMoMa v.1.9 (Keilwagen et al., 2019), and MCscan was rerun. The last four scaffolds (14, 15, 16 and 17) from *Anolis sagrei* were removed, as no orthologous groups were found.

Transcriptomics

After adapter trimming and quality control using fastp v0.23.3 (Chen et al., 2018), we mapped our RNA-seq reads to the reference genome of *Cerastes gasperettii* using Hisat2 v2.2.1 (Kim et al., 2019). Gene expression raw counts per gene across all samples were calculated with StringTie (Pertea et al., 2015). Initial exploration of our transcriptomic data revealed a clear batch effect for one of the three samples (Fig. S4), due to the low mapping of that sample to our reference genome. Therefore, we decided to remove individual CG1 from future RNA-seq analyses. Moreover, to avoid pseudoreplication, we also removed the accessory gland from individual CG009 due to its high similarity with the venom gland, suggesting that the venom gland rather than the accessory gland was sampled (Fig. S4). Differential expression analyses were carried out with the DESeq2 package v.1.42.0

(Love et al., 2014) from R v4.4.2 (R Core Team, 2021).Prior to analysis, genes with fewer than 10 counts across all samples were filtered out. For comparisons, we defined two groups: venom glands versus all other tissues. DESeq2 employs a negative binomial generalized linear model to estimate differences in gene expression, and the p-values were adjusted for multiple testing using the Benjamini-Hochberg method to control the false discovery rate (FDR). Genes with an adjusted p-value < 0.01 and a fold change > 2 were considered significantly differentially expressed. Finally, we identified the highly expressed genes found in the venom gland as well as the toxins uniquely expressed in the venom gland (following Suryamohan et al., (2020)) which were defined as (1) genes expressed in the venom gland (TPM > 500), (2) Differential Upregulated Genes (DUGs) with Fold Change (FC) > 2 comparing venom glands to all other tissues and (3) unique to venom glands (TPM < 500 in all other tissues).

<u>Proteomics</u>

A bottom-up mass spectrometry strategy (Calvete et al., 2021) was used to characterize the venom of *Cerastes gasperettii*. Briefly, the venom proteome (pool from individuals CN6134 and CN6135, both from United Arab Emirates (UAE); Table S1) was submitted to reverse-phase High-performance liquid chromatography (HPLC) decomplexation followed by SDS-PAGE analysis in 12% polyacrylamide gels run under non-reducing and reducing conditions. Protein bands were excised from Coomassie Brilliant Blue-stained gels and subjected to automated in-gel reduction and alkylation on a Genomics Solution ProGest™ Protein Digestion Workstation. Tryptic digests were submitted to MS/MS analysis on a nano-Acquity UltraPerformance LC® (UPLC®) equipped with a BEH130 C_{18} (100µm x 100mm, 1.7 µm particle size) column in-line with a Waters SYNAPT G2 High Definition mass spectrometer. Doubly and triply charged ions were selected for CID-MS/MS. Fragmentation spectra were matched against a customized database including the bony vertebrates taxonomy dataset of the NCBI non-redundant database (release 258 of October 15, 2023) plus the species-specific venom gland transcriptomic and genomic protein sequences gathered in this work. Search parameters were as follows: enzyme trypsin (two-missed cleavage allowed); MS/MS mass tolerance for monoisotopic ions: ± 0.6 Da; carbamidomethyl cysteine and oxidation of methionine were selected as fixed and variable modifications, respectively. Assignments with significance protein score threshold of $p < 0.05$ (Mascot

Score > 43) were taken into consideration, and all associated peptide ion hits were manually validated. Unmatched MS/MS spectra were *de novo* sequenced and manually matched to homologous snake toxins available in the NCBI non-redundant protein sequences database using the default parameters of the BLASTP program (https://blast.ncbi.nlm.nih.gov/Blast.cgi).

Local synteny analyses To explore toxin genomic organization across (sub)families, we used BLASTn, incorporating both toxin and non-toxin paralogs to identify the genomic location of SVMPs, SVSPs and PLA$_2$ toxin families, across the genome of *Cerastes gasperettii*, *Crotalus adamanteus*, *N. naja* and *A. feae*. We excluded *A. feae* for SVSPs and SVMPs local synteny analyses as those families were not assembled onto a single contig in the *A. feae* genome. Then, we aligned those regions using Mafft (Katoh & Standley, 2013): For SVMPs in CHR8:16.506.135 to CHR8:17.374.029, for SVSPs in CHR9: 17.531.416 to CHR9:17.788.049 and for PLA$_2$ in CHR17:7.882.542 to CHR17:7.916.827 Each species was annotated within the MSA using its own annotation as a reference in Geneious Prime 2023.0.4. Results were plotted using the gggenomes package (https://github.com/thackl/gggenomes) from R v4.4.2 (R Core Team, 2021).

Toxin phylogenies

We used phylogenetic inference to study the evolutionary history for the main groups of toxins (i.e., SVMPs and SVSPs), which were the most abundant in the proteome of *Cerastes gasperettii*, as well as PLA$_2$ as this family has been widely studied within the Viperidae family (Dowell et al., 2016; Myers et al., 2022). For the three main toxin families, we selected available toxin genes as well as non-toxin paralogous genes from venomous species; we also included other non-toxin paralogous genes from non-toxic species (for details about this see Supplementary datasets for the three main toxins). When nuclear sequences were obtained, we translated CDS to protein sequence, and then protein sequences were aligned with Mafft v7 (Katoh & Standley, 2013). Following Giorgianni et al., (2020), we built a phylogeny for each of the toxin groups with the translated CDS sequences, as explained above, using Phyml v3.3 (Guindon et al., 2010), implementing the Dayhoff substitution model and validating our inferred tree with aBayes support.

Demographic history

We inferred the demographic history of *Cerastes gasperettii* by implementing the Pairwise Sequential Markovian Coalescent (PSMC v0.6.5) software (Li & Durbin, 2011) on the short-read whole-genome data. Heterozygous positions were obtained from bam files with Samtools v1.9 mpileup function (H. Li et al., 2009), and data were filtered for low mapping (<30) and base quality (<30). Minimum and maximum depths were set at a third (27x) and twice (156x) the average coverage. Only autosomal chromosomes were considered. We used the squamate mutation rate of $2.4x10^{-9}$ substitutions/site/generation and a generation time of 3 years, following Green et al., (2014) and Schield et al., (2022), respectively. A total of ten bootstraps were calculated, plotting the final results with the psmc_plot.pl function from PSMC (https://github.com/lh3/psmc).

Genomic diversity

We downloaded Illumina data for *Bothrops jararaca* (SRR13839751 from Almeida et al., (2021)), *Crotalus viridis* (SRR19221440 from Schield et al., (2019)), *Naja kaouthia* (SRR8224383; Thongchum et al., (2019)), *Naja naja* (SRR10428156; Suryamohan et al., (2020)) and *Sistrurus tergeminus* (SRR12802282; Bylsma et al., (2022)). Then, we filtered for quality (Phred score of 30) and removed adapters with fastp v0.23.3 (Chen et al., 2018). Trimming of poly-G/X tails and correction in overlapped regions were specified. All other parameters were set as default. Filtered sequences were visually explored with FastQC v0.12.1 (Andrews, 2010) to ensure data quality and absence of adapters. *Cerastes gasperettii* filtered reads were mapped against the new reference genome of *Cerastes gasperettii* using the bwa mem algorithm v0.7.17 (Li, 2013). *B. jararaca*, *C. viridis* and *S. tergeminus* were mapped against the *C. viridis* (Schield et al., 2019) reference genome and *N. naja* and *N. kaouthia* were mapped against the *N. naja* reference genome (Suryamohan et al., 2020). Mapped reads were sorted with Samtools v1.9 (H. Li et al., 2009) and duplicated reads were marked and removed with PicardTools v2.28.0 (Broad Institute, 2021). Reads with mapping quality lower than 30 were discarded. SNP calling was carried out with HaplotypeCaller from GATK v.4.1.3.0 (McKenna et al., 2010), with BP_resolution and split by chromosome. For each chromosome, individual genotypes were joined using CombineGVCFs with convert-to-base-pair-resolution, and the GenotypeGVCFs tool was then applied

to include non-variant sites. Finally, for each individual, the whole dataset split by chromosome was concatenated with bcftools concat (Danecek et al., 2021), keeping only the autosomes. Then, for each sample, we used the raw dataset to calculate average genome heterozygosity. We generated non-overlapping sliding windows for each of the reference genomes and included only sites (both variant and invariant) with site quality higher than 30 (QUAL field in a VCF file from GATK). Only windows containing more than 60,000 unfiltered sites were considered. Visualization was carried out with ggplot2 (Wickham, 2016) in R v4.4.2 (R Core Team, 2021).

Results and Discussion

Genome assembly and annotation

We generated a high-quality chromosome-level assembly for the Arabian horned viper (*Cerastes gasperettii*) by combining PacBio HiFi (65 Gbp of data), Hi-C (96 Gbp of data) and Illumina data (135 Gbp of data) (Fig. 1 and Fig. S3). First, we *de novo* assembled the HiFi reads into 1,018 contigs (N50=45.7 Mbp; longest contig of 149.99 Mbp). Then, using the proximity ligation data (i.e., Hi-C), we scaffolded the genome into 319 scaffolds (N50=111.38 Mbp; largest scaffold 345.38 Mbp). After manual curation, the scaffolding parameters of our genome were improved (N50=214.14 Mbp; largest scaffold 361.99 Mbp), containing 99.44% of the genome present in 19 scaffolds or pseudochromosomes (7 macro-, 10 micro-, Z and W sex chromosomes; Table 1 and Fig. 1B). The total genome length was 1.63 Gb, similar to other venomous snakes (Margres et al., 2021a; Schield et al., 2019; Suryamohan et al., 2020; Vonk et al., 2013; Table 1), with a contig N50 of 45.6 Mbp, ~3.3 times more contiguous than the *N. naja* genome (Suryamohan et al., 2020), ~228 times more contiguous than the *Anolis sagrei* genome (Geneva et al., 2022), but 0.67 times less contiguous than the recently published *Crotalus adamanteus* genome (Hogan et al., 2024), making it one of the most contiguous chromosomal squamate genomes assembled to date (Table 1). We assessed the completeness of the assembly using BUSCO (Simão et al., 2015) with the sauropsida gene set (*n*=7,480). Upon evaluation, we successfully identified 92.8% of the genes (91.4% single-copy, 1.4% duplicated), while the remaining genes were fragmented (1%) or missing (6.2%; Fig. 1). For the *de novo* assembly, GC content and repeat content were 37.87% and 43.63%, respectively. The repetitive landscape was dominated by retroelements (30.25%), with a

majority of LINEs (21.25%) (Table S3). Finally, we annotated 27,158 different protein-coding genes within our assembly, with a total of 194 putative toxins or toxin-paralogs genes. Toxin genes were found in both macro- and microchromosomes (Fig. 1), and were found onto individual contigs. Finally, we also found a battery of 3FTxs and myotoxin-like genes, but they were not represented in our proteome and RNA-seq dataset (see below).

<u>Genomic architecture highly conserved among vipers</u>

Whole-genome synteny comparisons showed similarity between *Cerastes gasperettii* and *Crotalus adamanteus*, with large syntenic blocks both within macro- and microchromosomes (Fig. 2). Some chromosomal rearrangements were observed between viperids and elapids, as previously discussed by Suryamohan et al., (2020), with a fission of chromosome four in *N. naja* to form chromosomes five and seven in vipers, and a fusion of chromosomes five and six in *N. naja* to form chromosome four in vipers. Interestingly, several chromosomal rearrangements between lizards and snakes have occurred, as we found several fission events in the *A. sagrei* genome, including one fission from chromosome two to originate the current Z chromosome in snakes (Fig. 2).

<u>Toxins uniquely expressed in the venom glands</u>

Our analyses of multi-tissue transcriptomic data (23 samples from two individuals covering 13 different tissues) reported a total of 23,178 expressed genes (TPM > 1). Heatmap of the 2,000 most variable genes reported unique upregulated genes for each of the analyzed tissues (Fig. S5). The venom gland transcriptome contained a total of 7,237 genes expressed (TPM > 500), including a total of 65 putative toxin genes. From those, we did not detect any 3FTxs and/or myotoxin-like gene transcripts. Differential gene expression analyses revealed a total of 161 genes (33 putative toxin genes) that were differentially upregulated (FC > 2 and 1% FDR) in venom glands compared to other tissues (Fig. 3A and Fig. S6-7). Finally, a total of 10 toxin genes (*CRISP2*, *SVMP9, SVMP10, SVSP8, SVSP7, SVSP5, CTL14*, *CTL15*, *SVSP4* and *SVMP13*) were uniquely expressed in the venom gland, encoding for the minimal core venom effector (Fig. 3A) (Suryamohan et al., 2020), and in line with the main toxins found within the proteome (Fig. 3B), although some differences were observed(as the absence of PLA_2 within the highly-expressed genes), possibly due to individual venom differences. These 10 genes,

together with other SVMPs, SVSPs and C-type lectins (CTL), were highly expressed in the venom gland and form the core toxic effector components of the venom. Targeting the core toxins together with other well-known modulators of venom may help manufacture of synthetic antivenom treatments as well as improve neutralization tests of current antivenoms (Suryamohan et al., 2020). However, more transcriptomic data should be incorporated to correct for potential ontogenetic and geographical variation in venom composition in *C. gasperettii* (Avella et al., 2022; Kalita et al., 2018).

SVSPs and SVMPs as main toxins

Venom proteomics identified Snake venom Serine Proteases (SVSPs) and Snake Venom Metalloproteinases (SVMPs) as the most abundant toxin families within the venom of *Cerastes gasperettii*, with 37.38% and 22.19% of the venom being composed by peptides from those two families, respectively (Fig. 3B); the dominance of these two toxin families is consistent with previous research on the same genus (Casewell et al., 2014; Fahmi et al., 2012). Other toxin families identified were DISI (12.74%), CTL (7.25%), PLA_2 (5.47%), Cysteine-Rich Secretory Proteins (CRISP; 4.34%) or L-Amino acid oxidase (LAAO; 1.71%) (Fig. 3B). We did not detect any 3FTx or myotoxin-like peptides within the proteome.

SVMPs

We analyzed the evolution of venom of the most abundant venom toxin groups (i.e., SVMPs and SVSPs, as well as PLA_2). After a thorough manual curation, we used comparative genomics to evaluate the number and position of those genes in comparison with the Indian cobra (*N. naja*), the Eastern diamondback rattlesnake (*Crotalus adamanteus*), and the Fea's viper (*A. feae*). We reported a total of 13 fully contiguous tandem array SVMPs for *Cerastes gasperettii* (Fig. 4A), next to the non-toxic paralogous gene *ADAM28* and flanked by the *NEFL* and *NEFM* non-toxic genes. Microsyntenic analyses showed gene copy number variation between the studied species (Fig. 4A). Overall, we can see an expansion in the number of SVMPs within the Viperidae family, particularly in *Crotalus adamanteus* (22 copies unique to vipers and 10 lineage-specific copies) but also in *Cerastes gasperettii* (12 copies unique to vipers and one lineage-specific copy) (Fig. 4A). Then, we reconstructed the evolutionary history of this toxin family (Fig. 4B and 8). Phylogenetic analyses for this toxin group

reported a highly supported clade comprising *ADAM28* peptides, the non-toxic paralogous gene. The second clade of orthologous toxin-peptides were found within both elapid and viperid families (including species from Crotalinae and Viperinae subfamilies in viperids; Fig. S8) as well as two SVMPs from *A. feae*. Interestingly, we report a new toxin-coding gene within *Cerastes gasperettii* with a different evolutionary history, as it did not share orthology with any other gene (Fig. 4B). This new gene likely arose from a duplication event of *SVMP13*, within the group of SVMP *MDC1* toxins (Fig. S8). Our discovery of a novel SVMP gene in *C. gasperettii* adds to the growing body of work on the dynamic evolution of venom systems. Similar gene expansions and duplications have been observed in other species, such as PLA_2 toxin-coding genes found in the venom of *Azemiops feae* (Myers et al., 2022), highligting the lineage-specific nature of venom evolution. The gene we identified, possibly arising from an *SVMP13* duplication, do not share orthology with genes in other species, suggesting the presence of hidden toxin diversity in venom systems. This discovery highlights the importance of using genomics in studying venom evolution, as this putatively toxic gene was not found to be differentially upregulated in the venom gland or recovered in the proteome (Fig. 3). More genomic data will indicate if *SVMP12* is unique for the Viperinae subfamily, the *Cerastes* genus or if it is only found in *Cerastes gasperettii*. All other clades were unique to viperids (and some exclusive only to crotalids), except for a clade composed by SVMPs unique to elapids, as previously discussed in Suryamohan et al., (2020). Interestingly, one of the toxins (*SVMP8*) was not a class P-III SVMP, as it clusters within the MAD-4/5 clade (class P-II SVMP), contrary to the proteomic results where all SVMPs were categorized within the class P-III (Fig. 3B). Although there has been a clear expansion of the SVMP family within the *Crotalus* genus, our results suggest that the origin of that expansion was at the beginning of the Viperidae family, as most of the groups are also present within the Viperinae subfamily.

<u>PLA_2</u>

Regarding PLA_2, we report two tandem repeat venom genes for *Cerastes gasperettii* within the non-toxic *PLA_2-g2E* and *PLA_2-g2F* array (Fig. 4C), flanked by *OTUD3* and *MUL1* non-toxic genes, as previously reported in other species (Dowell et al., 2016; Margres et al., 2021a; Myers et al., 2022). The number of venomous PLA_2 in *Cerastes gasperettii* was lower than in *A. feae* and *Crotalus*

adamanteus. Phylogenetic results for PLA_2 genes showed a fully supported clade containing both non-toxic *PLA_2-g2E* and *PLA_2-g2F* as outgroups (Fig. 4D and Fig. S9). We also found all other PLA_2 groups reported in previous studies: *PLA_2-gC, PLA_2-gK, PLA_2-gB, PLA_2-gD* and *PLA_2-gA* (Dowell et al., 2016; Myers et al., 2022). The two genes for our target species clustered in different groups (Fig. 4D and Fig. S9). The first PLA_2 was a *PLA_2-gD*, which is a group of PLA_2s exclusively found in true vipers (subfamily Viperinae). The second one was a *PLA_2-gC* which is more ancestral as it is also found in other pitvipers and non-venomous snakes such as pythons (Dowell et al., 2016). The genomic results are consistent with the proteomics, indicating that specific duplications of PLA_2 toxin-coding genes have not occurred in *Cerastes gasperettii*.

<u>SVSPs</u>

We found eight different SVSPs within the genome of *Cerastes gasperettii*, flanked by *RBM42* and *GRAMD1A* non-toxic genes (Fig. 4E). For this toxin family, we were only able to compare the results with *Crotalus adamanteus*. We were unable to confidently determine the location of SVSPs in the *N. naja* genome (several regions were matching our venomous SVSP genes as well as the flanking genes). Moreover, *A. feae* was also not compared as SVSPs were not assembled in a single contig.. Phylogenetic results showed three clades, with two containing *Cerastes gasperettii* genes (Fig. 4F and Fig. S10). Group 1 was mainly present within *Crotalus*, although there was the presence of some true vipers species, but not in *Cerastes gasperettii* (Fig. S10). Group 2 contained six genes within *Crotalus adamanteus* and only two for *Cerastes gasperettii*. Interestingly, Group 3 was expanded in *Cerastes gasperettii* (Fig. 4E) with a total of six copies, while four were found within *Crotalus adamanteus*. Most of the toxins included in the analyses for true vipers were also found in Group 3 (Fig. S10), indicating a possible expansion of this group of toxins in true vipers (or gene losses in pit vipers). Overall, our high-quality chromosome level reference genome has shed light on the evolution of the main toxin-coding gene families, indicating a compelling correlation between the abundance of toxin-coding genes and the prevalence of these toxins in the venom of *Cerastes gasperettii*.

<u>Glacial periods drove population expasions in *C. gasperettii*</u>

The Arabian horned viper (*C. gasperettii*) is a widespread species, categorized as Least Concern by the IUCN (Egan et al., 2012). Genome-wide diversity was in line with its conservation status, as it showed similar heterozygosity levels compared to other venomous snakes (Fig. 5A). However, more individuals should be sampled along its distribution to verify that similar heterozygosity levels are found across its range. PSMC analyses showed several population expansions and contractions in the last 400 kya, whilst the effective population size of *Cerastes gasperettii* remained relatively constant from 1 until 10 Mya (Fig. 5B). Interestingly, population expansions were coincident with the Last glacial and Penultimate glacial periods (grey lines on Fig. 5B), with a large population increase during the Penultimate Glacial Period (PGP) (1.94 to 1.35 mya) (Fig. 5B). In fact, during glacial periods, global sea level dropped around 150 meters, exposing the floor and the sand to the wind, which promoted aridification in the Arabian Peninsula and potentially increased habitat suitability for the species (Burriel-Carranza et al., 2023; Glennie & Singhvi, 2002). PSMC results may vary depending on the generation time as well as the mutational rate specified. The absence of species-specific data for this analyses may bias our results, although it is a general consensus in the literature when inferring these demographyc analyses in snakes (e.g. Schield et al., 2019).

Conclusions

Our high-quality chromosome-level reference genome for *C. gasperettii* showed that chromosomal architecture is highly conserved between Crotalinae and Viperinae subfamilies, and differs from elapid genomes by a small number of chromosomal rearrangements. We also found the genomic coordinates of the main toxin-encoding genes, highlighting gene duplication as the main driver in the evolution of SVMP and SVSP toxins. We identified a new SVMP toxin-coding gene, showcasing the importance of using high-quality reference genomes (combined with other -omic techniques) for thoroughly characterizing toxin-encoding genes. Finally, this is a new and important resource for a large clade with few reference genomes available. Future genomic studies focusing on Old World viper evolution will benefit greatly from this resource, which will help unveil the origin and diversification of venom and serve as an essential genomic tool for further venomic studies on the subfamily Viperinae.

Acknowledgements

GM-R is supported by an FPI grant from the Ministerio de Ciencia, Innovación y Universidades, Spain (PRE2019-088729), SRH is awarded by the National Science Foundation Graduate Research Fellowship Program with grant no. 2136515, AT is supported by “la Caixa” doctoral fellowship program (LCF/BQ/DR20/11790007), BB-C is supported by FPU grant from Ministerio de Ciencia, Innovación y Universidades, Spain (FPU18/04742) and ME is supported by an FPI grant from Ministerio de Ciencia e Innovación (PRE2022-101473). In the UAE, we wish to thank His Highness Sheikh Dr. Sultan bin Mohammed Al Qasimi, Supreme Council Member and Ruler of Sharjah, H. E. Ms. Hana Saif al Suwaidi (Chairperson of the Environment and Protected Areas Authority, Sharjah) for their continuous support. Some of this research was carried out on the High Performance Computing resources at New York University Abu Dhabi. We thank Jonathan Wood and Klara Eleftheriadi for their input during the genome assembly and manual curation processes. We also thank Valéria Marques for her help in building the figures and Prem Aguilar for reviewing a previous version of the manuscript.

Data availability

Final assembly and raw reads files were deposited in NCBI under bioproject No. PRJNA1068073.

Funding

This work was funded by grant PID2021-128901NB-I00 (MCIN/AEI/10.13039/501100011033 and by ERDF, A way of making Europe; Spain) and grant 2021-SGR-00751 from the Departament de Recerca i Universitats from the Generalitat de Catalunya, Spain to SC.

Competing Interests

The authors declare that they have no competing interests.

Author’s contribution

Conceptualization: G.M.R., A.T., B.B.C., J.C., J.E., M.M., S.C. Investigation: S.H, V.P., M.E., T.B., S.B., M.H., J.T.G., D.P., J.C., M.M. Funding acquisition: S.C. Writing-original

draft: G.M.R. Writing-review & editing: All authors read, revised, and approved the manuscript final version.

Ethics statement

No in vivo experiments were performed. Specimens were collected and manipulated with the authorization and under strict control and permission of the government of the United Arab Emirates (Environment and Protected Areas Authority, Government of Sharjah), who approved the study. Specimens were captured and processed following the guidelines and protocols stated in the agreements obtained from the competent authority of the United Arab Emirates. Members of the government supervised collecting activities. All efforts were made to minimize animal suffering. All the research in the United Arab Emirates was done under the supervision and permission of the Environment and Protected Areas Authority, Government of Sharjah.

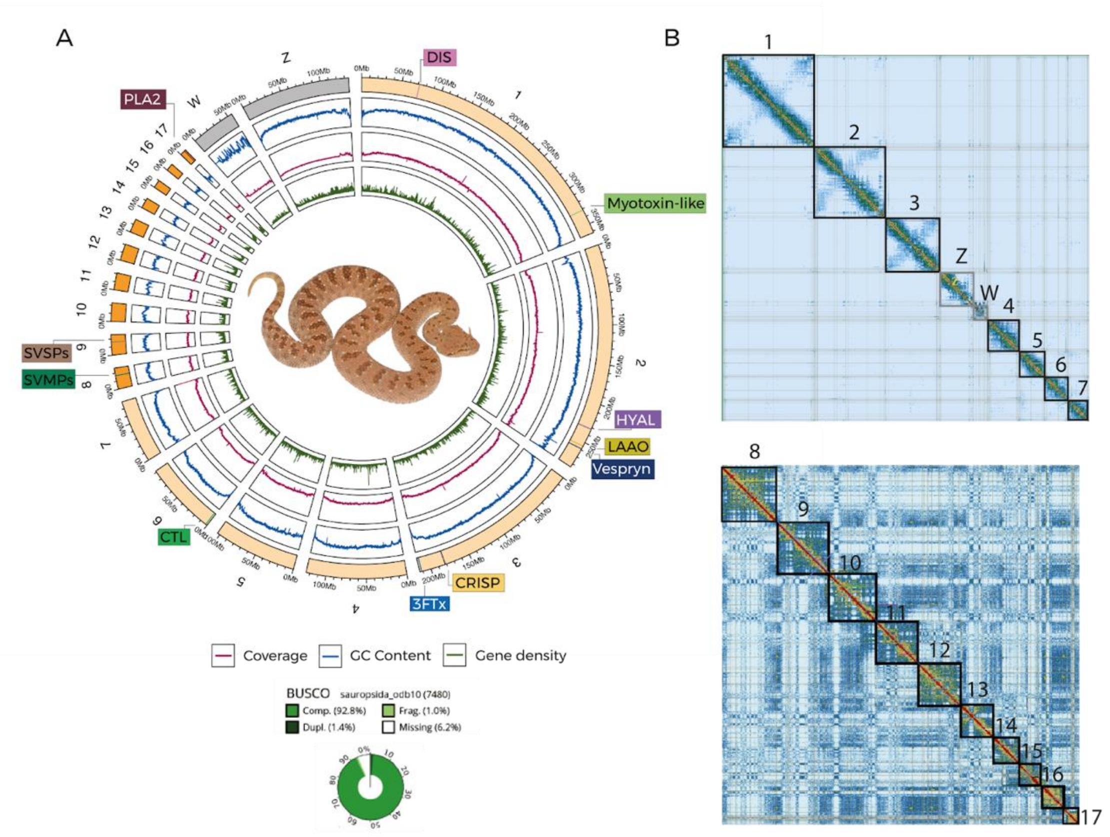

Fig. 1: A) Reference genome for *Cerastes gasperettii*, including BUSCO score, GC content, coverage level as well as the main toxins found within the genome. Macrochromosomes are shown in light orange whilst microchromosomes are shown in bright orange. Sex chromosomes are shown in gray. Abreviations are as follows: DIS, Disintegrins; HYAL, Hyaluronidases; LAAO, L-Amino acid oxidase; CRISP, Cysteine-rich secreted proteins; 3FTx, Three-finger toxins; CTL, C-type lectins; SVMPs, Snake venom metalloproteinases; SVSPs, Snake venom serine proteinases; PLA_2, Phospholipases. B) HiC contact map for the macrochromosomes (above), including the sex chromosomes (Z and W), and microchromosomes (below).

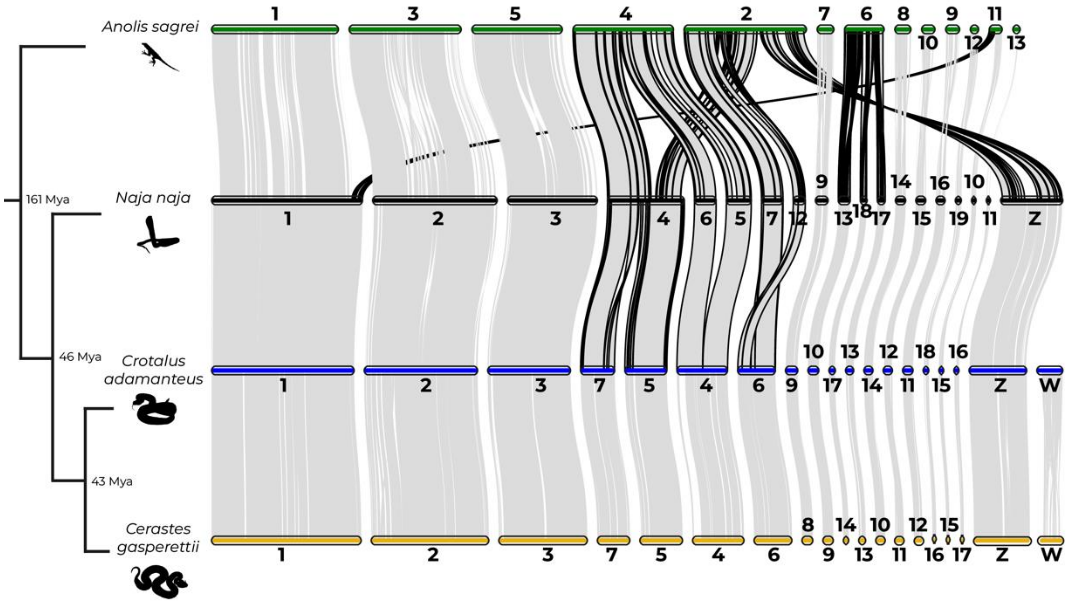

Fig. 2: Chromosome-level analyses for one Elapidae (*Naja naja*), one Crotalinae (*Crotalus adamanteus*) and one Viperinae (*Cerastes gasperettii*) species, with *Anolis sagrei* as the outgroup. The four smallest scaffolds (14, 15, 16 and 17) of *Anolis sagrei* were removed, as no orthologous groups were found with other species. Borders of regions showing evidence for chromosomal rearrangements are shown in black. Estimates for branch times obtained from TimeTree.org based on divergence times between Iguania and Serpentes, Elapidae and Viperidae and Crotalinae and Viperinae, respectively.

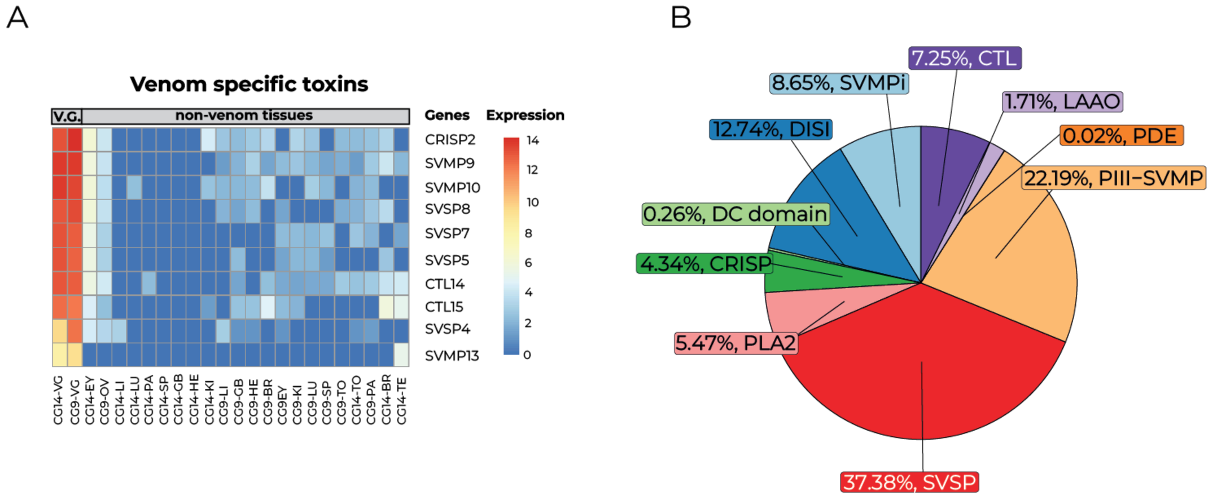

Fig. 3: Main toxins found in both the transcriptome and proteome of *Cerastes gasperettii*. A) Transcriptomic results with genes upregulated and exclusively found in the venom gland for both individuals. Each column represents a different tissue type per sample. Rows show the different genes, and colors correspond to different expression levels. Abbreviations are as follows: VG, Venom Gland; EY, Eye; OV, Ovary; LI, Liver; LU, Lung; PA, Pancreas; SP, Spleen; GB, Gallbladder; HE, Heart; KI, Kidney; LI, Liver; BR, Brain; TO, Tongue; TE, Testis. B) Proteomic results of venom composition for a pool of two individuals of *Cerastes gasperettii*. The pie chart displays the relative abundances of the toxin families found in the proteome of the *Cerastes gasperettii* venom. Abbreviations are as follows: SVMP, snake venom metalloproteinase; SVSP, snake venom serine proteases; PLA_2, phospholipases A_2; CRISP, cysteine-rich secretory proteins; DISI, disintegrins; CTL, C-type lectins; LAAO, L-amino-acid oxidases; PDE, phosphodiesterases.

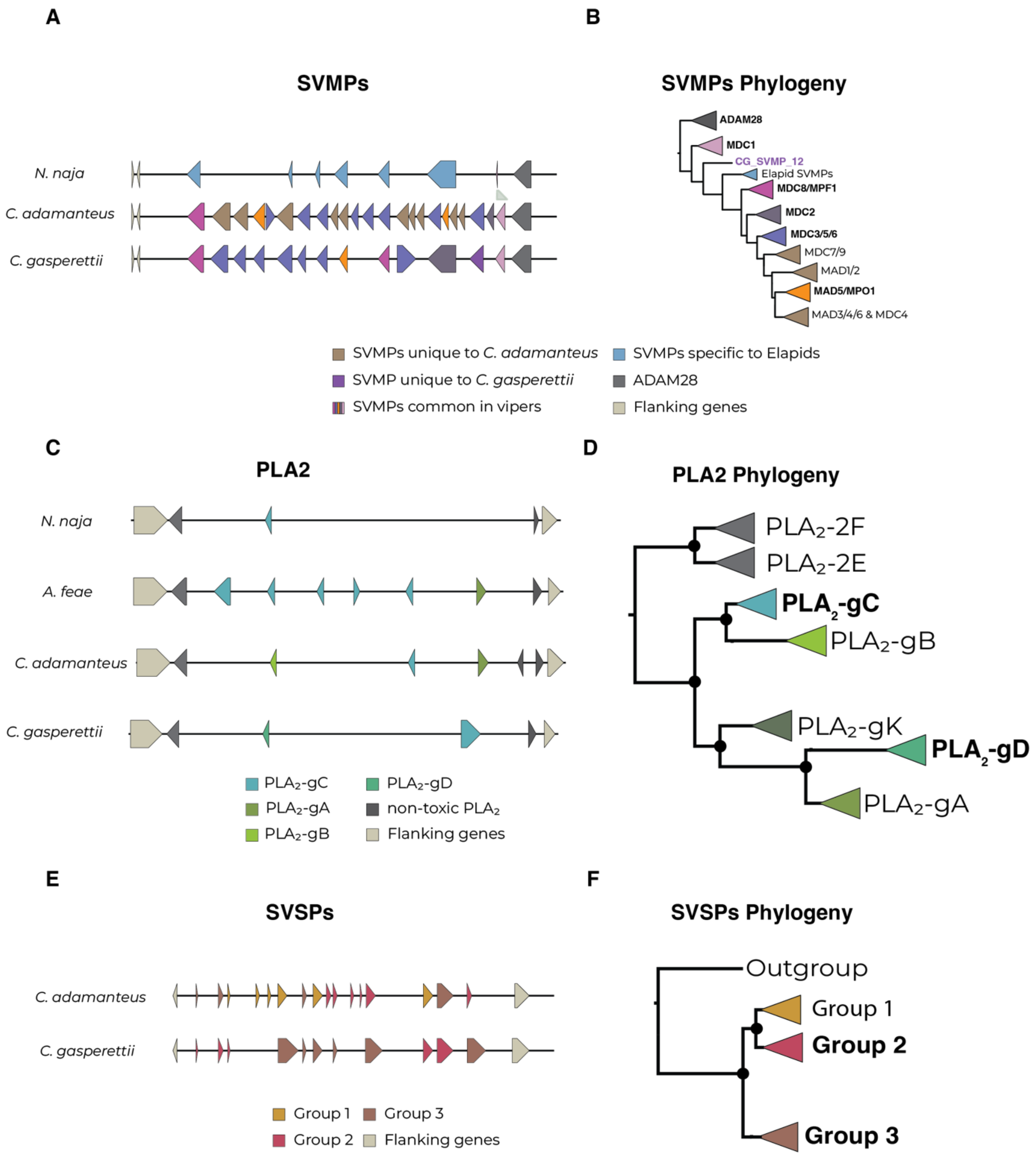

Fig. 4: A) Local synteny analyses for the SVMP toxin family in *Naja naja*, *Crotalus adamanteus* and *Cerastes gasperettii*. Different colors indicate orthologous genes unique to *C. gasperettii*, crotalids, true vipers or elapids. ADAM28 (right) as well as flanking genes (left) are also indicated. B) Phylogeny of SVMPs, in bold, groups that contained SVMPs from *Cerastes gasperettii*. In purple is indicated the gene exclusively found in *Cerastes gasperettii*. C) Local synteny analyses for PLA_2 in *Naja naja*, *Azemiops feae*, *Crotalus adamanteus* and *Cerastes gasperettii*. Non-toxic PLA_2 and flanking genes are also shown. D) Phylogeny of the PLA_2 gene family, with two non-toxic PLA_2 as outgroups. Some samples that did not fit in any category have been removed. For a complete phylogeny see Fig. S9. Note that PLA_2-gK is present in the phylogeny but not in the local synteny analyses, as any of the studied species contains it. E) Local synteny analyses for SVSPs for *Crotalus adamanteus* and *Cerastes gasperettii*. Flanking genes are also shown. F) Phylogeny for SVSPs with a non-toxic outgroup. For the three different phylogenies the groups that contained toxins from *Cerastes gasperettii* are highlighted in bold.

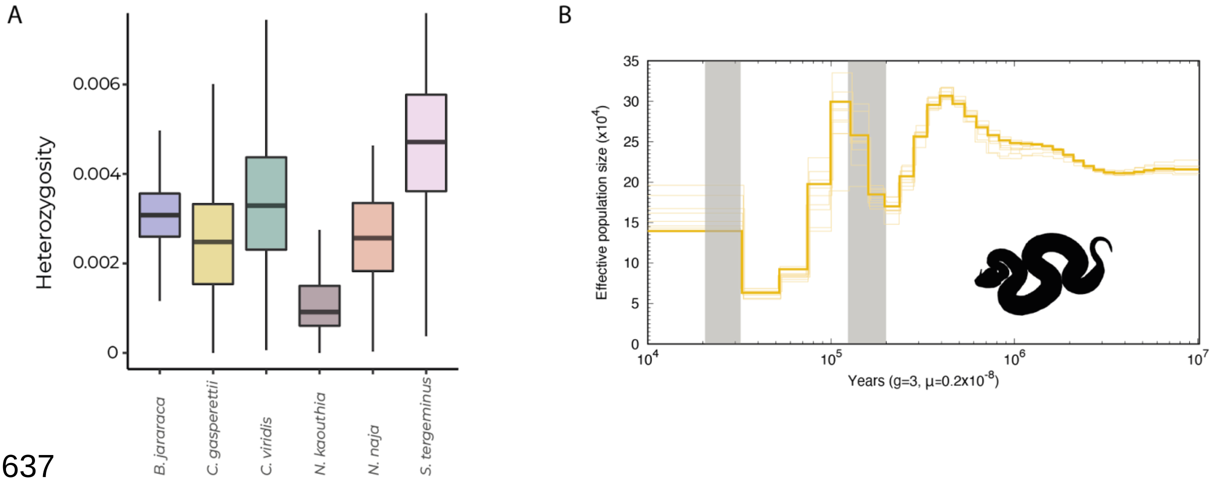

Fig. 5: A) Genome-wide diversity for a total of six different venomous snakes: *Bothrops jararaca*, *Cerastes gasperettii*, *Crotalus viridis*, *Naja kaouthia*, *Naja naja* and *Sistrurus tergeminus*. B) PSMC analysis recovering the ancient demographic history of *Cerastes gasperettii*. Generation time was set to 3 years and the substitution rate to $2.4x10^{-9}$ per site per year. Shaded lines represent 10 bootstrap estimates. Two last glacial periods are shown with grey lines.

Table 1: Comparison of our new reference genome for *Cerastes gasperettii* with other high-quality squamate genomes. Best value per category is shown in bold.

	Cerastes gasperettii	*Crotalus adamanteus*	*Naja naja*	*Anolis sagrei*
Genome size	1.63 Gbp	1.69 Gbp	1.79 Gbp	1.92 Gbp
Number of scaffolds	221	**27**	1,897	3,738
Scaffold N50	214.14 Mbp	208.9 Mbp	223.35 Mbp	**253.58** Mbp
Scaffold L50	**3**	**3**	**3**	4
Contig N50	45.6 Mbp	**67.5** Mbp	13.06 Mbp	0.2 Mbp

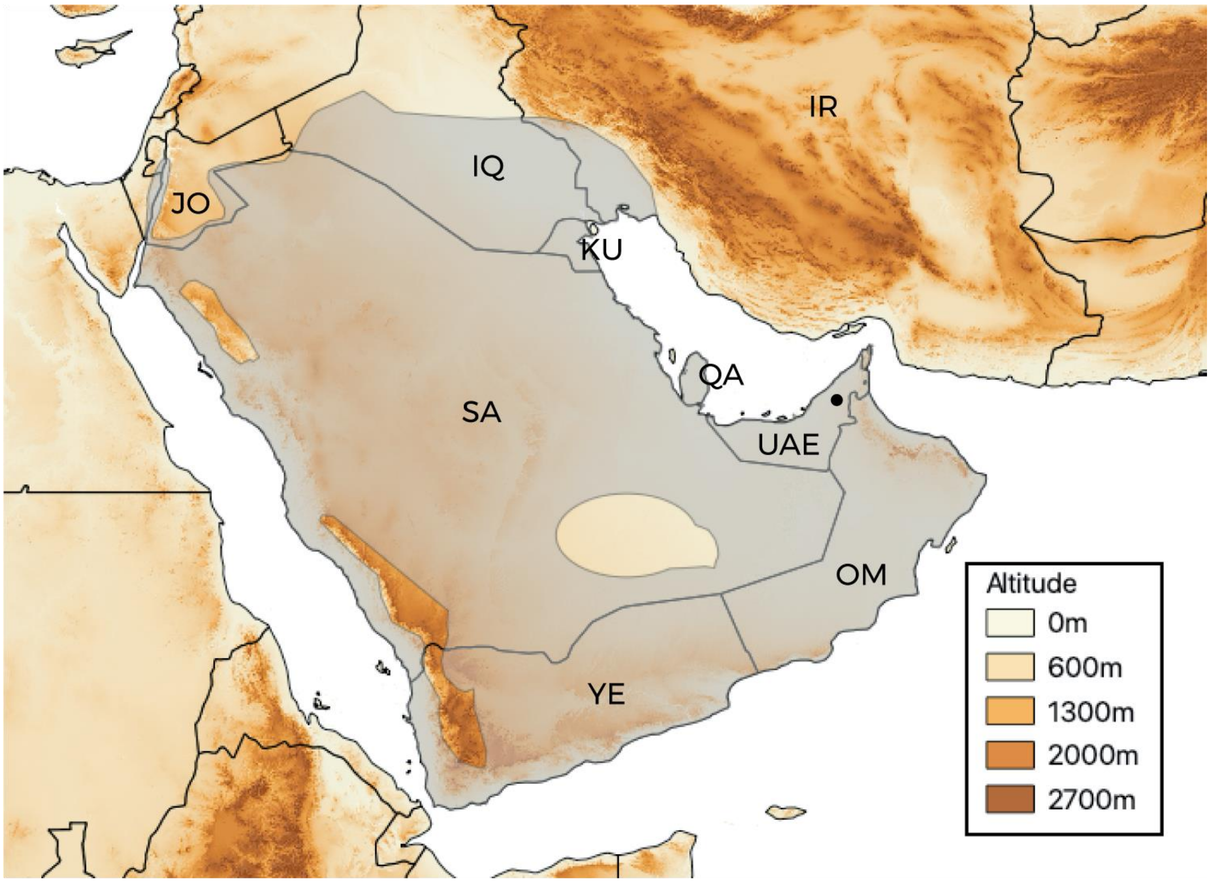

Fig. S1: Distribution map for the studied species *Cerastes gasperettii* with the location of our samples. Countries where the species is present are indicated. Abbreviations are as follows: JO, Jordania; SA, Saudi Arabia; YE, Yemen; OM, Oman; UAE, United Arab Emirates; IQ, Iraq; IR, Iran; KU, Kuwait, QA, Qatar.

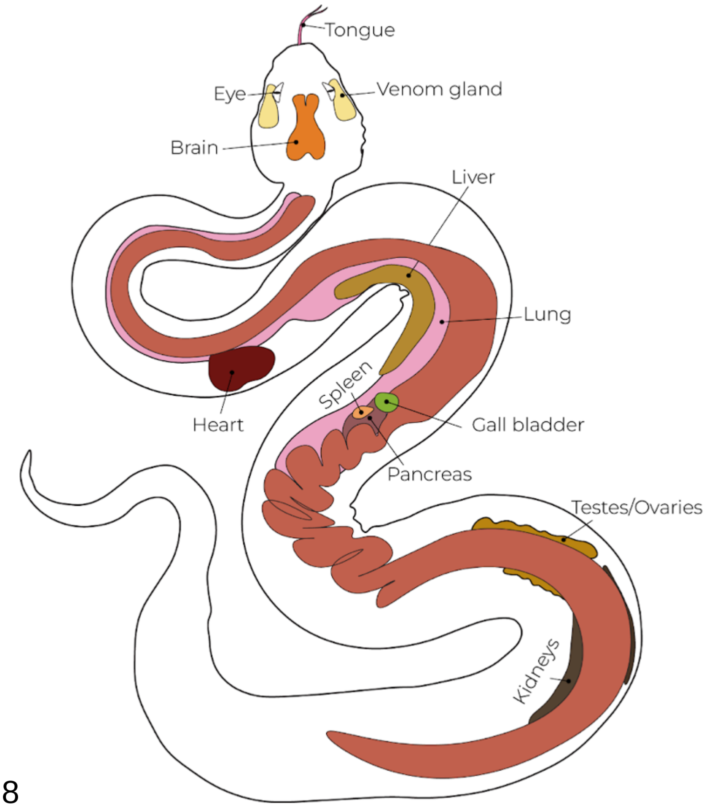

Fig. S2: Drawing of an Arabian horned viper depicting all the tissues sampled for RNA-seq analyses.

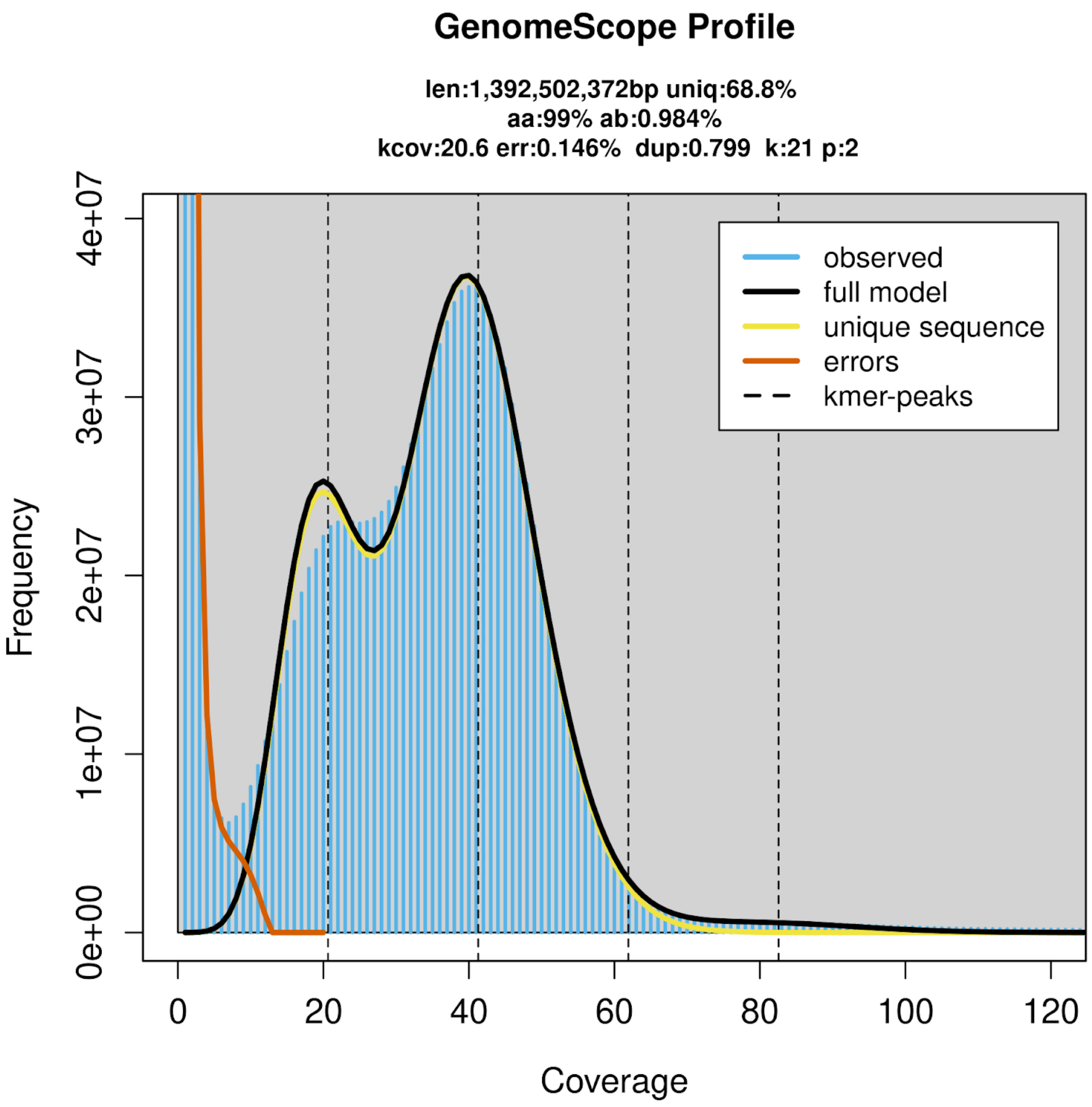

Fig. S3: Histogram from GenomeScope showing the frequency of reads in relation with their coverage.

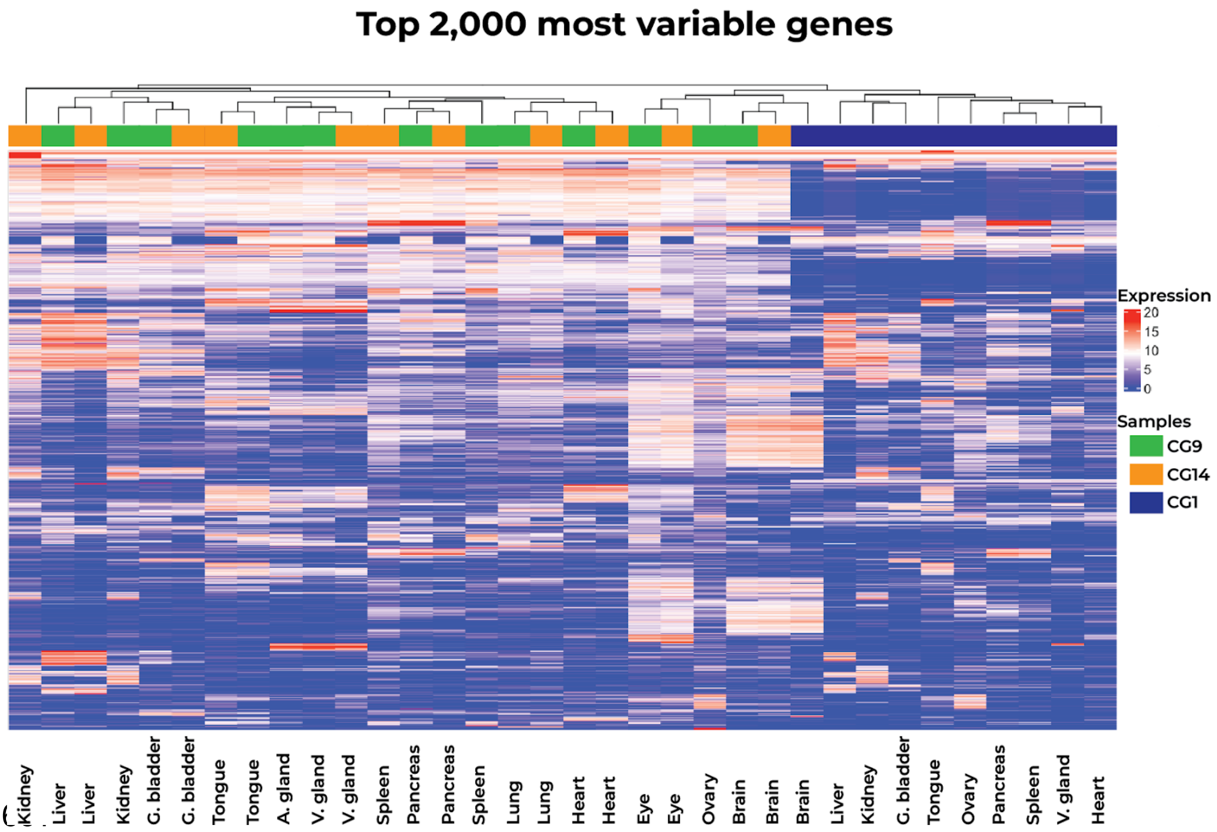

Fig. S4: Heatmap for the 2,000 most variable genes within our three samples, showing a clear batch effect of sample CG1 (possibly due to differences in sequencing time) as well as a high similarity between the putative accessory gland and the venom gland. Each column represents a different sampled tissue. The three different samples are depicted with different colors at the top of the heatmap. Abbreviations are as follows: G. bladder, gallbladder and V. gland, venom gland.

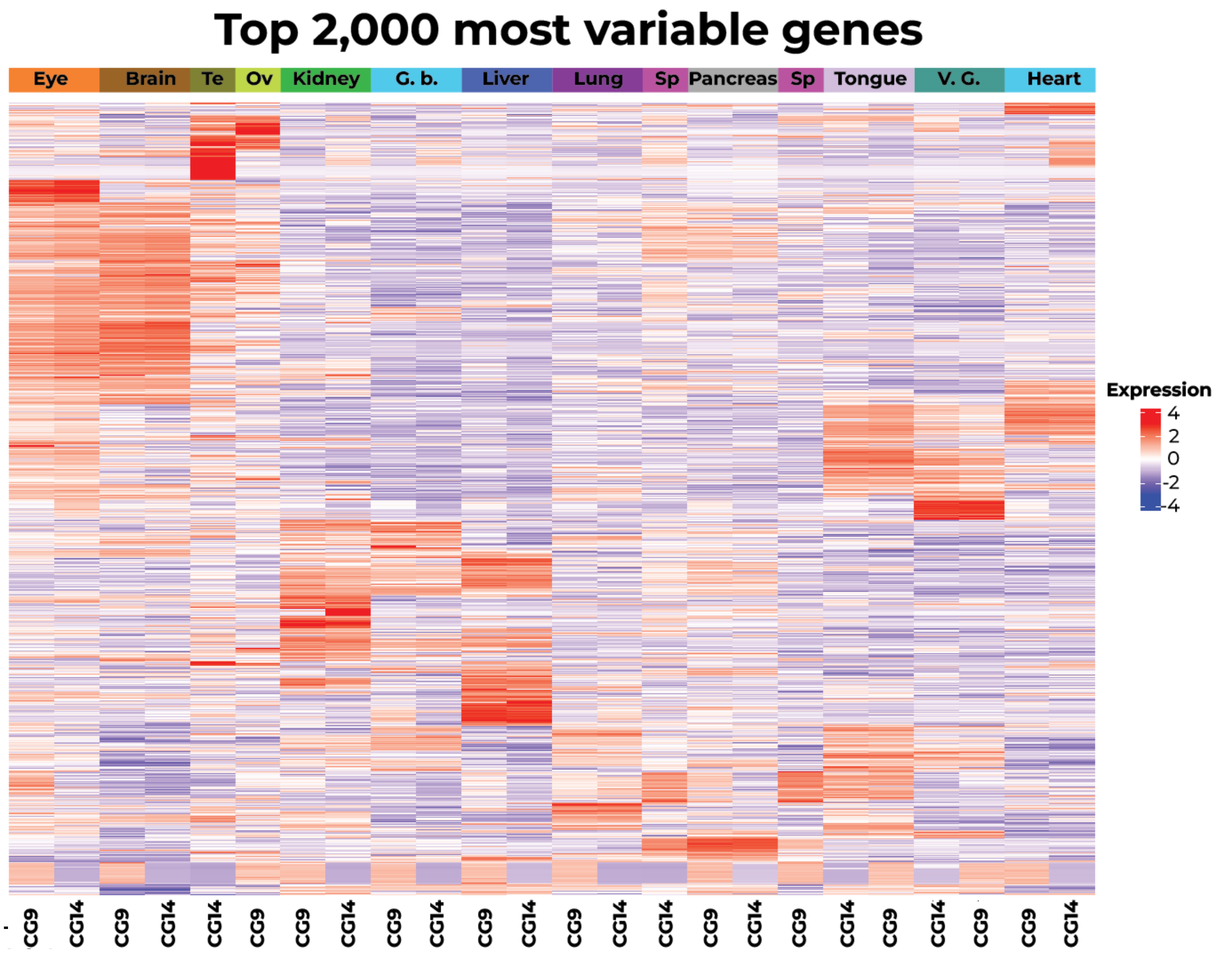

Fig. S5: Heatmap for the 2,000 most variable genes for both samples, reporting highly expressed genes unique for each tissue type. Each column represents one tissue sampled per individual. Expression levels were normalized. Abbreviations are as follows: Te, Testis; Ov, Ovary; G.b., gallbladder; Sp, Spleen and V.G., Venom gland.

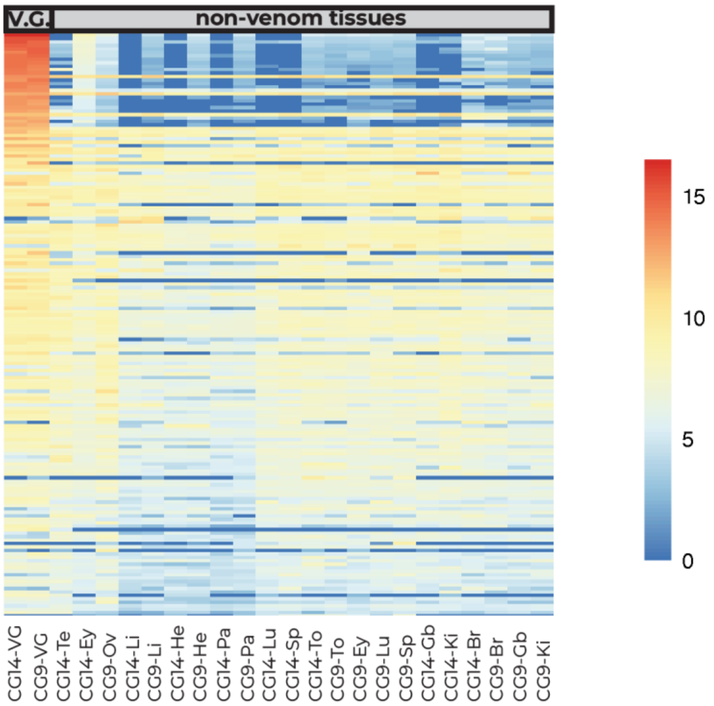

Fig. S6: Heatmap for the 161 upregulated genes found in the venom gland of *C. gasperettii* transcriptome including the 65 putative expressed toxins for both venom gland samples. Each column represents one tissue sampled per individual. Abbreviations are as follows: VG, Venom Gland; Ki, Kidney; GB, Gall Bladder; Lu; Lung; Sp, Spleen; He, Heart; Li, Liver; Pa, Pancreas; To, Tongue; Te, Testis; Ov, Ovary.

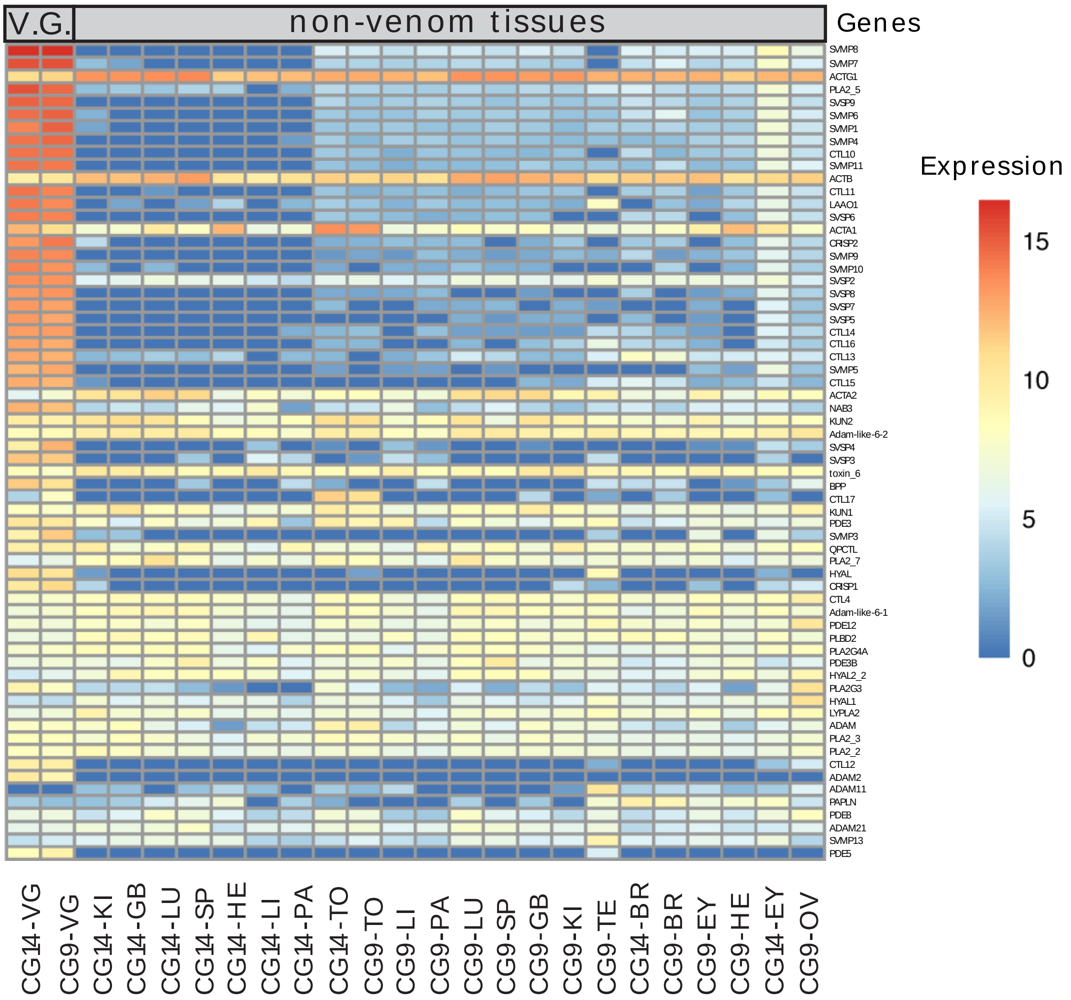

Fig. S7: Heatmap for the venom gland transcriptome for the 65 putative expressed toxins for both venom gland samples. Each column represents one tissue sampled per individual. Abbreviations are as follows: VG, Venom Gland; Ki, Kidney; GB, Gall Bladder; Lu; Lung; Sp, Spleen; He, Heart; Li, Liver; Pa, Pancreas; To, Tongue; Te, Testis; Ov, Ovary.

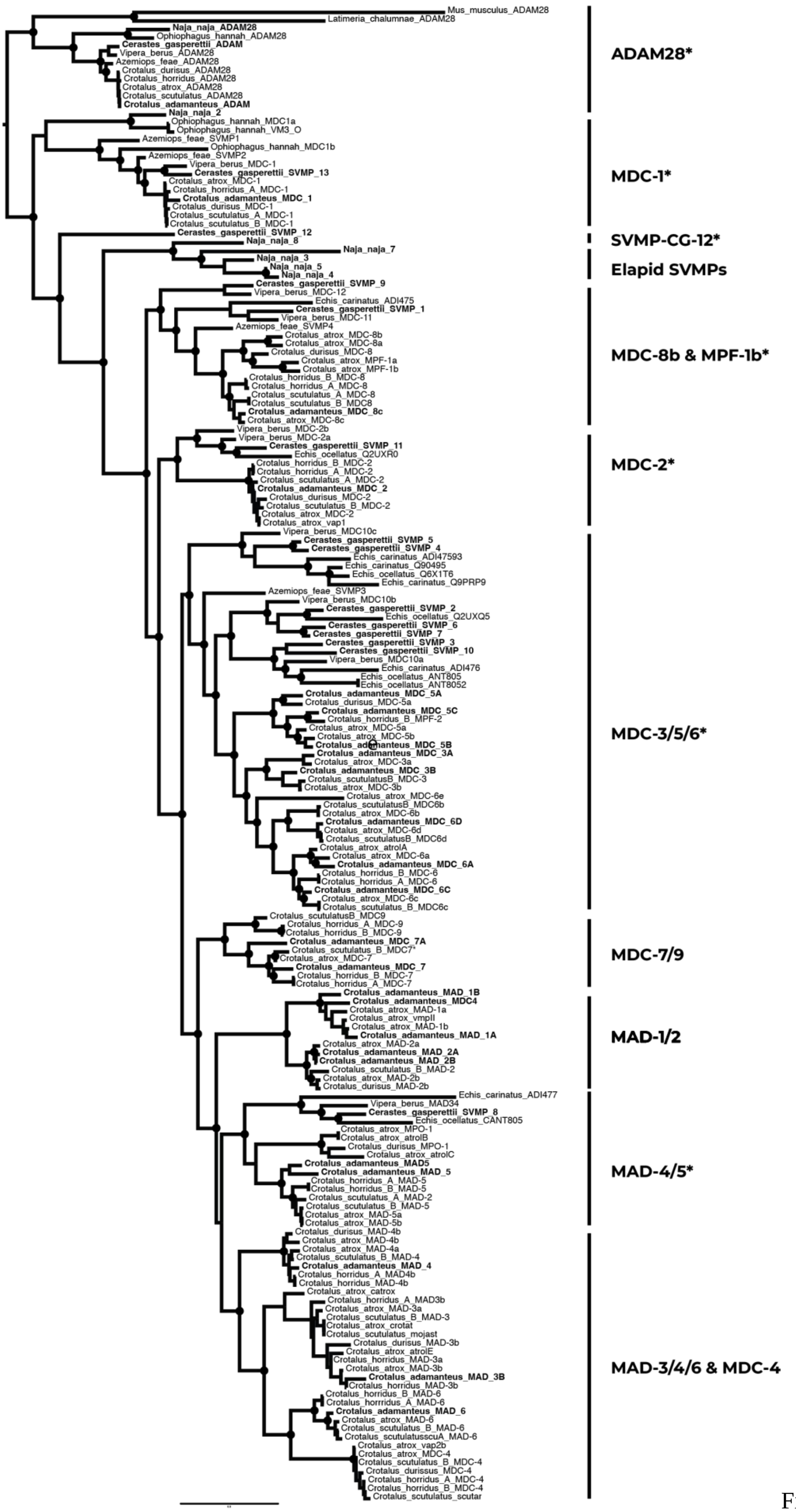

727

Fig. S8: Maximum

likelihood phylogeny for SVMP genes and its non-toxic paralog (ADAM28). Genes for *Cerastes gasperettii* are highlighted in bold. Toxin groups are identified following previous categorizations. Asterisks indicate if *Cerastes gasperettii* genes are present in that specific group. Branch support with aBayes values higher than 90 are depicted as circles.

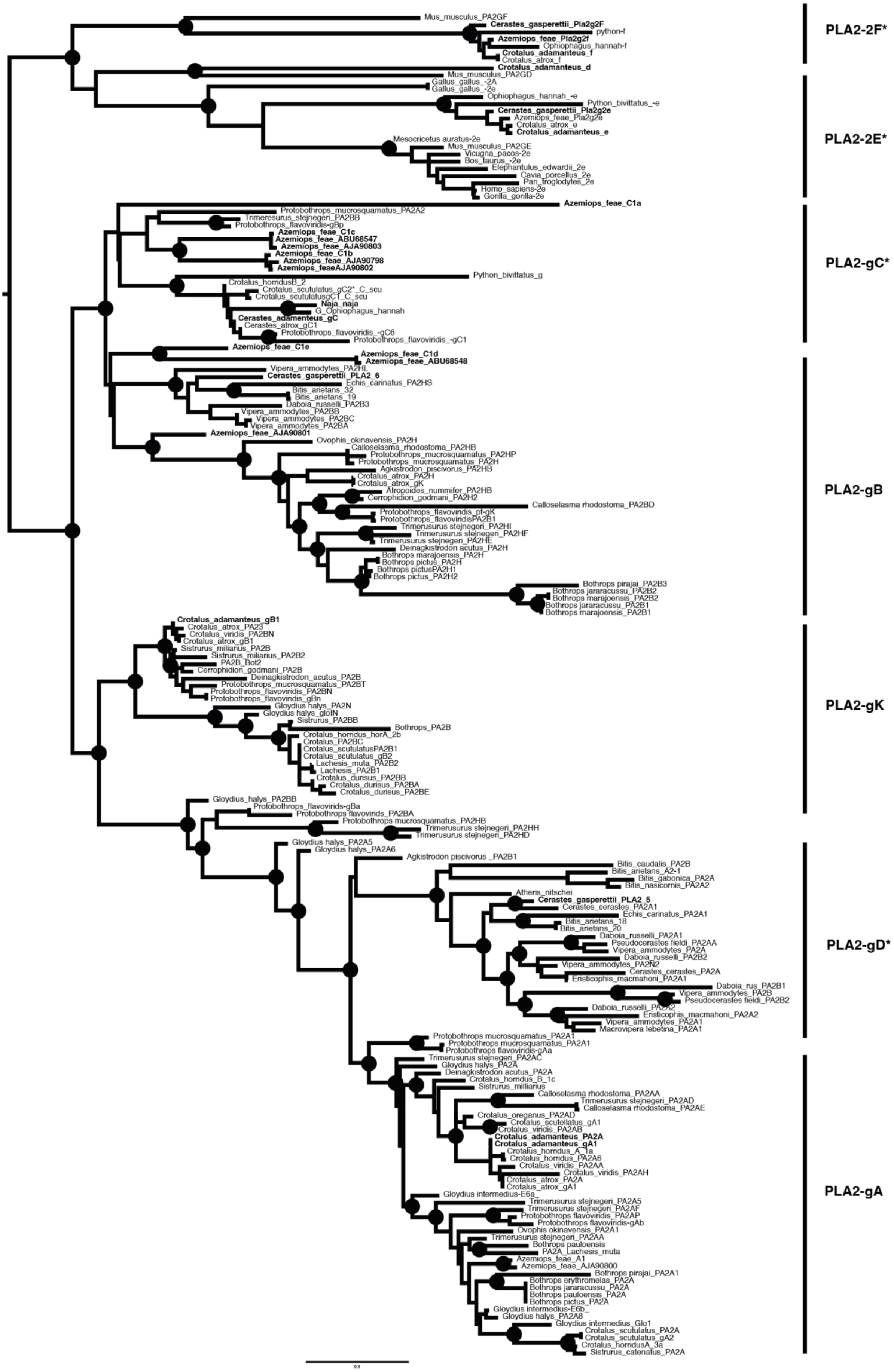

Fig. S9: Maximum likelihood phylogeny for PLA_2, with the two non-toxic genes as outgroups (PLA_2-

2F and PLA$_2$-2E). Asterisks in group labels indicate if *Cerastes gasperettii* genes are present in that specific group. Branch support with aBayes values higher than 90 are depicted as circles.

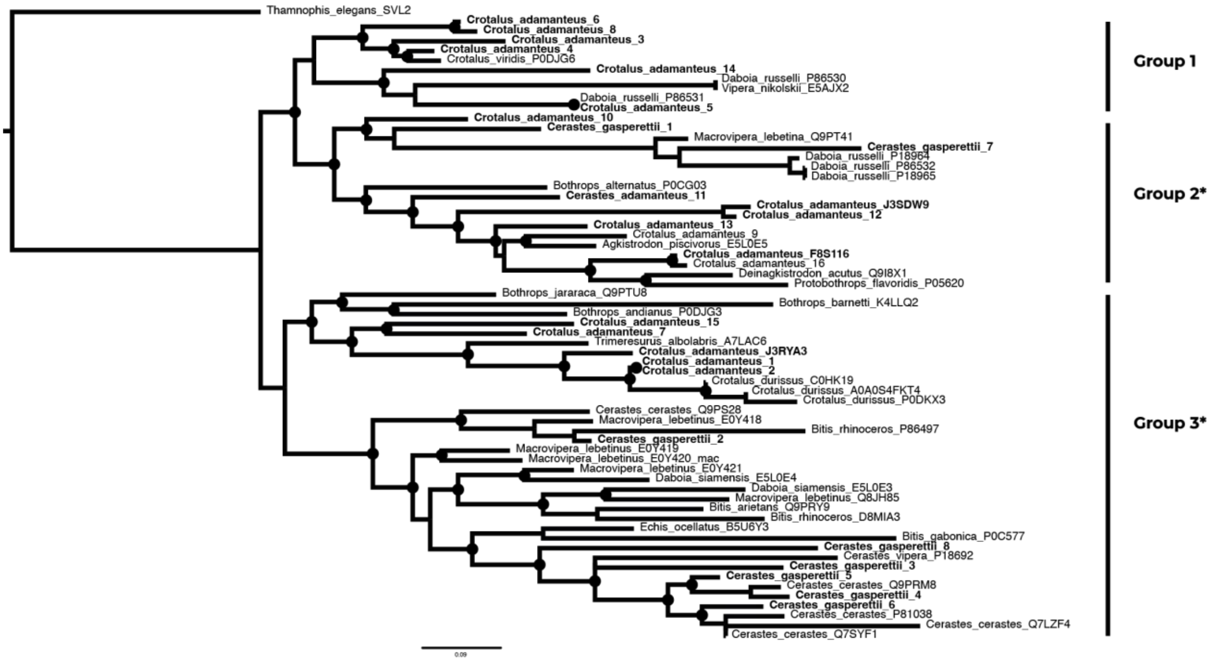

Fig. S10: Maximum likelihood phylogeny for SVSPs, with one sample from *Thamnophis elegans* as outgroup. Asterisks in group labels indicate if *Cerastes gasperettii* genes are present in that specific group. Branch support with aBayes values higher than 90 are depicted as circles.

Table S1: Individuals sampled in this study with their sex, sampling coordinates and data sequenced.

ID	Sex	Latitude	Longitude	Data sequenced
CG1	Female	25.284690	55.687860	HiFi, Omni-C, Illumina, RNA-seq, Iso-seq, Proteome
CG9	Female	25.284690	55.687860	RNA-seq
CG14	Male	25.284690	55.687860	RNA-seq

CN6134	-	UAE	UAE	Proteome
CN6135	-	UAE	UAE	Proteome

Table S2: Id, tissue type and number of reads sequenced per sample.

**ID**	**Tissue**	**Reads**
CG9	Tongue	44,672,733
CG9	Venom gland	41,124,132
CG9	Eye	41,951,109
CG9	Brain	42,800,966
CG9	Heart	40,715,947
CG9	Lung	42,518,938
CG9	Liver	42,251,137
CG9	Gallbladder	42,738,665
CG9	Spleen	40,909,550
CG9	Pancreas	40,527,010
CG9	Ovary	41,118,336
CG9	Kidney	40,620,023
CG9	Accessory gland	44,114,293
CG14	Tongue	41,455,346
CG14	Venom gland	41,035,764
CG14	Eye	40,753,220
CG14	Brain	43,413,973
CG14	Heart	42,338,980
CG14	Lung	42,068,410
CG14	Liver	21,549,210
CG14	Gallglabbder	50,571,941

CG14	Spleen	45,447,235
CG14	Pancreas	50,223,941
CG14	Testis	47,495,900
CG14	Kidney	45,945,776
CG1	Heart	47,362,067
CG1	Brain	45,740,571
CG1	Kidney	50,758,869
CG1	Gallbladder	40,546,711
CG1	Liver	48,058,958
CG1	Spleen	44,752,981
CG1	Tongue	46,837,490
CG1	Pancreas	45,023,783
CG1	Venom gland	49,775,424
CG1	Ovary	48,703,420

Table S3: Different types of repetitive elements masked within the genome:

Element	Number of elements	Length (bp)	Percentage
Retroelements	1524124	493932584	30.25 %
SINEs:	339152	55265721	3.38
Penelope	124778	19471740	1.19
LINEs:	988815	347028895	21.25
CRE/SLACS	0	0	0.00%
L2/CR1/Rex	480371	137654000	8.43
R1/LOA/Jockey	579	99034	0.01
R2/R4/NeSL	41793	10873028	0.67

RTE/Bov-B	128092	79663597	4.88
L1/CIN4	207974	95913575	5.87
LTR elements:	196157	91637968	5.61
BEL/Pao	16545	5263265	0.32
Ty1/Copia	25582	15088781	0.92
Gypsy/DIRS1	102598	63604234	3.90
Retroviral	50617	7642063	0.47
DNA transposons	707499	111444059	6.83
hobo-Activator	265944	30679712	1.88
Tc1-IS630-Pogo	227637	58877559	3.61
En-Spm	0	0	0.00%
MULE-MuDR	44	3962	0.00%
PiggyBac	138	6619	0.00%
Tourist/Harbinger	182161	18395721	1.13
Other	0	0	0.00%
Rolling-circles	2242	136656	0.01
Unclassified	205700	42385187	2.60
Total interspersed repeats	-	647761830	39.67
Small RNA	6134	652217	0.04
Satellites	35838	4217238	0.26
Simple repeats	765726	53044358	3.25
Low complexity	97863	6694649	0.41

Table S4: Abundances for the different toxin families identified in the proteome of *C. gasperettii*

Toxin family	Percentage
SVMPi	8.65%

DISI	12.74%
DC domain	0.26%
CRISP	4.34%
PLA2	5.47%
SVSP	37.38%
SVMP-III	22.19%
PDE	0.02%
LAAO	1.71%
CTL	7.25%

References

Alföldi, J., Di Palma, F., Grabherr, M., Williams, C., Kong, L., Mauceli, E., Russell, P., Lowe, C. B., Glor, R. E., Jaffe, J. D., Ray, D. A., Boissinot, S., Shedlock, A. M., Botka, C., Castoe, T. A., Colbourne, J. K., Fujita, M. K., Moreno, R. G., Ten Hallers, B. F., … Lindblad-Toh, K. (2011). The genome of the green anole lizard and a comparative analysis with birds and mammals. *Nature*, *477*(7366), 587–591. https://doi.org/10.1038/nature10390

Ali, S. A., Jackson, T. N. W., Casewell, N. R., Low, D. H. W., Rossi, S., Baumann, K., Fathinia, B., Visser, J., Nouwens, A., Hendrikx, I., Jones, A., Undheim, E. A., & Fry, B. G. (2015). Extreme venom variation in Middle Eastern vipers: A proteomics comparison of *Eristicophis macmahonii*, *Pseudocerastes fieldi* and *Pseudocerastes persicus*. *Journal of Proteomics*, *116*, 106–113. https://doi.org/10.1016/j.jprot.2014.09.003

Allio, R., Schomaker-Bastos, A., Romiguier, J., Prosdocimi, F., Nabholz, B., & Delsuc, F. (2020). MitoFinder: Efficient automated large-scale extraction of mitogenomic data in target enrichment phylogenomics. *Molecular Ecology Resources*, *20*(4), 892–905. https://doi.org/10.1111/1755-0998.13160

Almeida, D. D., Viala, V. L., Nachtigall, P. G., Broe, M., Gibbs, H. L., Serrano, S. M. D. T., Moura-da-Silva, A. M., Ho, P. L., Nishiyama-Jr, M. Y., & Junqueira-de-Azevedo, I. L. M. (2021). Tracking the recruitment and evolution of snake toxins using the evolutionary context provided by the *Bothrops jararaca* genome. *Proceedings of the National Academy of Sciences*, *118*(20), e2015159118. https://doi.org/10.1073/pnas.2015159118

Al-Sadoon, M. K., & Paray, B. A. (2016). Ecological aspects of the horned viper, *Cerastes cerastes gasperettii* in the central region of Saudi Arabia. *Saudi Journal of Biological Sciences*, *23*(1), 135–138. https://doi.org/10.1016/j.sjbs.2015.10.010

Amr, Z. S., Abu Baker, M. A., & Warrell, D. A. (2020). Terrestrial venomous snakes and snakebites in the Arab countries of the Middle East. *Toxicon*, *177*, 1–15. https://doi.org/10.1016/j.toxicon.2020.01.012

Andrews, S. (2010). *FastQC: a quality control tool for high throughput sequence data.*

Arnold, N. E., Robinson, M. D., & Carranza, S. (2009). A preliminary analysis of phylogenetic relationships and biogeography of the dangerously venomous Carpet Vipers, *Echis* (Squamata, Serpentes, Viperidae) based on mitochondrial DNA sequences. *Amphibia Reptilia*, *30*(2), 273–282. https://doi.org/10.1163/156853809788201090

Avella, I., Calvete, J. J., Sanz, L., Wüster, W., Licata, F., Quesada-Bernat, S., Rodríguez, Y., & Martínez-Freiría, F. (2022). Interpopulational variation and ontogenetic shift in the venom composition of Lataste's viper (*Vipera latastei*, Boscá 1878) from northern Portugal. *Journal of Proteomics*, *263*, 104613. https://doi.org/10.1016/j.jprot.2022.104613

Bao, W., Kojima, K. K., & Kohany, O. (2015). Repbase Update, a database of repetitive elements in eukaryotic genomes. *Mobile DNA*, *6*(1), 11. https://doi.org/10.1186/s13100-015-0041-9

Broad Institute. (2021). *Picard Tools*. Broad Institute, GitHub Repository.

Burriel-Carranza, B., Tejero-Cicuéndez, H., Carné, A., Riaño, G., Talavera, A., Saadi, S. A., Els, J., Šmíd, J., Tamar, K., Tarroso, P., & Carranza, S. (2023). The origin of a mountain biota: Hyper-aridity shaped reptile diversity in an Arabian biodiversity hotspot. https://doi.org/10.1101/2023.04.07.536010

Bylsma, R., Walkup, D. K., Hibbitts, T. J., Ryberg, W. A., Black, A. N., & DeWoody, J. A. (2022). Population genetic and genomic analyses of Western Massasauga (*Sistrurus tergeminus* ssp.): Implications for subspecies delimitation and conservation. *Conservation Genetics*, *23*(2), 271–283. https://doi.org/10.1007/s10592-021-01420-8

Calvete, J. J., Pla, D., Els, J., Carranza, S., Damm, M., Hempel, B.-F., John, E. B. O., Petras, D., Heiss, P., Nalbantsoy, A., Göçmen, B., Süssmuth, R. D., Calderón-Celis, F., Nosti, A. J., & Encinar, J. R. (2021). Combined Molecular and Elemental Mass Spectrometry Approaches for Absolute Quantification of Proteomes: Application to the Venomics Characterization of the Two Species of Desert Black Cobras, *Walterinnesia aegyptia* and *Walterinnesia morgani*. *Journal of Proteome Research*, *20*(11), 5064–5078. https://doi.org/10.1021/acs.jproteome.1c00608

Carranza, S., Els, J., & Burriel-Carranza, B. (2021). *A field guide to the reptiles of Oman*.

Casewell, N. R., Harrison, R. A., Wüster, W., & Wagstaff, S. C. (2009). Comparative venom gland transcriptome surveys of the saw-scaled vipers (Viperidae: *Echis*) reveal substantial intra-family gene diversity and novel venom transcripts. *BMC Genomics*, *10*(1), 564. https://doi.org/10.1186/1471-2164-10-564

Casewell, N. R., Wagstaff, S. C., Wüster, W., Cook, D. A. N., Bolton, F. M. S., King, S. I., Pla, D., Sanz, L., Calvete, J. J., & Harrison, R. A. (2014). Medically important differences in snake venom composition are dictated by distinct postgenomic mechanisms. *Proceedings of the National Academy of Sciences*, *111*(25), 9205–9210. https://doi.org/10.1073/pnas.1405484111

Casewell, N. R., Wüster, W., Vonk, F. J., Harrison, R. A., & Fry, B. G. (2013). Complex cocktails: The evolutionary novelty of venoms. *Trends in Ecology & Evolution*, *28*(4), 219–229. https://doi.org/10.1016/j.tree.2012.10.020

Challis, R., Richards, E., Rajan, J., Cochrane, G., & Blaxter, M. (2020). BlobToolKit – Interactive Quality Assessment of Genome Assemblies. *G3 Genes|Genomes|Genetics*, *10*(4), 1361–1374. https://doi.org/10.1534/g3.119.400908

Chen, S., Zhou, Y., Chen, Y., & Gu, J. (2018). Fastp: An ultra-fast all-in-one FASTQ preprocessor. *Bioinformatics*, *34*(17), i884–i890. https://doi.org/10.1093/bioinformatics/bty560

Cheng, H., Concepcion, G. T., Feng, X., Zhang, H., & Li, H. (2021). Haplotype-resolved de novo assembly using phased assembly graphs with hifiasm. *Nature Methods*, *18*(2), Article 2. https://doi.org/10.1038/s41592-020-01056-5

Dainat, J., Hereñú, D., Dr. K. D. Murray, Davis, E., Crouch, K., LucileSol, Agostinho, N., Pascal-Git, Zollman, Z., & Tayyrov. (2023). *NBISweden/AGAT: AGAT-v1.2.0* (v1.2.0) [Computer software]. Zenodo. https://doi.org/10.5281/ZENODO.3552717

Danecek, P., Bonfield, J. K., Liddle, J., Marshall, J., Ohan, V., Pollard, M. O., Whitwham, A., Keane, T., McCarthy, S. A., Davies, R. M., & Li, H. (2021). Twelve years of SAMtools and BCFtools. *GigaScience*, *10*(2). https://doi.org/10.1093/gigascience/giab008

Dowell, N. L., Giorgianni, M. W., Kassner, V. A., Selegue, J. E., Sanchez, E. E., & Carroll, S. B. (2016). The Deep Origin and Recent Loss of Venom Toxin Genes in Rattlesnakes. *Current Biology*, *26*(18), 2434–2445. https://doi.org/10.1016/j.cub.2016.07.038

Drukewitz, S. H., & Von Reumont, B. M. (2019). The Significance of Comparative Genomics in Modern Evolutionary Venomics. *Frontiers in Ecology and Evolution*, *7*, 163. https://doi.org/10.3389/fevo.2019.00163

Dussex, N., van der Valk, T., Morales, H. E., Wheat, C. W., Díez-del-Molino, D., von Seth, J., Foster, Y., Kutschera, V. E., Guschanski, K., Rhie, A., Phillippy, A. M., Korlach, J., Howe, K., Chow, W., Pelan, S., Mendes Damas, J. D., Lewin, H. A., Hastie, A. R., Formenti, G., … Dalén, L. (2021). Population genomics of the critically endangered kākāpō. *Cell Genomics*, *1*(1), 100002. https://doi.org/10.1016/j.xgen.2021.100002

Egan, D., Amr, Z., Al Johany, A., Els, J., Papenfuss, T., Nilson, Sadek, R., Disi, A., Hraoui-Bloquet, S., Werner, Y., & Anderson, S. (2012). *The IUCN Red List of Threatened Species: Cerastes gasperettii* [dataset]. https://doi.org/10.2305/IUCN.UK.2012.RLTS.T164599A1060588.en

Fahmi, L., Makran, B., Pla, D., Sanz, L., Oukkache, N., Lkhider, M., Harrison, R. A., Ghalim, N., & Calvete, J. J. (2012). Venomics and antivenomics profiles of North African *Cerastes cerastes* and *C. vipera* populations reveals a potentially important therapeutic weakness. *Journal of Proteomics*, *75*(8), 2442–2453. https://doi.org/10.1016/j.jprot.2012.02.021

Ferraz, C. R., Arrahman, A., Xie, C., Casewell, N. R., Lewis, R. J., Kool, J., & Cardoso, F. C. (2019). Multifunctional Toxins in Snake Venoms and Therapeutic Implications: From Pain to Hemorrhage and Necrosis. *Frontiers in Ecology and Evolution*, *7*. https://www.frontiersin.org/articles/10.3389/fevo.2019.00218

Flynn, J. M., Hubley, R., Goubert, C., Rosen, J., Clark, A. G., Feschotte, C., & Smit, A. F. (2020). RepeatModeler2 for automated genomic discovery of transposable element families. *Proceedings of the National Academy of Sciences*, *117*(17), 9451–9457. https://doi.org/10.1073/pnas.1921046117

Formenti, G., Abueg, L., Brajuka, A., Brajuka, N., Gallardo-Alba, C., Giani, A., Fedrigo, O., & Jarvis, E. D. (2022). Gfastats: Conversion, evaluation and manipulation of genome sequences using assembly graphs. *Bioinformatics*, *38*(17), 4214–4216. https://doi.org/10.1093/bioinformatics/btac460

Frantz, L. A. F., Bradley, D. G., Larson, G., & Orlando, L. (2020). Animal domestication in the era of ancient genomics. *Nature Reviews Genetics*, *21*(8), Article 8. https://doi.org/10.1038/s41576-020-0225-0

Fry, B. (Ed.). (2015). *Venomous reptiles and their toxins: Evolution, pathophysiology, and biodiscovery*. Oxford University Press.

Fry, B. G., Roelants, K., Champagne, D. E., Scheib, H., Tyndall, J. D. A., King, G. F., Nevalainen, T. J., Norman, J. A., Lewis, R. J., Norton, R. S., Renjifo, C., & de la Vega, R. C. R. (2009). The toxicogenomic multiverse: Convergent recruitment of proteins into animal venoms. *Annual Review of Genomics and Human Genetics*, *10*, 483–511. https://doi.org/10.1146/annurev.genom.9.081307.164356

Fry, B. G., Scheib, H., van der Weerd, L., Young, B., McNaughtan, J., Ramjan, S. F. R., Vidal, N., Poelmann, R. E., & Norman, J. A. (2008). Evolution of an Arsenal: Structural and Functional Diversification of the Venom System in the Advanced Snakes (Caenophidia)*. *Molecular & Cellular Proteomics*, *7*(2), 215–246. https://doi.org/10.1074/mcp.M700094-MCP200

Fry, B. G., & Wüster, W. (2004). Assembling an Arsenal: Origin and Evolution of the Snake Venom Proteome Inferred from Phylogenetic Analysis of Toxin Sequences. *Molecular Biology and Evolution*, *21*(5), 870–883. https://doi.org/10.1093/molbev/msh091

Gabriel, L., Hoff, K. J., Brůna, T., Borodovsky, M., & Stanke, M. (2021). TSEBRA: Transcript selector for BRAKER. *BMC Bioinformatics*, *22*(1), 566. https://doi.org/10.1186/s12859-021-04482-0

Geneva, A. J., Park, S., Bock, D. G., De Mello, P. L. H., Sarigol, F., Tollis, M., Donihue, C. M., Reynolds, R. G., Feiner, N., Rasys, A. M., Lauderdale, J. D., Minchey, S. G., Alcala, A. J., Infante, C. R., Kolbe, J. J., Schluter, D., Menke, D. B., & Losos, J. B. (2022). Chromosome-scale genome assembly of the brown anole (*Anolis sagrei*), an emerging model species. *Communications Biology*, *5*(1), 1126. https://doi.org/10.1038/s42003-022-04074-5

Ghurye, J., Rhie, A., Walenz, B. P., Schmitt, A., Selvaraj, S., Pop, M., Phillippy, A. M., & Koren, S. (2019). Integrating Hi-C links with assembly graphs for chromosome-scale assembly. *PLoS Computational Biology*, *15*(8), e1007273. https://doi.org/10.1371/journal.pcbi.1007273

Gilbert, C., Meik, J. M., Dashevsky, D., Card, D. C., Castoe, T. A., & Schaack, S. (2014). Endogenous hepadnaviruses, bornaviruses and circoviruses in snakes. *Proceedings of the Royal Society B: Biological Sciences*, *281*(1791), 20141122. https://doi.org/10.1098/rspb.2014.1122

Giorgianni, M. W., Dowell, N. L., Griffin, S., Kassner, V. A., Selegue, J. E., & Carroll, S. B. (2020). The origin and diversification of a novel protein family in venomous snakes. *Proceedings of the National Academy of Sciences*, *117*(20), 10911–10920. https://doi.org/10.1073/pnas.1920011117

Glennie, K. W., & Singhvi, A. K. (2002). Event stratigraphy, paleoenvironment and chronology of SE Arabian deserts. *Quaternary Science Reviews*, *21*(7), 853–869. https://doi.org/10.1016/S0277-3791(01)00133-0

Green, R. E., Braun, E. L., Armstrong, J., Earl, D., Nguyen, N., Hickey, G., Vandewege, M. W., St. John, J. A., Capella-Gutiérrez, S., Castoe, T. A., Kern, C., Fujita, M. K., Opazo, J. C., Jurka, J., Kojima, K. K., Caballero, J., Hubley, R. M., Smit, A. F., Platt, R. N., … Ray, D. A. (2014). Three crocodilian genomes reveal ancestral patterns of evolution among archosaurs. *Science*, *346*(6215), 1254449. https://doi.org/10.1126/science.1254449

Guan, D., McCarthy, S. A., Wood, J., Howe, K., Wang, Y., & Durbin, R. (2020). Identifying and removing haplotypic duplication in primary genome assemblies. *Bioinformatics*, *36*(9), 2896–2898. https://doi.org/10.1093/bioinformatics/btaa025

Guindon, S., Dufayard, J.-F., Lefort, V., Anisimova, M., Hordijk, W., & Gascuel, O. (2010). New Algorithms and Methods to Estimate Maximum-Likelihood Phylogenies: Assessing the Performance of PhyML 3.0. *Systematic Biology*, *59*(3), 307–321. https://doi.org/10.1093/sysbio/syq010

Gurevich, A., Saveliev, V., Vyahhi, N., & Tesler, G. (2013). QUAST: Quality assessment tool for genome assemblies. *Bioinformatics (Oxford, England)*, *29*(8), 1072–1075. https://doi.org/10.1093/bioinformatics/btt086

Gutiérrez, J. M., Warrell, D. A., Williams, D. J., Jensen, S., Brown, N., Calvete, J. J., ... & Global Snakebite Initiative. (2013). The need for full integration of snakebite envenoming within a global strategy to combat the neglected tropical diseases: the way forward. *PLoS neglected tropical diseases*, *7*(6), e2162.

Gutiérrez, J. M., Calvete, J. J., Habib, A. G., Harrison, R. A., Williams, D. J., & Warrell, D. A. (2017). Snakebite envenoming. *Nature Reviews Disease Primers*, *3*(1), Article 1. https://doi.org/10.1038/nrdp.2017.63

Hirst, Samuel R., Rhett M. Rautsaw, Cameron M. VanHorn, Marc A. Beer, Preston J. McDonald, Ramsés Alejandro Rosales García, Bruno Rodriguez Lopez et al. "Where the "ruber" Meets the Road: Using the Genome of the Red Diamond Rattlesnake to Unravel the Evolutionary Processes Driving Venom Evolution." *Genome Biology and Evolution* 16, no. 9 (2024): https://doi.org/10.1093/gbe/evae198

Hogan, M. P., Holding, M. L., Nystrom, G. S., Colston, T. J., Bartlett, D. A., Mason, A. J., Ellsworth, S. A., Rautsaw, R. M., Lawrence, K. C., Strickland, J. L., He, B., Fraser, P., Margres, M. J., Gilbert, D. M., Gibbs, H. L., Parkinson, C. L., & Rokyta, D. R. (2024). The genetic regulatory architecture and epigenomic basis for age-related changes in rattlesnake venom. *Proceedings of the National Academy of Sciences*, *121*(16), e2313440121. https://doi.org/10.1073/pnas.2313440121

Jan, V., Maroun, R. C., Robbe-Vincent, A., De Haro, L., & Choumet, V. (2002). Toxicity evolution of *Vipera aspis aspis* venom: Identification and molecular modeling of a novel phospholipase A2 heterodimer neurotoxin11Nucleotide sequence data reported are available in the EMBL database under the accession numbers AJ459806 and AJ459807. *FEBS Letters*, *527*(1), 263–268. https://doi.org/10.1016/S0014-5793(02)03205-2

Jin, J.-J., Yu, W.-B., Yang, J.-B., Song, Y., dePamphilis, C. W., Yi, T.-S., & Li, D.-Z. (2020). GetOrganelle: A fast and versatile toolkit for accurate de novo assembly of organelle genomes. *Genome Biology*, *21*(1), 241. https://doi.org/10.1186/s13059-020-02154-5

Jones, P., Binns, D., Chang, H.-Y., Fraser, M., Li, W., McAnulla, C., McWilliam, H., Maslen, J., Mitchell, A., Nuka, G., Pesseat, S., Quinn, A. F., Sangrador-Vegas, A., Scheremetjew, M., Yong, S.-Y., Lopez, R., & Hunter, S. (2014). InterProScan 5: Genome-scale protein function classification. *Bioinformatics*, *30*(9), 1236–1240. https://doi.org/10.1093/bioinformatics/btu031

Kalita, B., Mackessy, S. P., & Mukherjee, A. K. (2018). Proteomic analysis reveals geographic variation in venom composition of Russell's Viper in the Indian subcontinent: Implications for clinical manifestations post-envenomation and antivenom treatment. *Expert Review of Proteomics*, *15*(10), 837–849. https://doi.org/10.1080/14789450.2018.1528150

Katoh, K., & Standley, D. M. (2013). MAFFT Multiple Sequence Alignment Software Version 7: Improvements in Performance and Usability. *Molecular Biology and Evolution*, *30*(4), 772–780. https://doi.org/10.1093/molbev/mst010

Keilwagen, J., Hartung, F., & Grau, J. (2019). GeMoMa: Homology-Based Gene Prediction Utilizing Intron Position Conservation and RNA-seq Data. In M. Kollmar (Ed.), *Gene Prediction* (Vol. 1962, pp. 161–177). Springer New York. https://doi.org/10.1007/978-1-4939-9173-0_9

Kiełbasa, S. M., Wan, R., Sato, K., Horton, P., & Frith, M. C. (2011). Adaptive seeds tame genomic sequence comparison. *Genome Research*, *21*(3), 487–493. https://doi.org/10.1101/gr.113985.110

Kim, D., Paggi, J. M., Park, C., Bennett, C., & Salzberg, S. L. (2019). Graph-based genome alignment and genotyping with HISAT2 and HISAT-genotype. *Nature Biotechnology*, *37*(8), 907–915. https://doi.org/10.1038/s41587-019-0201-4

King, G. F. (2011). Venoms as a platform for human drugs: Translating toxins into therapeutics. *Expert Opinion on Biological Therapy*, *11*(11), 1469–1484. https://doi.org/10.1517/14712598.2011.621940

Li, D., Luo, R., Liu, C.-M., Leung, C.-M., Ting, H.-F., Sadakane, K., Yamashita, H., & Lam, T.-W. (2016). MEGAHIT v1.0: A fast and scalable metagenome assembler driven by advanced methodologies and community practices. *Methods*, *102*, 3–11. https://doi.org/10.1016/j.ymeth.2016.02.020

Li, H. (2013). *Aligning sequence reads, clone sequences and assembly contigs with BWA-MEM*. http://arxiv.org/abs/1303.3997

Li, H., & Durbin, R. (2011). Inference of human population history from individual whole-genome sequences. *Nature 2011 475:7357*, *475*(7357), 493–496. https://doi.org/10.1038/nature10231

Li, H., Handsaker, B., Wysoker, A., Fennell, T., Ruan, J., Homer, N., Marth, G., Abecasis, G., & Durbin, R. (2009). The Sequence Alignment/Map format and SAMtools. *Bioinformatics*, *25*(16), 2078–2079. https://doi.org/10.1093/bioinformatics/btp352

Li, L., Huang, J., & Lin, Y. (2018). Snake Venoms in Cancer Therapy: Past, Present and Future. *Toxins*, *10*(9), 346. https://doi.org/10.3390/toxins10090346

Love, M. I., Huber, W., & Anders, S. (2014). Moderated estimation of fold change and dispersion for RNA-seq data with DESeq2. *Genome Biology*, *15*(12), 550. https://doi.org/10.1186/s13059-014-0550-8

Mackessy, S. P. (2010). Evolutionary trends in venom composition in the Western Rattlesnakes (*Crotalus viridis* sensu lato): Toxicity vs. tenderizers. *Toxicon*, *55*(8), 1463–1474. https://doi.org/10.1016/j.toxicon.2010.02.028

Margres, M. J., McGivern, J. J., Wray, K. P., Seavy, M., Calvin, K., & Rokyta, D. R. (2014). Linking the transcriptome and proteome to characterize the venom of the eastern diamondback rattlesnake (*Crotalus adamanteus*). *Journal of Proteomics*, *96*, 145–158. https://doi.org/10.1016/j.jprot.2013.11.001

Margres, M. J., Rautsaw, R. M., Strickland, J. L., Mason, A. J., Schramer, T. D., Hofmann, E. P., Stiers, E., Ellsworth, S. A., Nystrom, G. S., Hogan, M. P., Bartlett, D. A., Colston, T. J., Gilbert, D. M., Rokyta, D. R., & Parkinson, C. L. (2021). The Tiger Rattlesnake genome reveals a complex genotype underlying a simple venom phenotype. *Proceedings of the National Academy of Sciences*, *118*(4), e2014634118. https://doi.org/10.1073/pnas.2014634118

Margres, M. J., Wray, K. P., Sanader, D., McDonald, P. J., Trumbull, L. M., Patton, A. H., & Rokyta, D. R. (2021). Varying Intensities of Introgression Obscure Incipient Venom-Associated

Speciation in the Timber Rattlesnake (*Crotalus horridus*). *Toxins*, *13*(11), Article 11. https://doi.org/10.3390/toxins13110782

Martin, M. (2011). Cutadapt removes adapter sequences from high-throughput sequencing reads. *EMBnet.Journal*, *17*(1), 10. https://doi.org/10.14806/ej.17.1.200

McKenna, A., Hanna, M., Banks, E., Sivachenko, A., Cibulskis, K., Kernytsky, A., Garimella, K., Altshuler, D., Gabriel, S., Daly, M., & DePristo, M. A. (2010). The genome analysis toolkit: A MapReduce framework for analyzing next-generation DNA sequencing data. *Genome Research*, *20*(9), 1297–1303. https://doi.org/10.1101/gr.107524.110

Mochales-Riaño, G., Burriel-Carranza, B., Barros, M. I., Velo-Antón, G., Talavera, A., Spilani, L., Tejero-Cicuéndez, H., Crochet, P.-A., Piris, A., García-Cardenete, L., Busais, S., Els, J., Shobrak, M., Brito, J. C., Šmíd, J., Carranza, S., & Martínez-Freiría, F. (2024). Hidden in the sand: Phylogenomics unravel an unexpected evolutionary history for the desert-adapted vipers of the genus *Cerastes*. *Molecular Phylogenetics and Evolution*, *191*, 107979. https://doi.org/10.1016/j.ympev.2023.107979

Myers, E. A., Strickland, J. L., Rautsaw, R. M., Mason, A. J., Schramer, T. D., Nystrom, G. S., Hogan, M. P., Yooseph, S., Rokyta, D. R., & Parkinson, C. L. (2022). De Novo Genome Assembly Highlights the Role of Lineage-Specific Gene Duplications in the Evolution of Venom in Fea's Viper (*Azemiops feae*). *Genome Biology and Evolution*, *14*(7), evac082. https://doi.org/10.1093/gbe/evac082

Orteu, A., & Jiggins, C. D. (2020). The genomics of coloration provides insights into adaptive evolution. *Nature Reviews Genetics*, *21*(8), Article 8. https://doi.org/10.1038/s41576-020-0234-z

Osipov, A., & Utkin, Y. (2023). What Are the Neurotoxins in Hemotoxic Snake Venoms? *International Journal of Molecular Sciences*, *24*(3), Article 3. https://doi.org/10.3390/ijms24032919

Pardos-Blas, J. R., Irisarri, I., Abalde, S., Afonso, C. M. L., Tenorio, M. J., & Zardoya, R. (2021). The genome of the venomous snail *Lautoconus ventricosus* sheds light on the origin of conotoxin diversity. *GigaScience*, *10*(5), giab037. https://doi.org/10.1093/gigascience/giab037

Pertea, M., Pertea, G. M., Antonescu, C. M., Chang, T.-C., Mendell, J. T., & Salzberg, S. L. (2015). StringTie enables improved reconstruction of a transcriptome from RNA-seq reads. *Nature Biotechnology*, *33*(3), 290–295. https://doi.org/10.1038/nbt.3122

Pook, C. E., Joger, U., Stümpel, N., & Wüster, W. (2009). When continents collide: Phylogeny, historical biogeography and systematics of the medically important viper genus *Echis* (Squamata: Serpentes: Viperidae). *Molecular Phylogenetics and Evolution*, *53*(3), 792–807. https://doi.org/10.1016/j.ympev.2009.08.002

R Core Team. (2021a). *R: A Language and Environment for Statistical Computing*. R Foundation for Statistical Computing. https://www.R-project.org/

Ranallo-Benavidez, T. R., Jaron, K. S., & Schatz, M. C. (2020). GenomeScope 2.0 and Smudgeplot for reference-free profiling of polyploid genomes. *Nature Communications*, *11*(1), Article 1. https://doi.org/10.1038/s41467-020-14998-3

Rhie, A., McCarthy, S. A., Fedrigo, O., Damas, J., Formenti, G., Koren, S., Uliano-Silva, M., Chow, W., Fungtammasan, A., Gedman, G. L., Cantin, L. J., Thibaud-Nissen, F., Haggerty, L., Lee, C., Ko, B. J., Kim, J., Bista, I., Smith, M., Haase, B., … Jarvis, E. D. (2020). *Towards complete and error-free genome assemblies of all vertebrate species* (p. 2020.05.22.110833). bioRxiv. https://doi.org/10.1101/2020.05.22.110833

Rhie, A., Walenz, B. P., Koren, S., & Phillippy, A. M. (2020). Merqury: Reference-free quality, completeness, and phasing assessment for genome assemblies. *Genome Biology*, *21*(1), 245. https://doi.org/10.1186/s13059-020-02134-9

Rokyta, D. R., Margres, M. J., Ward, M. J., & Sanchez, E. E. (2017). The genetics of venom ontogeny in the eastern diamondback rattlesnake (*Crotalus adamanteus*). *PeerJ*, *5*, e3249. https://doi.org/10.7717/peerj.3249

Russell, F. E., & Campbell, J. R. (2015). *Venomous terrestrial Snakes of the Middle East*. Edition Chimaira.

Saethang, T., Somparn, P., Payungporn, S., Sriswasdi, S., Yee, K. T., Hodge, K., Knepper, M. A., Chanhome, L., Khow, O., Chaiyabutr, N., Sitprija, V., & Pisitkun, T. (2022). Identification of

Daboia siamensis venome using integrated multi-omics data. *Scientific Reports*, *12*(1), Article 1. https://doi.org/10.1038/s41598-022-17300-1

San-Jose, L. M., & Roulin, A. (2017). Genomics of coloration in natural animal populations. *Philosophical Transactions of the Royal Society B: Biological Sciences*, *372*(1724), 20160337. https://doi.org/10.1098/rstb.2016.0337

Schield, D. R., Card, D. C., Hales, N. R., Perry, B. W., Pasquesi, G. M., Blackmon, H., Adams, R. H., Corbin, A. B., Smith, C. F., Ramesh, B., Demuth, J. P., Betrán, E., Tollis, M., Meik, J. M., Mackessy, S. P., & Castoe, T. A. (2019). The origins and evolution of chromosomes, dosage compensation, and mechanisms underlying venom regulation in snakes. *Genome Research*, *29*(4), 590–601. https://doi.org/10.1101/gr.240952.118

Schield, D. R., Perry, B. W., Adams, R. H., Holding, M. L., Nikolakis, Z. L., Gopalan, S. S., Smith, C. F., Parker, J. M., Meik, J. M., DeGiorgio, M., Mackessy, S. P., & Castoe, T. A. (2022). The roles of balancing selection and recombination in the evolution of rattlesnake venom. *Nature Ecology & Evolution*, *6*(9), 1367–1380. https://doi.org/10.1038/s41559-022-01829-5

Schneemann, M., Cathomas, R., Laidlaw, S. T., El Nahas, A. M., Theakston, R. D. G., & Warrell, D. A. (2004). Life-threatening envenoming by the Saharan horned viper (*Cerastes cerastes*) causing micro-angiopathic haemolysis, coagulopathy and acute renal failure: Clinical cases and review. *QJM: An International Journal of Medicine*, *97*(11), 717–727. https://doi.org/10.1093/qjmed/hch118

Simão, F. A., Waterhouse, R. M., Ioannidis, P., Kriventseva, E. V., & Zdobnov, E. M. (2015). BUSCO: Assessing genome assembly and annotation completeness with single-copy orthologs. *Bioinformatics (Oxford, England)*, *31*(19), 3210–3212. https://doi.org/10.1093/bioinformatics/btv351

Šmíd, J., & Tolley, K. A. (2019). Calibrating the tree of vipers under the fossilized birth-death model. *Scientific Reports*, *9*(1), 5510. https://doi.org/10.1038/s41598-019-41290-2

Smith, C. F., Nikolakis, Z. L., Perry, B. W., Schield, D. R., Meik, J. M., Saviola, A. J., Castoe, T. A., Parker, J., & Mackessy, S. P. (2023). The best of both worlds? Rattlesnake hybrid zones generate complex combinations of divergent venom phenotypes that retain high toxicity. *Biochimie*. https://doi.org/10.1016/j.biochi.2023.07.008

Solovyev, V., Kosarev, P., Seledsov, I., & Vorobyev, D. (2006). Automatic annotation of eukaryotic genes, pseudogenes and promoters. *Genome Biology*, *7*(Suppl 1), S10. https://doi.org/10.1186/gb-2006-7-s1-s10

Suryamohan, K., Krishnankutty, S. P., Guillory, J., Jevit, M., Schröder, M. S., Wu, M., Kuriakose, B., Mathew, O. K., Perumal, R. C., Koludarov, I., Goldstein, L. D., Senger, K., Dixon, M. D., Velayutham, D., Vargas, D., Chaudhuri, S., Muraleedharan, M., Goel, R., Chen, Y.-J. J., … Seshagiri, S. (2020). The Indian cobra reference genome and transcriptome enables comprehensive identification of venom toxins. *Nature Genetics*, *52*(1), 106–117. https://doi.org/10.1038/s41588-019-0559-8

Tang, H., Bowers, J. E., Wang, X., Ming, R., Alam, M., & Paterson, A. H. (2008). Synteny and Collinearity in Plant Genomes. *Science*, *320*(5875), 486–488. https://doi.org/10.1126/science.1153917

Tang, H., Krishnakumar, V., Jingping Li, Tiany, MichelMoser, Maria, & Yim, W. C. (2017). *tanghaibao/jcvi: JCVI v0.7.5* (v0.7.5) [Computer software]. Zenodo. https://doi.org/10.5281/ZENODO.846919

Tang, S., Lomsadze, A., & Borodovsky, M. (2015). Identification of protein coding regions in RNA transcripts. *Nucleic Acids Research*, *43*(12), e78–e78. https://doi.org/10.1093/nar/gkv227

Tasoulis, T., & Isbister, G. (2017). A Review and Database of Snake Venom Proteomes. *Toxins*, *9*(9), 290. https://doi.org/10.3390/toxins9090290

Tempel, S. (2012). Using and Understanding RepeatMasker. In Y. Bigot (Ed.), *Mobile Genetic Elements* (Vol. 859, pp. 29–51). Humana Press. https://doi.org/10.1007/978-1-61779-603-6_2

Thongchum, R., Singchat, W., Laopichienpong, N., Tawichasri, P., Kraichak, E., Prakhongcheep, O., Sillapaprayoon, S., Muangmai, N., Baicharoen, S., Suntrarachun, S., Chanhome, L., Peyachoknagul, S., & Srikulnath, K. (2019). Diversity of PBI-DdeI satellite DNA in snakes correlates with rapid independent evolution and different functional roles. *Scientific Reports*, *9*(1), 15459. https://doi.org/10.1038/s41598-019-51863-w

Title, P. O., Singhal, S., Grundler, M. C., Costa, G. C., Pyron, R. A., Colston, T. J., Grundler, M. R., Prates, I., Stepanova, N., Jones, M. E. H., Cavalcanti, L. B. Q., Colli, G. R., Di-Poï, N., Donnellan, S. C., Moritz, C., Mesquita, D. O., Pianka, E. R., Smith, S. A., Vitt, L. J., & Rabosky, D. L. (2024). The macroevolutionary singularity of snakes. *Science*, *383*(6685), 918–923. https://doi.org/10.1126/science.adh2449

Uetz, P. (2021). The Reptile Database: Curating the biodiversity literature without funding. *Biodiversity Information Science and Standards*, *5*, e75448. https://doi.org/10.3897/biss.5.75448

Vitt, L. J., & Caldwell, J. P. (2014). *Herpetology: An introductory biology of amphibians and reptiles* (Fourth edition). Elsevier, AP, Academic Press is an imprint of Elsevier.

Vonk, F. J., Casewell, N. R., Henkel, C. V., Heimberg, A. M., Jansen, H. J., McCleary, R. J. R., Kerkkamp, H. M. E., Vos, R. A., Guerreiro, I., Calvete, J. J., Wüster, W., Woods, A. E., Logan, J. M., Harrison, R. A., Castoe, T. A., De Koning, A. P. J., Pollock, D. D., Yandell, M., Calderon, D., … Richardson, M. K. (2013). The king cobra genome reveals dynamic gene evolution and adaptation in the snake venom system. *Proceedings of the National Academy of Sciences*, *110*(51), 20651–20656. https://doi.org/10.1073/pnas.1314702110

Vyas, V. K., Brahmbhatt, K., Bhatt, H., & Parmar, U. (2013). Therapeutic potential of snake venom in cancer therapy: Current perspectives. *Asian Pacific Journal of Tropical Biomedicine*, *3*(2), 156–162. https://doi.org/10.1016/S2221-1691(13)60042-8

Walker, B. J., Abeel, T., Shea, T., Priest, M., Abouelliel, A., Sakthikumar, S., Cuomo, C. A., Zeng, Q., Wortman, J., Young, S. K., & Earl, A. M. (2014). Pilon: An Integrated Tool for Comprehensive Microbial Variant Detection and Genome Assembly Improvement. *PLOS ONE*, *9*(11), e112963. https://doi.org/10.1371/journal.pone.0112963

Weinstein, S. A., White, J., Keyler, D. E., & Warrell, D. A. (2013). Non-front-fanged colubroid snakes: A current evidence-based analysis of medical significance. *Toxicon*, *69*, 103–113. https://doi.org/10.1016/j.toxicon.2013.02.003

Werren, J. H., Richards, S., Desjardins, C. A., Niehuis, O., Gadau, J., Colbourne, J. K., THE NASONIA GENOME WORKING GROUP, Beukeboom, L. W., Desplan, C., Elsik, C. G., Grimmelikhuijzen, C. J. P., Kitts, P., Lynch, J. A., Murphy, T., Oliveira, D. C. S. G., Smith, C. D., Zande, L. van de, Worley, K. C., Zdobnov, E. M., … Gibbs, R. A. (2010). Functional and Evolutionary Insights from the Genomes of Three Parasitoid Nasonia Species. *Science*, *327*(5963), 343–348. https://doi.org/10.1126/science.1178028

Westeen, E. P., Escalona, M., Holding, M. L., Beraut, E., Fairbairn, C., Marimuthu, M. P. A., Nguyen, O., Perri, R., Fisher, R. N., Toffelmier, E., Shaffer, H. B., & Wang, I. J. (2023). A genome assembly for the southern Pacific rattlesnake, *Crotalus oreganus helleri* , in the western rattlesnake species complex. *Journal of Heredity*, *114*(6), 681–689. https://doi.org/10.1093/jhered/esad045

Wickham, H. (2016). *ggplot2: Elegant Graphics for Data Analysis*. Springer-Verlag New York. https://ggplot2.tidyverse.org

Williams, D. J., Faiz, M. A., Abela-Ridder, B., Ainsworth, S., Bulfone, T. C., Nickerson, A. D., Habib, A. G., Junghanss, T., Fan, H. W., Turner, M., Harrison, R. A., & Warrell, D. A. (2019). Strategy for a globally coordinated response to a priority neglected tropical disease: Snakebite envenoming. *PLOS Neglected Tropical Diseases*, *13*(2), e0007059. https://doi.org/10.1371/journal.pntd.0007059

Wüster, W., Peppin, L., Pook, C. E., & Walker, D. E. (2008). A nesting of vipers: Phylogeny and historical biogeography of the Viperidae (Squamata: Serpentes). *Molecular Phylogenetics and Evolution*, *49*(2), 445–459. https://doi.org/10.1016/j.ympev.2008.08.019

Zancolli, G., Calvete, J. J., Cardwell, M. D., Greene, H. W., Hayes, W. K., Hegarty, M. J., Herrmann, H.-W., Holycross, A. T., Lannutti, D. I., Mulley, J. F., Sanz, L., Travis, Z. D., Whorley, J. R., Wüster, C. E., & Wüster, W. (2019). When one phenotype is not enough: Divergent evolutionary trajectories govern venom variation in a widespread rattlesnake species. *Proceedings of the Royal Society B: Biological Sciences*, *286*(1898), 20182735. https://doi.org/10.1098/rspb.2018.2735

Zancolli, G., Reijnders, M., Waterhouse, R. M., & Robinson-Rechavi, M. (2022). Convergent evolution of venom gland transcriptomes across Metazoa. *Proceedings of the National Academy of Sciences*, *119*(1), e2111392119. https://doi.org/10.1073/pnas.2111392119

Manuscript track changes

Click here to access/download;Manuscript;Manuscript track changes.docx

Chromosome-level reference genome for the medically important Arabian horned viper (*Cerastes gasperettii*)

Gabriel Mochales-Riaño[1], Samuel R. Hirst[2], Adrián Talavera[1], Bernat Burriel-Carranza[1,3], Viviana Pagone[1], Maria Estarellas[1], Theo Busschau[4], Stéphane Boissinot[4], Michael P. Hogan[5,6], Jordi Tena-Garcés[7], Davinia Pla[7], Juan J. Calvete[7], Johannes Els[8], Mark J. Margres[2], Salvador Carranza[1]

[1] IBE, Institute of Evolutionary Biology (CSIC-Universitat Pompeu Fabra)

[2] Department of Integrative Biology, University of South Florida, Tampa, FL 33620, USA

[3] Museu de Ciències Naturals de Barcelona, P° Picasso s/n, Parc Ciutadella, 08003 Barcelona, Spain

[4] New York University Abu Dhabi, Abu Dhabi, United Arab Emirates,

[5] Department of Biological Sciences, Florida State University, Tallahassee, FL 33306 USA

[6] University of Michigan, Department of Ecology and Evolutionary Biology, Ann Arbor, MI (48109-1085) USA

[7] Evolutionary and Translational Venomics Laboratory, Consejo Superior de Investigaciones Científicas (CSIC) 46010 Valencia, Spain

[8] Breeding Centre for Endangered Arabian Wildlife, Environment and Protected Areas Authority, Sharjah, United Arab Emirates

Corresponding: gabriel.mochales@csic.es

Abstract

Venoms have traditionally been studied from a proteomic and/or transcriptomic perspective, often overlooking the true genetic complexity underlying venom production. The recent surge in genome-based venom research (sometimes called “venomics”) has proven to be instrumental in deepening our ~~molecular~~ understanding of venom evolution at the molecular level, particularly through the identification and mapping of toxin-coding loci across the broader chromosomal architecture. Although venomous snakes are a model system in venom research, the number of high-quality reference genomes in the group remains limited. In this study, we present a chromosome-resolution reference genome for

the Arabian horned viper (*Cerastes gasperettii*), a venomous snake native to the Arabian Peninsula. Our highly-contiguous genome (genome size: 1.63 Gbp, contig N50: 45.6 Mbp and BUSCO: 92.8%) allowed us to explore macrochromosomal rearrangements within the Viperidae family, as well as across squamates. We identified the main highly-expressed toxin genes within the venom glands ~~compousing~~ comprising the venom's core, in line with our proteomic results. We also compared microsyntenic changes in the main toxin gene clusters with those of other venomous snake species, highlighting the pivotal role of gene duplication and loss in the emergence and diversification of Snake Venom Metalloproteinases (SVMPs) and Snake Venom Serine Proteases (SVSPs) for *Cerastes gasperettii*. Using Illumina short-read sequencing data, we reconstructed the demographic history and genome-wide ~~diversity~~ heterozigosity of the species, revealing how historical aridity likely drove population expansions. Finally, this study highlights the importance of using long-read sequencing as well as chromosome-level reference genomes to disentangle the origin and diversification of toxin gene families in venomous snake species.

Keywords: Toxin evolution; ~~microsynteny~~gene synteny; genomics; transcriptomics; venom

Background

The rise of genomics in non-model organisms has led to an increase in the number of high-quality reference genomes available in recent years (Dussex et al., 2021; Hogan et al., 2024; Margres et al., 2021a; Pardos-Blas et al., 2021; Schield et al., 2019; Suryamohan et al., 2020). ~~Such A~~Aadvances in sequencing technologies have catalyzed the study of several complex traits from a genomic perspective, such as coloration, domestication, or venom, among others (Drukewitz & Von Reumont, 2019; Frantz et al., 2020; Margres et al., 2021a; Orteu & Jiggins, 2020; San-Jose & Roulin, 2017). Among these, venom genomic research has been particularly important in enhancing our understanding of the origin, evolution and dynamics of this medically relevant trait (Casewell et al., 2013; Dowell et al., 2016; Giorgianni et al., 2020; Werren et al., 2010). Venom is a potentially lethal cocktail rich in proteins and peptides (from now on referred to as "toxins") which are actively secreted by specialized venom glands (Casewell et al., 2013; Fry et al., 2009). Toxins can have different effects depending on

their type, interactions with other molecules, and the organism in which they are introduced, with convergent outcomes in different taxa (Fry et al., 2009; Zancolli et al., 2022). Historically, venom research has primarily been conducted using proteomic (and transcriptomic) approaches (see Drukewitz & Von Reumont (2019) and references therein). The identification of venom toxins and the characterization of their evolution using reference genomes is a recent and novel field at all taxonomic levels (Vonk et al., 2013). Previous works have shown that changes in gene regulation can result in the activation and deactivation of venom-coding genes at all taxonomic levels and within the same individual (Avella et al., 2022; Hogan et al., 2024; Margres, Rautsaw, et al., 2021; Zancolli et al., 2022). This suggests, suggesting that transcriptomic and proteomic data are critical for studying venom in conjunction with well annotated reference genomes to exclusively studying the expression of venom toxins (i.e., proteomics or transcriptomics) is insufficient to disentangle the complete number and biochemical nature of the toxins an individual can potentially transcribe (Drukewitz & Von Reumont, 2019). Ultimately, the study of venom genomics may yield evolutionary insights into antivenom or drug discovery, as it enables the identification of unexpressed toxin-coding genes. These genes, often overlooked by transcriptomic or proteomic approaches unless ontogeny analyses or in-depth venom expression studies are performed, may target unique physiological pathways. Such discoveries could lead to it can identify unexpressed toxin-coding genes that target specific physiological pathways, potentially leading to new novel therapies for human illnesses including but not limited to cancer (Casewell et al., 2013; King, 2011; L. Li et al., 2018; Vyas et al., 2013). Unexpressed toxin-coding genes are particularly noteworthy as they may represent evolutionary 'reservoirs' of bioactive molecules. These genes could encode toxins with unique mechanisms of action, offering untapped potential for drug discovery or therapeutic innovation.

Venom has evolved independently in multiple groups including cnidarians, molluscs, arthropods, squamates and even mammals (Casewell et al., 2013; Fry et al., 2009). However, vVenomous snakes are one of the most life-threatening animal groups to humans (Williams et al., 2019) and, therefore, the a fundamental medically relevant model system in venom research. Venomous snakes are a diverse group with more than 600 species (Uetz, 2021), where venom has evolved with the objective of immobilizing and digesting their prey (Fry & Wüster, 2004). From those, more than 370

Formatted: English (United States)

Formatted: Font color: Black

species have been classified as of medically important~~ce~~ by the World Health Organization (WHO) due to their potential severe effects on humans (Gutiérrez et al., 2013). ~~In fact, s~~Snakebite is considered a neglected tropical disease, with annual mortality exceeding 100,000 victims worldwide (Gutiérrez et al., 2017; Williams et al., 2019). ~~Within venomous snakes, t~~The most medically important venomous snake families are Elapidae, Viperidae and Atractaspididae (Tasoulis & Isbister, 2017), although within Colubridae (*sensu lato*) there are certain medically important venomous species as well (Weinstein et al., 2013). Envenomation by certain members of these families can result in a range of pathologies, spanning neurotoxic, hemotoxic, and/or cytotoxic effects ~~(among others)~~ depending on the number and composition of toxins. Neurotoxic venoms primarily target the central nervous system and are mainly composed of small proteins including three-finger toxins (3FTs), snake venom phospholipases A_2 group I (SV-G^I-PLA_2) or dendrotoxins, and are usually associated with elapid snakes (Ferraz et al., 2019). Conversely, hemotoxic and cytotoxic venoms generally are comprised of large enzymatic proteins and protein complexes, including snake venom metalloproteases (SVMP), serine proteases (SP) or snake venom phospholipases A_2 group II (SV-G^{II}-PLA_2), and are typically associated with viperid snakes (Fry, 2015; Fry et al., 2008; Tasoulis & Isbister, 2017). While these historical classifications have proven to be somewhat useful for treating envenomations medically, recent studies have revealed that the presence of these toxins are not exclusive to specific snake families (Osipov & Utkin, 2023).

Vipers (family Viperidae) are a monophyletic lineage of venomous snakes found across Eurasia, Africa and America (Vitt & Caldwell, 2014), receiving extensive research attention primarily due to their medical relevance (Arnold et al., 2009; Casewell et al., 2009; Pook et al., 2009; Šmíd & Tolley, 2019; Wüster et al., 2008). The majority of venom studies in this group have primarily been ~~conducted~~ investigated using a proteomic approach, with early venom work being highly motivated by the medical field, with a limited number of studies employing genomic approaches (but see Almeida et al., (2021); Margres et al., (2021a); Myers et al., (2022); Schield et al., (2019); Hirst et al., (~~in review~~2024); Hogan et al., (2024)). Sequencing efforts to obtain high-quality reference genomes have mainly focused on pitvipers (Crotalinae subfamily, 11 reference genomes, NCBI last accessed 13 March 2024), especially within the *Crotalus* (*n*=6) genus, and have focused on the study of venom evolution (Gilbert et al., 2014; Hogan et al., 2024~~1~~; Margres et al., 2021a; Schield et al., 2019; Westeen et al.,

2023). Other viperids have also been sequenced (although in lower numbers) from both Azemiopinae and Viperinae subfamilies (one and four, respectively) (Myers et al., 2022; Saethang et al., 2022; Talavera et al., in review). Currently, reference genomes are only available for~~a total of~~ 16 viper species ~~within the Viperidae family posses an available reference genome at NCBI, corresponding to 3.6% of the~~ out of the total 387 total species via the NCBI genomic database (Uetz, 2021). Vipers display extensive variation in venom composition between and within genera (Ali et al., 2015; Mackessy, 2010) and even intraspecifically (Jan et al., 2002; Zancolli et al., 2019). Such differences are most likely due to the high diversity of venom genes and their different effects on prey but also, at least i~~o~~n some ~~occasions~~cases, the result of introgression with related species (Jan et al., 2002; Margres et al., 2021b; Smith et al., 2023). This provides an extraordinary opportunity to study trait evolution both at ~~the~~ inter- and intraspecific levels.

Native to the Arabian Peninsula, the Arabian horned viper (*Cerastes gasperettii*, family Viperidae) is a venomous snake currently recognized within the highest medical importance category (WHO; accessed July, 2024). Extending from the Sinai Peninsula to southwestern Iran in the north and reaching as far as Yemen and Oman in the south, its distribution is widespread (Fig. S1). Found mainly in sandy habitats, this arid-adapted ground-dwelling snake with generalist requirements (Carranza et al., 2021; Mochales-Riaño et al., 2024; Russell & Campbell, 2015) is one of the most common venomous snakes found in Arabia and is responsible for occasional snakebite envenomations (Al-Sadoon & Paray, 2016; Amr et al., 2020; Schneemann et al., 2004).

In this study, we present a high-quality chromosome-level reference genome assembly for the Arabian horned viper (*Cerastes gasperettii*, NCBI: txid110202), being one of the first within the Viperinae subfamily. Our highly-contiguous genome showcases a high level of similarity at the chromosome level within the Viperidae family with some minor rearrangements with elapids. Moreover, combining~~employing~~ genomics, transcriptomics, and proteomics~~several -omics techniques~~, we characterized the main toxins found in its venom and the location of those toxins in the genome, comparing their evolutionary history and gene copy number variation with other venomous species. We deciphered ~~numerous genomic attributes of this species including its genetic diversity~~its adequate levels of genetic diversity ~~and failed to find evidence of inbreeding~~. Finally, we reconstructed the demographic

history for the species, revealing how historical increases in aridity likely drove population expansions. Overall, the genomic resources generated in this study provide an essential reference resource for forthcoming studies on venom evolution.

Methods

Sampling

Three adult specimens (two females and one male) of *Cerastes gasperettii gasperettii* were used for this study (Table S1). Blood was extracted only from a single female individual (the heterogametic sex, sample CG1) to obtain High-molecular-weight (HMW) genomic DNA (gDNA). We anesthesized the individual, extracted blood from the heart and stored in ethanol and EDTA. For each of the three individuals, we extracted twelve different tissues, including the venom gland, which was stored in RNAlater™ until RNA extraction (Table S1 and Fig. S2). Before dissections, venom was extracted and snakes were allowed to recover for four days to maximize the venom gland transcription. We only extracted the left venom gland per individual, as previous research within the same family has shown that both venom glands provide indistinguishable results (Rokyta et al., 2017).

DNA extraction, library preparation and sequencing

We extracted gDNA from the blood of a female individual (CG1 in Table S1) using the MagAttract HMW Kit (Qiagen) following manufacturer's protocols without modifications. Then, we sequenced a total of two 8M SMRT HiFi cells in a Sequel II PacBio machine, aiming for a ~30x of coverage, at the University of Leiden. Hi-C libraries were prepared using the Omni-C kit (Dovetail Genomics), following the manufacturer's protocol and using blood stored in EDTA, at the National Center for Genomic Analyses (CNAG), in Barcelona, Spain. The library was paired-end sequenced on a NovaSeq 6000 (2 × 150 bp) following the manufacturer's protocol for dual indexing and aiming for a coverage of ~60x. Finally, we sequenced short-read whole-genome data of the same individual using a NEB Ultra II FS DNA kit; the library was paired-end sequenced on a NovaSeq 6000 (2 × 150 bp) at the Core sequencing platform from the New York University of Abu Dhabi, aiming for ~70x depth of coverage.

RNA extraction, library preparation and sequencing

We extracted RNA from the same three individuals described above (Table S1 and Fig. S2). RNA was isolated using the HighPurity™ Total RNA Extraction Kit (Canvax, Valladolid, Spain). We selected a total of 35 samples (~~including venom glands, tongue, liver and pancreas, among others;~~ Table S2). RNA libraries were prepared with the VAHTS Universal V8 RNA-seq Library Prep Kit, being strand-specific and were sequenced on a NovaSeq 6000 (2 × 150 bp) aiming for an average of 40M read pairs per sample (Table S2), but we first sequenced the reference individual and later on the other two samples. Moreover, we sequenced one 8M SMRT HiFi cell on a Sequel II PacBio machine containing two Iso-seq HiFi libraries at University of Leiden: one containing only the venom gland, and the second library being a pool of eight high-quality tissues (brain, kidney, liver, gallbladder, spleen, tongue, pancreas and ovary).

Genome assembly and scaffolding

Quality control of~~n~~ HiFi and Illumina reads was ~~assessed~~ performed using FastQC v0.12.1 (Andrews, 2010) and adapters were removed with cutadapt v4.9 (Martin, 2011). In order to initially explore the genome size, heterozygosity levels and coverage data, ~~To make an initial exploration of the genome, using the raw HiFi reads,~~ we generated a k-mer profile with Meryl v1.4.1 (Rhie, Walenz, et al., 2020), using the raw HiFi reads and default parameters, and visualized it with GenomeScope2 v2.0.1 (Ranallo-Benavidez et al., 2020). Then, we assembled the genome following the VGP assembly pipeline v2.0 (Rhie~~, McCarthy,~~ et al., 2020). PacBio HiFi reads were assembled into contigs using the software Hifiasm v0.21.0 (Cheng et al., 2021), producing primary and alternate assemblies. We used *purge_dups* (Guan et al., 2020) to remove haplotypic duplicates from the primary assembly and added them to the alternate assembly. Then, we scaffolded the resulting haplotypic ~~primary~~ assembly using the Hi-C data with SALSA2 v1 (Ghurye et al., 2019), with default parameters. Following the VGP assembly pipeline (Rhie et al., 2020), m~~M~~anual curation was performed with ~~Pretext~~Pretext v0.2.5 (https://pipelines.tol.sanger.ac.uk/curationpretext). Breaks were not mannualy created and we joint contings on gaps previously identified by SALSA2. We used the ~78x Illumina data to polish the assembly with one round of Pilon v1.24 (Walker et al., 2014). The mitochondrial genome was obtained

Formatted: No underline

Formatted: No underline

Formatted: No underline

Field Code Changed

Formatted: Font color: Blue

Formatted: No underline

with GetOrganelle v1.7.7.1 (Jin et al., 2020), using the available mitochondrial genome of several *Echis* species (*E. coloratus*, *E. carinatus* and *E. omanensis*) to seed the assembly (NCBI accession numbers: SRX18902082, SRX18902083, SRX18902084, respectively).

Genome assembly quality evaluation

Quality assessment and general metrics for the final assembly were estimated with both QUAST v.5.1.0 (Gurevich et al., 2013) and gfastats v1.3.8 (Formenti et al., 2022). Possible contaminations were evaluated with BlobToolKit v.4.4.0 (Challis et al., 2020) using the NCBI taxdump database. We also used MitoFinder v.1.4.2 (Allio et al., 2020; ~~D.~~ Li et al., 2016) to confirm that the mitochondrial genome was absent in the assembled nuclear reference genome. Completeness of the genome assembly was assessed with BUSCO v5.3.0. against the sauropsida_odb10 database (*n*=7,480).

Genome annotation

First, we identified repetitive elements using RepeatModeler v.2.0.3 (Flynn et al., 2020) for *de novo* predictions of repeat families. To annotate genome-wide complex repeats, we used RepeatMasker v.4.1.3 (Tempel, 2012) with default settings to identify known Tetrapoda repeats present in the curated Repbase database (Bao et al., 2015). Then, we ran three iterative rounds of RepeatMasker to annotate the known and unknown elements identified by RepeatModeler in order to maximize the known elements at the expense of diminishing the unknown elements. Later, we ~~and~~ soft-masked the genome for simple repeats. We used GeMoMa v.1.9 (Keilwagen et al., 2019) to annotate protein-coding genes, combining both the RNA-seq data generated in this study as described above (already mapped in to our new assembly) as well as annotations from seven other squamate genomes already published: *Anolis carolinensis* ~~from (~~Alföldi et al., ~~(~~2011), *Crotalus adamanteus* ~~from (~~Hogan et al., ~~(~~2024), *Crotalus tigris* ~~from (~~Margres et al., ~~(~~2021a), *Ophiophagus hannah* ~~from (~~Vonk et al., ~~(~~2013), *Naja naja* ~~from (~~Suryamohan et al., (2020), *Crotalus ruber* ~~from (~~Hirst et al., ~~(in review~~2024) and *Crotalus viridis* ~~from (~~Schield et al., ~~(~~2019). We ~~previously~~ quality checked and removed the adapters of the RNA-seq data using fastp v0.23.3 (Chen et al., 2018), as well as mapped the transcriptomic data to our new reference genome with Hisat2 v2.2.1 (Kim et al., 2019). Additionally, we also removed the adapters for the Iso-seq data with fastp v0.23.3 (Chen et al., 2018) and mapped the long-read transcriptomic data to our new

Formatted: Font: Not Bold

reference genome with pbmm2, collapsing mapped reads into unique isoforms with isoseq3 and annotated with GeneMarkS-T v5.1 (S. Tang et al., 2015). We combined both annotations (GeMoMa and GeneMarkS-T) with TSEBRA (Gabriel et al., 2021). We blast BLASTp our predicted proteins to a Uniprot protein database for a total of ten species (*C. gasperettii*, *C. vipera*, *C. cerastes*, *Anolis carolinensis*, *Crotalus viridis*, *Crotalus tigris*, *Crotalus ruber*, *Crotalus adamanteus*, *Ophiophagus hannah* and *Naja naja*). Simultaneously, we ran Interproscan v5.72 (Jones et al., 2014) on our predicted proteins. Then, we combined both functional annotations with AGAT v1.4.1 (Dainat et al., 2023). Finally, as toxin-coding genevenom gene families are known to occur in large tandem arrays and the number of paralogs can be underestimated in particular gene families (Schield et al., 2019), we performed additional annotation steps for venom toxin genes:. Following Margres et al., (2021a), we used a combination of empirical annotation in FGENESH+ (Solovyev et al., 2006), as well as manual annotation using RNA-seq and Iso-seq alignments; the former identified all genes regardless of expression, whereas the latter was used to explicitly identify expressed toxins.

Macrosynteny Chromosome-level analyses

Whole genomeChromosomal synteny was explored between our new chromosome-level reference genome for the Arabian horned viper together with the Eastern diamondback rattlesnake (*Crotalus adamanteus*) (Hogan et al., 2024), the Indian cobra (*Naja naja*) (Suryamohan et al., 2020) and the Brown anole (*Anolis sagrei*) (Geneva et al., 2022) using Mcscan v1.4.23 (H. Tang et al., 2008). Protein sequences from each of the three venomous snakes were extracted using AGAT (v1.2.1) (Dainat et al., 2023) and were pairwise aligned with LAST (Kiełbasa et al., 2011), implemented in the JCVI python module (Tang et al., 2017). A first alignment was used between the three species to identify chromosomes assembled in the reverse complement, which were corrected using SAMtools faidx (v1.18.1) (Danecek et al., 2021) using both options reverse-complement and mark-strand. Gene annotations for the new reference (with the corresponding reversed chromosomes) were annotated using GeMoMa v.1.9 (Keilwagen et al., 2019), and MCscan was rerun. The last four scaffolds (14, 15, 16 and 17) from *Anolis sagrei* were removed, as no orthologous groups were found.

Transcriptomics

After adapter trimming and quality control using fastp v0.23.3 (Chen et al., 2018), we mapped our RNA-seq reads to the reference genome of *Cerastes gasperettii* using Hisat2 v2.2.1 (Kim et al., 2019). Gene expression raw counts per gene across all samples were calculated with StringTie (Pertea et al., 2015). Initial exploration of our transcriptomic data revealed a clear batch effect for one of the three samples (Fig. S4), due to the low mapping of that sample to our reference genome. Therefore, we decided to remove individual CG1 from future RNA-seq analyses. Moreover, to avoid pseudoreplication, we also removed the accessory gland from individual CG009 due to its high similarity with the venom gland, suggesting that the venom gland rather than the accessory gland was sampled (Fig. S4). Differential expression analyses were carried out with the DESeq2 package v.1.42.0 (Love et al., 2014) from R v4.4.2 (R Core Team, 2021)~~,~~. Prior to analysis, genes with fewer than 10 counts across all samples were filtered out. For comparisons, we defined two groups: venom glands versus all other tissues. DESeq2 employs a negative binomial generalized linear model to estimate differences in gene expression, and the p-values were adjusted for multiple testing using the Benjamini-Hochberg method to control the false discovery rate (FDR). Genes with an adjusted p-value < 0.01 and a fold change > 2 were considered significantly differentially expressed. ~~using the DESeq2 median of ratios normalization.~~ Finally, we identified the highly expressed genes found in the venom gland as well as the toxins uniquely expressed in the venom gland (following Suryamohan et al., (2020)) which were defined as (1) genes expressed in the venom gland (TPM > 500), (2) Differential Upregulated Genes (DUGs) with Fold Change (FC) > 2 comparing venom glands to all other tissues and (3) unique to venom glands (TPM < 500 in all other tissues).

Proteomics

A bottom-up mass spectrometry strategy (Calvete et al., 2021) was used to characterize the venom ~~arsenal~~ of *Cerastes gasperettii*. Briefly, the venom proteome (pool from individuals CN6134 and CN6135, both from United Arab Emirates (UAE); Table S1) was submitted to reverse-phase High-performance liquid chromatography (HPLC) decomplexation followed by SDS-PAGE analysis in 12% polyacrylamide gels run under non-reducing and reducing conditions. Protein bands were excised from Coomassie Brilliant Blue-stained gels and subjected to automated in-gel reduction and alkylation on a

Formatted: Font: 11 pt

Formatted: Font: 11 pt

Formatted: Font color: Black

Genomics Solution ProGest™ Protein Digestion Workstation. Tryptic digests were submitted to MS/MS analysis on a nano-Acquity UltraPerformance LC® (UPLC®) equipped with a BEH130 C_{18} (100µm x 100mm, 1.7 µm particle size) column in-line with a Waters SYNAPT G2 High Definition mass spectrometer. Doubly and triply charged ions were selected for CID-MS/MS. Fragmentation spectra were matched against a customized database including the bony vertebrates taxonomy dataset of the NCBI non-redundant database (release 258 of October 15, 2023) plus the species-specific venom gland transcriptomic and genomic protein sequences gathered in this work. Search parameters were as follows: enzyme trypsin (two-missed cleavage allowed); MS/MS mass tolerance for monoisotopic ions: ± 0.6 Da; carbamidomethyl cysteine and oxidation of methionine were selected as fixed and variable modifications, respectively. Assignments with significance protein score threshold of $p < 0.05$ (Mascot Score > 43) were taken into consideration, and all associated peptide ion hits were manually validated. Unmatched MS/MS spectra were *de novo* sequenced and manually matched to homologous snake toxins venom proteins available in the NCBI non-redundant protein sequences database using the default parameters of the BLASTP program (https://blast.ncbi.nlm.nih.gov/Blast.cgi).

Local synteny analyses Microsynteny

To explore toxin genomic organization across (sub)families, we used blastBLASTn, incorporating both toxin and non-toxin paralogs to identify the genomic location of SVMPs, SVSPs and PLA_2 toxin families, across the genome of *Cerastes gasperettii*, *Crotalus adamanteus*, *N. naja* and *A. feae*. We excluded *A. feae* for SVSPs and SVMPs local synteny microsynteny analyses as those families were not assembled onto a single contig in the *A. feae* genome. Then, we aligned those regions using Mafft (Katoh & Standley, 2013): For SVMPs in CHR8:16.506.135 to CHR8:17.374.029, for SVSPs in CHR9: 17.531.416 to CHR9:17.788.049 and for PLA_2 in CHR17:7.882.542 to CHR17:7.916.827. Each species was annotated within the MSA using its own annotation as a reference in Geneious Prime 2023.0.4. Results were plotted using the gggenomes package (https://github.com/thackl/gggenomes) from R v4.4.2 (R Core Team, 2021).

Toxin phylogenies

Formatted: Subscript

Formatted: English (United States)

We used phylogenetic inference to study the evolutionary history for the main groups of toxins (i.e., SVMPs and SVSPs), which were the most abundant in the proteome of *Cerastes gasperettii*, as well as PLA_2 as this family has been widely studied within the Viperidae family (Dowell et al., 2016; Myers et al., 2022). For the three main toxin families, we selected available toxin genes as well as non-toxin paralogous genes from venomous species; we also included other non-toxin paralogous genes from non-toxic species (for details about this see Supplementary ~~Information~~datasets for the three main toxins). When ~~needed~~nuclear sequences were obtained, we translated CDS to protein sequence, and then protein sequences were aligned with Mafft v7 (Katoh & Standley, 2013). Following Giorgianni et al., (2020), we built a phylogeny for each of the toxin groups with the translated CDS sequences, as explained above, using Phyml v3.3 (Guindon et al., 2010), implementing the Dayhoff substitution model and validating our inferred tree with aBayes support.

Demographic history

We inferred the demographic history of *Cerastes gasperettii* by implementing the Pairwise Sequential Markovian Coalescent (PSMC v0.6.5) software (~~H.~~ Li & Durbin, 2011) on the short-read whole-genome data. Heterozygous positions were obtained from bam files with Samtools v1.9 mpileup function (H. Li et al., 2009), and data were filtered for low mapping (<30) and base quality (<30). Minimum and maximum depths were set at a third (27x) and twice (156x) the average coverage. Only autosomal chromosomes were considered. We used the squamate mutation rate of $2.4x10^{-9}$ substitutions/site/generation and a generation time of 3 years, following Green et al., (2014) and Schield et al., (2022), respectively. A total of ten bootstraps were calculated, plotting the final results with the psmc_plot.pl function from PSMC (https://github.com/lh3/psmc).

Genomic diversity

We downloaded Illumina data for *Bothrops jararaca* (SRR13839751 from Almeida et al., (2021)), *Crotalus viridis* (SRR19221440 from Schield et al., (2019)), *Naja kaouthia* (SRR-8224383; Thongchum et al., (2019)), *Naja naja* (SRR10428156; Suryamohan et al., (2020)) and *Sistrurus tergeminus* (SRR12802282; Bylsma et al., (2022)). Then, we filtered for quality (Phred score of 30) and removed adapters with fastp v0.23.3 (Chen et al., 2018). Trimming of poly-G/X tails and correction in overlapped

regions were specified. All other parameters were set as default. Filtered sequences were visually explored with FastQC v0.12.1 (Andrews, 2010) to ensure data quality and absence of adapters. *Cerastes gasperettii* f~~F~~iltered reads were mapped against the new reference genome of *Cerastes gasperettii* using the bwa mem algorithm v0.7.17 (~~H.~~ Li, 2013). *B. jararaca*, *C. viridis* and *S. tergeminus* were mapped against the *C. viridis* (Schield et al., 2019) reference genome and *N. naja* and *N. kaouthia* were mapped against the *N. naja* reference genome (Suryamohan et al., 2020). Mapped reads were sorted with Samtools v1.9 (H. Li et al., 2009) and duplicated reads were marked and removed with PicardTools v2.28.0 (Broad Institute, 2021). Reads with mapping quality lower than 30 were discarded. SNP calling was carried out with HaplotypeCaller from GATK v.4.1.3.0 (McKenna et al., 2010), with BP_resolution and split by chromosome. For each chromosome, individual genotypes were joined using CombineGVCFs with convert-to-base-pair-resolution, and the GenotypeGVCFs tool was then applied to include non-variant sites. Finally, for each individual, the whole dataset split by chromosome was concatenated with bcftools concat (Danecek et al., 2021), keeping only the autosomes. Then, for each sample, we used the raw dataset to calculate average genome heterozygosity. We generated non-overlapping sliding windows for each of the ~~of 100 Kbp for the newly assembled *Cerastes gasperettii*~~ reference genomes and included only sites (both variant and invariant) with site quality higher than 30 (QUAL field in a VCF file from GATK). Only windows containing more than 60,000 unfiltered sites were considered. Visualization was carried out with ggplot2 (Wickham, 2016) in R v4.4.2 (R Core Team, 2021).

Results and Discussion

Genome assembly and annotation

We generated a high-quality chromosome-level assembly for the Arabian horned viper (*Cerastes gasperettii*) by combining PacBio HiFi (65 Gbp of data~~~40x~~), Hi-C (~~~60x~~96 Gbp of data) and Illumina data (~~~78x~~135 Gbp of data) (Fig. 1 and Fig. S3). First, we *de novo* assembled the HiFi reads into 1,018 contigs (N50=45.7 Mbp; longest contig of 149.99 Mbp). Then, using the proximity ligation data (i.e., Hi-C), we scaffolded the genome into 319 scaffolds (N50=111.38 Mbp; largest scaffold 345.38 Mbp). After manual curation, ~~we enhanced the~~ the ~~-~~scaffolding parameters of our genome were improved

Formatted: Font: Italic

(N50=214.14 Mbp; largest scaffold 361.99 Mbp), containing 99.44% of the genome present in 19 scaffolds or pseudochromosomes (7 macro-, 10 micro-, Z and W sex chromosomes; Table 1 and Fig. 1B). The total genome length was 1.63 Gb, similar to other venomous snakes (Margres et al., 2021a; Schield et al., 2019; Suryamohan et al., 2020; Vonk et al., 2013; Table 1), with a contig N50 of 45.6 Mbp, ~3.3 times more contiguous than the *N. naja* genome (Suryamohan et al., 2020), ~228 times more contiguous than the *Anolis sagrei* genome (Geneva et al., 2022), but 0.67 times less contiguous than the recently published *Crotalus adamanteus* genome (Hogan et al., 2024), making it one of the most contiguous chromosomal squamate genomes assembled to date (Table 1). We assessed the completeness of the assembly using BUSCO (Simão et al., 2015) with the sauropsida gene set (*n*=7,480). Upon evaluation, we successfully identified 92.8% of the genes (91.4% single-copy, 1.4% duplicated), while the remaining genes were fragmented (1%) or missing (6.2%; Fig. 1). For the *de novo* assembly, GC content and repeat content were 37.87% and 43.63%, respectively. The repetitive landscape was dominated by retroelements (30.25%), with a majority of LINEs (21.25%) (Table S3). Finally, we annotated 27,158 different protein-coding genes within our assembly, with a total of 194 putative toxins or toxin-paralogs genes. Toxin genes were found in both ~~usually found in venomous snakes (see proteome results below) were mainly found on~~ macro- and microchromosomes~~, although major toxin groups were found on microchromosomes (SVMPs, SVSPs and PLA$_2$; (~~Fig. 1), and were found onto individual contigs. ~~.~~ Finally, we also found a battery of 3FTxs and myotoxin-like genes, but they were not represented in our proteome and RNA-seq dataset (see below).

~~Macrosynteny~~Genomic architecture highly conserved among vipers

Whole-genome synteny comparisons showed ~~a great level of~~ similarity between *Cerastes gasperettii* and *Crotalus adamanteus*, with large syntenic blocks both within macro- and microchromosomes (Fig. 2). Some chromosomal rearrangements were observed between viperids and elapids, as previously discussed by Suryamohan et al., (2020), with a fission of chromosome four in *N. naja* to form chromosomes five and seven in vipers, and a fusion of chromosomes five and six in *N. naja* to form chromosome four in vipers. Interestingly, several chromosomal rearrangements between lizards and snakes have occurred, as we found several fission events in the *A. sagrei* genome, including one fission

from chromosome two to originate the current Z chromosome in snakes (Fig. 2). ~~The last four scaffolds (14, 15, 16 and 17) from *Anolis sagrei* were removed, as no orthologous groups were found.~~ ~~Macrosyntenic differences between lizards and snakes could be related to the innovations in different areas such as locomotion, feeding and sensory processing that snakes experienced during their origin more than 150 Mya (Title et al., 2024) as well as explain the high level of chromosomal similarity within snakes.~~

~~Transcriptomics~~Toxins uniquely expressed in the venom glands

Our analyses of multi-tissue transcriptomic data (23 samples from two individuals covering 13 different tissues) reported a total of 23,178 expressed genes (TPM > 1). Heatmap ~~analyses with~~ of the ~~most~~ 2,000 most variable genes reported unique upregulated genes for each of the ~~different~~ analyzed tissues (Fig. S5). The venom gland transcriptome contained a total of 7,237 genes expressed (TPM > 500), including a total of 65 putative toxin genes. From those, we did not detect any 3FTxs and/or myotoxin-like gene transcripts. Differential gene expression analyses revealed a total of 161 genes (33 putative toxin genes) that were differentially upregulated (FC > 2 and 1% FDR) in venom glands compared to other tissues (Fig. 3A and Fig. S6-7). Finally, a total of 10 toxin genes (*CRISP2*, *SVMP9*, *SVMP10*, *SVSP8*, *SVSP7*, *SVSP5*, *CTL14*, *CTL15*, *SVSP4* and *SVMP13*) were uniquely expressed in the venom gland, encoding for the minimal core venom effector (Fig. 3A) (Suryamohan et al., 2020), and in line with the main toxins found within the proteome (Fig. 3B), although some differences were observed(as the absence of PLA_2 within the highly-expressed genes), possibly due to individual venom differences. These 10 genes, together with other SVMPs, SVSPs~~, Disintegrins (DISI)~~ and C-type lectins (CTL), were highly expressed in the venom gland and form the core toxic effector components of the venom. Targeting the core ~~venom~~ toxins together with other well-known modulators of venom may help manufacture of synthetic antivenom treatments as well as improve neutralization tests of current antivenoms (Suryamohan et al., 2020). However, more transcriptomic data should be incorporated to correct for potential ontogenetic and geographical variation in venom composition in *C. gasperettii* (Avella et al., 2022; Kalita et al., 2018).

SVSPs and SVMPs as main toxins~~ proteins~~

Venom proteomics identified Snake venom Serine Proteases (SVSPs) and Snake Venom Metalloproteinases (~~–~~SVMPs) as the most abundant toxin families within the venom of *Cerastes gasperettii*, with 37.38% and 22.19% of the venom being composed by peptides from those two families, respectively (Fig. 3B); the dominance of these two toxin families is consistent with previous research on the same genus (Casewell et al., 2014; Fahmi et al., 2012). Other toxin families identified were DISI (12.74%), CTL (7.25%), PLA_2 (5.47%), Cysteine-Rich Secretory Proteins (CRISP; 4.34%) or L-Amino acid oxidase (LAAO; 1.71%) (Fig. 3B). We did not detect any 3FTx or myotoxin-like peptides within the proteome.

SVMPs

We ~~studied~~ analyzed the evolution of venom of the most~~evolution within the most~~ abundant venom toxin groups (i.e., SVMPs and SVSPs, as well as PLA_2). After a thorough manual curation, we used comparative genomics to evaluate the number and position of those genes in comparison with the Indian cobra (*N. naja*), the Eastern diamondback rattlesnake (*Crotalus adamanteus*), and the Fea's viper (*A. feae*). We reported a total of 13 fully contiguous tandem ~~repeat~~ array SVMPs for *Cerastes gasperettii* (Fig. 4A), next to the non-toxic paralogous gene *ADAM28* and flanked by the *NEFL* and *NEFM* non-toxic genes. Microsyntenic analyses showed gene copy number variation between the studied species (Fig. 4A). Overall, we can see an expansion in the number of SVMPs within the Viperidae family, particularly in *Crotalus adamanteus* (22 copies unique to vipers and 10 lineage-specific copies) but also in *Cerastes gasperettii* (12 copies unique to vipers and one lineage-specific copy) (Fig. 4A). ~~The amplification of SVMP copy numbers is consistent with our proteomic results, as SVMPs were the second most abundant component of the venom (Fig. 3B).~~ Then, we reconstructed the evolutionary history of this toxin family (Fig. 4B and 8~~Fig. S6~~). Phylogenetic analyses for this toxin group reported a highly supported clade comprising *ADAM28* peptides, the non-toxic paralogous gene. The second clade of orthologous toxin-peptides were found within both elapid and viperid families (including species from Crotalinae and Viperinae subfamilies in viperids; Fig. S8~~6~~) as well as two SVMPs from *A. feae*. Interestingly, we report a new toxin-coding gene within *Cerastes gasperettii* with a different evolutionary history, as it did not share orthology with any other gene (Fig. 4B). This new gene likely

Formatted: Font: 11 pt

arose from a duplication event of *SVMP13*, within the group of SVMP *MDC1* toxins (Fig. S86). Our discovery of a novel SVMP gene in *C. gasperettii* adds to the growing body of work on the dynamic evolution of venom systems. Similar gene expansions and duplications have been observed in other species, such as PLA$_2$ toxin-coding genes found in the venom of *Azemiops feae* (Myers et al., 2022), highligting the lineage-specific nature of venom evolution. The gene we identified, possibly arising from an *SVMP13* duplication, do not share orthology with genes in other species, suggesting the presence of hidden toxin diversity in venom systems. This discovery highlights the importance of using genomics in studying venom evolution, as this putatively toxic gene was not found to be differentially upregulated in the venom gland or recovered in the proteome (Fig. 3). More genomic data will indicate if *SVMP12* is unique for the Viperinae subfamily, the *Cerastes* genus or if it is only found in *Cerastes gasperettii*. All other clades were unique to viperids (and some exclusive only to crotalids), except for a clade composed by SVMPs unique to elapids, as previously discussed in Suryamohan et al., (2020). Interestingly, one of the toxins (*SVMP8*) was not a class P-III SVMP, as it clusters within the MAD-4/5 clade (class P-II SVMP), contrary to the proteomic results where all SVMPs were categorized within the class P-III (Fig. 3B). Although there has been a clear expansion of the SVMP family within the *Crotalus* genus, our results suggest that the origin of that expansion was at the beginning of the Viperidae family, as most of the groups are also present within the Viperinae subfamily.

PLA$_2$

Regarding PLA$_2$, we report two tandem repeat venomous genes for *Cerastes gasperettii* within the non-toxic *PLA$_2$-g2E* and *PLA$_2$-g2F* array (Fig. 4C), flanked by *OTUD3* and *MUL1* non-toxic genes, as previously reported in other species (Dowell et al., 2016; Margres et al., 2021a; Myers et al., 2022). The number of venomous PLA$_2$ in *Cerastes gasperettii* was lower than in *A. feae* and *Crotalus adamanteus*. This difference may be expected, as PLA$_2$ only represents around 5% of the proteome for *Cerastes gasperettii* (Fig. 3B) whilst PLA2 are abundant toxins in the proteome for the other two species (Margres et al., 2014; Myers et al., 2022). Phylogenetic results for PLA$_2$ genes showed a fully supported clade containing both non-toxic *PLA$_2$-g2E* and *PLA$_2$-g2F* as outgroups (Fig. 4D and Fig. S97). We also found all other PLA$_2$ groups reported in previous studies: *PLA$_2$-gC*, *PLA$_2$-gK*, *PLA$_2$-gB*, *PLA$_2$-gD* and

Formatted: Subscript

Formatted: Font: Italic

Formatted: Font color: Black

PLA_2-gA (Dowell et al., 2016; Myers et al., 2022). The two genes for our target species clustered in different groups (Fig. 4D and Fig. S9~~7~~). The first PLA_2 was a *PLA_2-gD*, which is a group of PLA_2s exclusively found in true vipers (subfamily Viperinae). The second one was a *PLA_2-gC* which is more ancestral as it is also found in other pitvipers and non-venomous snakes such as pythons (Dowell et al., 2016). The genomic results are consistent with the proteomics, indicating that specific duplications of PLA_2 toxin-coding genes have not occurred in *Cerastes gasperettii*.

SVSPs

~~Finally, w~~We found eight different SVSPs within the genome of *Cerastes gasperettii*, flanked by *RBM42* and *GRAMD1A* non-toxic genes (Fig. 4E). For this toxin family, we were only able to compare the results with *Crotalus adamanteus*. We were unable to confidently ~~did not~~ determine ~~with enough confidence~~ the location of SVSPs ~~with~~in the *N. naja* genome (several regions were matching our venomous SVSP genes as well as the flanking genes). Moreover, *A. feae* was also ~~discarded~~ not compared as SVSPs were not assembled in a single contig. ~~The high number of SVSP genes found (although lower than in *Crotalus adamanteus*) were in line with the proteomic results, as SVSPs are the most abundant toxin in the proteome (Fig. 3B)~~. Phylogenetic results showed three clades, with two ~~of them~~ containing *Cerastes gasperettii* genes (Fig. 4F and Fig. S10~~8~~). Group 1 was mainly present within *Crotalus*, although there was the presence of some true vipers species, but not in *Cerastes gasperettii* (Fig. S10~~8~~). Group 2 contained six genes within *Crotalus adamanteus* and only two for *Cerastes gasperettii*. Interestingly, Group 3 was expanded in *Cerastes gasperettii* (Fig. 4E) with a total of six copies, while four were found within *Crotalus adamanteus*. Most of the toxins ~~peptides~~ included in the analyses for true vipers were also found in Group 3 (Fig. S10~~8~~), indicating a possible expansion of this group of toxins in true vipers (or gene losses in pit vipers). Overall, our high-quality chromosome level reference genome has shed light on the evolution of the main toxin-coding gene families, indicating a compelling correlation between the abundance of toxin-coding genes and the prevalence of these toxins in the venom of *Cerastes gasperettii*.

~~Genomic diversity and ancient demographic history~~Glacial periods drove population expasions in *C. gasperettii*

Formatted: Font: Italic

The Arabian horned viper (*C. gasperettii*) is a widespread species, categorized as Least Concern by the IUCN (Egan et al., 2012). Genome-wide diversity was in line with its conservation status, as it showed similar heterozygosity levels compared to other venomous snakes (Fig. 5A). However, more individuals should be sampled along its distribution to verify that similar heterozygosity levels are found across its range. PSMC analyses showed several population expansions and contractions in the last 400 kya, whilst the effective population size of *Cerastes gasperettii* remained relatively constant from 1 until 10 Mya (Fig. 5B). Interestingly, population expansions were coincident with the Last glacial and Penultimate glacial periods (grey lines on Fig. 5B), with a large population increase during the Penultimate Glacial Period (PGP) (1.94~~x10^{5}~~ to 1.35~~x10^{5}~~ mya) (Fig. 5B). In fact, during glacial periods, global sea level dropped around 150 meters, exposing the floor and the sand to the wind, which promoted aridification in the Arabian Peninsula and potentially increased habitat suitability for the species (Burriel-Carranza et al., 2023; Glennie & Singhvi, 2002). PSMC results may vary depending on the generation time as well as the mutational rate specified. The absence of species-specific data for this analyses may bias our results, although it is a general consensus in the literature when inferring these demographyc analyses in snakes (e.g. Schield et al., 2019).

Conclusions

Our high-quality chromosome-level reference genome for *C. gasperettii* showed that chromosomal architecture is highly conserved between Crotalinae and Viperinae subfamilies, and differs from elapid genomes by a small number of chromosomal rearrangements. We also found the genomic coordinates of the main toxin-encoding genes, highlighting gene duplication as the main driver in the evolution of SVMP and SVSP toxins. We identified a new SVMP toxin-coding gene, showcasing the importance of using high-quality reference genomes (combined with other -omic techniques) for thoroughly characterizing toxin-encoding genes. Finally, this is a new and important resource for a large clade with ~~currently~~ few reference genomes available. Future genomic studies focusing on Old World viper evolution will benefit greatly from this resource, which will help unveil the origin and diversification of venom and serve as an essential genomic tool for further venomic studies on the subfamily Viperinae.

Acknowledgements

GM-R is supported by an FPI grant from the Ministerio de Ciencia, Innovación y Universidades, Spain (PRE2019-088729), SRH is awarded by the National Science Foundation Graduate Research Fellowship Program with grant no. 2136515, AT is supported by "la Caixa" doctoral fellowship program (LCF/BQ/DR20/11790007), BB-C is supported by FPU grant from Ministerio de Ciencia, Innovación y Universidades, Spain (FPU18/04742) and ME is supported by an FPI grant from Ministerio de Ciencia e Innovación (PRE2022-101473). In the UAE, we wish to thank His Highness Sheikh Dr. Sultan bin Mohammed Al Qasimi, Supreme Council Member and Ruler of Sharjah, H. E. Ms. Hana Saif al Suwaidi (Chairperson of the Environment and Protected Areas Authority, Sharjah) for their continuous support. Some of this research was carried out on the High Performance Computing resources at New York University Abu Dhabi. We thank Jonathan Wood and Klara Eleftheriadi for their input during the genome assembly and manual curation processes. We also thank Valéria Marques for her help in building the figures and Prem Aguilar for reviewing a previous version of the manuscript.

Data availability

Final assembly and raw reads files were deposited in NCBI under bioproject No. PRJNA1068073.

Funding

This work was funded by grant PID2021-128901NB-I00 (MCIN/AEI/10.13039/501100011033 and by ERDF, A way of making Europe; Spain) and grant 2021-SGR-00751 from the Departament de Recerca i Universitats from the Generalitat de Catalunya, Spain to SC.

Competing Interests

The authors declare that they have no competing interests.

Author's contribution

Conceptualization: G.M.R., A.T., B.B.C., J.C., J.E., M.M., S.C. Investigation: S.H, V.P., M.E., T.B., S.B., M.H., J.T.G., D.P., J.C., M.M. Funding acquisition: S.C. Writing-original

draft: G.M.R. Writing-review & editing: All authors read, revised, and approved the manuscript final version.

Ethics statement

No in vivo experiments were performed. Specimens were collected and manipulated with the authorization and under strict control and permission of the government of the United Arab Emirates (Environment and Protected Areas Authority, Government of Sharjah), who approved the study. Specimens were captured and processed following the guidelines and protocols stated in the agreements obtained from the competent authority of the United Arab Emirates. Members of the government supervised collecting activities. All efforts were made to minimize animal suffering. All the research in the United Arab Emirates was done under the supervision and permission of the Environment and Protected Areas Authority, Government of Sharjah.

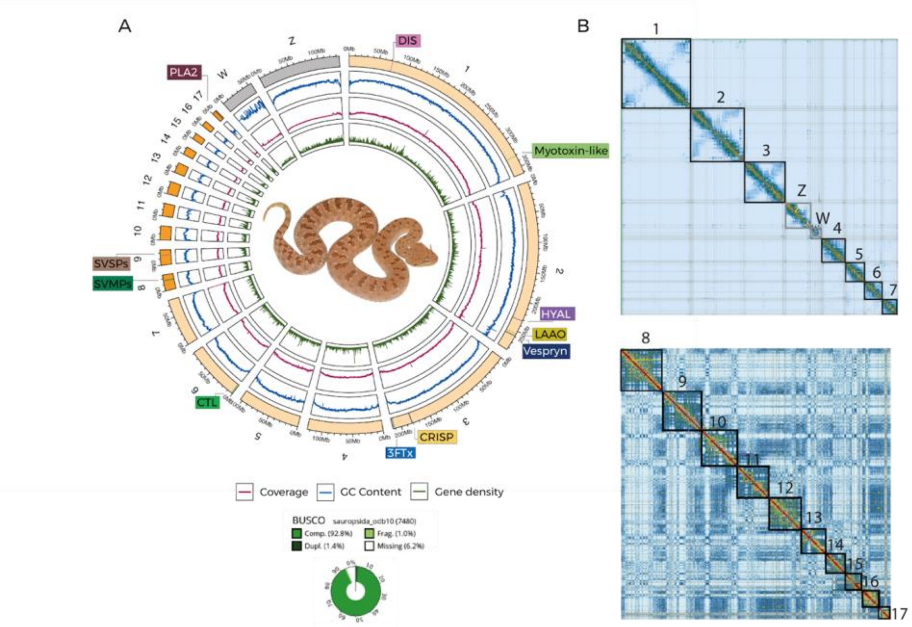
A
B
PLA2
DIS
Myotoxin-like
HYAL
LAAO
Vespryn
CRISP
3FTx
CTL
SVMPs
SVSPs
Coverage
GC Content
Gene density
BUSCO sauropsida_odb10 (7480)
Comp. (92.8%)
Frag. (1.0%)
Dupl. (1.4%)
Missing (6.2%)

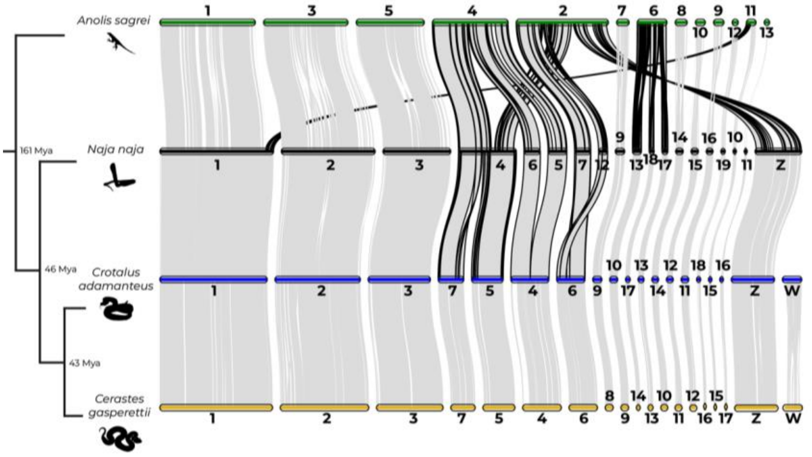
Anolis sagrei
Naja naja
Crotalus adamanteus
Cerastes gasperettii
161 Mya
46 Mya
43 Mya

Fig. 1: A) Reference genome for *Cerastes gasperettii*, including BUSCO score, GC content, coverage level as well as the main toxins found within the genome. Macrochromosomes are shown in light orange whilst microchromosomes are shown in bright orange. Sex chromosomes are shown in gray. Abreviations are as follows: DIS, Disintegrins; HYAL, Hyaluronidases; LAAO, L-Amino acid oxidase; CRISP, Cysteine-rich secreted proteins; 3FTx, Three-finger toxins; CTL, C-type lectins; SVMPs, Snake venom metalloproteinases; SVSPs, Snake venom serine proteinases; PLA_2, Phospholipases. B) Linkage HiC contact map for the macrochromosomes (above), including the sex chromosomes (Z and W), and microchromosomes (below).

Formatted: English (United Kingdom)

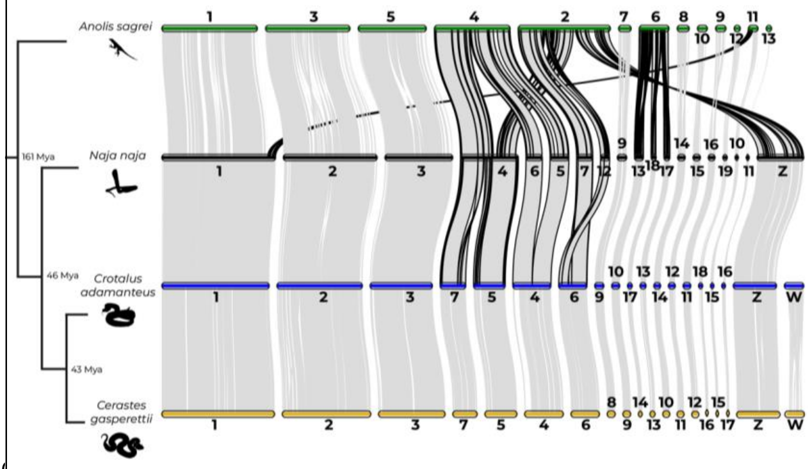

Fig. 2: Macrosynteny Chromosome-level analyses for one Elapidae (*Naja naja*), one Crotalinae (*Crotalus adamanteus*) and one Viperinae (*Cerastes gasperettii*) species, with *Anolis sagrei* as the outgroup. The four smallest scaffolds (14, 15, 16 and 17) of *Anolis sagrei* were removed, as no orthologous groups were found with other species. Borders of regions showing evidence for chromosomal rearrangements are shown in black. Estimates for branch times obtained from TimeTree.org based on divergence times between Iguania and Serpentes, Elapidae and Viperidae and Crotalinae and Viperinae, respectively.

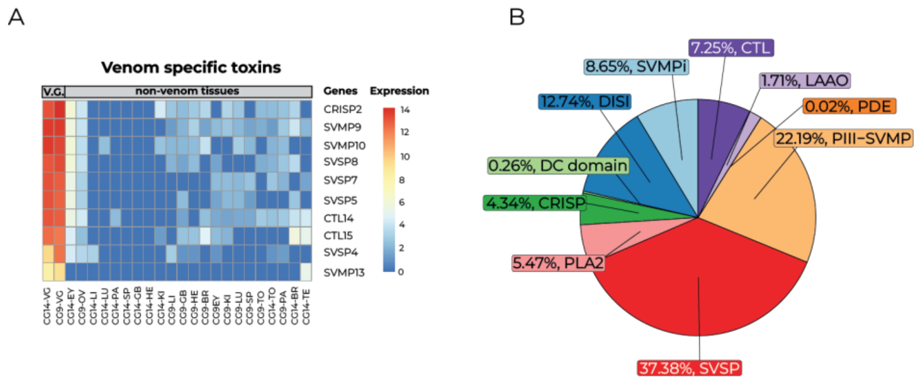

Fig. 3: Main toxins found in both the transcriptome and proteome of *Cerastes gasperettii*. A) Transcriptomic results with gGenes upregulated and exclusively found in the venom gland for both individuals. Each column represents a different tissue type per sample. Rows show the different genes, and colors correspond to different expression levels. Abbreviations are as follows: VG, Venom Gland; EY, Eye; OV, Ovary; LI, Liver; LU, Lung; PA, Pancreas; SP, Spleen; GB, Gallbladder; HE, Heart; KI, Kidney; LI, Liver; BR, Brain; TO, Tongue; TE, Testis. B) Proteomic results of vVenom composition for a pool of one two individuals of *Cerastes gasperettii*. The pie chart displays the relative abundances of the toxin families found in the proteome of the *Cerastes gasperettii* venom. Abbreviations are as follows: SVMP, snake venom metalloproteinase; SVSP, snake venom serine proteases; PLA_2, phospholipases A_2; CRISP, cysteine-rich secretory proteins; DISI, disintegrins; CTL, C-type lectins; LAAO, L-amino-acid oxidases; PDE, phosphodiesterases.

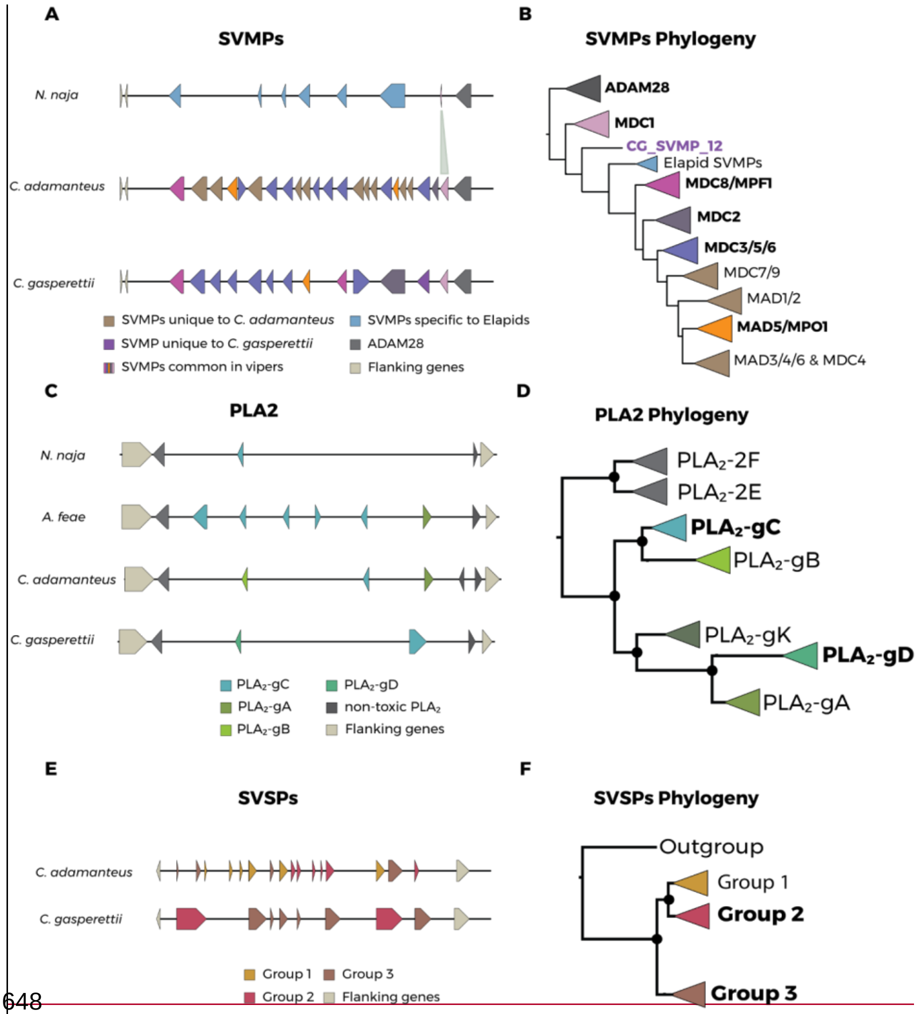
A
SVMPs
N. naja
C. adamanteus
C. gasperettii
SVMPs unique to C. adamanteus
SVMP unique to C. gasperettii
SVMPs common in vipers
SVMPs specific to Elapids
ADAM28
Flanking genes
B
SVMPs Phylogeny
ADAM28
MDC1
CG_SVMP_12
Elapid SVMPs
MDC8/MPF1
MDC2
MDC3/5/6
MDC7/9
MAD1/2
MAD5/MPO1
MAD3/4/6 & MDC4
C
PLA2
N. naja
A. feae
C. adamanteus
C. gasperettii
PLA2-gC
PLA2-gA
PLA2-gB
PLA2-gD
non-toxic PLA2
Flanking genes
D
PLA2 Phylogeny
PLA2-2F
PLA2-2E
PLA2-gC
PLA2-gB
PLA2-gK
PLA2-gD
PLA2-gA
E
SVSPs
C. adamanteus
C. gasperettii
Group 1
Group 2
Group 3
Flanking genes
F
SVSPs Phylogeny
Outgroup
Group 1
Group 2
Group 3

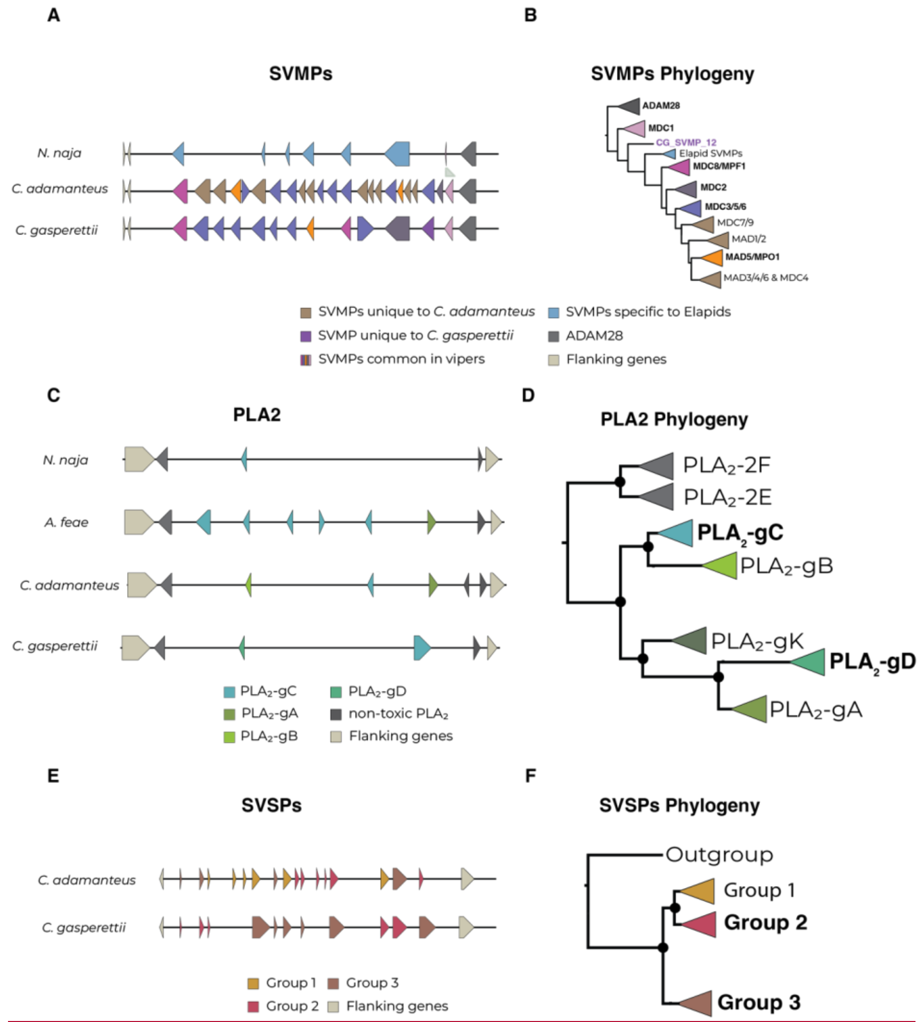

Fig. 4: A) ~~Microsynteny~~ Local synteny analyses for the SVMP toxin family in *Naja naja*, *Crotalus adamanteus* and *Cerastes gasperettii*. Different colors indicate orthologous genes unique to *C. gasperettii*, crotalids, true vipers or elapids. ADAM28 (right) as well as flanking genes (left) are also indicated. B) Phylogeny of SVMPs, in bold, groups that contained SVMPs from *Cerastes gasperettii*. In purple is indicated the gene exclusively found in *Cerastes gasperettii*. C) Local synteny analyses for PLA_2 in *Naja naja*, *Azemiops feae*, *Crotalus adamanteus* and *Cerastes gasperettii*. Non-toxic PLA_2 and flanking genes are also shown. D) Phylogeny of the PLA_2 gene family, with two non-toxic PLA_2 as outgroups. Some samples that did not fit in any category have been removed. For a complete phylogeny see Fig. S9. Note that PLA_2-gK is present in the phylogeny but not in the local synteny analyses, as any of the studied species contains it. E) Local synteny analyses for SVSPs for *Crotalus adamanteus* and *Cerastes gasperettii*. Flanking genes are also shown. F) Phylogeny for SVSPs with a non-toxic outgroup. For the three different phylogenies the groups that contained toxins from *Cerastes gasperettii* are highlighted in bold. ~~In bold, groups that contained SVMPs from *Cerastes gasperettii*. In purple is indicated the gene exclusively found in *Cerastes gasperettii*. C) Microsynteny for PLA_2 in *Naja naja*, *Azemiops feae*,~~

Crotalus adamanteus and *Cerastes gasperettii*. Non-toxic PLA_2 and flanking genes are also shown. D) Phylogeny of the PLA_2 gene family, with two non-toxic PLA_2 as outgroups. Some samples that did not fit in any category have been removed. For a complete phylogeny see Fig. S7. Note that PLA_2-gK is present in the phylogeny but not in the microsynteny, as any of the studied species contains it. E) Microsynteny for SVSPs for *Crotalus adamanteus* and *Cerastes gasperettii*. Flanking genes are also shown. F) Phylogeny for SVSPs with a non-toxic outgroup. For the three different phylogenies the groups that contained toxins from *Cerastes gasperettii* are highlighted in bold.

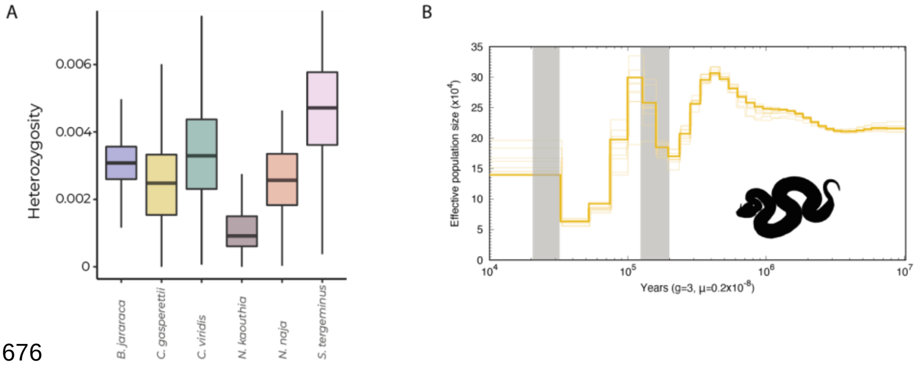

Fig. 5: A) Genome-wide diversity for a total of six different venomous snakes: *Bothrops jararaca*, *Cerastes gasperettii*, *Crotalus viridis*, *Naja kaouthia*, *Naja naja* and *Sistrurus tergeminus*. B) PSMC analysis recovering the ancient demographic history of *Cerastes gasperettii*. Generation time was set to 3 years and the substitution rate to $2.4x10^{-9}$ per site per year. Shaded lines represent 10 bootstrap estimates. Two last glacial periods are shown with grey lines.

Table 1: Comparison of our new reference genome for *Cerastes gasperettii* with other high-quality squamate genomes. Best value per category is shown in bold.

	Cerastes gasperettii	*Crotalus adamanteus*	*Naja naja*	*Anolis sagrei*
Genome size	1.63 Gbp	1.69 Gbp	1.79 Gbp	1.92 Gbp
Number of scaffolds	221	**27**	1,897	3,738

Scaffold N50	214.14 Mbp	208.9 Mbp	223.35 Mbp	**253.58** Mbp
Scaffold L50	**3**	**3**	**3**	4
Contig N50	45.6 Mbp	**67.5** Mbp	13.06 Mbp	0.2 Mbp

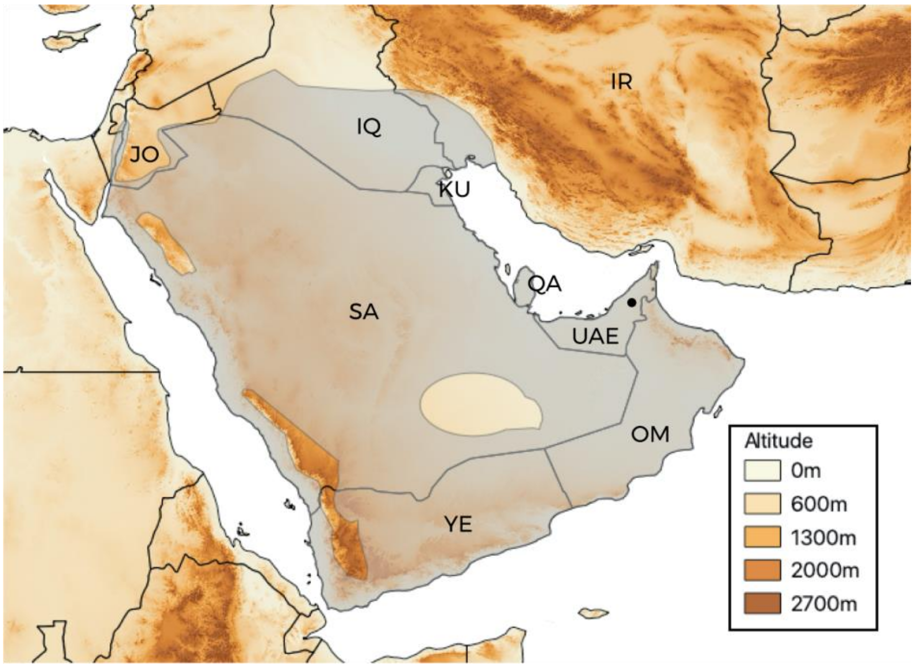

Fig. S1: Distribution map for the studied species *Cerastes gasperettii* with the location of our samples. Countries where the species is present are indicated. Abbreviations are as follows: JO, Jordania; SA, Saudi Arabia; YE, Yemen; OM, Oman; UAE, United Arab Emirates; IQ, Iraq; IR, Iran; KU, Kuwait, QA, Qatar.

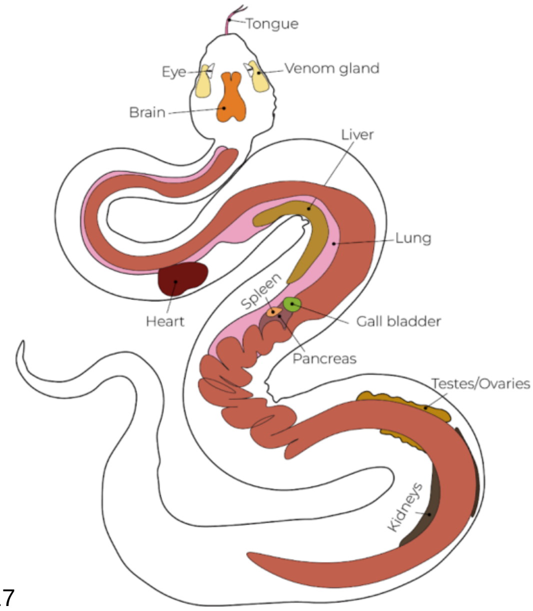

Fig. S2: Drawing of an Arabian horned viper depicting all the tissues sampled for RNA-seq analyses.

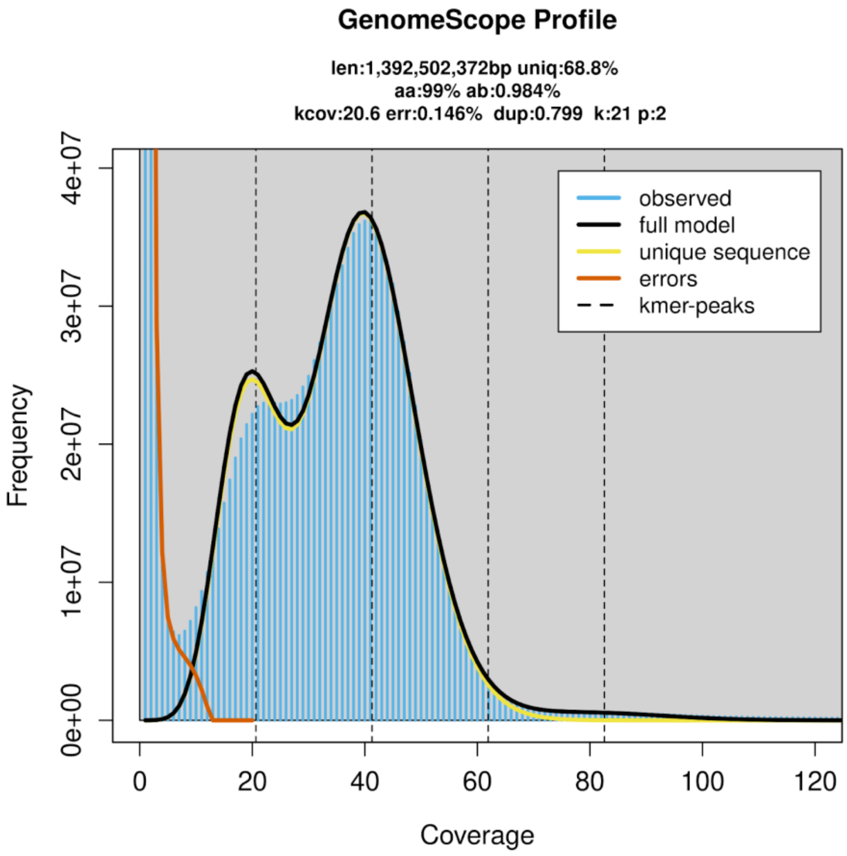

Fig. S3: Histogram from GenomeScope showing the frequency of reads in relation with their coverage.

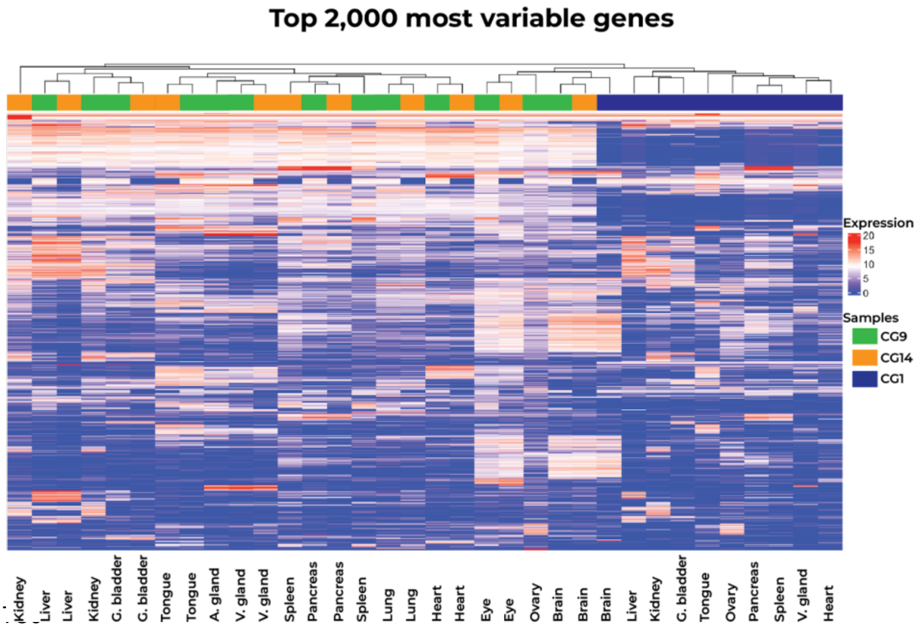

Fig. S4: Heatmap for the 2,000 most variable genes within our three samples, showing a clear batch effect of sample CG1 (possibly due to differences in sequencing time) as well as a high similarity between the putative accessory gland and the venom gland. Each column represents a different sampled tissue. The three different samples are depicted with different colors at the top of the heatmap. Abbreviations are as follows: G. bladder, gallbladder and V. gland, venom gland.

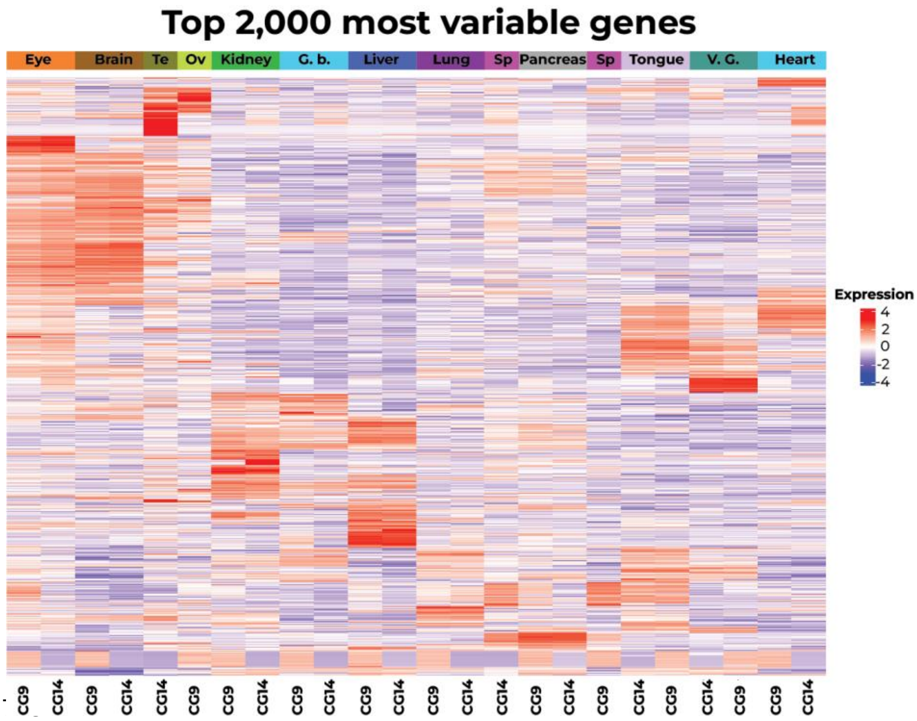

Fig. S5: Heatmap for the 2,000 most variable genes for both samples, reporting highly expressed genes unique for each tissue type. Each column represents one tissue sampled per individual. Expression levels were normalized. Abbreviations are as follows: Te, Testis; Ov, Ovary; G.b., gallbladder; Sp, Spleen and V.G., Venom gland.

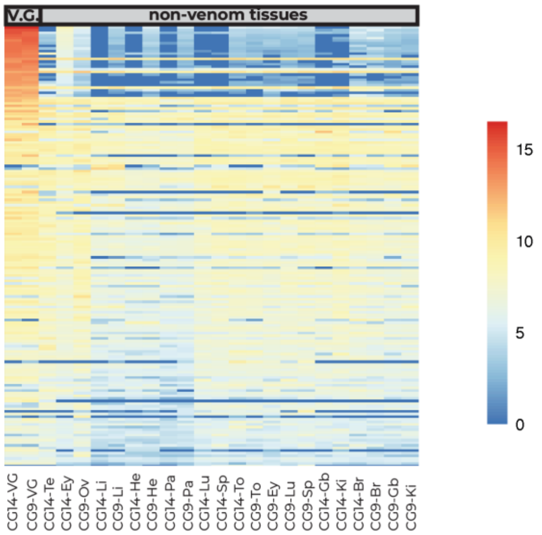

Fig. S6: Heatmap for the 161 upregulated genes found in the venom gland of *C. gasperettii* transcriptome including the 65 putative expressed toxins for both venom gland samples. Each column represents one tissue sampled per individual. Abbreviations are as follows: VG, Venom Gland; Ki, Kidney; GB, Gall Bladder; Lu; Lung; Sp, Spleen; He, Heart; Li, Liver; Pa, Pancreas; To, Tongue; Te, Testis; Ov, Ovary.

Formatted: Centered

Formatted: Spanish (Spain)

Formatted: English (United States)

Formatted: English (United States)

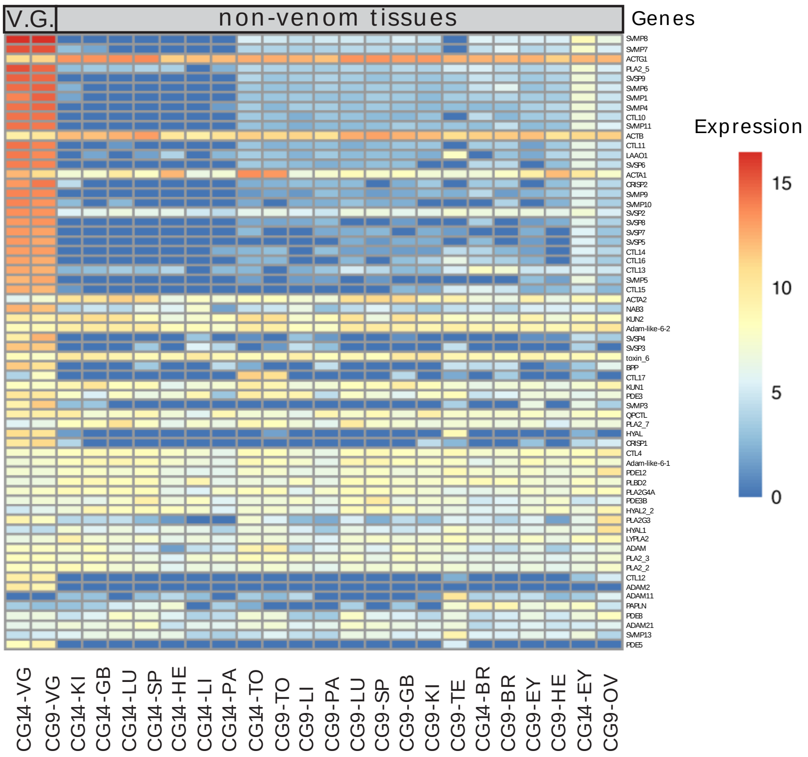

Fig. S7: Heatmap for the venom gland transcriptome for the 65 putative expressed toxins for both venom gland samples. Each column represents one tissue sampled per individual. Abbreviations are as follows: VG, Venom Gland; Ki, Kidney; GB, Gall Bladder; Lu, Lung; Sp, Spleen; He, Heart; Li, Liver; Pa, Pancreas; To, Tongue; Te, Testis; Ov, Ovary.

Formatted: English (United States)

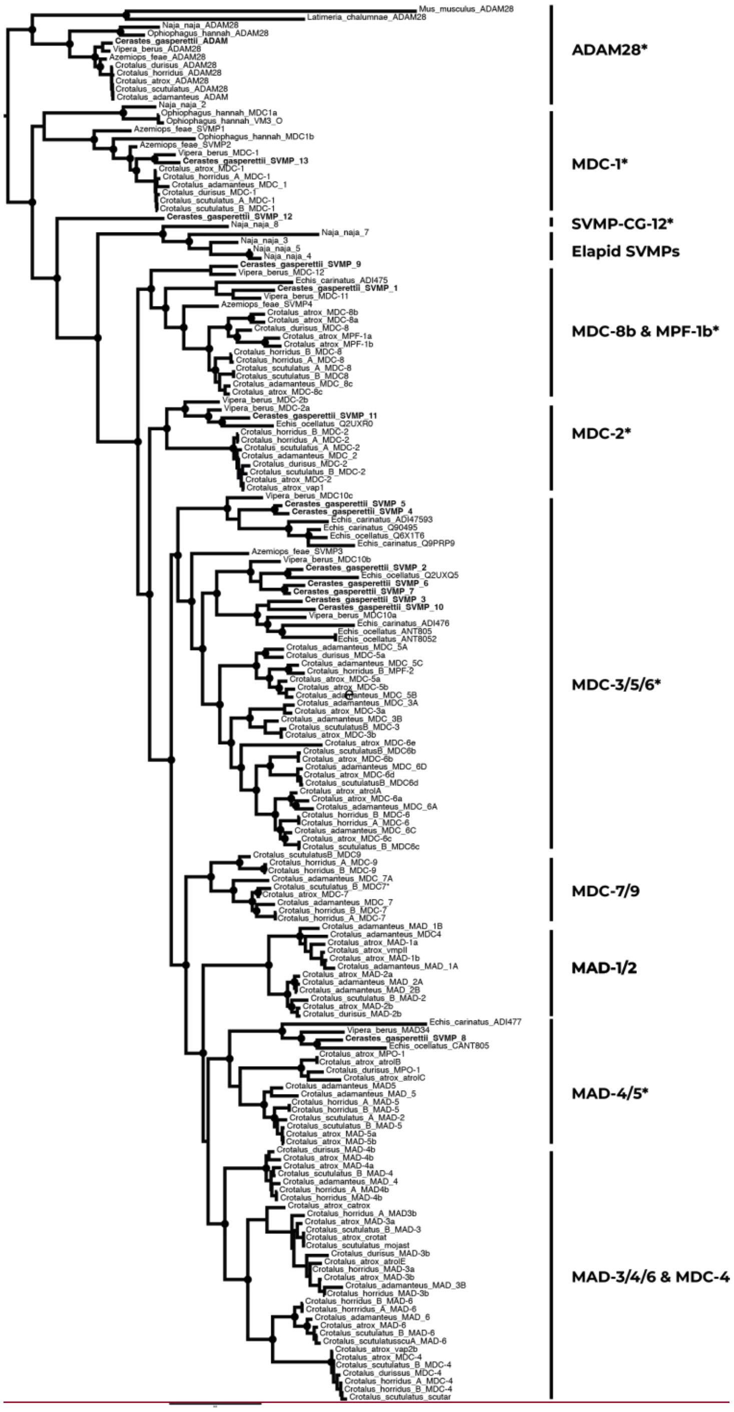
ADAM28*
MDC-1*
SVMP-CG-12*
Elapid SVMPs
MDC-8b & MPF-1b*
MDC-2*
MDC-3/5/6*
MDC-7/9
MAD-1/2
MAD-4/5*
MAD-3/4/6 & MDC-4

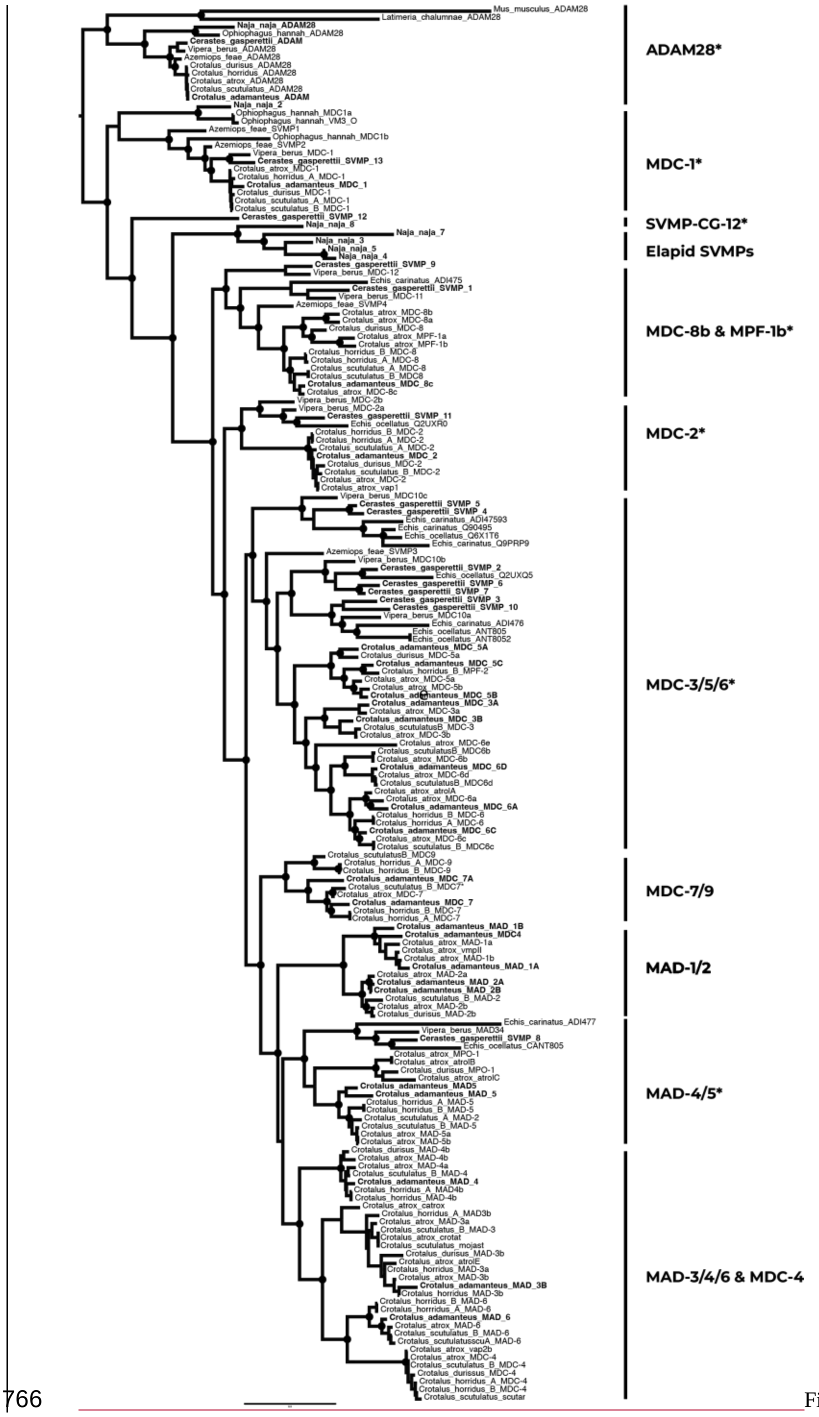

Fig. S86: Maximum

likelihood phylogeny for SVMP genes and its non-toxic paralog (ADAM28). Genes for *Cerastes gasperettii* are highlighted in bold. Toxin groups are identified following previous categorizations. Asterisks indicate if *Cerastes gasperettii* genes are present in that specific group. Branch support with aBayes values higher than 90 are depicted as circles.

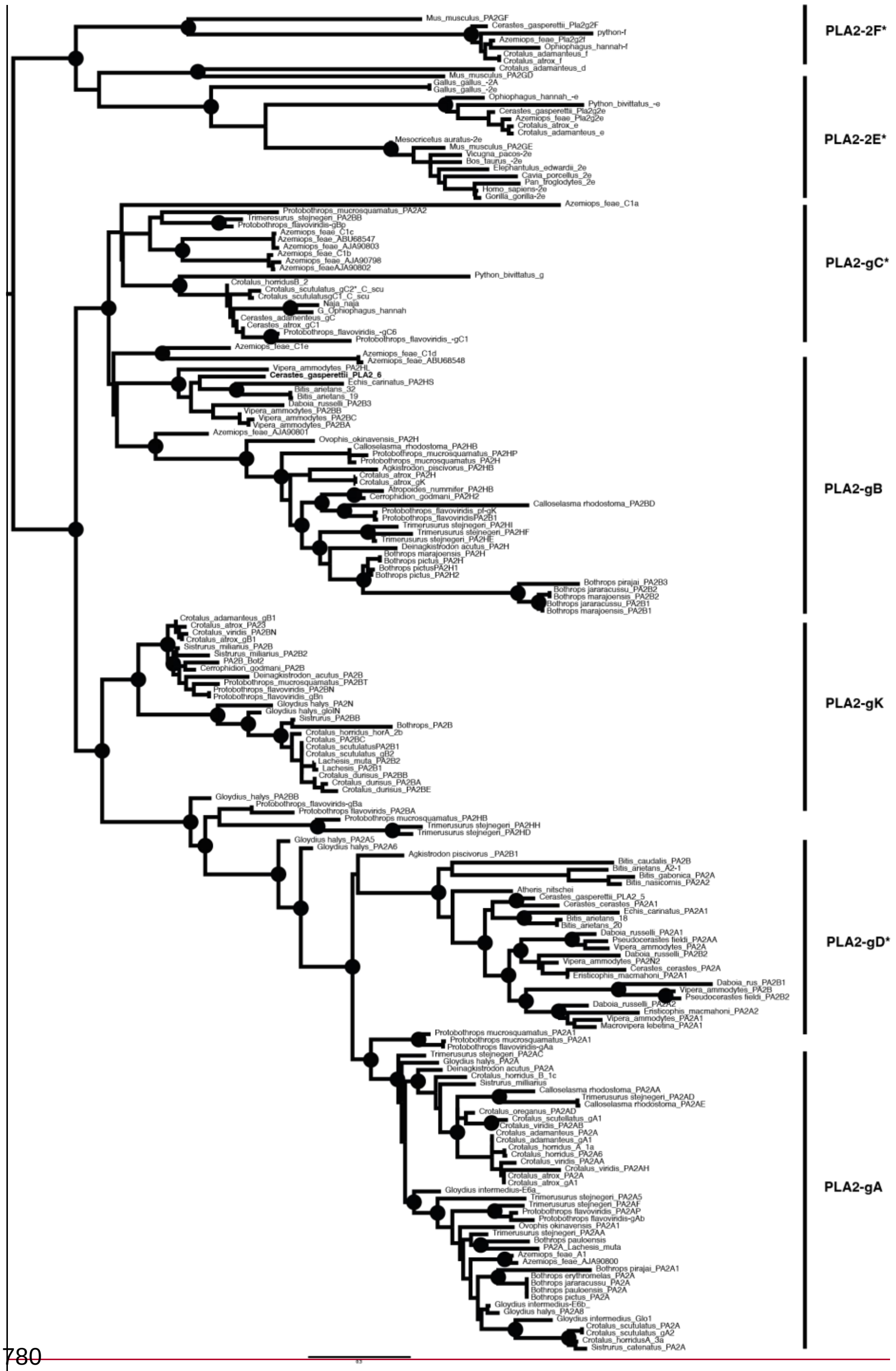
PLA2-2F*
PLA2-2E*
PLA2-gC*
PLA2-gB
PLA2-gK
PLA2-gD*
PLA2-gA

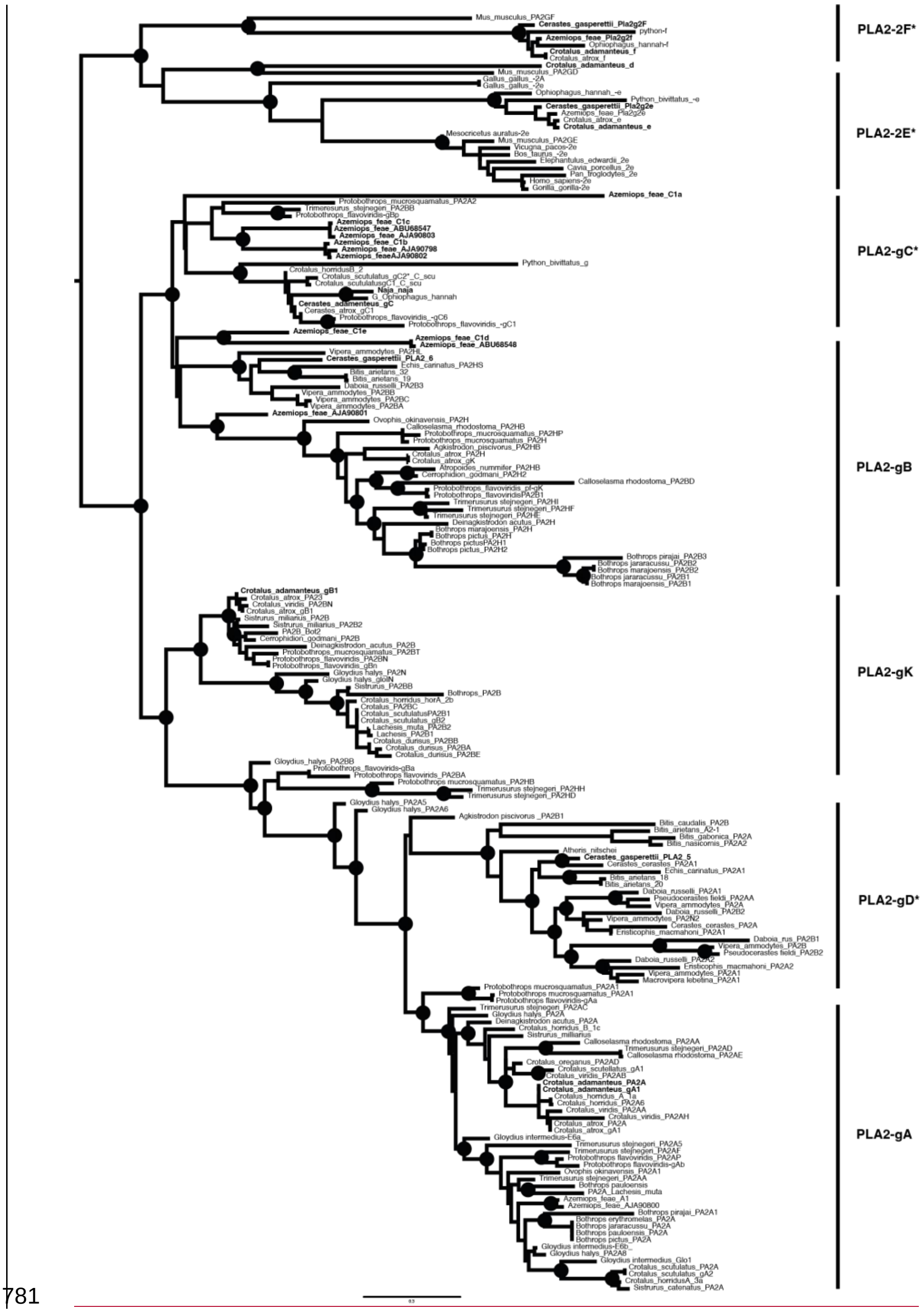
PLA2-2F*
PLA2-2E*
PLA2-gC*
PLA2-gB
PLA2-gK
PLA2-gD*
PLA2-gA

Fig. S97: Maximum likelihood phylogeny for PLA₂, with the two non-toxic genes as outgroups (PLA₂-2F and PLA₂-2E). Asterisks in group labels indicate if *Cerastes gasperettii* genes are present in that specific group. Branch support with aBayes values higher than 90 are depicted as circles.

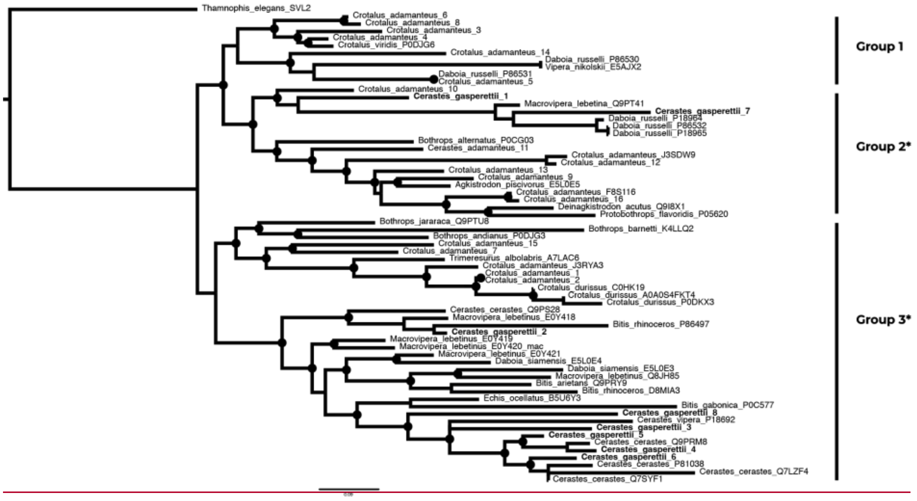

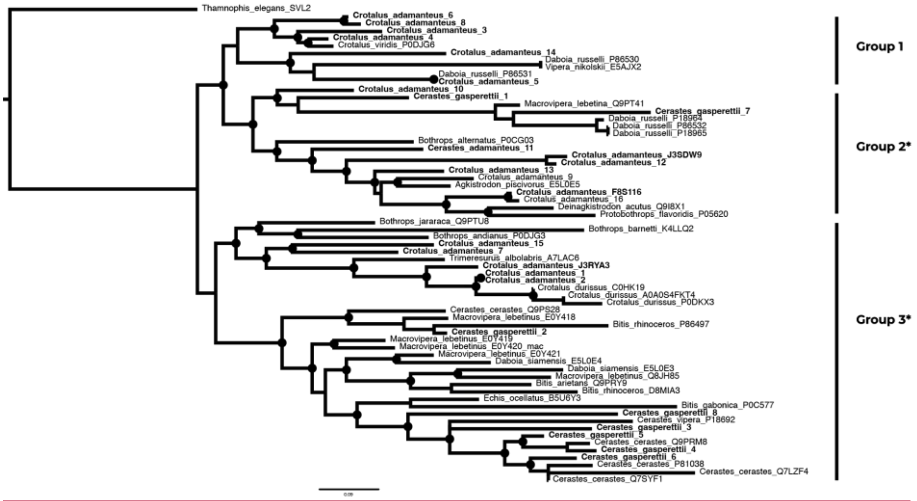

Fig. S10~~8~~: Maximum likelihood phylogeny for SVSPs, with one sample from *Thamnophis elegans* as outgroup. Asterisks in group labels indicate if *Cerastes gasperettii* genes are present in that specific group. Branch support with aBayes values higher than 90 are depicted as circles.

Table S1: Individuals sampled in this study with their sex, sampling coordinates and data sequenced.

**ID**	**Sex**	**Latitude**	**Longitude**	**Data sequenced**
CG1	Female	25.284690	55.687860	HiFi, Omni-C, Illumina, RNA-seq, Iso-seq, Proteome
CG9	Female	25.284690	55.687860	RNA-seq
CG14	Male	25.284690	55.687860	RNA-seq
CN6134	-	~~-~~UAE	~~-~~UAE	Proteome
CN6135	-	~~-~~UAE	UAE~~-~~	Proteome

Table S2: Id, tissue type and number of reads sequenced per sample.

**ID**	**Tissue**	**Reads**
CG9	Tongue	44,672,733
CG9	Venom gland	41,124,132
CG9	Eye	41,951,109
CG9	Brain	42,800,966
CG9	Heart	40,715,947
CG9	Lung	42,518,938
CG9	Liver	42,251,137
CG9	Gallbladder	42,738,665
CG9	Spleen	40,909,550
CG9	Pancreas	40,527,010
CG9	Ovary	41,118,336

CG9	Kidney	40,620,023
CG9	Accessory gland	44,114,293
CG14	Tongue	41,455,346
CG14	Venom gland	41,035,764
CG14	Eye	40,753,220
CG14	Brain	43,413,973
CG14	Heart	42,338,980
CG14	Lung	42,068,410
CG14	Liver	21,549,210
CG14	Gallglabbder	50,571,941
CG14	Spleen	45,447,235
CG14	Pancreas	50,223,941
CG14	Testis	47,495,900
CG14	Kidney	45,945,776
CG1	Heart	47,362,067
CG1	Brain	45,740,571
CG1	Kidney	50,758,869
CG1	Gallbladder	40,546,711
CG1	Liver	48,058,958
CG1	Spleen	44,752,981
CG1	Tongue	46,837,490
CG1	Pancreas	45,023,783
CG1	Venom gland	49,775,424
CG1	Ovary	48,703,420

Table S3: Different types of repetitive elements masked within the genome:

Element	Number of elements	Length (bp)	Percentage
Retroelements	1524124	493932584	30.25 %
SINEs:	339152	55265721	3.38
Penelope	124778	19471740	1.19
LINEs:	988815	347028895	21.25
CRE/SLACS	0	0	0.00%
L2/CR1/Rex	480371	137654000	8.43
R1/LOA/Jockey	579	99034	0.01
R2/R4/NeSL	41793	10873028	0.67
RTE/Bov-B	128092	79663597	4.88
L1/CIN4	207974	95913575	5.87
LTR elements:	196157	91637968	5.61
BEL/Pao	16545	5263265	0.32
Ty1/Copia	25582	15088781	0.92
Gypsy/DIRS1	102598	63604234	3.90
Retroviral	50617	7642063	0.47
DNA transposons	707499	111444059	6.83
hobo-Activator	265944	30679712	1.88
Tc1-IS630-Pogo	227637	58877559	3.61
En-Spm	0	0	0.00%
MULE-MuDR	44	3962	0.00%
PiggyBac	138	6619	0.00%
Tourist/Harbinger	182161	18395721	1.13
Other	0	0	0.00%
Rolling-circles	2242	136656	0.01
Unclassified	205700	42385187	2.60
Total interspersed repeats	-	647761830	39.67

Small RNA	6134	652217	0.04
Satellites	35838	4217238	0.26
Simple repeats	765726	53044358	3.25
Low complexity	97863	6694649	0.41

Table S4: Abundances for the different toxin families identified in the proteome of *C. gasperettii*

Toxin family	Percentage
SVMPi	8.65%
DISI	12.74%
DC domain	0.26%
CRISP	4.34%
PLA2	5.47%
SVSP	37.38%
SVMP-III	22.19%
PDE	0.02%
LAAO	1.71%
CTL	7.25%

References

Alföldi, J., Di Palma, F., Grabherr, M., Williams, C., Kong, L., Mauceli, E., Russell, P., Lowe, C. B., Glor, R. E., Jaffe, J. D., Ray, D. A., Boissinot, S., Shedlock, A. M., Botka, C., Castoe, T. A., Colbourne, J. K., Fujita, M. K., Moreno, R. G., Ten Hallers, B. F., … Lindblad-Toh, K. (2011). The genome of the green anole lizard and a comparative analysis with birds and mammals. *Nature*, *477*(7366), 587–591. https://doi.org/10.1038/nature10390

Ali, S. A., Jackson, T. N. W., Casewell, N. R., Low, D. H. W., Rossi, S., Baumann, K., Fathinia, B., Visser, J., Nouwens, A., Hendrikx, I., Jones, A., Undheim, E. A., & Fry, B. G. (2015). Extreme venom variation in Middle Eastern vipers: A proteomics comparison of *Eristicophis macmahonii*, *Pseudocerastes fieldi* and *Pseudocerastes persicus*. *Journal of Proteomics*, *116*, 106–113. https://doi.org/10.1016/j.jprot.2014.09.003

Allio, R., Schomaker-Bastos, A., Romiguier, J., Prosdocimi, F., Nabholz, B., & Delsuc, F. (2020). MitoFinder: Efficient automated large-scale extraction of mitogenomic data in target enrichment phylogenomics. *Molecular Ecology Resources*, *20*(4), 892–905. https://doi.org/10.1111/1755-0998.13160

Almeida, D. D., Viala, V. L., Nachtigall, P. G., Broe, M., Gibbs, H. L., Serrano, S. M. D. T., Moura-da-Silva, A. M., Ho, P. L., Nishiyama-Jr, M. Y., & Junqueira-de-Azevedo, I. L. M. (2021). Tracking the recruitment and evolution of snake toxins using the evolutionary context provided by the *Bothrops jararaca* genome. *Proceedings of the National Academy of Sciences*, *118*(20), e2015159118. https://doi.org/10.1073/pnas.2015159118

Al-Sadoon, M. K., & Paray, B. A. (2016). Ecological aspects of the horned viper, *Cerastes cerastes gasperettii* in the central region of Saudi Arabia. *Saudi Journal of Biological Sciences*, *23*(1), 135–138. https://doi.org/10.1016/j.sjbs.2015.10.010

Amr, Z. S., Abu Baker, M. A., & Warrell, D. A. (2020). Terrestrial venomous snakes and snakebites in the Arab countries of the Middle East. *Toxicon*, *177*, 1–15. https://doi.org/10.1016/j.toxicon.2020.01.012

Andrews, S. (2010). *FastQC: a quality control tool for high throughput sequence data.*

Arnold, N. E., Robinson, M. D., & Carranza, S. (2009). A preliminary analysis of phylogenetic relationships and biogeography of the dangerously venomous Carpet Vipers, *Echis* (Squamata, Serpentes, Viperidae) based on mitochondrial DNA sequences. *Amphibia Reptilia*, *30*(2), 273–282. https://doi.org/10.1163/156853809788201090

Avella, I., Calvete, J. J., Sanz, L., Wüster, W., Licata, F., Quesada-Bernat, S., Rodríguez, Y., & Martínez-Freiría, F. (2022). Interpopulational variation and ontogenetic shift in the venom composition of Lataste's viper (*Vipera latastei*, Boscá 1878) from northern Portugal. *Journal of Proteomics*, *263*, 104613. https://doi.org/10.1016/j.jprot.2022.104613

Bao, W., Kojima, K. K., & Kohany, O. (2015). Repbase Update, a database of repetitive elements in eukaryotic genomes. *Mobile DNA*, *6*(1), 11. https://doi.org/10.1186/s13100-015-0041-9

Broad Institute. (2021). *Picard Tools*. Broad Institute, GitHub Repository.

Burriel-Carranza, B., Tejero-Cicuéndez, H., Carné, A., Riaño, G., Talavera, A., Saadi, S. A., Els, J., Šmíd, J., Tamar, K., Tarroso, P., & Carranza, S. (2023). The origin of a mountain biota: Hyper-aridity shaped reptile diversity in an Arabian biodiversity hotspot. https://doi.org/10.1101/2023.04.07.536010

Bylsma, R., Walkup, D. K., Hibbitts, T. J., Ryberg, W. A., Black, A. N., & DeWoody, J. A. (2022). Population genetic and genomic analyses of Western Massasauga (*Sistrurus tergeminus* ssp.): Implications for subspecies delimitation and conservation. *Conservation Genetics*, *23*(2), 271–283. https://doi.org/10.1007/s10592-021-01420-8

Calvete, J. J., Pla, D., Els, J., Carranza, S., Damm, M., Hempel, B.-F., John, E. B. O., Petras, D., Heiss, P., Nalbantsoy, A., Göçmen, B., Süssmuth, R. D., Calderón-Celis, F., Nosti, A. J., & Encinar, J. R. (2021). Combined Molecular and Elemental Mass Spectrometry Approaches for Absolute Quantification of Proteomes: Application to the Venomics Characterization of the Two Species of Desert Black Cobras, *Walterinnesia aegyptia* and *Walterinnesia morgani*. *Journal of Proteome Research*, *20*(11), 5064–5078. https://doi.org/10.1021/acs.jproteome.1c00608

Carranza, S., Els, J., & Burriel-Carranza, B. (2021). *A field guide to the reptiles of Oman*.

Casewell, N. R., Harrison, R. A., Wüster, W., & Wagstaff, S. C. (2009). Comparative venom gland transcriptome surveys of the saw-scaled vipers (Viperidae: *Echis*) reveal substantial intra-family gene diversity and novel venom transcripts. *BMC Genomics*, *10*(1), 564. https://doi.org/10.1186/1471-2164-10-564

Casewell, N. R., Wagstaff, S. C., Wüster, W., Cook, D. A. N., Bolton, F. M. S., King, S. I., Pla, D., Sanz, L., Calvete, J. J., & Harrison, R. A. (2014). Medically important differences in snake venom composition are dictated by distinct postgenomic mechanisms. *Proceedings of the*

Formatted: Font: Italic

Formatted: Font: Italic

Formatted: Font: Italic

Formatted: Font: Italic

Formatted: Font: Italic

Formatted: Font: Italic

Formatted: Font: Not Italic

Formatted: Font: Italic

Formatted: Font: Italic

National Academy of Sciences, *111*(25), 9205–9210. https://doi.org/10.1073/pnas.1405484111

Casewell, N. R., Wüster, W., Vonk, F. J., Harrison, R. A., & Fry, B. G. (2013). Complex cocktails: The evolutionary novelty of venoms. *Trends in Ecology & Evolution*, *28*(4), 219–229. https://doi.org/10.1016/j.tree.2012.10.020

Challis, R., Richards, E., Rajan, J., Cochrane, G., & Blaxter, M. (2020). BlobToolKit – Interactive Quality Assessment of Genome Assemblies. *G3 Genes|Genomes|Genetics*, *10*(4), 1361–1374. https://doi.org/10.1534/g3.119.400908

Chen, S., Zhou, Y., Chen, Y., & Gu, J. (2018). Fastp: An ultra-fast all-in-one FASTQ preprocessor. *Bioinformatics*, *34*(17), i884–i890. https://doi.org/10.1093/bioinformatics/bty560

Cheng, H., Concepcion, G. T., Feng, X., Zhang, H., & Li, H. (2021). Haplotype-resolved de novo assembly using phased assembly graphs with hifiasm. *Nature Methods*, *18*(2), Article 2. https://doi.org/10.1038/s41592-020-01056-5

Dainat, J., Hereñú, D., Dr. K. D. Murray, Davis, E., Crouch, K., LucileSol, Agostinho, N., Pascal-Git, Zollman, Z., & Tayyrov. (2023). *NBISweden/AGAT: AGAT-v1.2.0* (v1.2.0) [Computer software]. Zenodo. https://doi.org/10.5281/ZENODO.3552717

Danecek, P., Bonfield, J. K., Liddle, J., Marshall, J., Ohan, V., Pollard, M. O., Whitwham, A., Keane, T., McCarthy, S. A., Davies, R. M., & Li, H. (2021). Twelve years of SAMtools and BCFtools. *GigaScience*, *10*(2). https://doi.org/10.1093/gigascience/giab008

Dowell, N. L., Giorgianni, M. W., Kassner, V. A., Selegue, J. E., Sanchez, E. E., & Carroll, S. B. (2016). The Deep Origin and Recent Loss of Venom Toxin Genes in Rattlesnakes. *Current Biology*, *26*(18), 2434–2445. https://doi.org/10.1016/j.cub.2016.07.038

Drukewitz, S. H., & Von Reumont, B. M. (2019). The Significance of Comparative Genomics in Modern Evolutionary Venomics. *Frontiers in Ecology and Evolution*, *7*, 163. https://doi.org/10.3389/fevo.2019.00163

Dussex, N., van der Valk, T., Morales, H. E., Wheat, C. W., Díez-del-Molino, D., von Seth, J., Foster, Y., Kutschera, V. E., Guschanski, K., Rhie, A., Phillippy, A. M., Korlach, J., Howe, K., Chow, W., Pelan, S., Mendes Damas, J. D., Lewin, H. A., Hastie, A. R., Formenti, G., … Dalén, L. (2021). Population genomics of the critically endangered kākāpō. *Cell Genomics*, *1*(1), 100002. https://doi.org/10.1016/j.xgen.2021.100002

Egan, D., Amr, Z., Al Johany, A., Els, J., Papenfuss, T., Nilson, Sadek, R., Disi, A., Hraoui-Bloquet, S., Werner, Y., & Anderson, S. (2012). *The IUCN Red List of Threatened Species: Cerastes gasperettii* [dataset]. https://doi.org/10.2305/IUCN.UK.2012.RLTS.T164599A1060588.en

Fahmi, L., Makran, B., Pla, D., Sanz, L., Oukkache, N., Lkhider, M., Harrison, R. A., Ghalim, N., & Calvete, J. J. (2012). Venomics and antivenomics profiles of North African *Cerastes cerastes* and *C. vipera* populations reveals a potentially important therapeutic weakness. *Journal of Proteomics*, *75*(8), 2442–2453. https://doi.org/10.1016/j.jprot.2012.02.021

Ferraz, C. R., Arrahman, A., Xie, C., Casewell, N. R., Lewis, R. J., Kool, J., & Cardoso, F. C. (2019). Multifunctional Toxins in Snake Venoms and Therapeutic Implications: From Pain to Hemorrhage and Necrosis. *Frontiers in Ecology and Evolution*, *7*. https://www.frontiersin.org/articles/10.3389/fevo.2019.00218

Flynn, J. M., Hubley, R., Goubert, C., Rosen, J., Clark, A. G., Feschotte, C., & Smit, A. F. (2020). RepeatModeler2 for automated genomic discovery of transposable element families. *Proceedings of the National Academy of Sciences*, *117*(17), 9451–9457. https://doi.org/10.1073/pnas.1921046117

Formenti, G., Abueg, L., Brajuka, A., Brajuka, N., Gallardo-Alba, C., Giani, A., Fedrigo, O., & Jarvis, E. D. (2022). Gfastats: Conversion, evaluation and manipulation of genome sequences using assembly graphs. *Bioinformatics*, *38*(17), 4214–4216. https://doi.org/10.1093/bioinformatics/btac460

Frantz, L. A. F., Bradley, D. G., Larson, G., & Orlando, L. (2020). Animal domestication in the era of ancient genomics. *Nature Reviews Genetics*, *21*(8), Article 8. https://doi.org/10.1038/s41576-020-0225-0

Fry, B. (Ed.). (2015). *Venomous reptiles and their toxins: Evolution, pathophysiology, and biodiscovery*. Oxford University Press.

Formatted: Font: Italic

Formatted: Font: Italic

Fry, B. G., Roelants, K., Champagne, D. E., Scheib, H., Tyndall, J. D. A., King, G. F., Nevalainen, T. J., Norman, J. A., Lewis, R. J., Norton, R. S., Renjifo, C., & de la Vega, R. C. R. (2009). The toxicogenomic multiverse: Convergent recruitment of proteins into animal venoms. *Annual Review of Genomics and Human Genetics*, *10*, 483–511. https://doi.org/10.1146/annurev.genom.9.081307.164356

Fry, B. G., Scheib, H., van der Weerd, L., Young, B., McNaughtan, J., Ramjan, S. F. R., Vidal, N., Poelmann, R. E., & Norman, J. A. (2008). Evolution of an Arsenal: Structural and Functional Diversification of the Venom System in the Advanced Snakes (Caenophidia)*. *Molecular & Cellular Proteomics*, *7*(2), 215–246. https://doi.org/10.1074/mcp.M700094-MCP200

Fry, B. G., & Wüster, W. (2004). Assembling an Arsenal: Origin and Evolution of the Snake Venom Proteome Inferred from Phylogenetic Analysis of Toxin Sequences. *Molecular Biology and Evolution*, *21*(5), 870–883. https://doi.org/10.1093/molbev/msh091

Gabriel, L., Hoff, K. J., Brůna, T., Borodovsky, M., & Stanke, M. (2021). TSEBRA: Transcript selector for BRAKER. *BMC Bioinformatics*, *22*(1), 566. https://doi.org/10.1186/s12859-021-04482-0

Geneva, A. J., Park, S., Bock, D. G., De Mello, P. L. H., Sarigol, F., Tollis, M., Donihue, C. M., Reynolds, R. G., Feiner, N., Rasys, A. M., Lauderdale, J. D., Minchey, S. G., Alcala, A. J., Infante, C. R., Kolbe, J. J., Schluter, D., Menke, D. B., & Losos, J. B. (2022). Chromosome-scale genome assembly of the brown anole (*Anolis sagrei*), an emerging model species. *Communications Biology*, *5*(1), 1126. https://doi.org/10.1038/s42003-022-04074-5

Ghurye, J., Rhie, A., Walenz, B. P., Schmitt, A., Selvaraj, S., Pop, M., Phillippy, A. M., & Koren, S. (2019). Integrating Hi-C links with assembly graphs for chromosome-scale assembly. *PLoS Computational Biology*, *15*(8), e1007273. https://doi.org/10.1371/journal.pcbi.1007273

Gilbert, C., Meik, J. M., Dashevsky, D., Card, D. C., Castoe, T. A., & Schaack, S. (2014). Endogenous hepadnaviruses, bornaviruses and circoviruses in snakes. *Proceedings of the Royal Society B: Biological Sciences*, *281*(1791), 20141122. https://doi.org/10.1098/rspb.2014.1122

Giorgianni, M. W., Dowell, N. L., Griffin, S., Kassner, V. A., Selegue, J. E., & Carroll, S. B. (2020). The origin and diversification of a novel protein family in venomous snakes. *Proceedings of the National Academy of Sciences*, *117*(20), 10911–10920. https://doi.org/10.1073/pnas.1920011117

Glennie, K. W., & Singhvi, A. K. (2002). Event stratigraphy, paleoenvironment and chronology of SE Arabian deserts. *Quaternary Science Reviews*, *21*(7), 853–869. https://doi.org/10.1016/S0277-3791(01)00133-0

Green, R. E., Braun, E. L., Armstrong, J., Earl, D., Nguyen, N., Hickey, G., Vandewege, M. W., St. John, J. A., Capella-Gutiérrez, S., Castoe, T. A., Kern, C., Fujita, M. K., Opazo, J. C., Jurka, J., Kojima, K. K., Caballero, J., Hubley, R. M., Smit, A. F., Platt, R. N., … Ray, D. A. (2014). Three crocodilian genomes reveal ancestral patterns of evolution among archosaurs. *Science*, *346*(6215), 1254449. https://doi.org/10.1126/science.1254449

Guan, D., McCarthy, S. A., Wood, J., Howe, K., Wang, Y., & Durbin, R. (2020). Identifying and removing haplotypic duplication in primary genome assemblies. *Bioinformatics*, *36*(9), 2896–2898. https://doi.org/10.1093/bioinformatics/btaa025

Guindon, S., Dufayard, J.-F., Lefort, V., Anisimova, M., Hordijk, W., & Gascuel, O. (2010). New Algorithms and Methods to Estimate Maximum-Likelihood Phylogenies: Assessing the Performance of PhyML 3.0. *Systematic Biology*, *59*(3), 307–321. https://doi.org/10.1093/sysbio/syq010

Gurevich, A., Saveliev, V., Vyahhi, N., & Tesler, G. (2013). QUAST: Quality assessment tool for genome assemblies. *Bioinformatics (Oxford, England)*, *29*(8), 1072–1075. https://doi.org/10.1093/bioinformatics/btt086

Gutiérrez, J. M., Warrell, D. A., Williams, D. J., Jensen, S., Brown, N., Calvete, J. J., ... & Global Snakebite Initiative. (2013). The need for full integration of snakebite envenoming within a global strategy to combat the neglected tropical diseases: the way forward. *PLoS neglected tropical diseases*, *7*(6), e2162.

Formatted: Font: Italic

Formatted: Font: (Default) Times New Roman, 11 pt

Formatted: Indent: Left: 0", Line spacing: single

Formatted: Font: 11 pt

Gutiérrez, J. M., Calvete, J. J., Habib, A. G., Harrison, R. A., Williams, D. J., & Warrell, D. A. (2017). Snakebite envenoming. *Nature Reviews Disease Primers*, *3*(1), Article 1. https://doi.org/10.1038/nrdp.2017.63

Hirst, Samuel R., Rhett M. Rautsaw, Cameron M. VanHorn, Marc A. Beer, Preston J. McDonald, Ramsés Alejandro Rosales García, Bruno Rodriguez Lopez et al. "Where the "ruber" Meets the Road: Using the Genome of the Red Diamond Rattlesnake to Unravel the Evolutionary Processes Driving Venom Evolution." *Genome Biology and Evolution* 16, no. 9 (2024): https://doi.org/10.1093/gbe/evae198

Hogan, M. P., Holding, M. L., Nystrom, G. S., Colston, T. J., Bartlett, D. A., Mason, A. J., Ellsworth, S. A., Rautsaw, R. M., Lawrence, K. C., Strickland, J. L., He, B., Fraser, P., Margres, M. J., Gilbert, D. M., Gibbs, H. L., Parkinson, C. L., & Rokyta, D. R. (2024). The genetic regulatory architecture and epigenomic basis for age-related changes in rattlesnake venom. *Proceedings of the National Academy of Sciences*, *121*(16), e2313440121. https://doi.org/10.1073/pnas.2313440121

Hogan, M. P., Whittington, A. C., Broe, M. B., Ward, M. J., Gibbs, H. L., & Rokyta, D. R. (2021). The Chemosensory Repertoire of the Eastern Diamondback Rattlesnake (*Crotalus adamanteus*) Reveals Complementary Genetics of Olfactory and Vomeronasal-Type Receptors. *Journal of Molecular Evolution*, *89*(4), 313–328. https://doi.org/10.1007/s00239-021-10007-3

Jan, V., Maroun, R. C., Robbe-Vincent, A., De Haro, L., & Choumet, V. (2002). Toxicity evolution of *Vipera aspis aspis* venom: Identification and molecular modeling of a novel phospholipase A2 heterodimer neurotoxin11Nucleotide sequence data reported are available in the EMBL database under the accession numbers AJ459806 and AJ459807. *FEBS Letters*, *527*(1), 263–268. https://doi.org/10.1016/S0014-5793(02)03205-2

Jin, J.-J., Yu, W.-B., Yang, J.-B., Song, Y., dePamphilis, C. W., Yi, T.-S., & Li, D.-Z. (2020). GetOrganelle: A fast and versatile toolkit for accurate de novo assembly of organelle genomes. *Genome Biology*, *21*(1), 241. https://doi.org/10.1186/s13059-020-02154-5

Jones, P., Binns, D., Chang, H.-Y., Fraser, M., Li, W., McAnulla, C., McWilliam, H., Maslen, J., Mitchell, A., Nuka, G., Pesseat, S., Quinn, A. F., Sangrador-Vegas, A., Scheremetjew, M., Yong, S.-Y., Lopez, R., & Hunter, S. (2014). InterProScan 5: Genome-scale protein function classification. *Bioinformatics*, *30*(9), 1236–1240. https://doi.org/10.1093/bioinformatics/btu031

Kalita, B., Mackessy, S. P., & Mukherjee, A. K. (2018). Proteomic analysis reveals geographic variation in venom composition of Russell's Viper in the Indian subcontinent: Implications for clinical manifestations post-envenomation and antivenom treatment. *Expert Review of Proteomics*, *15*(10), 837–849. https://doi.org/10.1080/14789450.2018.1528150

Katoh, K., & Standley, D. M. (2013). MAFFT Multiple Sequence Alignment Software Version 7: Improvements in Performance and Usability. *Molecular Biology and Evolution*, *30*(4), 772–780. https://doi.org/10.1093/molbev/mst010

Keilwagen, J., Hartung, F., & Grau, J. (2019). GeMoMa: Homology-Based Gene Prediction Utilizing Intron Position Conservation and RNA-seq Data. In M. Kollmar (Ed.), *Gene Prediction* (Vol. 1962, pp. 161–177). Springer New York. https://doi.org/10.1007/978-1-4939-9173-0_9

Kiełbasa, S. M., Wan, R., Sato, K., Horton, P., & Frith, M. C. (2011). Adaptive seeds tame genomic sequence comparison. *Genome Research*, *21*(3), 487–493. https://doi.org/10.1101/gr.113985.110

Kim, D., Paggi, J. M., Park, C., Bennett, C., & Salzberg, S. L. (2019). Graph-based genome alignment and genotyping with HISAT2 and HISAT-genotype. *Nature Biotechnology*, *37*(8), 907–915. https://doi.org/10.1038/s41587-019-0201-4

King, G. F. (2011). Venoms as a platform for human drugs: Translating toxins into therapeutics. *Expert Opinion on Biological Therapy*, *11*(11), 1469–1484. https://doi.org/10.1517/14712598.2011.621940

Li, D., Luo, R., Liu, C.-M., Leung, C.-M., Ting, H.-F., Sadakane, K., Yamashita, H., & Lam, T.-W. (2016). MEGAHIT v1.0: A fast and scalable metagenome assembler driven by advanced methodologies and community practices. *Methods*, *102*, 3–11. https://doi.org/10.1016/j.ymeth.2016.02.020

Formatted: Font: 10 pt

Formatted: Font: (Default) Times New Roman, Spanish (Spain)

Formatted: Indent: Left: 0", Line spacing: single

Formatted: Font: (Default) Times New Roman

Formatted: Font: (Default) Times New Roman, 10 pt

Formatted: Font: 10 pt

Formatted: Font: Italic

Formatted: Font: Italic

Li, H. (2013). *Aligning sequence reads, clone sequences and assembly contigs with BWA-MEM*. http://arxiv.org/abs/1303.3997

Li, H., & Durbin, R. (2011). Inference of human population history from individual whole-genome sequences. *Nature 2011 475:7357*, *475*(7357), 493–496. https://doi.org/10.1038/nature10231

Li, H., Handsaker, B., Wysoker, A., Fennell, T., Ruan, J., Homer, N., Marth, G., Abecasis, G., & Durbin, R. (2009). The Sequence Alignment/Map format and SAMtools. *Bioinformatics*, *25*(16), 2078–2079. https://doi.org/10.1093/bioinformatics/btp352

Li, L., Huang, J., & Lin, Y. (2018). Snake Venoms in Cancer Therapy: Past, Present and Future. *Toxins*, *10*(9), 346. https://doi.org/10.3390/toxins10090346

Love, M. I., Huber, W., & Anders, S. (2014). Moderated estimation of fold change and dispersion for RNA-seq data with DESeq2. *Genome Biology*, *15*(12), 550. https://doi.org/10.1186/s13059-014-0550-8

Mackessy, S. P. (2010). Evolutionary trends in venom composition in the Western Rattlesnakes (*Crotalus viridis* sensu lato): Toxicity vs. tenderizers. *Toxicon*, *55*(8), 1463–1474. https://doi.org/10.1016/j.toxicon.2010.02.028

Margres, M. J., McGivern, J. J., Wray, K. P., Seavy, M., Calvin, K., & Rokyta, D. R. (2014). Linking the transcriptome and proteome to characterize the venom of the eastern diamondback rattlesnake (*Crotalus adamanteus*). *Journal of Proteomics*, *96*, 145–158. https://doi.org/10.1016/j.jprot.2013.11.001

Margres, M. J., Rautsaw, R. M., Strickland, J. L., Mason, A. J., Schramer, T. D., Hofmann, E. P., Stiers, E., Ellsworth, S. A., Nystrom, G. S., Hogan, M. P., Bartlett, D. A., Colston, T. J., Gilbert, D. M., Rokyta, D. R., & Parkinson, C. L. (2021). The Tiger Rattlesnake genome reveals a complex genotype underlying a simple venom phenotype. *Proceedings of the National Academy of Sciences*, *118*(4), e2014634118. https://doi.org/10.1073/pnas.2014634118

Margres, M. J., Wray, K. P., Sanader, D., McDonald, P. J., Trumbull, L. M., Patton, A. H., & Rokyta, D. R. (2021). Varying Intensities of Introgression Obscure Incipient Venom-Associated Speciation in the Timber Rattlesnake (*Crotalus horridus*). *Toxins*, *13*(11), Article 11. https://doi.org/10.3390/toxins13110782

Martin, M. (2011). Cutadapt removes adapter sequences from high-throughput sequencing reads. *EMBnet.Journal*, *17*(1), 10. https://doi.org/10.14806/ej.17.1.200

McKenna, A., Hanna, M., Banks, E., Sivachenko, A., Cibulskis, K., Kernytsky, A., Garimella, K., Altshuler, D., Gabriel, S., Daly, M., & DePristo, M. A. (2010). The genome analysis toolkit: A MapReduce framework for analyzing next-generation DNA sequencing data. *Genome Research*, *20*(9), 1297–1303. https://doi.org/10.1101/gr.107524.110

Mochales-Riaño, G., Burriel-Carranza, B., Barros, M. I., Velo-Antón, G., Talavera, A., Spilani, L., Tejero-Cicuéndez, H., Crochet, P.-A., Piris, A., García-Cardenete, L., Busais, S., Els, J., Shobrak, M., Brito, J. C., Šmíd, J., Carranza, S., & Martínez-Freiría, F. (2024). Hidden in the sand: Phylogenomics unravel an unexpected evolutionary history for the desert-adapted vipers of the genus *Cerastes*. *Molecular Phylogenetics and Evolution*, *191*, 107979. https://doi.org/10.1016/j.ympev.2023.107979

Myers, E. A., Strickland, J. L., Rautsaw, R. M., Mason, A. J., Schramer, T. D., Nystrom, G. S., Hogan, M. P., Yooseph, S., Rokyta, D. R., & Parkinson, C. L. (2022). De Novo Genome Assembly Highlights the Role of Lineage-Specific Gene Duplications in the Evolution of Venom in Fea's Viper (*Azemiops feae*). *Genome Biology and Evolution*, *14*(7), evac082. https://doi.org/10.1093/gbe/evac082

Orteu, A., & Jiggins, C. D. (2020). The genomics of coloration provides insights into adaptive evolution. *Nature Reviews Genetics*, *21*(8), Article 8. https://doi.org/10.1038/s41576-020-0234-z

Osipov, A., & Utkin, Y. (2023). What Are the Neurotoxins in Hemotoxic Snake Venoms? *International Journal of Molecular Sciences*, *24*(3), Article 3. https://doi.org/10.3390/ijms24032919

Pardos-Blas, J. R., Irisarri, I., Abalde, S., Afonso, C. M. L., Tenorio, M. J., & Zardoya, R. (2021). The genome of the venomous snail *Lautoconus ventricosus* sheds light on the origin of conotoxin diversity. *GigaScience*, *10*(5), giab037. https://doi.org/10.1093/gigascience/giab037

Formatted: Font: Italic

Formatted: Font: Italic

Formatted: Font: Italic

Formatted: Font: Italic

Pertea, M., Pertea, G. M., Antonescu, C. M., Chang, T.-C., Mendell, J. T., & Salzberg, S. L. (2015). StringTie enables improved reconstruction of a transcriptome from RNA-seq reads. *Nature Biotechnology*, *33*(3), 290–295. https://doi.org/10.1038/nbt.3122

Pook, C. E., Joger, U., Stümpel, N., & Wüster, W. (2009). When continents collide: Phylogeny, historical biogeography and systematics of the medically important viper genus *Echis* (Squamata: Serpentes: Viperidae). *Molecular Phylogenetics and Evolution*, *53*(3), 792–807. https://doi.org/10.1016/j.ympev.2009.08.002

R Core Team. (2021a). *R: A Language and Environment for Statistical Computing*. R Foundation for Statistical Computing. https://www.R-project.org/

~~R Core Team. (2021b). *R: A Language and Environment for Statistical Computing*. https://www.R-project.org/~~

Ranallo-Benavidez, T. R., Jaron, K. S., & Schatz, M. C. (2020). GenomeScope 2.0 and Smudgeplot for reference-free profiling of polyploid genomes. *Nature Communications*, *11*(1), Article 1. https://doi.org/10.1038/s41467-020-14998-3

Rhie, A., McCarthy, S. A., Fedrigo, O., Damas, J., Formenti, G., Koren, S., Uliano-Silva, M., Chow, W., Fungtammasan, A., Gedman, G. L., Cantin, L. J., Thibaud-Nissen, F., Haggerty, L., Lee, C., Ko, B. J., Kim, J., Bista, I., Smith, M., Haase, B., … Jarvis, E. D. (2020). *Towards complete and error-free genome assemblies of all vertebrate species* (p. 2020.05.22.110833). bioRxiv. https://doi.org/10.1101/2020.05.22.110833

Rhie, A., Walenz, B. P., Koren, S., & Phillippy, A. M. (2020). Merqury: Reference-free quality, completeness, and phasing assessment for genome assemblies. *Genome Biology*, *21*(1), 245. https://doi.org/10.1186/s13059-020-02134-9

Rokyta, D. R., Margres, M. J., Ward, M. J., & Sanchez, E. E. (2017). The genetics of venom ontogeny in the eastern diamondback rattlesnake (*Crotalus adamanteus*). *PeerJ*, *5*, e3249. https://doi.org/10.7717/peerj.3249

Russell, F. E., & Campbell, J. R. (2015). *Venomous terrestrial Snakes of the Middle East*. Edition Chimaira.

Saethang, T., Somparn, P., Payungporn, S., Sriswasdi, S., Yee, K. T., Hodge, K., Knepper, M. A., Chanhome, L., Khow, O., Chaiyabutr, N., Sitprija, V., & Pisitkun, T. (2022). Identification of *Daboia siamensis* venome using integrated multi-omics data. *Scientific Reports*, *12*(1), Article 1. https://doi.org/10.1038/s41598-022-17300-1

San-Jose, L. M., & Roulin, A. (2017). Genomics of coloration in natural animal populations. *Philosophical Transactions of the Royal Society B: Biological Sciences*, *372*(1724), 20160337. https://doi.org/10.1098/rstb.2016.0337

Schield, D. R., Card, D. C., Hales, N. R., Perry, B. W., Pasquesi, G. M., Blackmon, H., Adams, R. H., Corbin, A. B., Smith, C. F., Ramesh, B., Demuth, J. P., Betrán, E., Tollis, M., Meik, J. M., Mackessy, S. P., & Castoe, T. A. (2019). The origins and evolution of chromosomes, dosage compensation, and mechanisms underlying venom regulation in snakes. *Genome Research*, *29*(4), 590–601. https://doi.org/10.1101/gr.240952.118

Schield, D. R., Perry, B. W., Adams, R. H., Holding, M. L., Nikolakis, Z. L., Gopalan, S. S., Smith, C. F., Parker, J. M., Meik, J. M., DeGiorgio, M., Mackessy, S. P., & Castoe, T. A. (2022). The roles of balancing selection and recombination in the evolution of rattlesnake venom. *Nature Ecology & Evolution*, *6*(9), 1367–1380. https://doi.org/10.1038/s41559-022-01829-5

Schneemann, M., Cathomas, R., Laidlaw, S. T., El Nahas, A. M., Theakston, R. D. G., & Warrell, D. A. (2004). Life-threatening envenoming by the Saharan horned viper (*Cerastes cerastes*) causing micro-angiopathic haemolysis, coagulopathy and acute renal failure: Clinical cases and review. *QJM: An International Journal of Medicine*, *97*(11), 717–727. https://doi.org/10.1093/qjmed/hch118

Simão, F. A., Waterhouse, R. M., Ioannidis, P., Kriventseva, E. V., & Zdobnov, E. M. (2015). BUSCO: Assessing genome assembly and annotation completeness with single-copy orthologs. *Bioinformatics (Oxford, England)*, *31*(19), 3210–3212. https://doi.org/10.1093/bioinformatics/btv351

Šmíd, J., & Tolley, K. A. (2019). Calibrating the tree of vipers under the fossilized birth-death model. *Scientific Reports*, *9*(1), 5510. https://doi.org/10.1038/s41598-019-41290-2

Formatted: Font: Italic

Formatted: Font: Italic

Formatted: Font: Italic

Smith, C. F., Nikolakis, Z. L., Perry, B. W., Schield, D. R., Meik, J. M., Saviola, A. J., Castoe, T. A., Parker, J., & Mackessy, S. P. (2023). The best of both worlds? Rattlesnake hybrid zones generate complex combinations of divergent venom phenotypes that retain high toxicity. *Biochimie*. https://doi.org/10.1016/j.biochi.2023.07.008

Solovyev, V., Kosarev, P., Seledsov, I., & Vorobyev, D. (2006). Automatic annotation of eukaryotic genes, pseudogenes and promoters. *Genome Biology*, *7*(Suppl 1), S10. https://doi.org/10.1186/gb-2006-7-s1-s10

Suryamohan, K., Krishnankutty, S. P., Guillory, J., Jevit, M., Schröder, M. S., Wu, M., Kuriakose, B., Mathew, O. K., Perumal, R. C., Koludarov, I., Goldstein, L. D., Senger, K., Dixon, M. D., Velayutham, D., Vargas, D., Chaudhuri, S., Muraleedharan, M., Goel, R., Chen, Y.-J. J., … Seshagiri, S. (2020). The Indian cobra reference genome and transcriptome enables comprehensive identification of venom toxins. *Nature Genetics*, *52*(1), 106–117. https://doi.org/10.1038/s41588-019-0559-8

Tang, H., Bowers, J. E., Wang, X., Ming, R., Alam, M., & Paterson, A. H. (2008). Synteny and Collinearity in Plant Genomes. *Science*, *320*(5875), 486–488. https://doi.org/10.1126/science.1153917

Tang, H., Krishnakumar, V., Jingping Li, Tiany, MichelMoser, Maria, & Yim, W. C. (2017). *tanghaibao/jcvi: JCVI v0.7.5* (v0.7.5) [Computer software]. Zenodo. https://doi.org/10.5281/ZENODO.846919

Tang, S., Lomsadze, A., & Borodovsky, M. (2015). Identification of protein coding regions in RNA transcripts. *Nucleic Acids Research*, *43*(12), e78–e78. https://doi.org/10.1093/nar/gkv227

Tasoulis, T., & Isbister, G. (2017). A Review and Database of Snake Venom Proteomes. *Toxins*, *9*(9), 290. https://doi.org/10.3390/toxins9090290

Tempel, S. (2012). Using and Understanding RepeatMasker. In Y. Bigot (Ed.), *Mobile Genetic Elements* (Vol. 859, pp. 29–51). Humana Press. https://doi.org/10.1007/978-1-61779-603-6_2

Thongchum, R., Singchat, W., Laopichienpong, N., Tawichasri, P., Kraichak, E., Prakhongcheep, O., Sillapaprayoon, S., Muangmai, N., Baicharoen, S., Suntrarachun, S., Chanhome, L., Peyachoknagul, S., & Srikulnath, K. (2019). Diversity of PBI-DdeI satellite DNA in snakes correlates with rapid independent evolution and different functional roles. *Scientific Reports*, *9*(1), 15459. https://doi.org/10.1038/s41598-019-51863-w

Title, P. O., Singhal, S., Grundler, M. C., Costa, G. C., Pyron, R. A., Colston, T. J., Grundler, M. R., Prates, I., Stepanova, N., Jones, M. E. H., Cavalcanti, L. B. Q., Colli, G. R., Di-Poï, N., Donnellan, S. C., Moritz, C., Mesquita, D. O., Pianka, E. R., Smith, S. A., Vitt, L. J., & Rabosky, D. L. (2024). The macroevolutionary singularity of snakes. *Science*, *383*(6685), 918–923. https://doi.org/10.1126/science.adh2449

Uetz, P. (2021). The Reptile Database: Curating the biodiversity literature without funding. *Biodiversity Information Science and Standards*, *5*, e75448. https://doi.org/10.3897/biss.5.75448

Vitt, L. J., & Caldwell, J. P. (2014). *Herpetology: An introductory biology of amphibians and reptiles* (Fourth edition). Elsevier, AP, Academic Press is an imprint of Elsevier.

Vonk, F. J., Casewell, N. R., Henkel, C. V., Heimberg, A. M., Jansen, H. J., McCleary, R. J. R., Kerkkamp, H. M. E., Vos, R. A., Guerreiro, I., Calvete, J. J., Wüster, W., Woods, A. E., Logan, J. M., Harrison, R. A., Castoe, T. A., De Koning, A. P. J., Pollock, D. D., Yandell, M., Calderon, D., … Richardson, M. K. (2013). The king cobra genome reveals dynamic gene evolution and adaptation in the snake venom system. *Proceedings of the National Academy of Sciences*, *110*(51), 20651–20656. https://doi.org/10.1073/pnas.1314702110

Vyas, V. K., Brahmbhatt, K., Bhatt, H., & Parmar, U. (2013). Therapeutic potential of snake venom in cancer therapy: Current perspectives. *Asian Pacific Journal of Tropical Biomedicine*, *3*(2), 156–162. https://doi.org/10.1016/S2221-1691(13)60042-8

Walker, B. J., Abeel, T., Shea, T., Priest, M., Abouelliel, A., Sakthikumar, S., Cuomo, C. A., Zeng, Q., Wortman, J., Young, S. K., & Earl, A. M. (2014). Pilon: An Integrated Tool for Comprehensive Microbial Variant Detection and Genome Assembly Improvement. *PLOS ONE*, *9*(11), e112963. https://doi.org/10.1371/journal.pone.0112963

Formatted: Font: (Default) Times New Roman, 11 pt

Formatted: Indent: Left: 0", Line spacing: single

Formatted: Font: (Default) Times New Roman, 11 pt

Formatted: Font: 11 pt

Weinstein, S. A., White, J., Keyler, D. E., & Warrell, D. A. (2013). Non-front-fanged colubroid snakes: A current evidence-based analysis of medical significance. *Toxicon*, *69*, 103–113. https://doi.org/10.1016/j.toxicon.2013.02.003

Werren, J. H., Richards, S., Desjardins, C. A., Niehuis, O., Gadau, J., Colbourne, J. K., THE NASONIA GENOME WORKING GROUP, Beukeboom, L. W., Desplan, C., Elsik, C. G., Grimmelikhuijzen, C. J. P., Kitts, P., Lynch, J. A., Murphy, T., Oliveira, D. C. S. G., Smith, C. D., Zande, L. van de, Worley, K. C., Zdobnov, E. M., … Gibbs, R. A. (2010). Functional and Evolutionary Insights from the Genomes of Three Parasitoid Nasonia Species. *Science*, *327*(5963), 343–348. https://doi.org/10.1126/science.1178028

Westeen, E. P., Escalona, M., Holding, M. L., Beraut, E., Fairbairn, C., Marimuthu, M. P. A., Nguyen, O., Perri, R., Fisher, R. N., Toffelmier, E., Shaffer, H. B., & Wang, I. J. (2023). A genome assembly for the southern Pacific rattlesnake, *Crotalus oreganus helleri* , in the western rattlesnake species complex. *Journal of Heredity*, *114*(6), 681–689. https://doi.org/10.1093/jhered/esad045

Wickham, H. (2016). *ggplot2: Elegant Graphics for Data Analysis*. Springer-Verlag New York. https://ggplot2.tidyverse.org

Williams, D. J., Faiz, M. A., Abela-Ridder, B., Ainsworth, S., Bulfone, T. C., Nickerson, A. D., Habib, A. G., Junghanss, T., Fan, H. W., Turner, M., Harrison, R. A., & Warrell, D. A. (2019). Strategy for a globally coordinated response to a priority neglected tropical disease: Snakebite envenoming. *PLOS Neglected Tropical Diseases*, *13*(2), e0007059. https://doi.org/10.1371/journal.pntd.0007059

Wüster, W., Peppin, L., Pook, C. E., & Walker, D. E. (2008). A nesting of vipers: Phylogeny and historical biogeography of the Viperidae (Squamata: Serpentes). *Molecular Phylogenetics and Evolution*, *49*(2), 445–459. https://doi.org/10.1016/j.ympev.2008.08.019

Zancolli, G., Calvete, J. J., Cardwell, M. D., Greene, H. W., Hayes, W. K., Hegarty, M. J., Herrmann, H.-W., Holycross, A. T., Lannutti, D. I., Mulley, J. F., Sanz, L., Travis, Z. D., Whorley, J. R., Wüster, C. E., & Wüster, W. (2019). When one phenotype is not enough: Divergent evolutionary trajectories govern venom variation in a widespread rattlesnake species. *Proceedings of the Royal Society B: Biological Sciences*, *286*(1898), 20182735. https://doi.org/10.1098/rspb.2018.2735

Zancolli, G., Reijnders, M., Waterhouse, R. M., & Robinson-Rechavi, M. (2022). Convergent evolution of venom gland transcriptomes across Metazoa. *Proceedings of the National Academy of Sciences*, *119*(1), e2111392119. https://doi.org/10.1073/pnas.2111392119

Formatted: Indent: Left: 0", Hanging: 0.5", Line spacing: single

Personal Cover Click here to access/download;Personal Cover;reviews.docx

Dear Hongfang Zhang,

Thank you for considering our manuscript for review, as well as all the reviewer's comments. We have now incorporated such suggestions which have significantly improved the manuscript. Below we provide a point-by-point justification of such changes and we hope that this version is now fit for publication in *GigaScience*.

One last thing, two of the reviewers mentioned that they did not have access to some of the data, which was updated to GigaScience, could you please verify that? We would also like to upload a new supplementary file, as recommended by one of the reviewers.

Sincerely,
Gabriel Mochales Riaño

Reviewer #1: In the manuscript entitled 'Chromosome-level reference genome for the medically important Arabian horned viper (Cerastes gasperettii)', the authors assembled a high-quality chromosome-level reference genome for the Arabian horned viper (Cerastes gasperettii), a special Viperid species, which is an important data resource. Combined with multi omics data, the authors characterized the genome, conducted the analysis of toxin gene family, and identified a novel SVMP gene. The research is with great significance for the revelation of the origin and diversification of snake venom. Overall, I think the science and findings of the study are meaningful and merit publication, but in its current form, there are some issues should be noticed:

We thank reviewer 1 for his/her comments of our manuscript. We provide below a point-by-point answer to all his/her questions.

1. It should be noted that Fig. 1 and Fig. 2 both have unidentified border lines.

Sorry for that, we have now fixed it.

2. In all phylogenetic trees presented by the manuscript, it would be better for authors to indicate all species information.

The three toxin phylogenies have been modified and now all the tips include their species information.

3. I'm curious if the authors considered period differences in sampling, for example differences in venom glands after venom harvest or in the resting state, which could affect the analysis especially the transcriptome.

Before sampling the venom glands, venom was extracted, and snakes were allowed to recover for four days to maximize the venom gland transcription. This was absent from the text and now it has been added.

4. In the transcriptomics section, the author stated that the batch effect of CG1 was due to the low mapping of that sample to our reference genome. It is a misinterpretation to

me as CG1 itself is the genome sequencing sample. The authors should further explain for this.

RNA-seq sequencing was not produced at the same time for the three samples, we first sequenced the rna-seq samples from the reference genome individual and later on the two other samples. We do not find any explanation to this, but both the low mapping as well as the different clustering of the tissues hampers the analyses of the three samples together. For that, we decided to exclude it from the analyses. We have clarified it in the text.

5. The authors need to ensure that all data generated by the manuscript is accessible and information about the data is not currently available.

All the NGS data was uploaded to ncbi and all the non-NGS data was uploaded to GigaScience's internal repository. We will talk with the Editor to solve this issue, raised by several reviewers, we are sorry for that.

6. Please check the references to ensure that the formatting meets the publisher's requirements, e.g., some Latin names of species requiring italics.

This has been fixed now, thanks.

Reviewer #2: Mochales-Riaño et al. present a high-quality genome assembly for the Arabian horned viper and provide a suite of genomic analyses related to synteny, toxin gene evolution and expression, genomic diversity, and demographic history of this and related species. This species is a valuable addition to existing snake genome resources given its medical significance and the current underrepresentation of genomes for Viperidae. I also appreciate that the authors sequenced the heterogametic sex and successfully assembled both sex chromosomes. I do have a few questions and concerns about the manuscript in its current form that I highlight below. Most notably, I feel that the arguments throughout the manuscript about toxin gene copy number correlating with proteomic abundance to be poorly supported and generally problematic given the data and analyses that the authors present. I suggest that the authors reevaluate these claims, and either provide additional analyses in an effort to support these claims or otherwise remove them from the manuscript, as I don't think they are ultimately crucial to the value of this genome report.

We thank reviewer 2 for his/her comments of our manuscript. We provide below a point-by-point answer to all his/her questions.

Introduction:
I find the argument being made in the sentence beginning "Previous works have shown that changes in gene regulation" a bit confusing. Rather than this arguing that studying the expression of venom genes is "insufficient," I think that this instead argues that transcriptomic and proteomic data are critical for studying venom in conjunction with annotated genome sequence. You could for example have a species with 20 copies in a

particular tandem array, but only two of them are ever expressed at biologically meaningful levels and thus contribute proteins to the excreted venom. Knowing both the total number of copies in the genome and the number that are actually contributing to the venom proteome are both valuable and necessary for understanding the evolution of that gene family, its role and significance in venom phenotypes, etc.

We agree with reviewer 2 about this point and we have accordingly changed this section of the introduction to highlight how the combination of these techniques are necessary to study the evolution of venom. Now the paragraph reads like this: "Previous works have shown that changes in gene regulation can result in the activation and deactivation of venom-coding genes at all taxonomic levels and within the same individual (Avella et al., 2022; Hogan et al., 2024; Margres et al., 2021; Zancolli et al., 2022), suggesting that transcriptomic and proteomic data are critical for studying venom in conjunction with well annotated reference genomes to disentangle the complete number and biochemical nature of the toxins an individual can potentially transcribe (Drukewitz & Von Reumont, 2019). Ultimately, the study of venom genomics may yield insights into antivenom or drug discovery, as it can identify unexpressed toxin-coding genes that target specific physiological pathways, potentially leading to new therapies for human illnesses including but not limited to cancer (Casewell et al., 2013; King, 2011; L. Li et al., 2018)."

I'm also not sure I follow the logic of the next sentence. Why exactly would the identification of specifically "unexpressed" toxin genes be particularly notable for antivenom, drug discovery, therapeutics, etc.?

We have modified this sentence, now it reads like this:

"Ultimately, the study of venom genomics may yield insights into antivenom or drug discovery, as it enables the identification of unexpressed toxin-coding genes. These genes, often overlooked by transcriptomic or proteomic approaches unless ontogeny analyses or in-depth venom expression studies are performed, may target unique physiological pathways. Such discoveries could lead to novel therapies for human illnesses including but not limited to cancer."

"We deciphered numerous genomic attributes of this species including its genetic diversity and failed to find evidence of inbreeding" - lack of inbreeding is never discussed in the context of the heterozygosity results, but is pitched here as a major result of the paper. Did the authors have a priori expectations regarding inbreeding in this species?

It is true that we did not have a clear expectation of the genomic status. For this reason, we have now changed it to this:

“We deciphered numerous genomic attributes of this species, highlighting its adequate levels of genetic diversity”

Methods:
"Gene counts per gene…" - should this be "Gene expression counts per gene…"?

Reviewer 2 is right, we have included the word expression in this sentence, thank you for pointing it out.

Venom gland RNA-seq data was generated from three animals, but proteomic data was generated from a pool of two other animals. This is not ideal for linking gene expression to venom proteome composition, where you really would want venom collected from the same animals you are getting venom gland RNA from. This is especially true is there is intraspecific variation in venom phenotypes within this species. The latitude and longitude are not provided for the two proteome samples. Were these collected from the same latitude and longitude as the RNA-seq animals?

Intraspecific venom variation has not been described for this species, meaning that reviewer 2 is right and we should consider this. However, we were not able to extract venom from the reference individual and we do not have the exact coordinates for the two proteomic individuals. Although both individuals are from the same country as the reference genome, we have added that possible differences between transcriptomic and proteomic venom expression may rise due to geographical variation.

For analyses of heterozygosity, the authors map wgs data from diverse species against the cerastes reference and call variants. Why was this approach chosen over instead mapping the data for each species to either that species' reference (i.e., C. viridis and N. naja) or a more closely related species for those without a reference? Presumably that would reduce the potential influence of reference bias on these estimates of heterozygosity?

Yes, reviewer 2 is right and we may have reduced the heterozygosity levels of those species. We have reanalyzed each sample mapping it with the closest reference genome possible.

Results:
"Toxin genes usually found in venomous snakes (see proteome results below) were mainly found on macrochromosomes, although major toxin groups were found on microchromosomes (SVMPs, SVSPs and PLA2; Fig. 1)" this feels a bit contradictory. Maybe just can state that toxin genes were found on both macro and microchromosomes?

We have modified this section accordingly. Now it reads:

“Toxin genes were found in both macro- and microchromosomes (Fig. 1).”

"Finally, we also found a battery of 3FTxs and myotoxin-like genes, but they were not represented in our RNA-seq dataset (see below)." The authors do not further discuss this result as implied by "(see below)," unless that was simply referring to subsequent discussion of RNA-seq data. From what I can tell, these are also not present in the proteomic data, correct?

Exactly, we did not detect neither transcripts nor proteomic data for those two gene groups. We have now clarified it in the text.

"The venom gland transcriptome contained a total of 7,237 genes expressed (TPM > 500), including a total of 65 putative toxin genes. Differential gene expression analyses revealed a total of 161 genes (33 putative toxin genes) that were differentially upregulated (FC > 2 and 1% FDR) in venom glands compared to other tissues (Fig. 3A)." Figure 3A only shows 10 toxin genes with "unique" expression in the venom gland, not the 161 upregulated toxin genes as implied here. The authors should add a heatmap with these 161 genes to the supplement, if not to Figure 3 (guessing it might not fit).

Thanks for pointing that out, we have added now two extra supplementary heatmaps (Fig. S6 and S7), one including the upregulated genes in the venom gland and a second one only focusing on the toxins.

Fig 3: The authors do not discuss the lack of unique/upregulated expression evidence for PLA2s and Disintegrins in Fig 3A, despite their contribution to protein composition in Fig 3B. For disintegrins in particular, they represent a higher proportion of the venom proteome than CTLs and CRISPs, yet there is no evidence presented for high expression in these genes. What do the authors think is going on here? Could this be a technical issue related to the processing of the RNAseq data, perhaps related to the small size of these genes? Alternatively, could this be indicative of a mismatch between venom phenotypes of the animals used to generate transcriptomic versus proteomic data?

We agree with the reviewer that we did not discuss some of the differences between RNA-seq and proteomic datasets, possibly (as suggested) due to individual differences. We have now included it along the text.

In the text, the authors state "These genes, together with other SVMPs, SVSPs, Disintegrins (DISI) and C-type lectins (CTL), were highly expressed in the venom gland and form the core toxic effector components of the venom" but again there is no presented evidence for DISI expression in particular. Are these genes included in the 161 upregulated genes in the venom gland?

This sentence was referring to the highly expressed genes in the venom gland, and as the reviewer mentions, DISI are not there. DISI has been removed from the sentence, thank you for pointing that out.

The authors only present proteomic data in the form of a pie chart of overall composition grouped by toxin family (Fig 3B). Does the proteomic data generated here provide individual gene-level proteomic abundance estimates? If so, this would be valuable to include, especially in support of the authors claims about gene copy number being correlated with protein abundance. For example in Figure 3, SVMP9 and

SVMP10, and to a lesser extent SVMP13, are highly expressed and therefore possibly/likely the major contributors to SVMPs in the proteome. Is the SVMP section of the pie chart in Fig 3B dominated by proteins from these 3 genes?

We have now added one supplementary table (Table S4) with the values of figure 3B and a supplementary file including the proteomic abundance estimated at the gene-level. The claim about gene copy number being correlated with protein abundance is discussed below.

"We studied venom evolution within the most abundant toxin groups (i.e., SVMPs and SVSPs, as well as PLA2)." PLA2s are a relatively low proportion of the venom proteome in Fig 3B, and are not present in the expression heatmap in Fig 3A. Why were these chosen for further investigation over CTL, CRISP, DISI, etc.?

The reviewer is correct that the justification for including PLA2s in our comparative analyses is not very robust, as several other toxin families were found to be more abundant. However, genetic and genomic resources for PLA2s within snakes are more abundant than for other toxin families, paving the way to the study of this toxin family.

"The amplification of SVMP copy numbers is consistent with proteomic results, as SVMPs were the second most abundant component…". Related to my comment above, are all/many of these copies expressed in proteomic, or at least transcriptomic, data? As the data is currently presented, it appears that a small number of SVMPs are highly expressed and thus likely contributing to the proteome. This does not support, and might in fact contradict, the authors claim that there is an association with increased copy number and contribution to the proteome.

Regarding this fact, what we meant to say is that the toxin families that have undergone the highest number of gene duplication events throughout their evolution are also the most abundant in the proteome, regardless of whether all the genes in that toxin family are expressed or not. However, we agree with the reviewer that this statement could cause confusion, and therefore we have decided to remove it as well as other references for this claim along the text.

Related to this, and more generally, the authors do not present a convincing argument for the relationship between gene copy number and the resulting percentage of a given toxin gene family in the proteome. If copy number is directly related to the resulting amount of a toxin in the proteome, the authors would need to show that many/all of those copies are expressed in the transcriptomic data, and that proteins produced from those genes are present and contributing to the venom proteome (beyond just the total percentage for the family). Further, making any links between copy number and percent overall composition in the proteome is problematic, because it inherently is impacted by copy number variation and expression of all the other toxin genes. You could, in theory, have copy number expansion in a species where all the genes are expressed and contribute to the proteome, but no overall change in the percent of that toxin family in the proteome if other toxin families have also expanded and/or are expressed more highly. Related to this, there is currently no obvious baseline to compare against in order to make these claims that expansion has resulted in higher venom proteome composition (i.e., a situation where we have fewer

SVMP gene copies and a corresponding lower percentage of SVMP proteins in the venom proteome). This would potentially require comparison across species and/or populations with differing copy number, etc.

Thank you for this explanation, now it is clearer your specific point. We are aware that our data does not support this claim, and for this reason, we have decided to remove it from the manuscript.

My concerns above also apply to the interpretation of SVSP results: "The high number of SVSP genes found (although lower than in Crotalus adamanteus) were in line with the proteomic results, as SVSPs are the most abundant toxin in the proteome (Fig. 3B)." Further, C. adamanteus has a larger number of SVSP genes than C. gasperettii, yet a lower percent composition of SVSPs in the proteome (Margres et al. 2014), emphasizing my concerns about associating copy number and percent composition.

We agree with the reviewer and, as mentioned earlier, we have removed these claims from the manuscript. Thanks again for pointing it out.

Could the two large Group 2 SVSPs in Fig 4E be misannotations of multiple genes? Looking at the adamanteus genes above these, there genes starting and ending at roughly the same position the start and end of these large SVSPs, making me wonder if there are multiple cerastes genes that were annotated as one. In my own experience, I have seen similar situations where FGENESH+ was fed a large region containing multiple genes and annotated multiple genes together as one, so might just be worth double checking that that hasn't happened here. Alternatively, could these be gene fusions? If that's the case, that would presumably complicate the gene tree analyses, correct? i.e., these genes would probably need to excluded from those analyses.

The reviewer is right, thank you very much for spotting this. We have reannotated the region and we have identified the different genes. The different figures, as well as the annotation has been modified accordingly.

Reviewer #3: Dear Authors, thank you for compiling this resource and the manuscript. I apologise for the delay in my review. I have read the manuscript with great interest. I have some major concerns that need be addressed and a lot of minor concerns. Without line numbers, it was difficult to provide comments. I have chosen to write the part of the sentence that my comment refers to for you to consider for improvements.

We thank reviewer 3 for his/her comments of our manuscript. We are sorry for the lack of line numbers, they have been added now. We provide below a point-by-point answer to all his/her questions. We appreciate the thorough review provided, specially the minor concerns.

Major concerns:

- Abstract can include quantitative values for some key results such as the genome size, contiguity (e.g.N50, L90) and quality metrics (e.g. BUSCO) of the genome assembly among other result claims listed in the abstract.

We have now added quantitative metrics for the reference genome within the abstract.

- Venom as the keyword can perhaps be described/defined. Authors interchangeably use "venom", "toxin", "venom toxin", genes coding venom proteins. I strongly suggest the use of consistent terminologies that are well defined in the manuscript.

We have added venom as a keyword and we have carefully revised the manuscript regarding the use of the words venom, toxin, venom toxin and genes coding venom proteins, thanks for pointing that out.

- Methods need elaborate descriptions about reagents, procedures including for library preparations, sequencing machines, library kits and versions, etc. These are relevant for downstream analyses.

These details have been included, specially following the minor concerns suggested by the reviewer below. We hope it is more clear now.

- For all software, list parameters used, even if default, then explicitly state that "default parameters were used". For all software, list version numbers used for analyses.

We have incorporated parameters and version for the softwares.

- Authors are urged to change "macorsynteny" and "microsynteny" terms to chromosome level and local synteny analyses. This is to avoid confusion related to macro/microchromosomes.

We have accordingly modified the text with regard to both terms.

- "Genomic diversity" analyses use cross-species alignments and variant calling using software and methods developed for same species data. This can introduce significant bias in downstream interpretation and use of the variant data (heterozygosity measure may be). I suggest removal of this section because of lack of accuracy.

This concern was also raised by other reviewers, and we have decided to analyze each species using the closest available reference genome. This approach aims to minimize, as much as possible, the expected reduction in heterozygosity levels associated with mapping to a more distantly related species.

- Discussion of new discovery is largely lacking. I would appreciate if authors contextualized their results with other discoveries in the field.

We have have increased that section, now it reads like this:
"Our discovery of a novel SVMP gene in *C. gasperettii* adds to the growing body of work on the dynamic evolution of venom systems. Similar gene expansions and duplications have been observed in other species, such as PLA_2 toxin-coding genes found in the venom of *Azemiops feae* (Myers et al., 2022), highlighting the lineage-specific nature of venom evolution. The gene we identified, possibly arising from an *SVMP13* duplication, do not share orthology with genes in other species, suggesting the presence of hidden toxin diversity in venom systems"

- **Section headings in Results and Discussions can be changed to reflect main findings instead of "transcriptomics" or "genomic diversity".**

Titles have been changed, thank you for the suggestion.

- **One of the main findings is about SVMP gene family expansion. However, due to the lack of evidence about assembly accuracy in the region, accurate annotation of copies, and the effect of studying "primary assembly" instead of "haplotype assembly" at this region, I am not convinced of claims made in the paper. Appropriate justification is required for this section.**

It may be that there was a misunderstanding regarding the used terms. We did work with haplotypic assemblies, as we obtain them directly from the assembler when incorporating the linkage data. When we mention primary assembly is in fact to remove duplicates present in the primary assembly to transfer them to the secondary assembly, but we do not work with an assembly that combines both haplotypes, this has been specified in the text.
Regarding the assembly accuracy of the region is as high as the current technologies allows it, and as proven by the metrics of the genome, our genome shows contiguity metrics similar to other high-quality reference genomes such as the recently published in PNAS by Hogan et al., (2024), with venom-coding genes assembled in single contigs. Regarding the accurate annotation of the copies, as stated in the manuscript we manually curated the venom regions combining both empirical and manual annotations.

- **The nomenclature of SVMP genes is confusing. For example, In Figure 4A, they are all labelled as SVMPs with different colours, but then they are labelled as MDCs and MADs in Figure 4b and Supp Figure 6. Please label each gene in each species with consistent names that can reflect orthologous relationship. This is hard to discern, especially without appropriate species labels in Supp Figure 6.**

We have modified the supplementary figures, highlighting in bold the genes involved in Fig. 4, so we hope this makes it easier to spot the orthology between groups. Moreover, now the supplementary figures contain appropriate species labels.

- **Provide MSA files and trees used to infer evolutionary history. In the absence of the sequence alignments, and raw tree file, I am unable to evaluate this section of the manuscript. Please provide all required details for reviewers and readers.**

All the NGS data was uploaded to ncbi and all the non-NGS data was uploaded to GigaScience's internal repository. We will talk with the Editor to solve this issue, raised by several reviewers, we are sorry for that.

??: It is not clear what authors mean by the word, term, phrase. Please correct them to convey accurate meaning using established and accepted scientific terminologies and English conventions.

Minor concerns:

Abstract:
- **"compousing" ??**
- **"highly expressed toxin genes": in what tissues?**
- **"genome-wide diversity" ??**
- **"toxin gene families in venomous species" -> "toxin gene families in venomous snake species"**

All these suggestions within the abstract have been modified, thanks.

Background:
- **"Such advances in sequencing technologies": remove "Such"**

We have removed it

- **"depending on their type, interactions, and the organism": interactions with what?**

The word interaction was changed.

- **"proteomic (and transcriptomic) approaches": remove parenthesis**

We have removed it.

- **"to new therapies for human illnesses including but not": since the title contains "medically important", it would be great to include some specific examples here from the literature.**

We have incorporated new literature citing examples about it.

- **"However, venomous snakes are one": remove "However"**

We have removed it.

- **"therefore, the fundamental model system": change "fundamental" to "useful"**

We have changed it.

- **"of medical importance by the World Health Organization (WHO) due to their": provide citation**

We provided a citation.

- **"Within venomous snakes, the most medically": restructure the sentence for brevity and clarity.**

We have modified the sentence.

- **"cytotoxic effects (among others)": remove "(among others)"**

We have removed it.

- **"conducted using a proteomic approach": clarify what proteomic approach mean here.**
- **"Hirst et al., (in review);" : remove this citation**

We have added it as citation as it is already available.

- **"within the Viperidae family posses an available reference": change the word "posses" to something meaningful**

We have changed it to contains.

- **"Moreover, employing several -omics techniques": be specific about techniques**

We have specified the techniques used.

- **"We deciphered numerous genomic attributes": be specific**

We have changed it to "We deciphered its adequate levels of genetic diversity."

Methods:
- **Describe how blood was extracted from animals with all details including animal handling techniques, body part etc.**

We describe it now.

- **"was stored in RNAlater until RNA extraction": source for RNAlater**

It is from a company, we have specified it

- **"We extracted gDNA from the blood of a female individual": provide additional details such as the quantity of blood used, thawing process, qty of reagents, especially elution buffer etc. Manufacturer protocols may be suited best for mammalian blood (humans, mice) without nucleus in RBCs unlike snakes.**

No modifications were added to the protocol, but we have clarified it.

- **"Then, we sequenced a total of two 8M SMRT HiFi cells, aiming for a ~30x of coverage, at the University of Leiden": provide details of library preparation, sequencing machine etc.**

We have provided additional information.

- "(including venom glands, tongue, liver and pancreas, among others": Either list all or refer to the table.

We refer now to the table.

- "RNA libraries were prepared with the VAHTS": Was the library and sequencing strand specific? Provide complete details on these processes.

It was strand specific, now it is specified.

- "8M SMRT HiFi cell containing two Iso-seq HiFi libraries": use correct names of these and also include sequencing machine details.

We have changed the sentence as well as indicate the sequencing details.

- "Quality control on HiFi and Illumina reads was assessed using FastQC": correct the phrasing of this sentence

This has been corrected.

- "To make an initial exploration of the genome, …..we generated a k-mer profile with Meryl": Explicitly state the purpose of this analysis.

We have specified it: "In order to initially explore the genome size, heterozygosity levels and coverage data".

- "Manual curation was performed with Pretext": cite Pretext properly. Explain decisions of this manual curation. i.e. what evidence was used to join or break contigs.

Pretext does not have any scientific publication(or at least we did not find it), but we have provided the website.

- "Then, we ran three iterative rounds of RepeatMasker to annotate the known and unknown elements identified by RepeatModeler and soft-masked the genome for simple repeats": break this sentence into two and explain reasons for running RepeatMasker three times.

This has been specified.

- "We used GeMoMa v.1.9": Include all details about the annotations. This sentence is not sufficient for reproducibility. Were the RNAseq data assembled or provided as raw files to GeMoMa. How were they mapped to the genome assembly f

Details are included now.

- "published: Anolis carolinensis from Alföldi": Remove the word "from" here as citation is sufficient. Provide details of assembly versions, annotation version, database of annotations etc.

From was removed.

- "Crotalus ruber from Hirst et al., (in review)": remove this citation or list it as personal communication

As above, this has been included as the paper has been recently published.

- "We previously quality checked and removed the adapters of the RNA-seq data": remove "previously" and provide details on how adapters were removed from RNAseq data

We removed previously and provided information about how we removed the adapters with fastp.

- "also removed the adapters for the Iso-seq data": Explain how this was performed.

Same as above.

- "We blast our ..": Change all occurrence of "blast" to "BLAST" and specify parameters, if it was BLASTN or BLASTP or something else. This is not clear at all.

We have changed it and specified it.

- "we performed additional annotation steps for venom genes.": Details are not complete for reproducibility. State explicitly what decisions were made and how gene structure was determined. This is the main part of the paper and does require accurate details.

Details are following this sentence, we modified it and we hope now it is more clear.

- "Whole-genome synteny was explored between": synteny by definition refers to being on the same string/chromosome. Therefore whole-genome synteny as a term doesn't make sense given that genome is divided into chromosomes. Revise it to say "chromosomal synteny"

This has been changed.

- "chromosomes assembled in the reverse complement, which were corrected using SAMtools faidx": samtools faidx cannot do this. Explain how this was done.

We used samtools faidx with reverse-complement and mark-strand options.

- "After adapter trimming and quality control, we mapped our RNA-seq reads": how were adapters trimmed and QC implemented.
- "Gene counts per gene": change gene counts to read counts

This has been clarified now.

- "Differential expression analyses were carried out": requires additional details

such as filters applied for the count, groups compared, statistical model, multiple testing correction methods.

This has been now added: “Prior to analysis, genes with fewer than 10 counts across all samples were filtered out. For comparisons, we defined two groups: venom glands versus all other tissues. DESeq2 employs a negative binomial generalized linear model to estimate differences in gene expression, and the p-values were adjusted for multiple testing using the Benjamini-Hochberg method to control the false discovery rate (FDR). Genes with an adjusted p-value < 0.01 and a fold change > 2 were considered significantly differentially expressed.”

- **"characterize the venom arsenal of Cerastes gasperettii": change the arsenal word.**

Changed**.**

- **"Fragmentation spectra were matched against a customized database including the bony vertebrates taxonomy dataset of the NCBI non-redundant database": revise for accuracy**

This has been revised.

- **"Unmatched MS/MS spectra were de novo sequenced": spectra were sequenced how??"we used blast, incorporating both toxin and non-toxin paralogs": change blast to BLAST and provide additional details about the tool used**

This was chaned and we provided additional details about it.

- **"Then, we aligned those regions using Mafft (Katoh": provide coordinates of these regions for future research in each assembly**

Coordinates are now provided.

- **"history for the main groups of toxins (i.e.,": parenthesis is not closed.**

We have now closed it.

- **"we also included other non-toxin paralogous genes from nontoxic species (for details about this see Supplementary Information": where do I look into the supplementary information? Be very clear. Provide coordinates of regions that were compared.**

Supplementary information was provided to GigaScience. We already have told them that reviewers were not able to access the data.

- **"When needed, we translated CDS": when was this needed? Explain.**

Now it is explained: “When nuclear sequences were obtained”.

- **"built a phylogeny for each of the toxin groups using Phyml": I presume that this is done with translated CDS sequences in toxin genomic regions. Please clarify.**

Yes, we have clarified it.

- **"Heterozygous positions were obtained from bam files with Samtools v1.9": provide details as to how this was done. Samtools doesn't have features to operate at a site level and therefore I am confused.**

We used the mpileup function of samtools, now it is clarified.

- **"Filtered reads were mapped against the new reference genome of Cerastes gasperettii using the bwa mem algorithm": bwa mem is designed for same species comparisons. Here you have used it for cross-species. Provide justification and perhaps biases it may have introduced for distantly related species.**

We have mapped the other species to closer reference genomes and every sampled was handled independently. This is now clarified.

- **"SNP calling was carried out …": This is not appropriate as models assume same species data. You have used cross-species alignments, which can be highly biased.**

Same as above, thanks for pointing it out.

Results and Discussion:

- **"PacBio HiFi (~40x), Hi-C (~60x) and Illumina data (~78x)": change to number of base pairs. 40x for a genome of 2GB is 80GB data and for genome of 1GB size, it is 40GB data. Before sequencing and assembly, the genome size cannot be known.**

This has been changed to: "PacBio HiFi (65 Gbp of data), Hi-C (96 Gbp of data) and Illumina data (135 Gbp of data)".

- **"After manual curation, we enhanced the scaffolding parameters of our genome": what was done as manual curation. Please specify.**

It is an intrinsic outcome of the manual curation, we have modified it for a better understanding.

- **"~228 times more contiguous than the Anolis sagrei genome": how is 228 more measured. How is this useful as a metric without the known ground truth. Assemblies can and do have errors.**

It is true that assemblies have errors and they are not complete, but the contiguity value is a comparable metric between genomes, so one genome can be 228x more contiguous than another one, although it is not complete.

- **"27,158 different protein-coding genes within our assembly": this seems large compared to other species. Can you elaborate or compare these numbers with other species.**

We have recently published a gecko reference genome (Burriel-Carranza et al., 2024 Mol Ecol) with a total of 39,360 protein-coding genes, compared to which other species it is a large number?

- **"Toxin genes usually found in venomous snakes (see proteome results below) were mainly found on macrochromosomes, although major toxin groups were found on microchromosomes (SVMPs, SVSPs and PLA2; Fig. 1)." : please revise this statement. Two part of the sentence are saying opposite things. Second provide coordinates of these genes as GFF/BED file as supplementary file with their exon structure annotations for others to reuse this information.**

This was modified, as also indicated by another reviewer, coordinates are also provided in a previous comment of the same reviewer.

- **"showed a great level of similarity between Cerastes gasperettii and Crotalus adamanteus": provide quantitative metrics for "great" level of similarity.**

This sentence has been now modified.

- **"we found several fission events in the A. sagrei genome,": Since A. sagrei genome is not contiguous and chromosome scale, you cannot infer fissions as it may be artefact of non-contiguous assembly. If that is not the case, provide evidence of this.**

Anolis sagrei genome is at chromosome level as indicated in the publication https://www.nature.com/articles/s42003-022-04074-5

- **"The last four…": Belongs in methods**

This has been now moved to methods.

- **"Macrosyntenic differences between lizards and snakes": this is very superficial discussion point. Please remove it or strengthen it with evidence.**

We removed it, as it is not the point of the analyses or the paper.

- **"Heatmap analyses with the most 2,000": Revise this statement. It doesn't make sense. E.g. Heatmap is a visualisation technique and not analyses method.**

Thanks, we have removed it.

- **"We studied venom evolution within the most abundant toxin groups": rewrite the sentence for clarity and brevity.**

We have modified the sentence.

- **"After a thorough manual curation": Explain what was this manual curation process clearly and the purpose of it.**

This was explained in materials and methods

- **"contiguous tandem repeat SVMPs for": Change "repeat" to "array" because tandem repeat has a different meaning in genomics research context.**

Array is more acurate, thanks for the suggestion.

- **"flanked by the NEFL and NEFM": Unclear if they are both 5' or 3' of toxin genes. Clarify**

This has been clarified.

- **"Microsyntenic analyses showed": change to local synteny**

This has been chaned along the text.

- **"gene copy number variation between": Since these are duplicate copies, clearly state how gene copies were identified. Include details of open reading frames, exon structures, pseudogene status, etc**

Gene copies were identified through phylogeny methods, this has been now clarified in the text.

- **"we can see an expansion in": Describe number of new copies, their status as intact or not, and sequence similarity between copies. Provide evidence that there is no false duplication due to heterozygous allele collapse in the assembly.**

We provide details about it now: "*Crotalus adamanteus* (22 copies unique to vipers and 10 lineage-specific copies) but also in *Cerastes gasperettii* (12 copies unique to vipers and one lineage-specific copy)"
Regarding the false duplication due to heterozygous alleles, this was already discussed above. During the assembly process, we removed duplicates from the primary assembly.

- **"More genomic data will indicate if SVMP12": Did you mean SVMP13?**

No, we mean SVMP12, as it is the new discovered gene.

- **"This difference may be expected, as PLA2 only represents around 5% of the proteome for Cerastes gasperettii": This is not true. Proteome doesn't equal to genome in some cases and superficial inference such as this is not warranted.**

We agree with the reviewer this sentence has been removed.

- **For PSMC analyses, please discuss the effect of mutation rate and generation time.**

We have added this sentence: "PSMC results may vary depending on the generation time as well as the mutational rate specified. The absence of species-specific data for this analyses may bias our results, although it is a general consensus in the literature when inferring these demographyc analyses in snakes (e.g. Schield et al., 2019)."

Figures:

- **Figure 1: Add y-axis scales to the circos plot.**

This has been added now.

- Figure 1b legend says it is a linkage map, but looks more like HiC contact map. Please edit.

We have now changed it, thanks.

- Figure 1b legend also says "including the sex chromosomes", which is not consistent with the circos plot.

During the HiC contact map, chromosomes were sorted by size, because it was not until the end that we identified the sexual chromosomes and in the final assembly they were moved to the end, for this reason there is this inconsistency.

- Figure 3A refers to transcriptome and 3b to proteome. Please make this very clear.
We have clarified it, thanks.

- Figure 4A, C and E, label genes consistent with the phylogenetic trees in supplementary figures so readers can know their genomic arrangements.

We have underscored the genes used in Figure 4A, C and E within the supplementary figures, because Figure 4 is already complex and labelling them would make it even more complex.

- Figure S4: Discuss why CG1 sample separates from rest of the samples. Seems like a batch effect.

We have added that information, thank you.
