# Supplementary material for: Chromosome-level reference genome for the medically important Arabian horned viper (Cerastes gasperettii)
Source: Gigascience. 2025 Jun 6;14:giaf030. doi: 10.1093/gigascience/giaf030 (PMC12143202; doi:10.1093/gigascience/giaf030)
Supplement: giaf030_GIGA-D-24-00269_Revision_2 [file giaf030_giga-d-24-00269_revision_2.pdf]

GigaScience

Chromosome-level reference genome for the medically important Arabian horned viper (Cerastes gasperettii)

--Manuscript Draft--

**Manuscript Number:**	GIGA-D-24-00269R2
**Full Title:**	Chromosome-level reference genome for the medically important Arabian horned viper (Cerastes gasperettii)
**Article Type:**	Research
**Funding Information:**	Ministerio de Ciencia e Innovación (PID2021-128901NB-I00) — Dr. Salvador Carranza<br>Departament de recerca i Universitats (2021-SGR-00751) — Dr. Salvador Carranza
**Abstract:**	Venoms have traditionally been studied from a proteomic and/or transcriptomic perspective, often overlooking the true genetic complexity underlying venom production. The recent surge in genome-based venom research (sometimes called “venomics”) has proven to be instrumental in deepening our molecular understanding of venom evolution, particularly through the identification and mapping of toxin-coding loci across the broader chromosomal architecture. Although venomous snakes are a model system in venom research, the number of high-quality reference genomes in the group remains limited. In this study, we present a chromosome-resolution reference genome for the Arabian horned viper (Cerastes gasperettii), a venomous snake native to the Arabian Peninsula. Our highly-contiguous genome allowed us to explore macrochromosomal rearrangements within the Viperidae family, as well as across squamates. We identified the main highly-expressed toxin genes compousing the venom’s core, in line with our proteomic results. We also compared microsyntenic changes in the main toxin gene clusters with those of other venomous snake species, highlighting the pivotal role of gene duplication and loss in the emergence and diversification of Snake Venom Metalloproteinases (SVMPs) and Snake Venom Serine Proteases (SVSPs) for Cerastes gasperettii. Using Illumina short-read sequencing data, we reconstructed the demographic history and genome-wide diversity of the species, revealing how historical aridity likely drove population expansions. Finally, this study highlights the importance of using long-read sequencing as well as chromosome-level reference genomes to disentangle the origin and diversification of toxin gene families in venomous species.
**Corresponding Author:**	Gabriel Mochales Riaño<br>Institute of Evolutionary Biology: Institut de Biologia Evolutiva<br>barcelona, Barcelona SPAIN
**Corresponding Author Secondary Information:**	
**Corresponding Author's Institution:**	Institute of Evolutionary Biology: Institut de Biologia Evolutiva
**Corresponding Author's Secondary Institution:**	
**First Author:**	Gabriel Mochales Riaño
**First Author Secondary Information:**	
**Order of Authors:**	Gabriel Mochales Riaño<br>Samuel R. Hirst<br>Adrián Talavera<br>Bernat Burriel-Carranza<br>Viviana Pagone<br>Maria Estarellas<br>Theo Busschau

Powered by Editorial Manager® and ProduXion Manager® from Aries Systems Corporation

	Stéphane Boissinot
	Michael P. Hogan
	Jordi Tena-Garcés
	Davinia Pla
	Juan J. Calvete
	Johannes Els
	Mark J. Margres
	Salvador Carranza
**Order of Authors Secondary Information:**	
**Response to Reviewers:**	Dear Hongfang Zhang,<br><br>Thank you for potentially accepting our manuscript. We have now incorporated the indicated suggestions by the reviewers. Below we provide a point-by-point justification of such changes and we hope that this version is now fit for publication in GigaScience.<br><br>Sincerely,<br>Gabriel Mochales Riaño<br><br>Reviewer #1: The author basically addressed my concerns that the manuscript could be published. There are still unidentified border lines in Fig. 1 only, but this could be a typographical problem?<br><br>We thank again reviewer one for his/her suggestions. Regarding the border's lines, we think it may be an artifact when the pdf is created, as we do not seen them in our figure.<br><br>Reviewer #2: The authors have conducted substantial revisions and I thank them for addressing my concerns. While I am generally satisfied with the revised version of the manuscript (in particular the removal of claims relating gene copy number to composition), I have a few minor, but important suggestions listed below. Most importantly, I think the authors need to be more explicit about the limitations resulting from the RNAseq and proteomic data being collected from separate animals which may or may not exhibit distinct venom phenotypes.<br><br>We thank again reviewer 2 for his/her thorough comments and suggestions along the text. We are sorry for not being clear enough regarding the limitations of having RNA-seq and proteomic data from different individuals, we have now included these sentences in line 392, in the section "toxins uniquely expressed in the venom gland" within results and conclusions:<br><br>"Transcriptomic and proteomic data was obtained from different individuals which may exhibit phenotypic differences in the venom due to several factors such as local adaptation or geographical isolation. We encourage future research to explore this possibility."<br><br>129: "We deciphered its adequate levels of genetic diversity" - I appreciate the authors addressing my concern in the original draft, but feel that this new sentence reads a bit vague. Perhaps something like "We assessed genetic diversity in comparison with other venomous snakes" or something like that?<br><br>Thanks for the suggestion, we have now changed it.<br><br>Line 396-398: Please be more explicit about the limitations of these comparisons by stating that you do not have gene expression and proteomic data from the same glands.

	We have now added a sentence regarding this.<br><br>Related to this, I expressed concern in the initial review about the mismatch between RNA and proteomic data in Figure 3 for PLA2 and Disintegrins in particular, as they are large portions of the proteome but are not expressed highly in RNAseq data. Again, I think the authors should be explicit by highlighting this and any other major discrepancies between the RNA and proteomic data that may be driven by not sampling both from the same glands. This is important to highlight and will prevent confusion for the reader.<br><br>We have now highlighted these differences, thanks for pointing it out:<br>"However, transcriptomic and proteomic data for the same individuals should be sampled, as there are several mismatches between RNA-seq and proteome data (Fig. 3). For example, PLA2 and Disintegrins, which are present in the proteome but they are not part of the transcriptomic results, which may be highlighting venom differences in our samples."<br><br><br>Reviewer #3: Thank you for revising the manuscript. I have two minor comments remaining.<br>Thanks again for the suggestions. We provided below an answer to the following questions:<br><br>- Line 280: "Unmatched MS/MS spectra were de novo sequenced": this doesn't seem correct. They were not sequenced. They were matched/aligned to other snake toxin genes. Please correct the terminology here.<br><br>The MS/MS spectra of peptide ions unmatched in the genomic/transcriptomic reference databases were manually (de novo) sequenced and assigned by BLAST analysis to a known homologue venom toxin.<br><br>- Line 289-290: Thank you for providing coordinates. Please indicate the species for which these coordinates are for.<br><br>We have now indicated the species, thanks again.
**Additional Information:**	
**Question**	**Response**
Are you submitting this manuscript to a special series or article collection?	No
**Experimental design and statistics**<br><br>Full details of the experimental design and statistical methods used should be given in the Methods section, as detailed in our Minimum Standards Reporting Checklist. Information essential to interpreting the data presented should be made available in the figure legends.<br><br>Have you included all the information requested in your manuscript?	Yes
**Resources**	Yes

Powered by Editorial Manager® and ProduXion Manager® from Aries Systems Corporation

A description of all resources used, including antibodies, cell lines, animals and software tools, with enough information to allow them to be uniquely identified, should be included in the Methods section. Authors are strongly encouraged to cite Research Resource Identifiers (RRIDs) for antibodies, model organisms and tools, where possible.<br><br>Have you included the information requested as detailed in our Minimum Standards Reporting Checklist?	
**Availability of data and materials**<br><br>All datasets and code on which the conclusions of the paper rely must be either included in your submission or deposited in publicly available repositories (where available and ethically appropriate), referencing such data using a unique identifier in the references and in the "Availability of Data and Materials" section of your manuscript.<br><br>Have you have met the above requirement as detailed in our Minimum Standards Reporting Checklist?	Yes

Powered by Editorial Manager® and ProduXion Manager® from Aries Systems Corporation

Manuscript Click here to access/download;Manuscript;manuscript_Feb 28.docx

Chromosome-level reference genome for the medically important Arabian horned viper (*Cerastes gasperettii*)

Gabriel Mochales-Riaño[1], Samuel R. Hirst[2], Adrián Talavera[1], Bernat Burriel-Carranza[1,3], Viviana Pagone[1], Maria Estarellas[1], Theo Busschau[4], Stéphane Boissinot[4], Michael P. Hogan[5,6], Jordi Tena-Garcés[7], Davinia Pla[7], Juan J. Calvete[7], Johannes Els[8], Mark J. Margres[2], Salvador Carranza[1]

[1] IBE, Institute of Evolutionary Biology (CSIC-Universitat Pompeu Fabra)

[2] Department of Integrative Biology, University of South Florida, Tampa, FL 33620, USA

[3] Museu de Ciències Naturals de Barcelona, P° Picasso s/n, Parc Ciutadella, 08003 Barcelona, Spain

[4] New York University Abu Dhabi, Abu Dhabi, United Arab Emirates,

[5] Department of Biological Sciences, Florida State University, Tallahassee, FL 33306 USA

[6] University of Michigan, Department of Ecology and Evolutionary Biology, Ann Arbor, MI (48109-1085) USA

[7] Evolutionary and Translational Venomics Laboratory, Consejo Superior de Investigaciones Científicas (CSIC) 46010 Valencia, Spain

[8] Breeding Centre for Endangered Arabian Wildlife, Environment and Protected Areas Authority, Sharjah, United Arab Emirates

Corresponding: gabriel.mochales@csic.es

ORCID iDs: Gabriel Mochales Riaño [0000-0002-9130-2308]; Samuel R Hirst [0000-0002-3172-5210]; Adrián Talavera[0000-0002-4007-0965]; Bernat Burriel-Carranza [0000-0001-9832-1782]; Viviana Pagone [0000-0002-8725-629X]; Maria Estarellas [0000-0001-7360-6458]; Theo Busschau [0000-0001-6012-4943]; Stéphane Boissinot [0000-0002-8760-1284]; Michael P Hogan [0000-0003-3702-5079]; Jordi Tena-Garcés [0009-0003-7302-8303]; Davinia Pla [0000-0002-9810-7556]; Juan J Calvete [0000-0001-5026-3122]; Johannes Els [0000-0002-5614-1887]; Mark J Margres [0000-0002-6153-6701]; Salvador Carranza [0000-0002-5378-3008]

Abstract

Venoms have traditionally been studied from a proteomic and/or transcriptomic perspective, often overlooking the true genetic complexity underlying venom production. The recent surge in genome-based venom research (sometimes called “venomics”) has proven to be instrumental in deepening our understanding of venom evolution at the molecular level, particularly through the identification and mapping of toxin-coding loci across the broader chromosomal architecture. Although venomous snakes are a model system in venom research, the number of high-quality reference genomes in the group remains limited. In this study, we present a chromosome-resolution reference genome for the Arabian horned viper *Cerastes gasperettii* (NCBI:txid110202), a venomous snake native to the Arabian Peninsula. Our highly-contiguous genome (genome size: 1.63 Gbp, contig N50: 45.6 Mbp and BUSCO: 92.8%) allowed us to explore macrochromosomal rearrangements within the Viperidae family, as well as across squamates. We identified the main highly-expressed toxin genes within the venom glands comprising the venom’s core, in line with our proteomic results. We also compared microsyntenic changes in the main toxin gene clusters with those of other venomous snake species, highlighting the pivotal role of gene duplication and loss in the emergence and diversification of Snake Venom Metalloproteinases (SVMPs) and Snake Venom Serine Proteases (SVSPs) for *Cerastes gasperettii*. Using Illumina short-read sequencing data, we reconstructed the demographic history and genome-wide heterozigosity of the species, revealing how historical aridity likely drove population expansions. Finally, this study highlights the importance of using long-read sequencing as well as chromosome-level reference genomes to disentangle the origin and diversification of toxin gene families in venomous snake species.

Keywords: Toxin evolution; gene synteny; genomics; transcriptomics; venom

Background

The rise of genomics in non-model organisms has led to an increase in the number of high-quality reference genomes available in recent years [1-6]. Advances in sequencing technologies have catalyzed the study of several complex traits from a genomic perspective, such as coloration, domestication, or venom, among others [3,7-10]. Among these, venom genomic research has been

particularly important in enhancing our understanding of the origin, evolution and dynamics of this medically relevant trait [11-14]. Venom is a potentially lethal cocktail rich in proteins and peptides (from now on referred to as “toxins”) which are actively secreted by specialized venom glands [11,15]. Toxins can have different effects depending on their type, interactions with other molecules, and the organism in which they are introduced, with convergent outcomes in different taxa [15,16]. Historically, venom research has primarily been conducted using proteomic and transcriptomic approaches (see [7] and references therein). The identification of venom toxins and the characterization of their evolution using reference genomes is a recent and novel field [17]. Previous works have shown that changes in gene regulation can result in the activation and deactivation of venom-coding genes at all taxonomic levels and within the same individual [2,16,18,19]. This suggests that transcriptomic and proteomic data are critical for studying venom in conjunction with well annotated reference genomes to disentangle the complete number and biochemical nature of the toxins an individual can potentially transcribe [7]. Ultimately, the study of venom genomics may yield evolutionary insights into antivenom or drug discovery, as it enables the identification of unexpressed toxin-coding genes. These genes, often overlooked by transcriptomic or proteomic approaches unless ontogeny analyses or in-depth venom expression studies are performed, may target unique physiological pathways. Such discoveries could lead to novel therapies for human illnesses including but not limited to cancer [11,20-22]. Unexpressed toxin-coding genes are particularly noteworthy as they may represent evolutionary 'reservoirs' of bioactive molecules. These genes could encode toxins with unique mechanisms of action, offering untapped potential for drug discovery or therapeutic innovation.

Venom has evolved independently in multiple groups including cnidarians, molluscs, arthropods, squamates and even mammals [11,15]. Venomous snakes are one of the most life-threatening animal groups to humans [23] and, therefore, a medically relevant model system in venom research. Venomous snakes are a diverse group with more than 600 species [24], where venom has evolved with the objective of immobilizing and digesting their prey [25]. From those, more than 370 species have been classified as of medically important by the World Health Organization (WHO) due to their potential severe effects on humans [26]. Snakebite is considered a neglected tropical disease, with annual mortality exceeding 100,000 victims worldwide [23,27]. The most medically important

venomous snake families are Elapidae, Viperidae and Atractaspididae [28], although within Colubridae (*sensu lato*) there are certain medically important venomous species as well [29]. Envenomation by certain members of these families can result in a range of pathologies, spanning neurotoxic, hemotoxic, and/or cytotoxic effects depending on the number and composition of toxins. Neurotoxic venoms primarily target the central nervous system and are mainly composed of small proteins including three-finger toxins (3FTs), snake venom phospholipases A_2 group I (SV-G^I-PLA_2) or dendrotoxins, and are usually associated with elapid snakes [30]. Conversely, hemotoxic and cytotoxic venoms generally are comprised of large enzymatic proteins and protein complexes, including snake venom metalloproteases (SVMP), serine proteases (SP) or snake venom phospholipases A_2 group II (SV-G^{II}-PLA_2), and are typically associated with viperid snakes [28,31,32]. While these historical classifications have proven to be somewhat useful for treating envenomations medically, recent studies have revealed that the presence of these toxins are not exclusive to specific snake families [33].

Vipers (family Viperidae) are a monophyletic lineage of venomous snakes found across Eurasia, Africa and America [34], receiving extensive research attention primarily due to their medical relevance [35-39]. The majority of venom studies in this group have primarily been investigated using a proteomic approach, with early venom work being highly motivated by the medical field, with a limited number of studies employing genomic approaches (but see [2,3,5,40-42]. Sequencing efforts to obtain high-quality reference genomes have mainly focused on pitvipers (Crotalinae subfamily, 11 reference genomes, NCBI last accessed 13 March 2024), especially within the *Crotalus* (n=6) genus, and have focused on the study of venom evolution [2,3,5,43,44]. Other viperids have also been sequenced (although in lower numbers) from both Azemiopinae and Viperinae subfamilies (one and four, respectively) [45-47]. Currently, reference genomes are only available for 16 viper species out of the total 387 total species via the NCBI genomic database [24]. Vipers display extensive variation in venom composition between and within genera [48,49] and even intraspecifically [50,51]. Such differences are most likely due to the high diversity of venom genes and their different effects on prey but also, at least in some cases, the result of introgression with related species [19,50,51]. This provides an extraordinary opportunity to study trait evolution both at inter- and intraspecific levels.

Native to the Arabian Peninsula, the Arabian horned viper (*Cerastes gasperettii*, family Viperidae) is a venomous snake currently recognized within the highest medical importance category (WHO; accessed July, 2024). Extending from the Sinai Peninsula to southwestern Iran in the north and reaching as far as Yemen and Oman in the south, its distribution is widespread (Fig. S1). Found mainly in sandy habitats, this arid-adapted ground-dwelling snake with generalist requirements [52-54] is one of the most common venomous snakes found in Arabia and is responsible for occasional snakebite envenomations [55-57].

In this study, we present a high-quality chromosome-level reference genome assembly for the Arabian horned viper (*Cerastes gasperettii*, NCBI: txid110202), being one of the first within the Viperinae subfamily. Our highly-contiguous genome showcases a high level of similarity at the chromosome level within the Viperidae family with some minor rearrangements with elapids. Moreover, combining genomics, transcriptomics, and proteomics, we characterized the main toxins found in its venom and the location of those toxins in the genome, comparing their evolutionary history and gene copy number variation with other venomous species. We deciphered its adequate levels of genetic diversity. Finally, we reconstructed the demographic history for the species, revealing how historical increases in aridity likely drove population expansions. Overall, the genomic resources generated in this study provide an essential reference resource for forthcoming studies on venom evolution.

Methods

<u>Sampling</u>

Three adult specimens (two females and one male) of *Cerastes gasperettii gasperettii* were used for this study (Table S1). Blood was extracted only from a single female individual (the heterogametic sex, sample CG1) to obtain High-molecular-weight (HMW) genomic DNA (gDNA). We anesthesized the individual, extracted blood from the heart and stored in ethanol and EDTA. For each of the three individuals, we extracted twelve different tissues, including the venom gland, which was stored in RNAlater^{TM} until RNA extraction (Table S1 and Fig. S2). Before dissections, venom was extracted and snakes were allowed to recover for four days to maximize the venom gland transcription. We only

extracted the left venom gland per individual, as previous research within the same family has shown that both venom glands provide indistinguishable results [58].

DNA extraction, library preparation and sequencing

We extracted gDNA from the blood of a female individual (CG1 in Table S1) using the MagAttract HMW Kit (Qiagen) following manufacturer's protocols without modifications. Then, we sequenced a total of two 8M SMRT HiFi cells in a Sequel II PacBio machine, aiming for a ∼30x of coverage, at the University of Leiden. Hi-C libraries were prepared using the Omni-C kit (Dovetail Genomics), following the manufacturer's protocol and using blood stored in EDTA, at the National Center for Genomic Analyses (CNAG), in Barcelona, Spain. The library was paired-end sequenced on a NovaSeq 6000 (2 × 150 bp) following the manufacturer's protocol for dual indexing and aiming for a coverage of ~60x. Finally, we sequenced short-read whole-genome data of the same individual using a NEB Ultra II FS DNA kit; the library was paired-end sequenced on a NovaSeq 6000 (2 × 150 bp) at the Core sequencing platform from the New York University of Abu Dhabi, aiming for ∼70x depth of coverage.

RNA extraction, library preparation and sequencing

We extracted RNA from the same three individuals described above (Table S1 and Fig. S2). RNA was isolated using the HighPurity™ Total RNA Extraction Kit (Canvax, Valladolid, Spain). We selected a total of 35 samples (Table S2). RNA libraries were prepared with the VAHTS Universal V8 RNA-seq Library Prep Kit, being strand-specific and were sequenced on a NovaSeq 6000 (2 × 150 bp) aiming for an average of 40M read pairs per sample (Table S2), but we first sequenced the reference individual and later on the other two samples. Moreover, we sequenced one 8M SMRT HiFi cell on a Sequel II PacBio machine containing two Iso-seq HiFi libraries at University of Leiden: one containing only the venom gland, and the second library being a pool of eight high-quality tissues (brain, kidney, liver, gallbladder, spleen, tongue, pancreas and ovary).

Genome assembly and scaffolding

Quality control of HiFi and Illumina reads was performed using FastQC (FastQC, RRID: SCR_014583) v0.12.1 [59] and adapters were removed with cutadapt (cutadapt, RRID: SCR_011814) v4.9 [60]. In

order to initially explore the genome size, heterozygosity levels and coverage data, we generated a k-mer profile with Meryl (Meryl, RRID: SCR_026366) v1.4.1 [61], using the raw HiFi reads and default parameters, and visualized it with GenomeScope2 (GenomeScope2, RRID: SCR_017014) v2.0.1 [62]. Then, we assembled the genome following the VGP assembly pipeline v2.0 [63]. PacBio HiFi reads were assembled into contigs using the software Hifiasm (Hifiasm, RRID: SCR_021069) v0.21.0 [64], producing primary and alternate assemblies. We used *purge_dups* (purge_dups, RRID: SCR_021173) [65] to remove haplotypic duplicates from the primary assembly and added them to the alternate assembly. Then, we scaffolded the resulting haplotypic assembly using the Hi-C data with SALSA2 (Salsa, RRID: SCR_022013) v1 [66], with default parameters. Following the VGP assembly pipeline [63], manual curation was performed with Pretext (Pretext, RRID: SCR_022024) v0.2.5. Breaks were not mannualy created and we joint contings on gaps previously identified by SALSA2 (Salsa, RRID: SCR_022013). We used the ~78x Illumina data to polish the assembly with one round of Pilon (Pilon, RRID: SCR_014731) v1.24 [67]. The mitochondrial genome was obtained with GetOrganelle (GetOrganelle, RRID: SCR_022963) v1.7.7.1 [68], using the available mitochondrial genome of several *Echis* species (*E. coloratus, E. carinatus* and *E. omanensis*) to seed the assembly (NCBI accession numbers: SRX18902082, SRX18902083, SRX18902084, respectively).

Genome assembly quality evaluation

Quality assessment and general metrics for the final assembly were estimated with both QUAST (Quast, RRID:SCR_011228) v.5.1.0 [69] and gfastats (gfastats, RRID: SCR_026368) v1.3.8 [70]. Possible contaminations were evaluated with BlobToolKit (Blobtools, RRID: SCR_017618) v.4.4.0 [71] using the NCBI taxdump database. We also used MitoFinder v.1.4.2 [72,73] to confirm that the mitochondrial genome was absent in the assembled nuclear reference genome. Completeness of the genome assembly was assessed with BUSCO (Busco, RRID: SCR_015008) v5.3.0. against the sauropsida_odb10 database (*n*=7,480).

Genome annotation

First, we identified repetitive elements using RepeatModeler (RepeatModeler, RRID: SCR_015027) v.2.0.3 [74] for *de novo* predictions of repeat families. To annotate genome-wide complex repeats, we used RepeatMasker (RepeatMasker, RRID: SCR_012954) v.4.1.3 [75] with default settings to identify known Tetrapoda repeats present in the curated Repbase database [76]. Then, we ran three iterative rounds of RepeatMasker (RepeatMasker, RRID: SCR_012954) to annotate the known and unknown elements identified by RepeatModeler in order to maximize the known elements at the expense of diminishing the unknown elements. Later, we soft-masked the genome for simple repeats. We used GeMoMa (GeMoMa, RRID: SCR_017646) v.1.9 [77] to annotate protein-coding genes, combining both the RNA-seq data generated in this study as described above (already mapped in to our new assembly) as well as annotations from six other squamate genomes already published: *Crotalus adamanteus* [2], *Crotalus tigris* [3], *Ophiophagus hannah* [17], *Naja naja* [6], *Crotalus ruber* [42] and *Crotalus viridis* [5]. We quality checked and removed the adapters of the RNA-seq data using fastp v0.23.3 [78], as well as mapped the transcriptomic data to our new reference genome with Hisat2 (Hisat2, RRID: SCR_015530) v2.2.1 [79]. Additionally, we also removed the adapters for the Iso-seq data with fastp (fastp, RRID: 016962) v0.23.3 [78] and mapped the long-read transcriptomic data to our new reference genome with pbmm2 (pbmm2, RRID: SCR_025549), collapsing mapped reads into unique isoforms with isoseq3 and annotated with GeneMarkS-T (GeneMark, RRID: SCR_011930) v5.1 [80]. We combined both annotations (GeMoMa and GeneMarkS-T) with TSEBRA [81]. We BLASTp (blastp, RRID: SCR_001010) our predicted proteins to a Uniprot protein database for a total of ten species (*C. gasperettii*, *C. vipera*, *C. cerastes*, *Anolis carolinensis*, *Crotalus viridis*, *Crotalus tigris*, *Crotalus ruber*, *Crotalus adamanteus*, *Ophiophagus hannah* and *Naja naja*). Simultaneously, we ran Interproscan v5.72 [82] on our predicted proteins. Then, we combined both functional annotations with AGAT v1.4.1 [83]. Finally, as toxin-coding gene families are known to occur in large tandem arrays and the number of paralogs can be underestimated in particular gene families [5], we performed additional annotation steps for toxin genes: Following [3], we used a combination of empirical annotation in FGENESH+ (FGENESH, RRID: SCR_011928) [84], as well as manual annotation using RNA-seq and Iso-seq alignments; the former identified all genes regardless of expression, whereas the latter was used to explicitly identify expressed toxins.

Chromosome-level analyses

Chromosomal synteny was explored between our new chromosome-level reference genome for the Arabian horned viper together with the Eastern diamondback rattlesnake (*Crotalus adamanteus*) [2], the Indian cobra (*Naja naja*) [6] and the Brown anole (*Anolis sagrei*) [85] using Mcscan v1.4.23 [86]. Protein sequences from each of the three venomous snakes were extracted using AGAT v1.2.1 [83] and were pairwise aligned with LAST [87], implemented in the JCVI python module [88]. A first alignment was used between the three species to identify chromosomes assembled in the reverse complement, which were corrected using SAMtools faidx (samtools, RRID: SCR_002105) v1.18.1 [89] using both options reverse-complement and mark-strand. Gene annotations for the new reference (with the corresponding reversed chromosomes) were annotated using GeMoMa (GeMoMa, RRID: SCR_017646) v.1.9 [77], and MCscan (MCScan, RRID: SCR_017650) was rerun. The last four scaffolds (14, 15, 16 and 17) from *Anolis sagrei* were removed, as no orthologous groups were found.

Transcriptomics

After adapter trimming and quality control using fastp (fastp, RRID: SCR_016962) v0.23.3 [78], we mapped our RNA-seq reads to the reference genome of *Cerastes gasperettii* using Hisat2 (Hisat2, RRID: SCR_015530) v2.2.1 [79]. Gene expression raw counts per gene across all samples were calculated with StringTie (Stringtie, RRID: SCR_016323) [90]. Initial exploration of our transcriptomic data revealed a clear batch effect for one of the three samples (Fig. S4), due to the low mapping of that sample to our reference genome. Therefore, we decided to remove individual CG1 from future RNA-seq analyses. Moreover, to avoid pseudoreplication, we also removed the accessory gland from individual CG009 due to its high similarity with the venom gland, suggesting that the venom gland rather than the accessory gland was sampled (Fig. S4). Differential expression analyses were carried out with the DESeq2 package (DESeq2, RRID: SCR_015687) v.1.42.0 [91] from R (R, RRID: SCR_001905) v4.4.2 [92]..Prior to analysis, genes with fewer than 10 counts across all samples were filtered out. For comparisons, we defined two groups: venom glands versus all other tissues. DESeq2 (DESeq2, RRID: SCR_015687) employs a negative binomial generalized linear model to estimate differences in gene expression, and the p-values were adjusted for multiple testing using the Benjamini-

Hochberg method to control the false discovery rate (FDR). Genes with an adjusted p-value < 0.01 and a fold change > 2 were considered significantly differentially expressed. Finally, we identified the highly expressed genes found in the venom gland as well as the toxins uniquely expressed in the venom gland (following [6]) which were defined as (1) genes expressed in the venom gland (TPM > 500), (2) Differential Upregulated Genes (DUGs) with Fold Change (FC) > 2 comparing venom glands to all other tissues and (3) unique to venom glands (TPM < 500 in all other tissues).

Proteomics

A bottom-up mass spectrometry strategy [93] was used to characterize the venom of *Cerastes gasperettii*. Briefly, the venom proteome (pool from individuals CN6134 and CN6135, both from United Arab Emirates (UAE); Table S1) was submitted to reverse-phase High-performance liquid chromatography (HPLC) decomplexation followed by SDS-PAGE analysis in 12% polyacrylamide gels run under non-reducing and reducing conditions. Protein bands were excised from Coomassie Brilliant Blue-stained gels and subjected to automated in-gel reduction and alkylation on a Genomics Solution ProGest™ Protein Digestion Workstation. Tryptic digests were submitted to MS/MS analysis on a nano-Acquity UltraPerformance LC® (UPLC®) equipped with a BEH130 C_{18} (100µm x 100mm, 1.7 µm particle size) column in-line with a Waters SYNAPT G2 High Definition mass spectrometer. Doubly and triply charged ions were selected for CID-MS/MS. Fragmentation spectra were matched against a customized database including the bony vertebrates taxonomy dataset of the NCBI non-redundant database (release 258 of October 15, 2023) plus the species-specific venom gland transcriptomic and genomic protein sequences gathered in this work. Search parameters were as follows: enzyme trypsin (two-missed cleavage allowed); MS/MS mass tolerance for monoisotopic ions: ± 0.6 Da; carbamidomethyl cysteine and oxidation of methionine were selected as fixed and variable modifications, respectively. Assignments with significance protein score threshold of $p < 0.05$ (Mascot Score > 43) were taken into consideration, and all associated peptide ion hits were manually validated. Unmatched MS/MS spectra were *de novo* sequenced and manually matched to homologous snake toxins available in the NCBI non-redundant protein sequences database using the default parameters of the BLASTP program (RRID:SCR_001010).

Local synteny analyses

To explore toxin genomic organization across (sub)families, we used BLASTn, incorporating both toxin and non-toxin paralogs to identify the genomic location of SVMPs, SVSPs and PLA_2 toxin families, across the genome of *Cerastes gasperettii*, *Crotalus adamanteus, N. naja* and *A. feae*. We excluded *A. feae* for SVSPs and SVMPs local synteny analyses as those families were not assembled onto a single contig in the *A. feae* genome. Then, we aligned those regions using Mafft [94]: For SVMPs in CHR8:16.506.135 to CHR8:17.374.029, for SVSPs in CHR9: 17.531.416 to CHR9:17.788.049 and for PLA_2 in CHR17:7.882.542 to CHR17:7.916.827 Each species was annotated within the MSA using its own annotation as a reference in Geneious Prime 2023.0.4. Results were plotted using the gggenomes package [95] from R v4.4.2 [92].

Toxin phylogenies

We used phylogenetic inference to study the evolutionary history for the main groups of toxins (i.e., SVMPs and SVSPs), which were the most abundant in the proteome of *Cerastes gasperettii*, as well as PLA_2 as this family has been widely studied within the Viperidae family [12,96]. For the three main toxin families, we selected available toxin genes as well as non-toxin paralogous genes from venomous species; we also included other non-toxin paralogous genes from non-toxic species (for details about this see Supplementary datasets for the three main toxins). When nuclear sequences were obtained, we translated CDS to protein sequence, and then protein sequences were aligned with Mafft (Mafft, RRID: SCR_011811) v7 [94]. Following [13], we built a phylogeny for each of the toxin groups with the translated CDS sequences, as explained above, using Phyml (Phyml, RRID: SCR_014629) v3.3 [97], implementing the Dayhoff substitution model and validating our inferred tree with aBayes support.

Demographic history

We inferred the demographic history of *Cerastes gasperettii* by implementing the Pairwise Sequential Markovian Coalescent (PSMC v0.6.5) software [98] on the short-read whole-genome data. Heterozygous positions were obtained from bam files with Samtools v1.9 mpileup function [99], and data were filtered for low mapping (<30) and base quality (<30). Minimum and maximum depths were

set at a third (27x) and twice (156x) the average coverage. Only autosomal chromosomes were considered. We used the squamate mutation rate of $2.4x10^{-9}$ substitutions/site/generation and a generation time of 3 years, following [100,101], respectively. A total of ten bootstraps were calculated, plotting the final results with the psmc_plot.pl function from PSMC (RRID:SCR_017229).

Genomic diversity

We downloaded Illumina data for *Bothrops jararaca* (SRR13839751 from [40], *Crotalus viridis* (SRR19221440; [5]), *Naja kaouthia* (SRR8224383; [102]), *Naja naja* (SRR10428156; [6]) and *Sistrurus tergeminus* (SRR12802282; [103]). Then, we filtered for quality (Phred score of 30) and removed adapters with fastp (fastp, RRID: SCR_016962) v0.23.3 [78]. Trimming of poly-G/X tails and correction in overlapped regions were specified. All other parameters were set as default. Filtered sequences were visually explored with FastQC (fastQC, RRID: SCR_014583) v0.12.1 [59] to ensure data quality and absence of adapters. *Cerastes gasperettii* filtered reads were mapped against the new reference genome of *Cerastes gasperettii* using the bwa mem algorithm (bwa, RRID: SCR_010910) v0.7.17 [104]. *B. jararaca*, *C. viridis* and *S. tergeminus* were mapped against the *C. viridis* [5] reference genome and *N. naja* and *N. kaouthia* were mapped against the *N. naja* reference genome [6]. Mapped reads were sorted with Samtools (Samtools, RRID: SCR_002105) v1.9 [99] and duplicated reads were marked and removed with PicardTools (Picard, RRID: SCR_006525) v2.28.0 [105]. Reads with mapping quality lower than 30 were discarded. SNP calling was carried out with HaplotypeCaller from GATK (GATK, RRID: SCR_001876) v.4.1.3.0 [106], with BP_resolution and split by chromosome. For each chromosome, individual genotypes were joined using CombineGVCFs with convert-to-base-pair-resolution, and the GenotypeGVCFs tool was then applied to include non-variant sites. Finally, for each individual, the whole dataset split by chromosome was concatenated with bcftools concat (bcftools, RRID: SCR_ 005227) [89], keeping only the autosomes. Then, for each sample, we used the raw dataset to calculate average genome heterozygosity. We generated non-overlapping sliding windows for each of the reference genomes and included only sites (both variant and invariant) with site quality higher than 30 (QUAL field in a VCF file from GATK). Only windows containing more

than 60,000 unfiltered sites were considered. Visualization was carried out with ggplot2 (ggplot2, RRID: SCR_ 014601) [107] in R (R, RRID: SCR_001905) v4.4.2 [92].

Results and Discussion

Genome assembly and annotation

We generated a high-quality chromosome-level assembly for the Arabian horned viper (*Cerastes gasperettii*) by combining PacBio HiFi (65 Gbp of data), Hi-C (96 Gbp of data) and Illumina data (135 Gbp of data) (Fig. 1 and Fig. S3). First, we *de novo* assembled the HiFi reads into 1,018 contigs (N50=45.7 Mbp; longest contig of 149.99 Mbp). Then, using the proximity ligation data (i.e., Hi-C), we scaffolded the genome into 319 scaffolds (N50=111.38 Mbp; largest scaffold 345.38 Mbp). After manual curation, the scaffolding parameters of our genome were improved (N50=214.14 Mbp; largest scaffold 361.99 Mbp), containing 99.44% of the genome present in 19 scaffolds or pseudochromosomes (7 macro-, 10 micro-, Z and W sex chromosomes; Table 1 and Fig. 1B). The total genome length was 1.63 Gb, similar to other venomous snakes [3,5-6,17] (Table 1), with a contig N50 of 45.6 Mbp, ~3.3 times more contiguous than the *N. naja* genome [6], ~228 times more contiguous than the *Anolis sagrei* genome [85], but 0.67 times less contiguous than the recently published *Crotalus adamanteus* genome [2], making it one of the most contiguous chromosomal squamate genomes assembled to date (Table 1). We assessed the completeness of the assembly using BUSCO [108] with the sauropsida gene set (n=7,480). Upon evaluation, we successfully identified 92.8% of the genes (91.4% single-copy, 1.4% duplicated), while the remaining genes were fragmented (1%) or missing (6.2%; Fig. 1). For the *de novo* assembly, GC content and repeat content were 37.87% and 43.63%, respectively. The repetitive landscape was dominated by retroelements (30.25%), with a majority of LINEs (21.25%) (Table S3). Finally, we annotated 27,158 different protein-coding genes within our assembly, with a total of 194 putative toxins or toxin-paralogs genes. Toxin genes were found in both macro- and microchromosomes (Fig. 1), and were found onto individual contigs. Finally, we also found a battery of 3FTxs and myotoxin-like genes, but they were not represented in our proteome and RNA-seq dataset (see below).

Genomic architecture highly conserved among vipers

Whole-genome synteny comparisons showed similarity between *Cerastes gasperettii* and *Crotalus adamanteus*, with large syntenic blocks both within macro- and microchromosomes (Fig. 2). Some chromosomal rearrangements were observed between viperids and elapids, as previously discussed by [6], with a fission of chromosome four in *N. naja* to form chromosomes five and seven in vipers, and a fusion of chromosomes five and six in *N. naja* to form chromosome four in vipers. Interestingly, several chromosomal rearrangements between lizards and snakes have occurred, as we found several fission events in the *A. sagrei* genome, including one fission from chromosome two to originate the current Z chromosome in snakes (Fig. 2).

Toxins uniquely expressed in the venom glands

Our analyses of multi-tissue transcriptomic data (23 samples from two individuals covering 13 different tissues) reported a total of 23,178 expressed genes (TPM > 1). Heatmap of the 2,000 most variable genes reported unique upregulated genes for each of the analyzed tissues (Fig. S5). The venom gland transcriptome contained a total of 7,237 genes expressed (TPM > 500), including a total of 65 putative toxin genes. From those, we did not detect any 3FTxs and/or myotoxin-like gene transcripts. Differential gene expression analyses revealed a total of 161 genes (33 putative toxin genes) that were differentially upregulated (FC > 2 and 1% FDR) in venom glands compared to other tissues (Fig. 3A and Fig. S6-7). Finally, a total of 10 toxin genes (*CRISP2*, *SVMP9, SVMP10, SVSP8, SVSP7, SVSP5, CTL14*, *CTL15*, *SVSP4* and *SVMP13*) were uniquely expressed in the venom gland, encoding for the minimal core venom effector (Fig. 3A) [6], and in line with the main toxins found within the proteome (Fig. 3B), although some differences were observed(as the absence of PLA_2 within the highly-expressed genes), possibly due to individual venom differences. These 10 genes, together with other SVMPs, SVSPs and C-type lectins (CTL), were highly expressed in the venom gland and form the core toxic effector components of the venom. Targeting the core toxins together with other well-known modulators of venom may help manufacture of synthetic antivenom treatments as well as improve neutralization tests of current antivenoms [6]. However, more transcriptomic data should be incorporated to correct for potential ontogenetic and geographical variation in venom composition in *C. gasperettii* [18,109].

SVSPs and SVMPs as main toxins

Venom proteomics identified Snake venom Serine Proteases (SVSPs) and Snake Venom Metalloproteinases (SVMPs) as the most abundant toxin families within the venom of *Cerastes gasperettii*, with 37.38% and 22.19% of the venom being composed by peptides from those two families, respectively (Fig. 3B); the dominance of these two toxin families is consistent with previous research on the same genus [110,111]. Other toxin families identified were DISI (12.74%), CTL (7.25%), PLA_2 (5.47%), Cysteine-Rich Secretory Proteins (CRISP; 4.34%) or L-Amino acid oxidase (LAAO; 1.71%) (Fig. 3B). We did not detect any 3FTx or myotoxin-like peptides within the proteome.

SVMPs

We analyzed the evolution of venom of the most abundant venom toxin groups (i.e., SVMPs and SVSPs, as well as PLA_2). After a thorough manual curation, we used comparative genomics to evaluate the number and position of those genes in comparison with the Indian cobra (*N. naja*), the Eastern diamondback rattlesnake (*Crotalus adamanteus*), and the Fea's viper (*A. feae*). We reported a total of 13 fully contiguous tandem array SVMPs for *Cerastes gasperettii* (Fig. 4A), next to the non-toxic paralogous gene *ADAM28* and flanked by the *NEFL* and *NEFM* non-toxic genes. Microsyntenic analyses showed gene copy number variation between the studied species (Fig. 4A). Overall, we can see an expansion in the number of SVMPs within the Viperidae family, particurlarly in *Crotalus adamanteus* (22 copies unique to vipers and 10 lineage-specific copies) but also in *Cerastes gasperettii* (12 copies unique to vipers and one lineage-specific copy) (Fig. 4A). Then, we reconstructed the evolutionary history of this toxin family (Fig. 4B and 8). Phylogenetic analyses for this toxin group reported a highly supported clade comprising *ADAM28* peptides, the non-toxic paralogous gene. The second clade of orthologous toxin-peptides were found within both elapid and viperid families (including species from Crotalinae and Viperinae subfamilies in viperids; Fig. S8) as well as two SVMPs from *A. feae*. Interestingly, we report a new toxin-coding gene within *Cerastes gasperettii* with a different evolutionary history, as it did not share orthology with any other gene (Fig. 4B). This new gene likely arose from a duplication event of *SVMP13*, within the group of SVMP *MDC1* toxins (Fig. S8). Our discovery of a novel SVMP gene in *C. gasperettii* adds to the growing body of work on the

dynamic evolution of venom systems. Similar gene expansions and duplications have been observed in other species, such as PLA_2 toxin-coding genes found in the venom of *Azemiops feae* [96], highligting the lineage-specific nature of venom evolution. The gene we identified, possibly arising from an *SVMP13* duplication, do not share orthology with genes in other species, suggesting the presence of hidden toxin diversity in venom systems. This discovery highlights the importance of using genomics in studying venom evolution, as this putatively toxic gene was not found to be differentially upregulated in the venom gland or recovered in the proteome (Fig. 3). More genomic data will indicate if *SVMP12* is unique for the Viperinae subfamily, the *Cerastes* genus or if it is only found in *Cerastes gasperettii*. All other clades were unique to viperids (and some exclusive only to crotalids), except for a clade composed by SVMPs unique to elapids, as previously discussed in [6]. Interestingly, one of the toxins (*SVMP8*) was not a class P-III SVMP, as it clusters within the MAD-4/5 clade (class P-II SVMP), contrary to the proteomic results where all SVMPs were categorized within the class P-III (Fig. 3B). Although there has been a clear expansion of the SVMP family within the *Crotalus* genus, our results suggest that the origin of that expansion was at the beginning of the Viperidae family, as most of the groups are also present within the Viperinae subfamily.

<u>PLA_2</u>

Regarding PLA_2, we report two tandem repeat venom genes for *Cerastes gasperettii* within the non-toxic *PLA_2-g2E* and *PLA_2-g2F* array (Fig. 4C), flanked by *OTUD3* and *MUL1* non-toxic genes, as previously reported in other species [3,12,96]. The number of venomous PLA_2 in *Cerastes gasperettii* was lower than in *A. feae* and *Crotalus adamanteus*. Phylogenetic results for PLA_2 genes showed a fully supported clade containing both non-toxic *PLA_2-g2E* and *PLA_2-g2F* as outgroups (Fig. 4D and Fig. S9). We also found all other PLA_2 groups reported in previous studies: *PLA_2-gC, PLA_2-gK, PLA_2-gB, PLA_2-gD* and *PLA_2-gA* [12,96]. The two genes for our target species clustered in different groups (Fig. 4D and Fig. S9). The first PLA_2 was a *PLA_2-gD*, which is a group of PLA_2s exclusively found in true vipers (subfamily Viperinae). The second one was a *PLA_2-gC* which is more ancestral as it is also found in other pitvipers and non-venomous snakes such as pythons [12]. The genomic results are consistent with

the proteomics, indicating that specific duplications of PLA_2 toxin-coding genes have not occurred in *Cerastes gasperettii*.

SVSPs

We found eight different SVSPs within the genome of *Cerastes gasperettii*, flanked by *RBM42* and *GRAMD1A* non-toxic genes (Fig. 4E). For this toxin family, we were only able to compare the results with *Crotalus adamanteus*. We were unable to confidently determine the location of SVSPs in the *N. naja* genome (several regions were matching our venomous SVSP genes as well as the flanking genes). Moreover, *A. feae* was also not compared as SVSPs were not assembled in a single contig.. Phylogenetic results showed three clades, with two containing *Cerastes gasperettii* genes (Fig. 4F and Fig. S10). Group 1 was mainly present within *Crotalus*, although there was the presence of some true vipers species, but not in *Cerastes gasperettii* (Fig. S10). Group 2 contained six genes within *Crotalus adamanteus* and only two for *Cerastes gasperettii*. Interestingly, Group 3 was expanded in *Cerastes gasperettii* (Fig. 4E) with a total of six copies, while four were found within *Crotalus adamanteus*. Most of the toxins included in the analyses for true vipers were also found in Group 3 (Fig. S10), indicating a possible expansion of this group of toxins in true vipers (or gene losses in pit vipers). Overall, our high-quality chromosome level reference genome has shed light on the evolution of the main toxin-coding gene families, indicating a compelling correlation between the abundance of toxin-coding genes and the prevalence of these toxins in the venom of *Cerastes gasperettii*.

Glacial periods drove population expasions in *C. gasperettii*

The Arabian horned viper (*C. gasperettii*) is a widespread species, categorized as Least Concern by the IUCN [111]. Genome-wide diversity was in line with its conservation status, as it showed similar heterozygosity levels compared to other venomous snakes (Fig. 5A). However, more individuals should be sampled along its distribution to verify that similar heterozygosity levels are found across its range. PSMC analyses showed several population expansions and contractions in the last 400 kya, whilst the effective population size of *Cerastes gasperettii* remained relatively constant from 1 until 10 Mya (Fig. 5B). Interestingly, population expansions were coincident with the Last glacial and Penultimate glacial periods (grey lines on Fig. 5B), with a large population increase during the Penultimate Glacial Period

(PGP) (1.94 to 1.35 mya) (Fig. 5B). In fact, during glacial periods, global sea level dropped around 150 meters, exposing the floor and the sand to the wind, which promoted aridification in the Arabian Peninsula and potentially increased habitat suitability for the species [112,113]. PSMC results may vary depending on the generation time as well as the mutational rate specified. The absence of species-specific data for this analyses may bias our results, although it is a general consensus in the literature when inferring these demographyc analyses in snakes (e.g. [5]).

Conclusions

Our high-quality chromosome-level reference genome for *C. gasperettii* showed that chromosomal architecture is highly conserved between Crotalinae and Viperinae subfamilies, and differs from elapid genomes by a small number of chromosomal rearrangements. We also found the genomic coordinates of the main toxin-encoding genes, highlighting gene duplication as the main driver in the evolution of SVMP and SVSP toxins. We identified a new SVMP toxin-coding gene, showcasing the importance of using high-quality reference genomes (combined with other -omic techniques) for thoroughly characterizing toxin-encoding genes. Finally, this is a new and important resource for a large clade with few reference genomes available. Future genomic studies focusing on Old World viper evolution will benefit greatly from this resource, which will help unveil the origin and diversification of venom and serve as an essential genomic tool for further venomic studies on the subfamily Viperinae.

Acknowledgements

GM-R is supported by an FPI grant from the Ministerio de Ciencia, Innovación y Universidades, Spain (PRE2019-088729), SRH is awarded by the National Science Foundation Graduate Research Fellowship Program with grant no. 2136515, AT is supported by "la Caixa" doctoral fellowship program (LCF/BQ/DR20/11790007), BB-C is supported by FPU grant from Ministerio de Ciencia, Innovación y Universidades, Spain (FPU18/04742) and ME is supported by an FPI grant from Ministerio de Ciencia e Innovación (PRE2022-101473). In the UAE, we wish to thank His Highness Sheikh Dr. Sultan bin Mohammed Al Qasimi, Supreme Council Member and Ruler of Sharjah, H. E. Ms. Hana Saif al Suwaidi (Chairperson of the Environment and Protected Areas Authority, Sharjah) for their continuous support. Some of this research was carried out on the High Performance Computing

resources at New York University Abu Dhabi. We thank Jonathan Wood and Klara Eleftheriadi for their input during the genome assembly and manual curation processes. We also thank Valéria Marques for her help in building the figures and Prem Aguilar for reviewing a previous version of the manuscript.

Data availability

Final assembly and raw reads files were deposited in NCBI under bioproject No. PRJNA1068073. Proteomic data was published at PRIDE [114,115] under Project accession numbers PXD060777 and PXD060783. All additional supporting data are available in the *GigaScience* repository, GigaDB [116].

Funding

This work was funded by grant PID2021-128901NB-I00 (MCIN/AEI/10.13039/501100011033 and by ERDF, A way of making Europe; Spain) and grant 2021-SGR-00751 from the Departament de Recerca i Universitats from the Generalitat de Catalunya, Spain to SC.

Competing Interests

The authors declare that they have no competing interests.

Author's contribution

Conceptualization: G.M.R., A.T., B.B.C., J.C., J.E., M.M., S.C. Investigation: S.H, V.P., M.E., T.B., S.B., M.H., J.T.G., D.P., J.C., M.M. Funding acquisition: S.C. Writing-original draft: G.M.R. Writing-review & editing: All authors read, revised, and approved the manuscript final version.

Ethics statement

No in vivo experiments were performed. Specimens were collected and manipulated with the authorization and under strict control and permission of the government of the United Arab

Emirates (Environment and Protected Areas Authority, Government of Sharjah), who approved the study. Specimens were captured and processed following the guidelines and protocols stated in the agreements obtained from the competent authority of the United Arab Emirates. Members of the government supervised collecting activities. All efforts were made to minimize animal suffering. All the research in the United Arab Emirates was done under the supervision and permission of the Environment and Protected Areas Authority, Government of Sharjah.

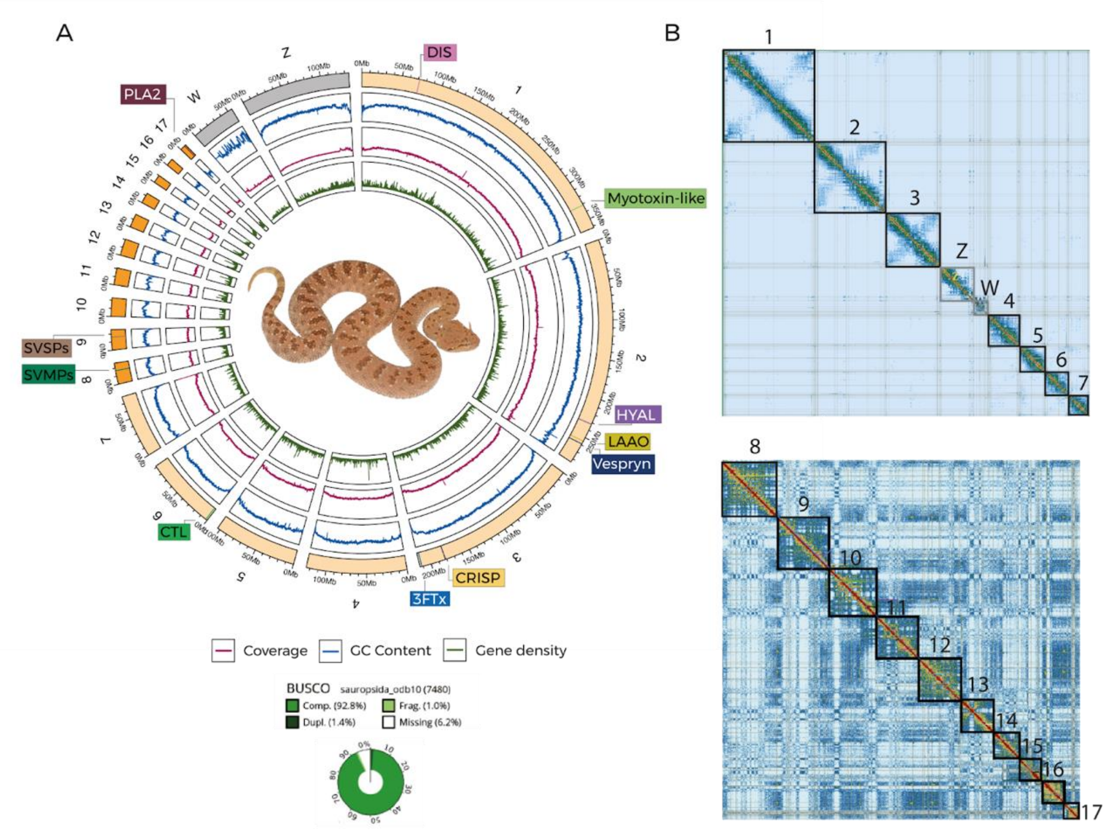

Fig. 1: A) Reference genome for *Cerastes gasperettii*, including BUSCO score, GC content, coverage level as well as the main toxins found within the genome. Macrochromosomes are shown in light orange whilst microchromosomes are shown in bright orange. Sex chromosomes are shown in gray. Abreviations are as follows: DIS, Disintegrins; HYAL, Hyaluronidases; LAAO, L-Amino acid oxidase; CRISP, Cysteine-rich secreted proteins; 3FTx, Three-finger toxins; CTL, C-type lectins; SVMPs, Snake venom metalloproteinases; SVSPs, Snake venom serine proteinases; PLA_2, Phospholipases. B) HiC contact map for the macrochromosomes (above), including the sex chromosomes (Z and W), and microchromosomes (below).

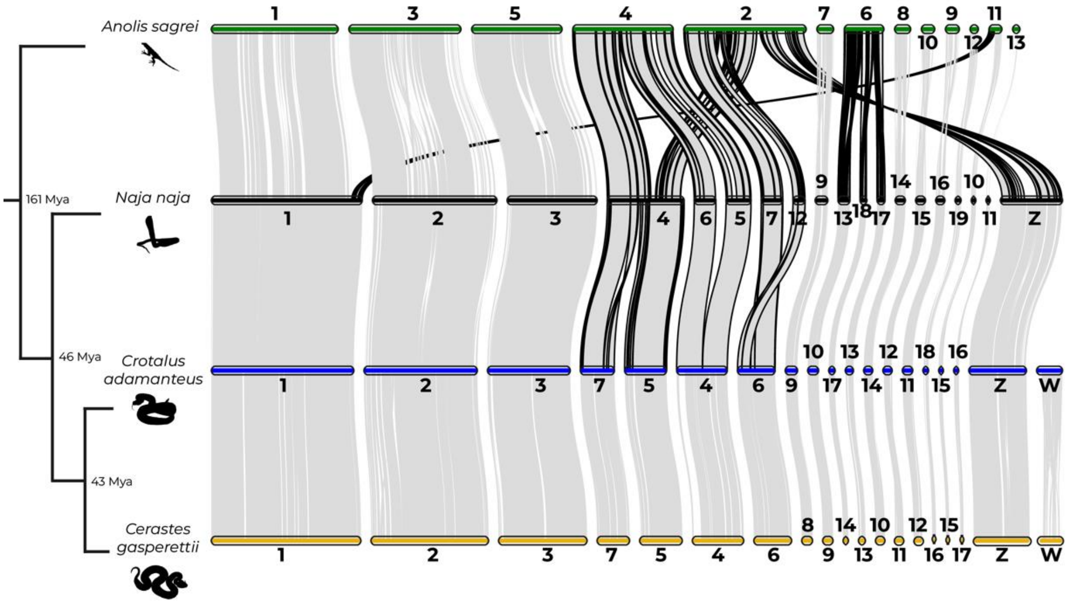

Fig. 2: Chromosome-level analyses for one Elapidae (*Naja naja*), one Crotalinae (*Crotalus adamanteus*) and one Viperinae (*Cerastes gasperettii*) species, with *Anolis sagrei* as the outgroup. The four smallest scaffolds (14, 15, 16 and 17) of *Anolis sagrei* were removed, as no orthologous groups were found with other species. Borders of regions showing evidence for chromosomal rearrangements are shown in black. Estimates for branch times obtained from TimeTree.org based on divergence times between Iguania and Serpentes, Elapidae and Viperidae and Crotalinae and Viperinae, respectively.

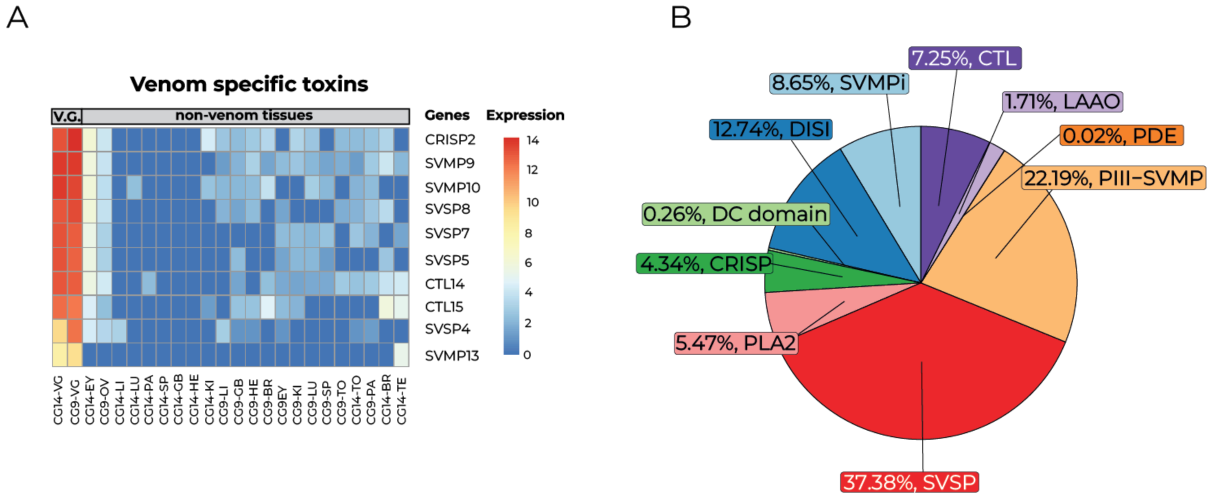

Fig. 3: Main toxins found in both the transcriptome and proteome of *Cerastes gasperettii*. A) Transcriptomic results with genes upregulated and exclusively found in the venom gland for both individuals. Each column represents a different tissue type per sample. Rows show the different genes, and colors correspond to different expression levels. Abbreviations are as follows: VG, Venom Gland; EY, Eye; OV, Ovary; LI, Liver; LU, Lung; PA, Pancreas; SP, Spleen; GB, Gallbladder; HE, Heart; KI, Kidney; LI, Liver; BR, Brain; TO, Tongue; TE, Testis. B) Proteomic results of venom composition for a pool of two individuals of *Cerastes gasperettii*. The pie chart displays the relative abundances of the toxin families found in the proteome of the *Cerastes gasperettii* venom. Abbreviations are as follows: SVMP, snake venom metalloproteinase; SVSP, snake venom serine proteases; PLA_2, phospholipases A_2; CRISP, cysteine-rich secretory proteins; DISI, disintegrins; CTL, C-type lectins; LAAO, L-amino-acid oxidases; PDE, phosphodiesterases.

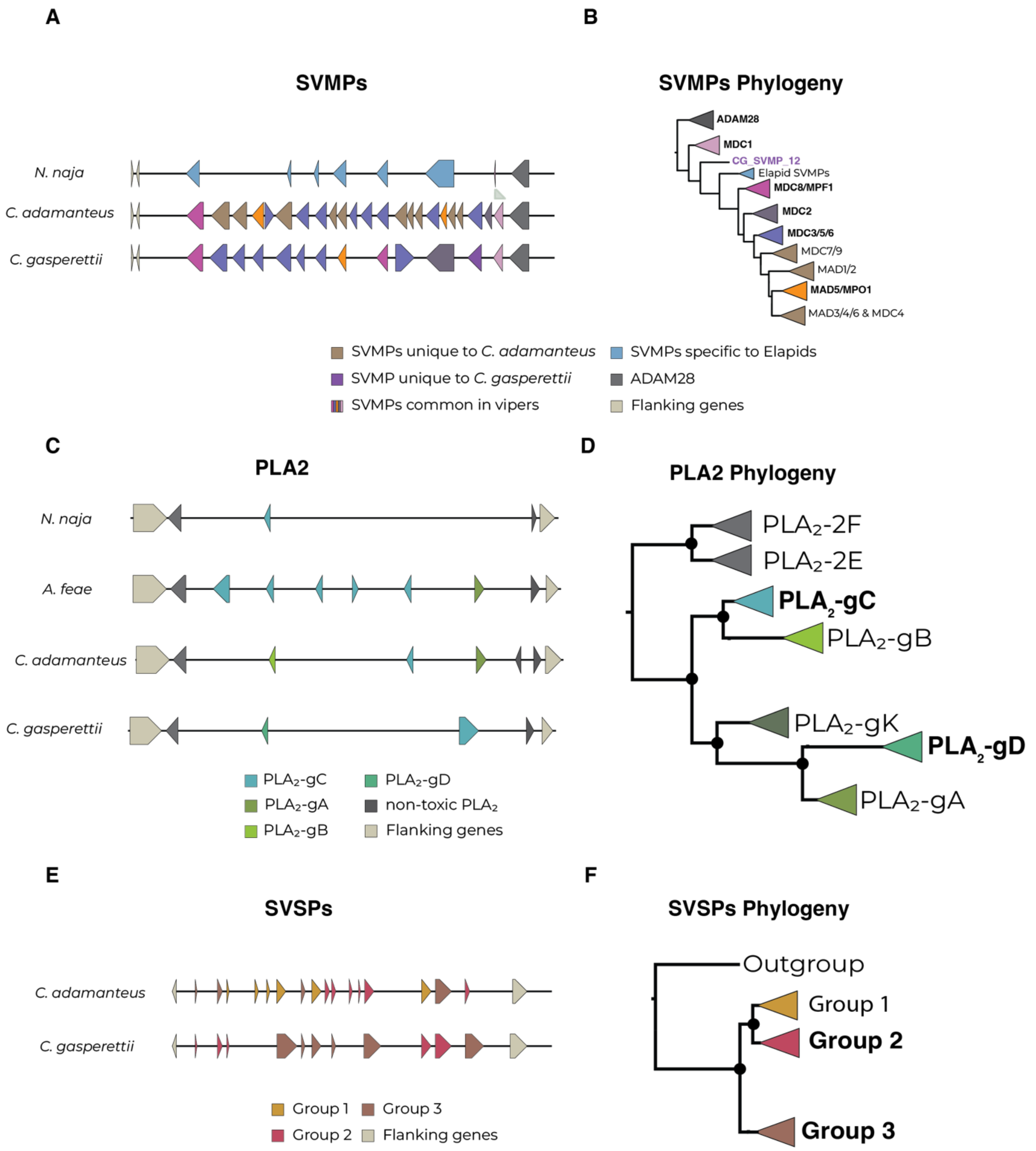

Fig. 4: A) Local synteny analyses for the SVMP toxin family in *Naja naja*, *Crotalus adamanteus* and *Cerastes gasperettii*. Different colors indicate orthologous genes unique to *C. gasperettii*, crotalids, true vipers or elapids. ADAM28 (right) as well as flanking genes (left) are also indicated. B) Phylogeny of SVMPs, in bold, groups that contained SVMPs from *Cerastes gasperettii*. In purple is indicated the gene exclusively found in *Cerastes gasperettii*. C) Local synteny analyses for PLA_2 in *Naja naja*, *Azemiops feae*, *Crotalus adamanteus* and *Cerastes gasperettii*. Non-toxic PLA_2 and flanking genes are also shown. D) Phylogeny of the PLA_2 gene family, with two non-toxic PLA_2 as outgroups. Some samples that did not fit in any category have been removed. For a complete phylogeny see Fig. S9. Note that PLA_2-gK is present in the phylogeny but not in the local synteny analyses, as any of the studied species contains it. E) Local synteny analyses for SVSPs for *Crotalus adamanteus* and *Cerastes gasperettii*. Flanking genes are also shown. F) Phylogeny for SVSPs with a non-toxic outgroup. For the three different phylogenies the groups that contained toxins from *Cerastes gasperettii* are highlighted in bold.

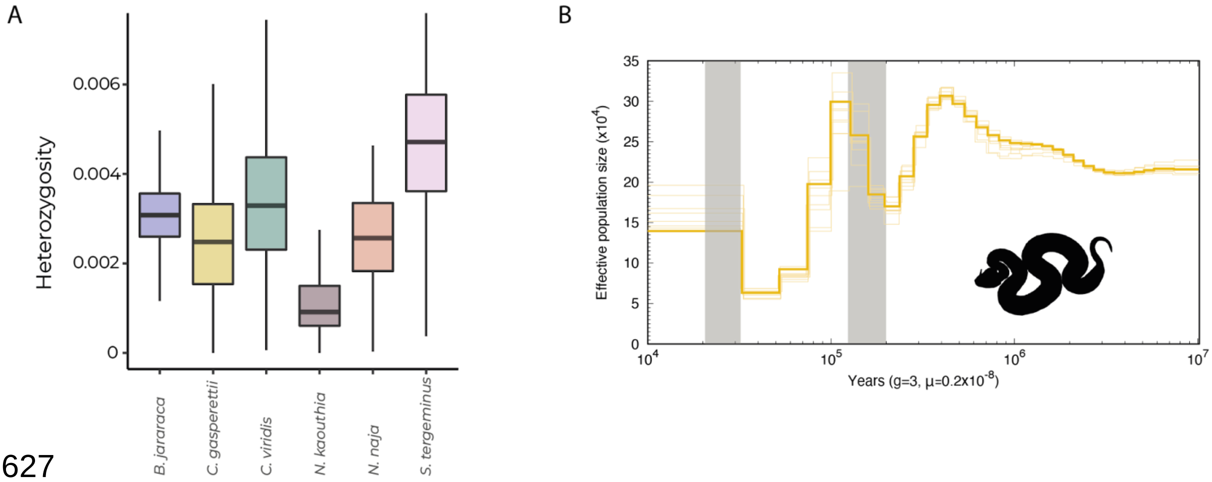

Fig. 5: A) Genome-wide diversity for a total of six different venomous snakes: *Bothrops jararaca*, *Cerastes gasperettii*, *Crotalus viridis*, *Naja kaouthia*, *Naja naja* and *Sistrurus tergeminus*. B) PSMC analysis recovering the ancient demographic history of *Cerastes gasperettii*. Generation time was set to 3 years and the substitution rate to $2.4x10^{-9}$ per site per year. Shaded lines represent 10 bootstrap estimates. Two last glacial periods are shown with grey lines.

Table 1: Comparison of our new reference genome for *Cerastes gasperettii* with other high-quality squamate genomes. Best value per category is shown in bold.

	Cerastes gasperettii	*Crotalus adamanteus*	*Naja naja*	*Anolis sagrei*
Genome size	1.63 Gbp	1.69 Gbp	1.79 Gbp	1.92 Gbp
Number of scaffolds	221	**27**	1,897	3,738
Scaffold N50	214.14 Mbp	208.9 Mbp	223.35 Mbp	**253.58** Mbp
Scaffold L50	**3**	**3**	**3**	4
Contig N50	45.6 Mbp	**67.5** Mbp	13.06 Mbp	0.2 Mbp

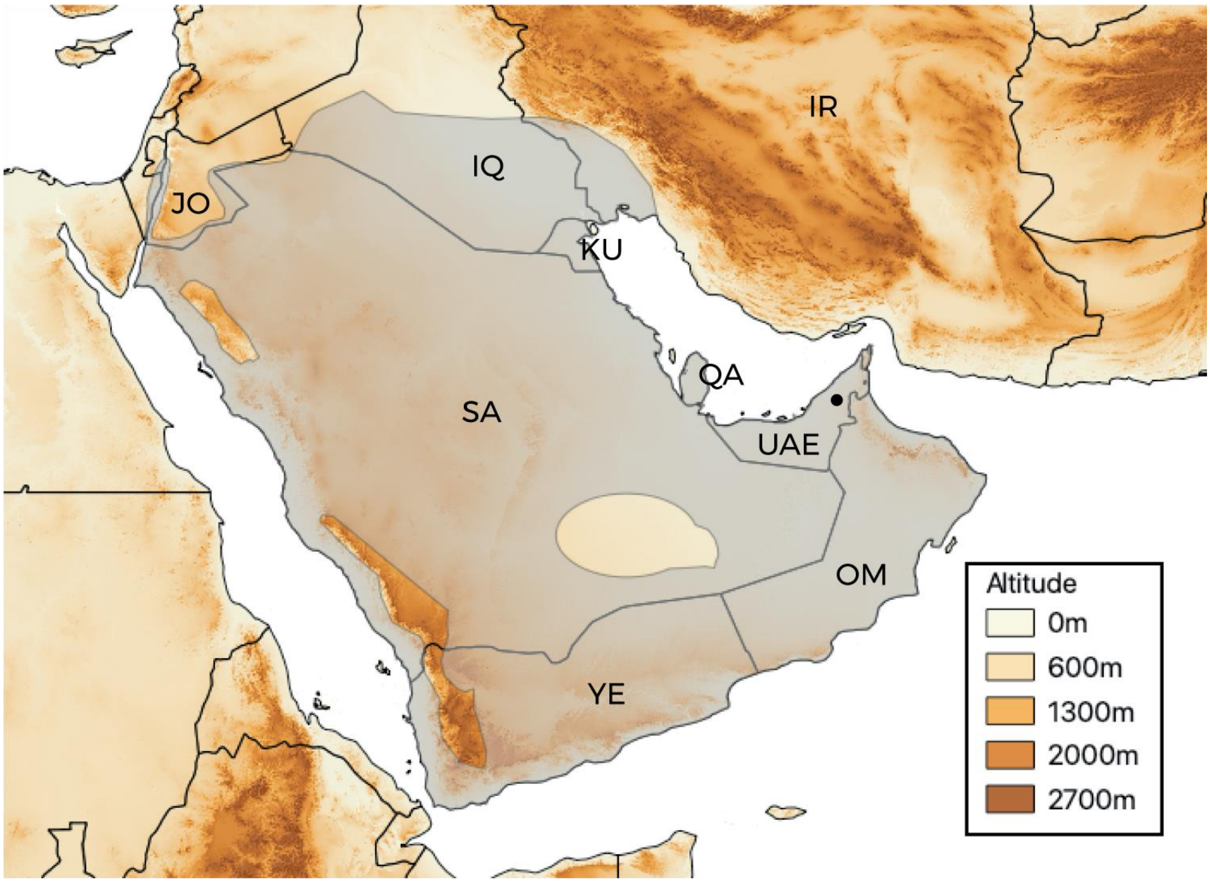

Fig. S1: Distribution map for the studied species *Cerastes gasperettii* with the location of our samples. Countries where the species is present are indicated. Abbreviations are as follows: JO, Jordania; SA, Saudi Arabia; YE, Yemen; OM, Oman; UAE, United Arab Emirates; IQ, Iraq; IR, Iran; KU, Kuwait, QA, Qatar.

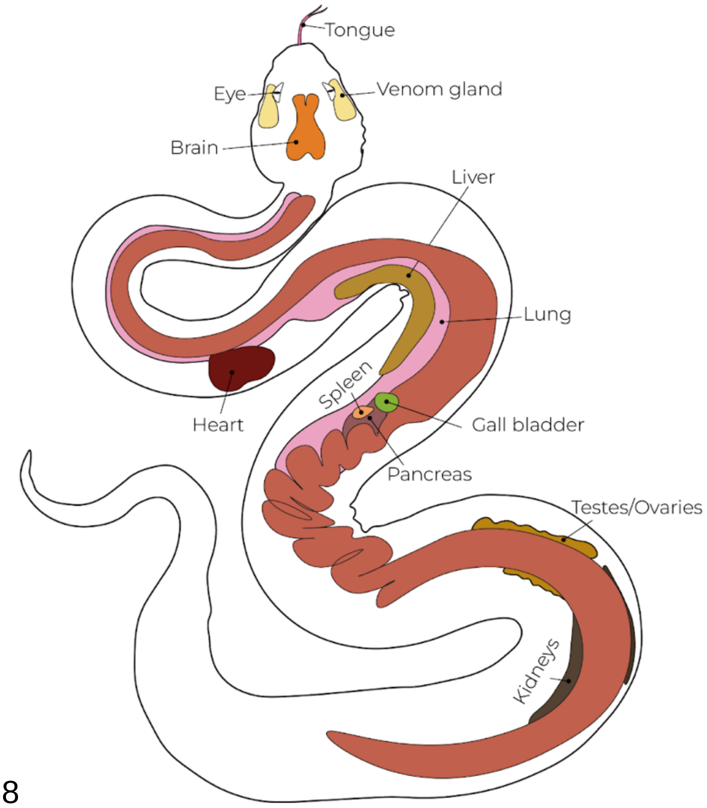

Fig. S2: Drawing of an Arabian horned viper depicting all the tissues sampled for RNA-seq analyses.

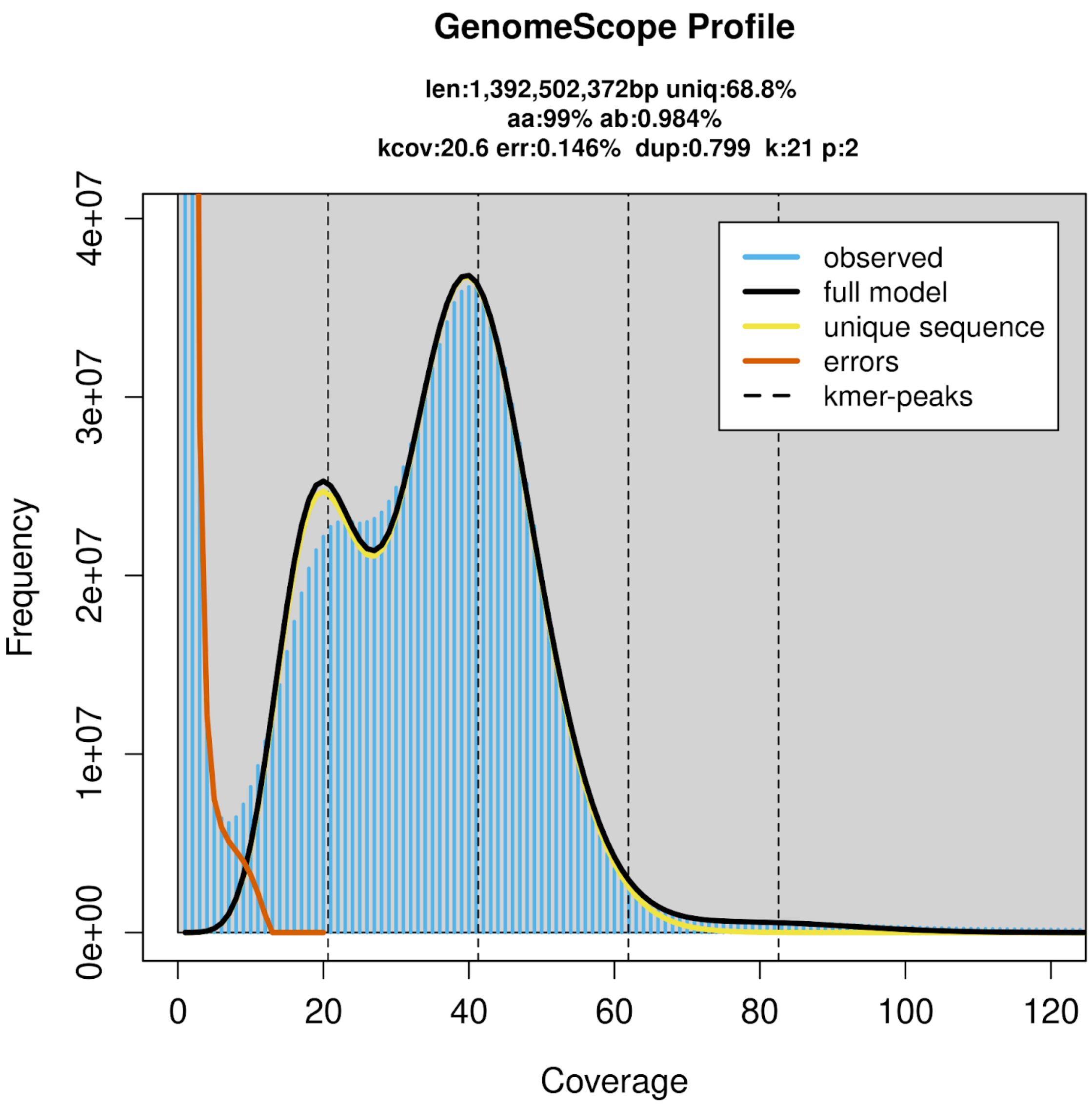

Fig. S3: Histogram from GenomeScope showing the frequency of reads in relation with their coverage.

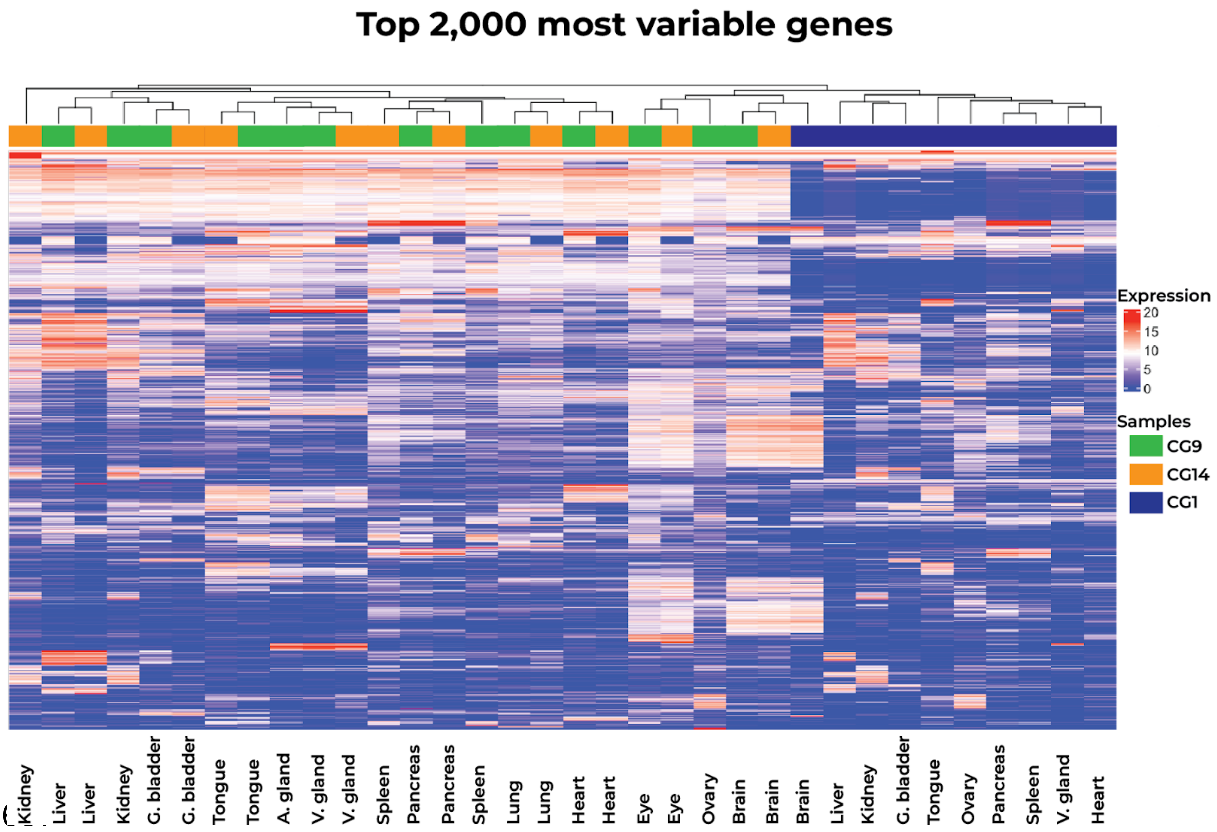

Fig. S4: Heatmap for the 2,000 most variable genes within our three samples, showing a clear batch effect of sample CG1 (possibly due to differences in sequencing time) as well as a high similarity between the putative accessory gland and the venom gland. Each column represents a different sampled tissue. The three different samples are depicted with different colors at the top of the heatmap. Abbreviations are as follows: G. bladder, gallbladder and V. gland, venom gland.

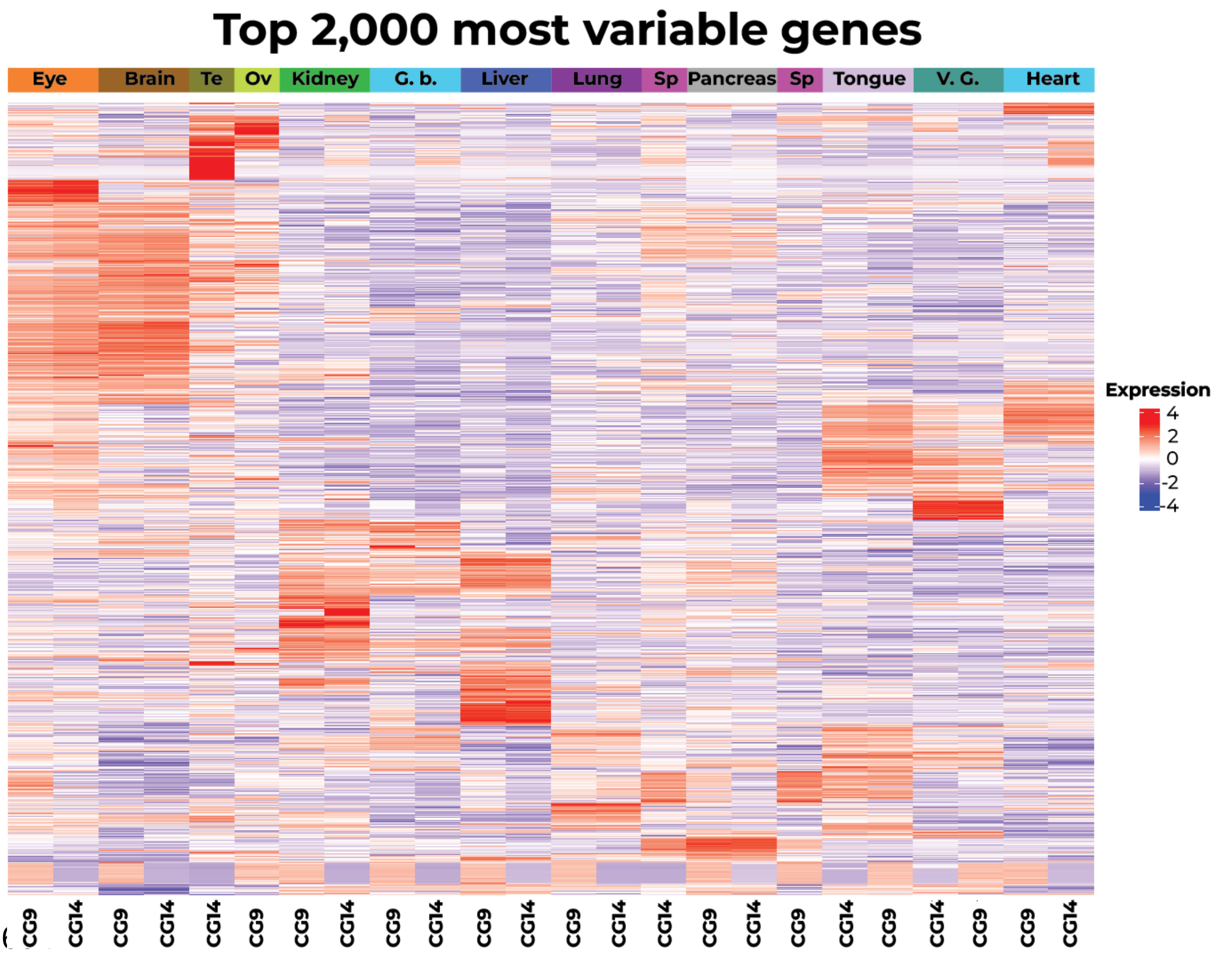

Fig. S5: Heatmap for the 2,000 most variable genes for both samples, reporting highly expressed genes unique for each tissue type. Each column represents one tissue sampled per individual. Expression levels were normalized. Abbreviations are as follows: Te, Testis; Ov, Ovary; G.b., gallbladder; Sp, Spleen and V.G., Venom gland.

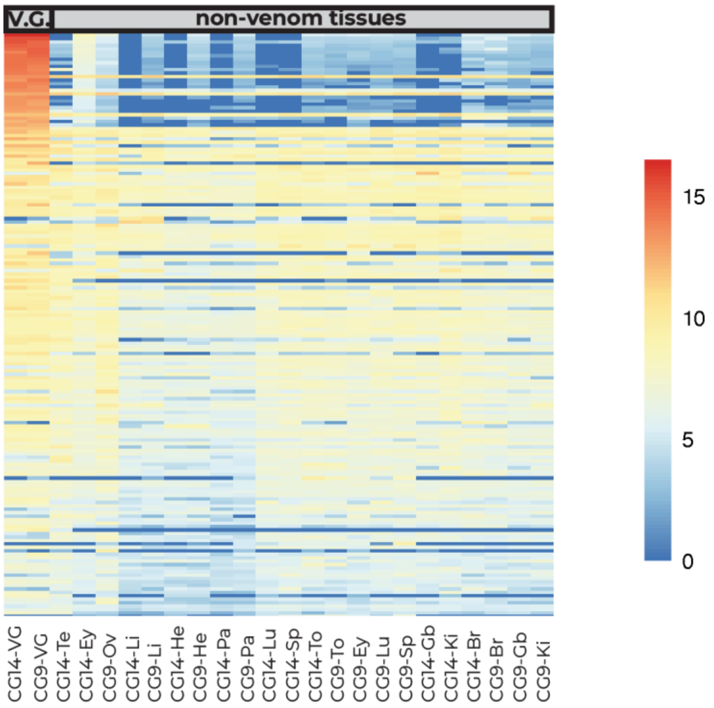

Fig. S6: Heatmap for the 161 upregulated genes found in the venom gland of *C. gasperettii* transcriptome including the 65 putative expressed toxins for both venom gland samples. Each column represents one tissue sampled per individual. Abbreviations are as follows: VG, Venom Gland; Ki, Kidney; GB, Gall Bladder; Lu; Lung; Sp, Spleen; He, Heart; Li, Liver; Pa, Pancreas; To, Tongue; Te, Testis; Ov, Ovary.

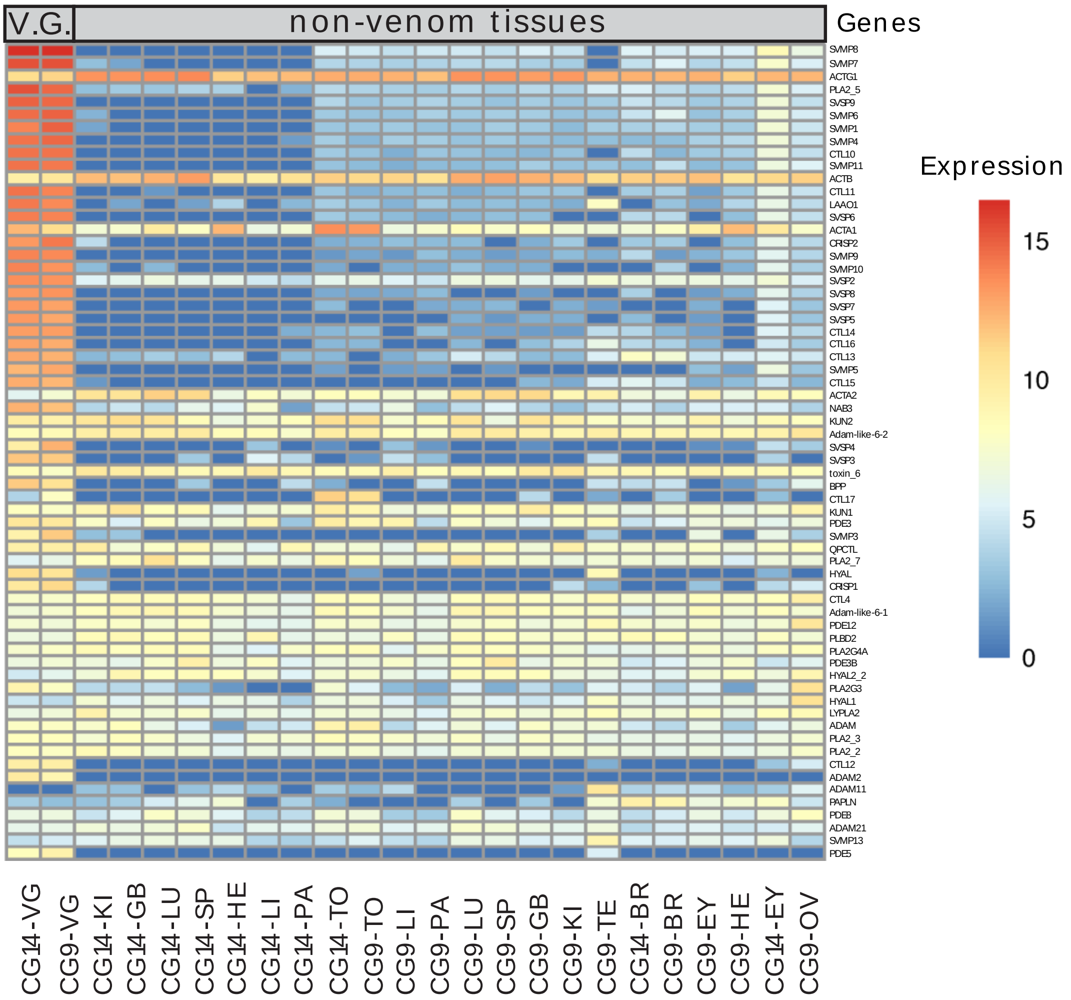

Fig. S7: Heatmap for the venom gland transcriptome for the 65 putative expressed toxins for both venom gland samples. Each column represents one tissue sampled per individual. Abbreviations are as follows: VG, Venom Gland; Ki, Kidney; GB, Gall Bladder; Lu; Lung; Sp, Spleen; He, Heart; Li, Liver; Pa, Pancreas; To, Tongue; Te, Testis; Ov, Ovary.

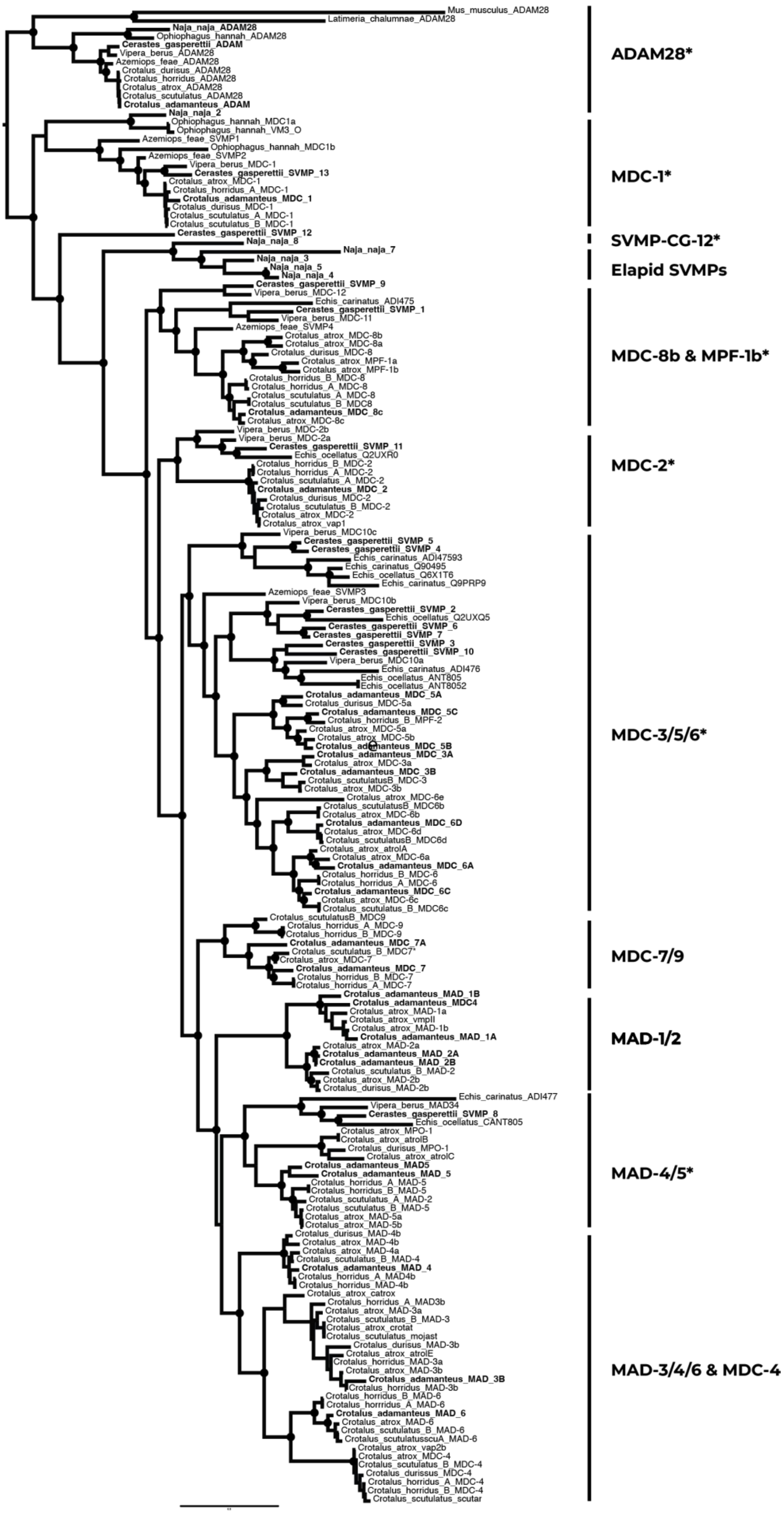

Fig. S8: Maximum

likelihood phylogeny for SVMP genes and its non-toxic paralog (ADAM28). Genes for *Cerastes gasperettii* are highlighted in bold. Toxin groups are identified following previous categorizations. Asterisks indicate if *Cerastes gasperettii* genes are present in that specific group. Branch support with aBayes values higher than 90 are depicted as circles.

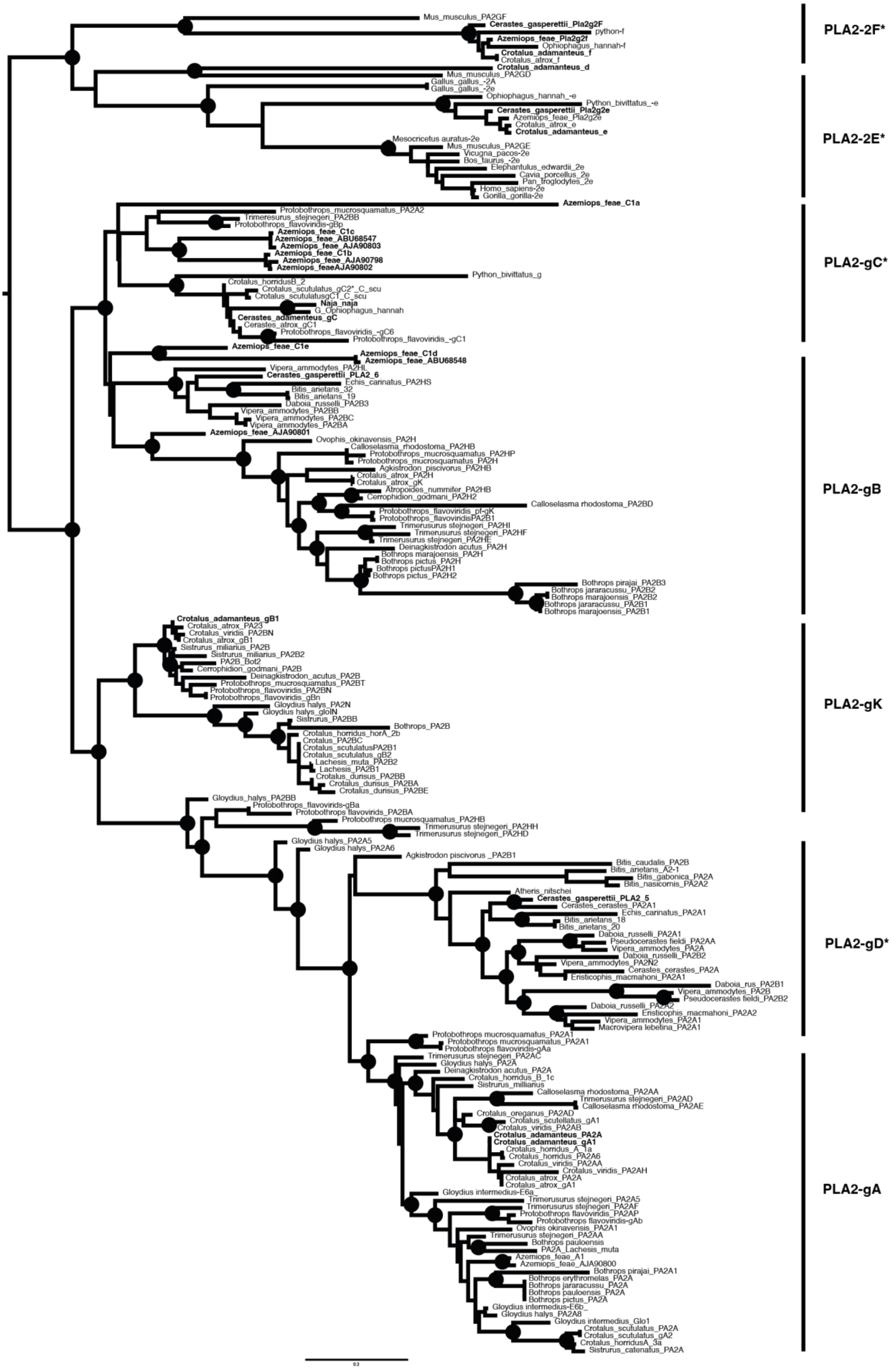

Fig. S9: Maximum likelihood phylogeny for PLA_2, with the two non-toxic genes as outgroups (PLA_2-

2F and PLA$_2$-2E). Asterisks in group labels indicate if *Cerastes gasperettii* genes are present in that specific group. Branch support with aBayes values higher than 90 are depicted as circles.

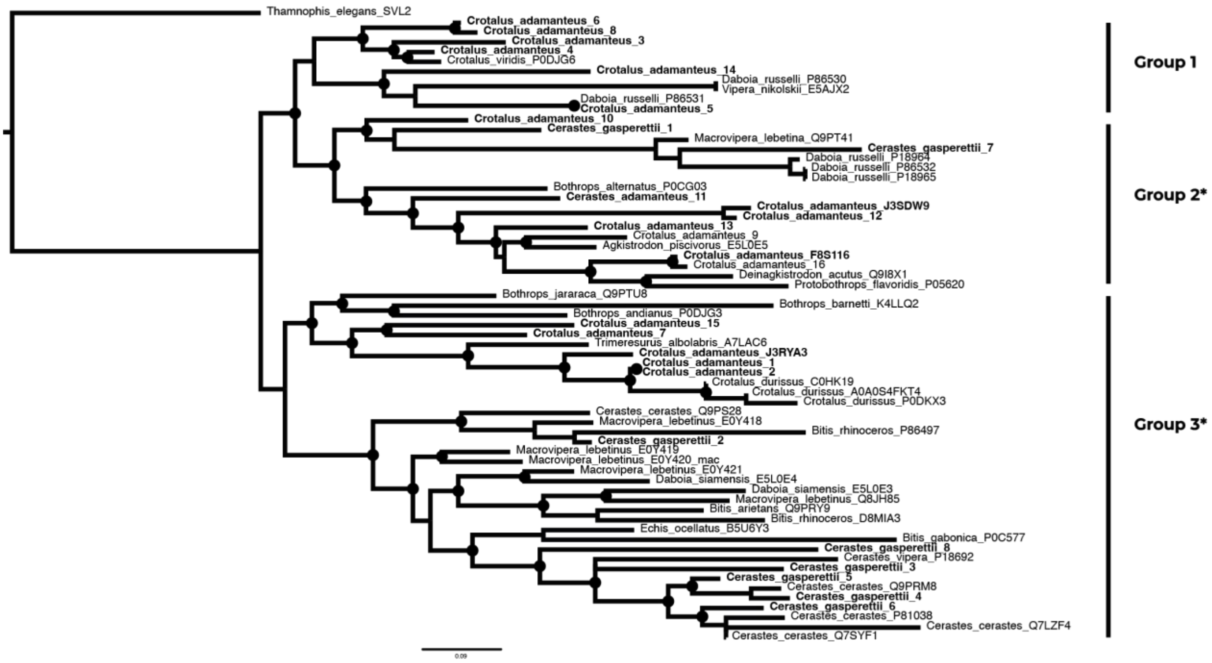

Fig. S10: Maximum likelihood phylogeny for SVSPs, with one sample from *Thamnophis elegans* as outgroup. Asterisks in group labels indicate if *Cerastes gasperettii* genes are present in that specific group. Branch support with aBayes values higher than 90 are depicted as circles.

Table S1: Individuals sampled in this study with their sex, sampling coordinates and data sequenced.

ID	Sex	Latitude	Longitude	Data sequenced
CG1	Female	25.284690	55.687860	HiFi, Omni-C, Illumina, RNA-seq, Iso-seq, Proteome
CG9	Female	25.284690	55.687860	RNA-seq
CG14	Male	25.284690	55.687860	RNA-seq

CN6134	-	UAE	UAE	Proteome
CN6135	-	UAE	UAE	Proteome

Table S2: Id, tissue type and number of reads sequenced per sample.

ID	Tissue	Reads
CG9	Tongue	44,672,733
CG9	Venom gland	41,124,132
CG9	Eye	41,951,109
CG9	Brain	42,800,966
CG9	Heart	40,715,947
CG9	Lung	42,518,938
CG9	Liver	42,251,137
CG9	Gallbladder	42,738,665
CG9	Spleen	40,909,550
CG9	Pancreas	40,527,010
CG9	Ovary	41,118,336
CG9	Kidney	40,620,023
CG9	Accessory gland	44,114,293
CG14	Tongue	41,455,346
CG14	Venom gland	41,035,764
CG14	Eye	40,753,220
CG14	Brain	43,413,973
CG14	Heart	42,338,980
CG14	Lung	42,068,410
CG14	Liver	21,549,210
CG14	Gallglabbder	50,571,941

CG14	Spleen	45,447,235
CG14	Pancreas	50,223,941
CG14	Testis	47,495,900
CG14	Kidney	45,945,776
CG1	Heart	47,362,067
CG1	Brain	45,740,571
CG1	Kidney	50,758,869
CG1	Gallbladder	40,546,711
CG1	Liver	48,058,958
CG1	Spleen	44,752,981
CG1	Tongue	46,837,490
CG1	Pancreas	45,023,783
CG1	Venom gland	49,775,424
CG1	Ovary	48,703,420

Table S3: Different types of repetitive elements masked within the genome:

Element	Number of elements	Length (bp)	Percentage
Retroelements	1524124	493932584	30.25 %
SINEs:	339152	55265721	3.38
Penelope	124778	19471740	1.19
LINEs:	988815	347028895	21.25
CRE/SLACS	0	0	0.00%
L2/CR1/Rex	480371	137654000	8.43
R1/LOA/Jockey	579	99034	0.01
R2/R4/NeSL	41793	10873028	0.67

RTE/Bov-B	128092	79663597	4.88
L1/CIN4	207974	95913575	5.87
LTR elements:	196157	91637968	5.61
BEL/Pao	16545	5263265	0.32
Ty1/Copia	25582	15088781	0.92
Gypsy/DIRS1	102598	63604234	3.90
Retroviral	50617	7642063	0.47
DNA transposons	707499	111444059	6.83
hobo-Activator	265944	30679712	1.88
Tc1-IS630-Pogo	227637	58877559	3.61
En-Spm	0	0	0.00%
MULE-MuDR	44	3962	0.00%
PiggyBac	138	6619	0.00%
Tourist/Harbinger	182161	18395721	1.13
Other	0	0	0.00%
Rolling-circles	2242	136656	0.01
Unclassified	205700	42385187	2.60
Total interspersed repeats	-	647761830	39.67
Small RNA	6134	652217	0.04
Satellites	35838	4217238	0.26
Simple repeats	765726	53044358	3.25
Low complexity	97863	6694649	0.41

Table S4: Abundances for the different toxin families identified in the proteome of *C. gasperettii*

Toxin family	Percentage
SVMPi	8.65%

DISI	12.74%
DC domain	0.26%
CRISP	4.34%
PLA2	5.47%
SVSP	37.38%
SVMP-III	22.19%
PDE	0.02%
LAAO	1.71%
CTL	7.25%

References

1. Dussex, N., van der Valk, T., Morales, H. E., Wheat, C. W., Díez-del-Molino, D., von Seth, J., Foster, Y., Kutschera, V. E., Guschanski, K., Rhie, A., Phillippy, A. M., Korlach, J., Howe, K., Chow, W., Pelan, S., Mendes Damas, J. D., Lewin, H. A., Hastie, A. R., Formenti, G., … Dalén, L. (2021). Population genomics of the critically endangered kākāpō. *Cell Genomics*, *1*(1), 100002. https://doi.org/10.1016/j.xgen.2021.100002
2. Hogan, M. P., Holding, M. L., Nystrom, G. S., Colston, T. J., Bartlett, D. A., Mason, A. J., Ellsworth, S. A., Rautsaw, R. M., Lawrence, K. C., Strickland, J. L., He, B., Fraser, P., Margres, M. J., Gilbert, D. M., Gibbs, H. L., Parkinson, C. L., & Rokyta, D. R. (2024). The genetic regulatory architecture and epigenomic basis for age-related changes in rattlesnake venom. *Proceedings of the National Academy of Sciences*, *121*(16), e2313440121. https://doi.org/10.1073/pnas.2313440121
3. Margres, M. J., Rautsaw, R. M., Strickland, J. L., Mason, A. J., Schramer, T. D., Hofmann, E. P., Stiers, E., Ellsworth, S. A., Nystrom, G. S., Hogan, M. P., Bartlett, D. A., Colston, T. J., Gilbert, D. M., Rokyta, D. R., & Parkinson, C. L. (2021). The Tiger Rattlesnake genome reveals a complex genotype underlying a simple venom phenotype. *Proceedings of the National Academy of Sciences*, *118*(4), e2014634118. https://doi.org/10.1073/pnas.2014634118
4. Pardos-Blas, J. R., Irisarri, I., Abalde, S., Afonso, C. M. L., Tenorio, M. J., & Zardoya, R. (2021). The genome of the venomous snail *Lautoconus ventricosus* sheds light on the origin of conotoxin diversity. *GigaScience*, *10*(5), giab037. https://doi.org/10.1093/gigascience/giab037
5. Schield, D. R., Card, D. C., Hales, N. R., Perry, B. W., Pasquesi, G. M., Blackmon, H., Adams, R. H., Corbin, A. B., Smith, C. F., Ramesh, B., Demuth, J. P., Betrán, E., Tollis, M., Meik, J. M., Mackessy, S. P., & Castoe, T. A. (2019). The origins and evolution of chromosomes, dosage compensation, and mechanisms underlying venom regulation in snakes. *Genome Research*, *29*(4), 590–601. https://doi.org/10.1101/gr.240952.118

6. Suryamohan, K., Krishnankutty, S. P., Guillory, J., Jevit, M., Schröder, M. S., Wu, M., Kuriakose, B., Mathew, O. K., Perumal, R. C., Koludarov, I., Goldstein, L. D., Senger, K., Dixon, M. D., Velayutham, D., Vargas, D., Chaudhuri, S., Muraleedharan, M., Goel, R., Chen, Y.-J. J., … Seshagiri, S. (2020). The Indian cobra reference genome and transcriptome enables comprehensive identification of venom toxins. *Nature Genetics*, *52*(1), 106–117. https://doi.org/10.1038/s41588-019-0559-8
7. Drukewitz, S. H., & Von Reumont, B. M. (2019). The Significance of Comparative Genomics in Modern Evolutionary Venomics. *Frontiers in Ecology and Evolution*, *7*, 163. https://doi.org/10.3389/fevo.2019.0016
8. Frantz, L. A. F., Bradley, D. G., Larson, G., & Orlando, L. (2020). Animal domestication in the era of ancient genomics. *Nature Reviews Genetics*, *21*(8), Article 8. https://doi.org/10.1038/s41576-020-0225-0
9. Orteu, A., & Jiggins, C. D. (2020). The genomics of coloration provides insights into adaptive evolution. *Nature Reviews Genetics*, *21*(8), Article 8. https://doi.org/10.1038/s41576-020-0234-z
10. San-Jose, L. M., & Roulin, A. (2017). Genomics of coloration in natural animal populations. *Philosophical Transactions of the Royal Society B: Biological Sciences*, *372*(1724), 20160337. https://doi.org/10.1098/rstb.2016.0337
11. Casewell, N. R., Wüster, W., Vonk, F. J., Harrison, R. A., & Fry, B. G. (2013). Complex cocktails: The evolutionary novelty of venoms. *Trends in Ecology & Evolution*, *28*(4), 219–229. https://doi.org/10.1016/j.tree.2012.10.020
12. Dowell, N. L., Giorgianni, M. W., Kassner, V. A., Selegue, J. E., Sanchez, E. E., & Carroll, S. B. (2016). The Deep Origin and Recent Loss of Venom Toxin Genes in Rattlesnakes. *Current Biology*, *26*(18), 2434–2445. https://doi.org/10.1016/j.cub.2016.07.038
13. Giorgianni, M. W., Dowell, N. L., Griffin, S., Kassner, V. A., Selegue, J. E., & Carroll, S. B. (2020). The origin and diversification of a novel protein family in venomous snakes. *Proceedings of the National Academy of Sciences*, *117*(20), 10911–10920. https://doi.org/10.1073/pnas.1920011117
14. Werren, J. H., Richards, S., Desjardins, C. A., Niehuis, O., Gadau, J., Colbourne, J. K., THE NASONIA GENOME WORKING GROUP, Beukeboom, L. W., Desplan, C., Elsik, C. G., Grimmelikhuijzen, C. J. P., Kitts, P., Lynch, J. A., Murphy, T., Oliveira, D. C. S. G., Smith, C. D., Zande, L. van de, Worley, K. C., Zdobnov, E. M., … Gibbs, R. A. (2010). Functional and Evolutionary Insights from the Genomes of Three Parasitoid Nasonia Species. *Science*, *327*(5963), 343–348. https://doi.org/10.1126/science.1178028
15. Fry, B. G., Roelants, K., Champagne, D. E., Scheib, H., Tyndall, J. D. A., King, G. F., Nevalainen, T. J., Norman, J. A., Lewis, R. J., Norton, R. S., Renjifo, C., & de la Vega, R. C. R. (2009). The toxicogenomic multiverse: Convergent recruitment of proteins into animal venoms. *Annual Review of Genomics and Human Genetics*, *10*, 483–511. https://doi.org/10.1146/annurev.genom.9.081307.164356
16. Zancolli, G., Reijnders, M., Waterhouse, R. M., & Robinson-Rechavi, M. (2022). Convergent evolution of venom gland transcriptomes across Metazoa. *Proceedings of the National Academy of Sciences*, *119*(1), e2111392119. https://doi.org/10.1073/pnas.2111392119
17. Vonk, F. J., Casewell, N. R., Henkel, C. V., Heimberg, A. M., Jansen, H. J., McCleary, R. J. R., Kerkkamp, H. M. E., Vos, R. A., Guerreiro, I., Calvete, J. J., Wüster, W., Woods, A. E., Logan, J. M., Harrison, R. A., Castoe, T. A., De Koning, A. P. J., Pollock, D. D., Yandell, M., Calderon, D., … Richardson, M. K. (2013). The king cobra genome reveals dynamic gene evolution and adaptation in the snake venom system. *Proceedings of the National Academy of Sciences*, *110*(51), 20651–20656. https://doi.org/10.1073/pnas.1314702110
18. Avella, I., Calvete, J. J., Sanz, L., Wüster, W., Licata, F., Quesada-Bernat, S., Rodríguez, Y., & Martínez-Freiría, F. (2022). Interpopulational variation and ontogenetic shift in the venom composition of Lataste's viper (*Vipera latastei*, Boscá 1878) from northern Portugal. *Journal of Proteomics*, *263*, 104613. https://doi.org/10.1016/j.jprot.2022.104613
19. Margres, M. J., Wray, K. P., Sanader, D., McDonald, P. J., Trumbull, L. M., Patton, A. H., & Rokyta, D. R. (2021). Varying Intensities of Introgression Obscure Incipient Venom-

Associated Speciation in the Timber Rattlesnake (*Crotalus horridus*). *Toxins*, *13*(11), Article 11. https://doi.org/10.3390/toxins13110782

20. King, G. F. (2011). Venoms as a platform for human drugs: Translating toxins into therapeutics. *Expert Opinion on Biological Therapy*, *11*(11), 1469–1484. https://doi.org/10.1517/14712598.2011.621940
21. Li, L., Huang, J., & Lin, Y. (2018). Snake Venoms in Cancer Therapy: Past, Present and Future. *Toxins*, *10*(9), 346. https://doi.org/10.3390/toxins10090346
22. Vyas, V. K., Brahmbhatt, K., Bhatt, H., & Parmar, U. (2013). Therapeutic potential of snake venom in cancer therapy: Current perspectives. *Asian Pacific Journal of Tropical Biomedicine*, *3*(2), 156–162. https://doi.org/10.1016/S2221-1691(13)60042-8
23. Williams, D. J., Faiz, M. A., Abela-Ridder, B., Ainsworth, S., Bulfone, T. C., Nickerson, A. D., Habib, A. G., Junghanss, T., Fan, H. W., Turner, M., Harrison, R. A., & Warrell, D. A. (2019). Strategy for a globally coordinated response to a priority neglected tropical disease: Snakebite envenoming. *PLOS Neglected Tropical Diseases*, *13*(2), e0007059. https://doi.org/10.1371/journal.pntd.0007059
24. Uetz, P. (2021). The Reptile Database: Curating the biodiversity literature without funding. *Biodiversity Information Science and Standards*, *5*, e75448. https://doi.org/10.3897/biss.5.75448
25. Fry, B. G., & Wüster, W. (2004). Assembling an Arsenal: Origin and Evolution of the Snake Venom Proteome Inferred from Phylogenetic Analysis of Toxin Sequences. *Molecular Biology and Evolution*, *21*(5), 870–883. https://doi.org/10.1093/molbev/msh091
26. Gutiérrez, J. M., Warrell, D. A., Williams, D. J., Jensen, S., Brown, N., Calvete, J. J., ... & Global Snakebite Initiative. (2013). The need for full integration of snakebite envenoming within a global strategy to combat the neglected tropical diseases: the way forward. *PLoS neglected tropical diseases*, *7*(6), e2162.
27. Gutiérrez, J. M., Calvete, J. J., Habib, A. G., Harrison, R. A., Williams, D. J., & Warrell, D. A. (2017). Snakebite envenoming. *Nature Reviews Disease Primers*, *3*(1), Article 1. https://doi.org/10.1038/nrdp.2017.63
28. Tasoulis, T., & Isbister, G. (2017). A Review and Database of Snake Venom Proteomes. *Toxins*, *9*(9), 290. https://doi.org/10.3390/toxins9090290
29. Weinstein, S. A., White, J., Keyler, D. E., & Warrell, D. A. (2013). Non-front-fanged colubroid snakes: A current evidence-based analysis of medical significance. *Toxicon*, *69*, 103–113. https://doi.org/10.1016/j.toxicon.2013.02.003
30. Ferraz, C. R., Arrahman, A., Xie, C., Casewell, N. R., Lewis, R. J., Kool, J., & Cardoso, F. C. (2019). Multifunctional Toxins in Snake Venoms and Therapeutic Implications: From Pain to Hemorrhage and Necrosis. *Frontiers in Ecology and Evolution*, *7*. https://www.frontiersin.org/articles/10.3389/fevo.2019.00218
31. Fry, B. (Ed.). (2015). *Venomous reptiles and their toxins: Evolution, pathophysiology, and biodiscovery*. Oxford University Press.
32. Fry, B. G., Scheib, H., van der Weerd, L., Young, B., McNaughtan, J., Ramjan, S. F. R., Vidal, N., Poelmann, R. E., & Norman, J. A. (2008). Evolution of an Arsenal: Structural and Functional Diversification of the Venom System in the Advanced Snakes (Caenophidia)*. *Molecular & Cellular Proteomics*, *7*(2), 215–246. https://doi.org/10.1074/mcp.M700094-MCP200
33. Osipov, A., & Utkin, Y. (2023). What Are the Neurotoxins in Hemotoxic Snake Venoms? *International Journal of Molecular Sciences*, *24*(3), Article 3. https://doi.org/10.3390/ijms24032919
34. Vitt, L. J., & Caldwell, J. P. (2014). *Herpetology: An introductory biology of amphibians and reptiles* (Fourth edition). Elsevier, AP, Academic Press is an imprint of Elsevier.
35. Arnold, N. E., Robinson, M. D., & Carranza, S. (2009). A preliminary analysis of phylogenetic relationships and biogeography of the dangerously venomous Carpet Vipers, *Echis* (Squamata, Serpentes, Viperidae) based on mitochondrial DNA sequences. *Amphibia Reptilia*, *30*(2), 273–282. https://doi.org/10.1163/156853809788201090

36. Casewell, N. R., Harrison, R. A., Wüster, W., & Wagstaff, S. C. (2009). Comparative venom gland transcriptome surveys of the saw-scaled vipers (Viperidae: *Echis*) reveal substantial intra-family gene diversity and novel venom transcripts. *BMC Genomics*, *10*(1), 564. https://doi.org/10.1186/1471-2164-10-564

37. Pook, C. E., Joger, U., Stümpel, N., & Wüster, W. (2009). When continents collide: Phylogeny, historical biogeography and systematics of the medically important viper genus *Echis* (Squamata: Serpentes: Viperidae). *Molecular Phylogenetics and Evolution*, *53*(3), 792–807. https://doi.org/10.1016/j.ympev.2009.08.002

38. Šmíd, J., & Tolley, K. A. (2019). Calibrating the tree of vipers under the fossilized birth-death model. *Scientific Reports*, *9*(1), 5510. https://doi.org/10.1038/s41598-019-41290-2

39. Wüster, W., Peppin, L., Pook, C. E., & Walker, D. E. (2008). A nesting of vipers: Phylogeny and historical biogeography of the Viperidae (Squamata: Serpentes). *Molecular Phylogenetics and Evolution*, *49*(2), 445–459. https://doi.org/10.1016/j.ympev.2008.08.019

40. Almeida, D. D., Viala, V. L., Nachtigall, P. G., Broe, M., Gibbs, H. L., Serrano, S. M. D. T., Moura-da-Silva, A. M., Ho, P. L., Nishiyama-Jr, M. Y., & Junqueira-de-Azevedo, I. L. M. (2021). Tracking the recruitment and evolution of snake toxins using the evolutionary context provided by the *Bothrops jararaca* genome. *Proceedings of the National Academy of Sciences*, *118*(20), e2015159118. https://doi.org/10.1073/pnas.2015159118

41. Myers, E. A., Strickland, J. L., Rautsaw, R. M., Mason, A. J., Schramer, T. D., Nystrom, G. S., Hogan, M. P., Yooseph, S., Rokyta, D. R., & Parkinson, C. L. (2022). De Novo Genome Assembly Highlights the Role of Lineage-Specific Gene Duplications in the Evolution of Venom in Fea's Viper (*Azemiops feae*). *Genome Biology and Evolution*, *14*(7), evac082. https://doi.org/10.1093/gbe/evac082

42. Hirst, Samuel R., Rhett M. Rautsaw, Cameron M. VanHorn, Marc A. Beer, Preston J. McDonald, Ramsés Alejandro Rosales García, Bruno Rodriguez Lopez et al. "Where the "ruber" Meets the Road: Using the Genome of the Red Diamond Rattlesnake to Unravel the Evolutionary Processes Driving Venom Evolution." *Genome Biology and Evolution* 16, no. 9 (2024): https://doi.org/10.1093/gbe/evae198

43. Gilbert, C., Meik, J. M., Dashevsky, D., Card, D. C., Castoe, T. A., & Schaack, S. (2014). Endogenous hepadnaviruses, bornaviruses and circoviruses in snakes. *Proceedings of the Royal Society B: Biological Sciences*, *281*(1791), 20141122. https://doi.org/10.1098/rspb.2014.1122

44. Westeen, E. P., Escalona, M., Holding, M. L., Beraut, E., Fairbairn, C., Marimuthu, M. P. A., Nguyen, O., Perri, R., Fisher, R. N., Toffelmier, E., Shaffer, H. B., & Wang, I. J. (2023). A genome assembly for the southern Pacific rattlesnake, *Crotalus oreganus helleri* , in the western rattlesnake species complex. *Journal of Heredity*, *114*(6), 681–689. https://doi.org/10.1093/jhered/esad045

45. Myers, E. A., Strickland, J. L., Rautsaw, R. M., Mason, A. J., Schramer, T. D., Nystrom, G. S., Hogan, M. P., Yooseph, S., Rokyta, D. R., & Parkinson, C. L. (2022). De Novo Genome Assembly Highlights the Role of Lineage-Specific Gene Duplications in the Evolution of Venom in Fea's Viper (*Azemiops feae*). *Genome Biology and Evolution*, *14*(7), evac082. https://doi.org/10.1093/gbe/evac082

46. Saethang, T., Somparn, P., Payungporn, S., Sriswasdi, S., Yee, K. T., Hodge, K., Knepper, M. A., Chanhome, L., Khow, O., Chaiyabutr, N., Sitprija, V., & Pisitkun, T. (2022). Identification of *Daboia siamensis* venome using integrated multi-omics data. *Scientific Reports*, *12*(1), Article 1. https://doi.org/10.1038/s41598-022-17300-1

47. Talavera, A., Palmada-Flores, M., Martínez-Freiría, F., Mochales-Riaño, G., Burriel-Carranza, B., Estarellas, M., Fernández-Guiberteau, D., Camina, Á., Ursenbacher, S., Vörös, J., & others. (2024). Unveiling the evolutionary history of European vipers and their venoms from a multi-omic approach. *bioRxiv*, 2024–12. https://doi.org/10.1101/2024.12.10.627732

48. Ali, S. A., Jackson, T. N. W., Casewell, N. R., Low, D. H. W., Rossi, S., Baumann, K., Fathinia, B., Visser, J., Nouwens, A., Hendrikx, I., Jones, A., Undheim, E. A., & Fry, B. G. (2015). Extreme venom variation in Middle Eastern vipers: A proteomics comparison of *Eristicophis*

macmahonii, *Pseudocerastes fieldi* and *Pseudocerastes persicus*. *Journal of Proteomics*, *116*, 106–113. https://doi.org/10.1016/j.jprot.2014.09.003

49. Mackessy, S. P. (2010). Evolutionary trends in venom composition in the Western Rattlesnakes (*Crotalus viridis* sensu lato): Toxicity vs. tenderizers. *Toxicon*, *55*(8), 1463–1474. https://doi.org/10.1016/j.toxicon.2010.02.028
50. Jan, V., Maroun, R. C., Robbe-Vincent, A., De Haro, L., & Choumet, V. (2002). Toxicity evolution of *Vipera aspis aspis* venom: Identification and molecular modeling of a novel phospholipase A2 heterodimer neurotoxin11Nucleotide sequence data reported are available in the EMBL database under the accession numbers AJ459806 and AJ459807. *FEBS Letters*, *527*(1), 263–268. https://doi.org/10.1016/S0014-5793(02)03205-2
51. Smith, C. F., Nikolakis, Z. L., Perry, B. W., Schield, D. R., Meik, J. M., Saviola, A. J., Castoe, T. A., Parker, J., & Mackessy, S. P. (2023). The best of both worlds? Rattlesnake hybrid zones generate complex combinations of divergent venom phenotypes that retain high toxicity. *Biochimie*. https://doi.org/10.1016/j.biochi.2023.07.008
52. Carranza, S., Els, J., & Burriel-Carranza, B. (2021). *A field guide to the reptiles of Oman*.
53. Mochales-Riaño, G., Burriel-Carranza, B., Barros, M. I., Velo-Antón, G., Talavera, A., Spilani, L., Tejero-Cicuéndez, H., Crochet, P.-A., Piris, A., García-Cardenete, L., Busais, S., Els, J., Shobrak, M., Brito, J. C., Šmíd, J., Carranza, S., & Martínez-Freiría, F. (2024). Hidden in the sand: Phylogenomics unravel an unexpected evolutionary history for the desert-adapted vipers of the genus *Cerastes*. *Molecular Phylogenetics and Evolution*, *191*, 107979. https://doi.org/10.1016/j.ympev.2023.107979
54. Russell, F. E., & Campbell, J. R. (2015). *Venomous terrestrial Snakes of the Middle East*. Edition Chimaira.
55. Al-Sadoon, M. K., & Paray, B. A. (2016). Ecological aspects of the horned viper, *Cerastes cerastes gasperettii* in the central region of Saudi Arabia. *Saudi Journal of Biological Sciences*, *23*(1), 135–138. https://doi.org/10.1016/j.sjbs.2015.10.010
56. Amr, Z. S., Abu Baker, M. A., & Warrell, D. A. (2020). Terrestrial venomous snakes and snakebites in the Arab countries of the Middle East. *Toxicon*, *177*, 1–15. https://doi.org/10.1016/j.toxicon.2020.01.012
57. Schneemann, M., Cathomas, R., Laidlaw, S. T., El Nahas, A. M., Theakston, R. D. G., & Warrell, D. A. (2004). Life-threatening envenoming by the Saharan horned viper (*Cerastes cerastes*) causing micro-angiopathic haemolysis, coagulopathy and acute renal failure: Clinical cases and review. *QJM: An International Journal of Medicine*, *97*(11), 717–727. https://doi.org/10.1093/qjmed/hch118
58. Rokyta, D. R., Margres, M. J., Ward, M. J., & Sanchez, E. E. (2017). The genetics of venom ontogeny in the eastern diamondback rattlesnake (*Crotalus adamanteus*). *PeerJ*, *5*, e3249. https://doi.org/10.7717/peerj.3249
59. Andrews, S. (2010). *FastQC: a quality control tool for high throughput sequence data.*
60. Martin, M. (2011). Cutadapt removes adapter sequences from high-throughput sequencing reads. *EMBnet.Journal*, *17*(1), 10. https://doi.org/10.14806/ej.17.1.200
61. Rhie, A., Walenz, B. P., Koren, S., & Phillippy, A. M. (2020). Merqury: Reference-free quality, completeness, and phasing assessment for genome assemblies. *Genome Biology*, *21*(1), 245. https://doi.org/10.1186/s13059-020-02134-9
62. Ranallo-Benavidez, T. R., Jaron, K. S., & Schatz, M. C. (2020). GenomeScope 2.0 and Smudgeplot for reference-free profiling of polyploid genomes. *Nature Communications*, *11*(1), Article 1. https://doi.org/10.1038/s41467-020-14998-3
63. Rhie, A., McCarthy, S. A., Fedrigo, O., Damas, J., Formenti, G., Koren, S., Uliano-Silva, M., Chow, W., Fungtammasan, A., Gedman, G. L., Cantin, L. J., Thibaud-Nissen, F., Haggerty, L., Lee, C., Ko, B. J., Kim, J., Bista, I., Smith, M., Haase, B., … Jarvis, E. D. (2020). Towards complete and error-free genome assemblies of all vertebrate species. Nature 592, 737–746 (2021). https://doi.org/10.1038/s41586-021-03451-0.
64. Cheng, H., Concepcion, G. T., Feng, X., Zhang, H., & Li, H. (2021). Haplotype-resolved de novo assembly using phased assembly graphs with hifiasm. *Nature Methods*, *18*(2), Article 2. https://doi.org/10.1038/s41592-020-01056-5

65. Guan, D., McCarthy, S. A., Wood, J., Howe, K., Wang, Y., & Durbin, R. (2020). Identifying and removing haplotypic duplication in primary genome assemblies. *Bioinformatics*, *36*(9), 2896–2898. https://doi.org/10.1093/bioinformatics/btaa025
66. Ghurye, J., Rhie, A., Walenz, B. P., Schmitt, A., Selvaraj, S., Pop, M., Phillippy, A. M., & Koren, S. (2019). Integrating Hi-C links with assembly graphs for chromosome-scale assembly. *PLoS Computational Biology*, *15*(8), e1007273. https://doi.org/10.1371/journal.pcbi.1007273
67. Walker, B. J., Abeel, T., Shea, T., Priest, M., Abouelliel, A., Sakthikumar, S., Cuomo, C. A., Zeng, Q., Wortman, J., Young, S. K., & Earl, A. M. (2014). Pilon: An Integrated Tool for Comprehensive Microbial Variant Detection and Genome Assembly Improvement. *PLOS ONE*, *9*(11), e112963. https://doi.org/10.1371/journal.pone.0112963
68. Jin, J.-J., Yu, W.-B., Yang, J.-B., Song, Y., dePamphilis, C. W., Yi, T.-S., & Li, D.-Z. (2020). GetOrganelle: A fast and versatile toolkit for accurate de novo assembly of organelle genomes. *Genome Biology*, *21*(1), 241. https://doi.org/10.1186/s13059-020-02154-5
69. Gurevich, A., Saveliev, V., Vyahhi, N., & Tesler, G. (2013). QUAST: Quality assessment tool for genome assemblies. *Bioinformatics (Oxford, England)*, *29*(8), 1072–1075. https://doi.org/10.1093/bioinformatics/btt086
70. Formenti, G., Abueg, L., Brajuka, A., Brajuka, N., Gallardo-Alba, C., Giani, A., Fedrigo, O., & Jarvis, E. D. (2022). Gfastats: Conversion, evaluation and manipulation of genome sequences using assembly graphs. *Bioinformatics*, *38*(17), 4214–4216. https://doi.org/10.1093/bioinformatics/btac460
71. Challis, R., Richards, E., Rajan, J., Cochrane, G., & Blaxter, M. (2020). BlobToolKit – Interactive Quality Assessment of Genome Assemblies. *G3 Genes|Genomes|Genetics*, *10*(4), 1361–1374. https://doi.org/10.1534/g3.119.400908
72. Allio, R., Schomaker-Bastos, A., Romiguier, J., Prosdocimi, F., Nabholz, B., & Delsuc, F. (2020). MitoFinder: Efficient automated large-scale extraction of mitogenomic data in target enrichment phylogenomics. *Molecular Ecology Resources*, *20*(4), 892–905. https://doi.org/10.1111/1755-0998.13160
73. Li, D., Luo, R., Liu, C.-M., Leung, C.-M., Ting, H.-F., Sadakane, K., Yamashita, H., & Lam, T.-W. (2016). MEGAHIT v1.0: A fast and scalable metagenome assembler driven by advanced methodologies and community practices. *Methods*, *102*, 3–11. https://doi.org/10.1016/j.ymeth.2016.02.020
74. Flynn, J. M., Hubley, R., Goubert, C., Rosen, J., Clark, A. G., Feschotte, C., & Smit, A. F. (2020). RepeatModeler2 for automated genomic discovery of transposable element families. *Proceedings of the National Academy of Sciences*, *117*(17), 9451–9457. https://doi.org/10.1073/pnas.1921046117
75. Tempel, S. (2012). Using and Understanding RepeatMasker. In Y. Bigot (Ed.), *Mobile Genetic Elements* (Vol. 859, pp. 29–51). Humana Press. https://doi.org/10.1007/978-1-61779-603-6_2
76. Bao, W., Kojima, K. K., & Kohany, O. (2015). Repbase Update, a database of repetitive elements in eukaryotic genomes. *Mobile DNA*, *6*(1), 11. https://doi.org/10.1186/s13100-015-0041-9
77. Keilwagen, J., Hartung, F., & Grau, J. (2019). GeMoMa: Homology-Based Gene Prediction Utilizing Intron Position Conservation and RNA-seq Data. In M. Kollmar (Ed.), *Gene Prediction* (Vol. 1962, pp. 161–177). Springer New York. https://doi.org/10.1007/978-1-4939-9173-0_9
78. Chen, S., Zhou, Y., Chen, Y., & Gu, J. (2018). Fastp: An ultra-fast all-in-one FASTQ preprocessor. *Bioinformatics*, *34*(17), i884–i890. https://doi.org/10.1093/bioinformatics/bty560
79. Kim, D., Paggi, J. M., Park, C., Bennett, C., & Salzberg, S. L. (2019). Graph-based genome alignment and genotyping with HISAT2 and HISAT-genotype. *Nature Biotechnology*, *37*(8), 907–915. https://doi.org/10.1038/s41587-019-0201-4
80. Tang, S., Lomsadze, A., & Borodovsky, M. (2015). Identification of protein coding regions in RNA transcripts. *Nucleic Acids Research*, *43*(12), e78–e78. https://doi.org/10.1093/nar/gkv227
81. Gabriel, L., Hoff, K. J., Brůna, T., Borodovsky, M., & Stanke, M. (2021). TSEBRA: Transcript selector for BRAKER. *BMC Bioinformatics*, *22*(1), 566. https://doi.org/10.1186/s12859-021-04482-0

82. Jones, P., Binns, D., Chang, H.-Y., Fraser, M., Li, W., McAnulla, C., McWilliam, H., Maslen, J., Mitchell, A., Nuka, G., Pesseat, S., Quinn, A. F., Sangrador-Vegas, A., Scheremetjew, M., Yong, S.-Y., Lopez, R., & Hunter, S. (2014). InterProScan 5: Genome-scale protein function classification. *Bioinformatics*, *30*(9), 1236–1240. https://doi.org/10.1093/bioinformatics/btu031
83. Dainat, J., Hereñú, D., Dr. K. D. Murray, Davis, E., Crouch, K., LucileSol, Agostinho, N., Pascal-Git, Zollman, Z., & Tayyrov. (2023). *NBISweden/AGAT: AGAT-v1.2.0* (v1.2.0) [Computer software]. Zenodo. https://doi.org/10.5281/ZENODO.3552717
84. Solovyev, V., Kosarev, P., Seledsov, I., & Vorobyev, D. (2006). Automatic annotation of eukaryotic genes, pseudogenes and promoters. *Genome Biology*, *7*(Suppl 1), S10. https://doi.org/10.1186/gb-2006-7-s1-s10
85. Geneva, A. J., Park, S., Bock, D. G., De Mello, P. L. H., Sarigol, F., Tollis, M., Donihue, C. M., Reynolds, R. G., Feiner, N., Rasys, A. M., Lauderdale, J. D., Minchey, S. G., Alcala, A. J., Infante, C. R., Kolbe, J. J., Schluter, D., Menke, D. B., & Losos, J. B. (2022). Chromosome-scale genome assembly of the brown anole (*Anolis sagrei*), an emerging model species. *Communications Biology*, *5*(1), 1126. https://doi.org/10.1038/s42003-022-04074-5
86. Tang, H., Bowers, J. E., Wang, X., Ming, R., Alam, M., & Paterson, A. H. (2008). Synteny and Collinearity in Plant Genomes. *Science*, *320*(5875), 486–488. https://doi.org/10.1126/science.1153917
87. Kiełbasa, S. M., Wan, R., Sato, K., Horton, P., & Frith, M. C. (2011). Adaptive seeds tame genomic sequence comparison. *Genome Research*, *21*(3), 487–493. https://doi.org/10.1101/gr.113985.110
88. Tang, H., Krishnakumar, V., Jingping Li, Tiany, MichelMoser, Maria, & Yim, W. C. (2017). *tanghaibao/jcvi: JCVI v0.7.5* (v0.7.5) [Computer software]. Zenodo. https://doi.org/10.5281/ZENODO.846919
89. Danecek, P., Bonfield, J. K., Liddle, J., Marshall, J., Ohan, V., Pollard, M. O., Whitwham, A., Keane, T., McCarthy, S. A., Davies, R. M., & Li, H. (2021). Twelve years of SAMtools and BCFtools. *GigaScience*, *10*(2). https://doi.org/10.1093/gigascience/giab008
90. Pertea, M., Pertea, G. M., Antonescu, C. M., Chang, T.-C., Mendell, J. T., & Salzberg, S. L. (2015). StringTie enables improved reconstruction of a transcriptome from RNA-seq reads. *Nature Biotechnology*, *33*(3), 290–295. https://doi.org/10.1038/nbt.3122
91. Love, M. I., Huber, W., & Anders, S. (2014). Moderated estimation of fold change and dispersion for RNA-seq data with DESeq2. *Genome Biology*, *15*(12), 550. https://doi.org/10.1186/s13059-014-0550-8
92. R Core Team. (2021a). *R: A Language and Environment for Statistical Computing*. R Foundation for Statistical Computing. https://www.R-project.org/
93. Calvete, J. J., Pla, D., Els, J., Carranza, S., Damm, M., Hempel, B.-F., John, E. B. O., Petras, D., Heiss, P., Nalbantsoy, A., Göçmen, B., Süssmuth, R. D., Calderón-Celis, F., Nosti, A. J., & Encinar, J. R. (2021). Combined Molecular and Elemental Mass Spectrometry Approaches for Absolute Quantification of Proteomes: Application to the Venomics Characterization of the Two Species of Desert Black Cobras, *Walterinnesia aegyptia* and *Walterinnesia morgani*. *Journal of Proteome Research*, *20*(11), 5064–5078. https://doi.org/10.1021/acs.jproteome.1c00608
94. Katoh, K., & Standley, D. M. (2013). MAFFT Multiple Sequence Alignment Software Version 7: Improvements in Performance and Usability. *Molecular Biology and Evolution*, *30*(4), 772–780. https://doi.org/10.1093/molbev/mst010
95. Hackl, T., Ankenbrand, M., van Adrichem, B., Wilkins, D., & Haslinger, K. (2024). Gggenomes: Effective and versatile visualizations for comparative genomics. (ArXiv). arXiv. https://doi.org/10.48550/arXiv.2411.13556
96. Myers, E. A., Strickland, J. L., Rautsaw, R. M., Mason, A. J., Schramer, T. D., Nystrom, G. S., Hogan, M. P., Yooseph, S., Rokyta, D. R., & Parkinson, C. L. (2022). De Novo Genome Assembly Highlights the Role of Lineage-Specific Gene Duplications in the Evolution of Venom in Fea's Viper (*Azemiops feae*). *Genome Biology and Evolution*, *14*(7), evac082. https://doi.org/10.1093/gbe/evac082

97. Guindon, S., Dufayard, J.-F., Lefort, V., Anisimova, M., Hordijk, W., & Gascuel, O. (2010). New Algorithms and Methods to Estimate Maximum-Likelihood Phylogenies: Assessing the Performance of PhyML 3.0. *Systematic Biology*, *59*(3), 307–321. https://doi.org/10.1093/sysbio/syq010
98. Li, H., & Durbin, R. (2011). Inference of human population history from individual whole-genome sequences. *Nature 2011 475:7357*, *475*(7357), 493–496. https://doi.org/10.1038/nature10231
99. Li, H., Handsaker, B., Wysoker, A., Fennell, T., Ruan, J., Homer, N., Marth, G., Abecasis, G., & Durbin, R. (2009). The Sequence Alignment/Map format and SAMtools. *Bioinformatics*, *25*(16), 2078–2079. https://doi.org/10.1093/bioinformatics/btp352
100. Green, R. E., Braun, E. L., Armstrong, J., Earl, D., Nguyen, N., Hickey, G., Vandewege, M. W., St. John, J. A., Capella-Gutiérrez, S., Castoe, T. A., Kern, C., Fujita, M. K., Opazo, J. C., Jurka, J., Kojima, K. K., Caballero, J., Hubley, R. M., Smit, A. F., Platt, R. N., … Ray, D. A. (2014). Three crocodilian genomes reveal ancestral patterns of evolution among archosaurs. *Science*, *346*(6215), 1254449. https://doi.org/10.1126/science.1254449
101. Schield, D. R., Perry, B. W., Adams, R. H., Holding, M. L., Nikolakis, Z. L., Gopalan, S. S., Smith, C. F., Parker, J. M., Meik, J. M., DeGiorgio, M., Mackessy, S. P., & Castoe, T. A. (2022). The roles of balancing selection and recombination in the evolution of rattlesnake venom. *Nature Ecology & Evolution*, *6*(9), 1367–1380. https://doi.org/10.1038/s41559-022-01829-5
102. Thongchum, R., Singchat, W., Laopichienpong, N., Tawichasri, P., Kraichak, E., Prakhongcheep, O., Sillapaprayoon, S., Muangmai, N., Baicharoen, S., Suntrarachun, S., Chanhome, L., Peyachoknagul, S., & Srikulnath, K. (2019). Diversity of PBI-DdeI satellite DNA in snakes correlates with rapid independent evolution and different functional roles. *Scientific Reports*, *9*(1), 15459. https://doi.org/10.1038/s41598-019-51863-w
103. Bylsma, R., Walkup, D. K., Hibbitts, T. J., Ryberg, W. A., Black, A. N., & DeWoody, J. A. (2022). Population genetic and genomic analyses of Western Massasauga (*Sistrurus tergeminus* ssp.): Implications for subspecies delimitation and conservation. *Conservation Genetics*, *23*(2), 271–283. https://doi.org/10.1007/s10592-021-01420-8
104. Li, H. (2013). *Aligning sequence reads, clone sequences and assembly contigs with BWA-MEM*. https://doi.org/10.48550/arXiv.1303.3997.
105. Broad Institute. (2021). *Picard Tools*. Broad Institute, GitHub Repository.
106. McKenna, A., Hanna, M., Banks, E., Sivachenko, A., Cibulskis, K., Kernytsky, A., Garimella, K., Altshuler, D., Gabriel, S., Daly, M., & DePristo, M. A. (2010). The genome analysis toolkit: A MapReduce framework for analyzing next-generation DNA sequencing data. *Genome Research*, *20*(9), 1297–1303. https://doi.org/10.1101/gr.107524.110
107. Wickham, H. (2016). *ggplot2: Elegant Graphics for Data Analysis*. Springer-Verlag New York. https://ggplot2.tidyverse.org
108. Simão, F. A., Waterhouse, R. M., Ioannidis, P., Kriventseva, E. V., & Zdobnov, E. M. (2015). BUSCO: Assessing genome assembly and annotation completeness with single-copy orthologs. *Bioinformatics (Oxford, England)*, *31*(19), 3210–3212. https://doi.org/10.1093/bioinformatics/btv351
109. Kalita, B., Mackessy, S. P., & Mukherjee, A. K. (2018). Proteomic analysis reveals geographic variation in venom composition of Russell's Viper in the Indian subcontinent: Implications for clinical manifestations post-envenomation and antivenom treatment. *Expert Review of Proteomics*, *15*(10), 837–849. https://doi.org/10.1080/14789450.2018.1528150
110. Casewell, N. R., Wagstaff, S. C., Wüster, W., Cook, D. A. N., Bolton, F. M. S., King, S. I., Pla, D., Sanz, L., Calvete, J. J., & Harrison, R. A. (2014). Medically important differences in snake venom composition are dictated by distinct postgenomic mechanisms. *Proceedings of the National Academy of Sciences*, *111*(25), 9205–9210. https://doi.org/10.1073/pnas.1405484111
111. Egan, D., Amr, Z., Al Johany, A., Els, J., Papenfuss, T., Nilson, Sadek, R., Disi, A., Hraoui-Bloquet, S., Werner, Y., & Anderson, S. (2012). *The IUCN Red List of Threatened Species: Cerastes gasperettii* [dataset]. https://doi.org/10.2305/IUCN.UK.2012.RLTS.T164599A1060588.en

112. Burriel-Carranza, B., Tejero-Cicuéndez, H., Carné, A., Riaño, G., Talavera, A., Saadi, S. A., Els, J., Šmíd, J., Tamar, K., Tarroso, P., & Carranza, S. (2023). The origin of a mountain biota: Hyper-aridity shaped reptile diversity in an Arabian biodiversity hotspot. https://doi.org/10.1101/2023.04.07.536010

113. Glennie, K. W., & Singhvi, A. K. (2002). Event stratigraphy, paleoenvironment and chronology of SE Arabian deserts. *Quaternary Science Reviews*, *21*(7), 853–869. https://doi.org/10.1016/S0277-3791(01)00133-0

114. Perez-Riverol Y, Bandla C, Kundu DJ, Kamatchinathan S, Bai J, Hewapathirana S, John NS, Prakash A, Walzer M, Wang S, Vizcaíno JA. The PRIDE database at 20 years: 2025 update. Nucleic Acids Res. 2025 Jan 6;53(D1):D543-D553. doi: 10.1093/nar/gkae1011.

115. Deutsch EW, Bandeira N, Perez-Riverol Y, Sharma V, Carver J, Mendoza L, Kundu DJ, Wang S, Bandla C, Kamatchinathan S, Hewapathirana S, Pullman B, Wertz J, Sun Z, Kawano S, Okuda S, Watanabe Y, MacLean B, MacCoss M, Zhu Y, Ishihama Y, Vizcaíno JA (2023). The ProteomeXchange Consortium at 10 years: 2023 update. Nucleic Acids Res. https://doi.org/10.1093/nar/gkac1040.

116. Mochales-Riaño G; Hirst SR; Talavera A; Burriel-Carranza B; Pagone V; Estarellas M; Busschau T; Boissinot S; Hogan MP; Tena-Garcés J; Pla D; Calvete JJ; Els J; Margres MJ; Carranza S (2025): Supporting data for "Chromosome-level reference genome for the medically important Arabian horned viper (Cerastes gasperettii)" GigaScience Database. https://doi.org/10.5524/102647.
