# Supplementary material for: Chromosome-level reference genome for the medically important Arabian horned viper (Cerastes gasperettii)
Source: Gigascience. 2025 Jun 6;14:giaf030. doi: 10.1093/gigascience/giaf030 (PMC12143202; doi:10.1093/gigascience/giaf030)
Supplement: giaf030_Authors_Response_To_Reviewer_Comments_original_submission [file giaf030_authors_response_to_reviewer_comments_original_submission.pdf]

Author's Response To Reviewer Comments

Reviewer #1: In the manuscript entitled 'Chromosome-level reference genome for the medically important Arabian horned viper (Cerastes gasperettii)', the authors assembled a high-quality chromosome-level reference genome for the Arabian horned viper (Cerastes gasperettii), a special Viperid species, which is an important data resource. Combined with multi omics data, the authors characterized the genome, conducted the analysis of toxin gene family, and identified a novel SVMP gene. The research is with great significance for the revelation of the origin and diversification of snake venom. Overall, I think the science and findings of the study are meaningful and merit publication, but in its current form, there are some issues should be noticed:

We thank reviewer 1 for his/her comments of our manuscript. We provide below a point-by-point answer to all his/her questions.

1. It should be noted that Fig. 1 and Fig. 2 both have unidentified border lines.

Sorry for that, we have now fixed it.

2. In all phylogenetic trees presented by the manuscript, it would be better for authors to indicate all species information.

The three toxin phylogenies have been modified and now all the tips include their species information.

3. I'm curious if the authors considered period differences in sampling, for example differences in venom glands after venom harvest or in the resting state, which could affect the analysis especially the transcriptome.

Before sampling the venom glands, venom was extracted, and snakes were allowed to recover for four days to maximize the venom gland transcription. This was absent from the text and now it has been added.

4. In the transcriptomics section, the author stated that the batch effect of CG1 was due to the low mapping of that sample to our reference genome. It is a misinterpretation to me as CG1 itself is the genome sequencing sample. The authors should further explain for this.

RNA-seq sequencing was not produced at the same time for the three samples, we first sequenced the rna-seq samples from the reference genome individual and later on the two other samples. We do not find any explanation to this, but both the low mapping as well as the different clustering of the tissues hampers the analyses of the three samples together. For that, we decided to exclude it from the analyses. We have clarified it in the text.

5. The authors need to ensure that all data generated by the manuscript is accessible and information about the data is not currently available.

All the NGS data was uploaded to ncbi and all the non-NGS data was uploaded to GigaScience's internal repository. We will talk with the Editor to solve this issue, raised by several reviewers, we are sorry for that.

6. Please check the references to ensure that the formatting meets the publisher's requirements, e.g., some Latin names of species requiring italics.

This has been fixed now, thanks.

Reviewer #2: Mochales-Riaño et al. present a high-quality genome assembly for the Arabian horned viper and provide a suite of genomic analyses related to synteny, toxin gene evolution and expression, genomic diversity, and demographic history of this and related species. This species is a valuable addition to existing snake genome resources given its medical significance and the current underrepresentation of genomes for Viperidae. I also appreciate that the authors sequenced the heterogametic sex and successfully assembled both sex chromosomes. I do have a few questions and concerns about the manuscript in its current form that I highlight below. Most notably, I feel that the arguments throughout the manuscript about toxin gene copy number correlating with proteomic abundance to be poorly supported and generally problematic given the data and analyses that the authors present. I suggest that the authors reevaluate these claims, and either provide additional analyses in an effort to support these claims or otherwise remove them from the manuscript, as I don't think they are ultimately crucial to the value of this genome report.

We thank reviewer 2 for his/her comments of our manuscript. We provide below a point-by-point answer to all his/her questions.

Introduction:
I find the argument being made in the sentence beginning "Previous works have shown that changes in gene regulation" a bit confusing. Rather than this arguing that studying the expression of venom genes is "insufficient," I think that this instead argues that transcriptomic and proteomic data are critical for studying venom in conjunction with annotated genome sequence. You could for example have a species with 20 copies in a particular tandem array, but only two of them are ever expressed at biologically meaningful levels and thus contribute proteins to the excreted venom. Knowing both the total number of copies in the genome and the number that are actually contributing to the venom proteome are both valuable and necessary for understanding the evolution of that gene family, its role and significance in venom phenotypes, etc.

We agree with reviewer 2 about this point and we have accordingly changed this section of the introduction to highlight how the combination of these techniques are necessary to study the evolution of venom. Now the paragraph reads like this: “Previous works have shown that changes in gene regulation can result in the activation and deactivation of venom-coding genes at all taxonomic levels and within the same individual (Avella et al., 2022; Hogan et al., 2024; Margres et al., 2021; Zancolli et al., 2022), suggesting that transcriptomic and proteomic data are critical for studying venom in conjunction with well annotated reference genomes to disentangle the complete number and biochemical nature of the toxins an individual can potentially transcribe (Drukewitz & Von Reumont, 2019). Ultimately, the study of venom genomics may yield insights into antivenom or drug discovery, as it can identify unexpressed toxin-coding genes that target specific physiological pathways, potentially leading to new therapies for human illnesses including but not limited to cancer (Casewell et al., 2013; King, 2011; L. Li et al., 2018).”

I'm also not sure I follow the logic of the next sentence. Why exactly would the identification of specifically "unexpressed" toxin genes be particularly notable for antivenom, drug discovery, therapeutics, etc.?

We have modified this sentence, now it reads like this:

“Ultimately, the study of venom genomics may yield insights into antivenom or drug discovery, as it enables the identification of unexpressed toxin-coding genes. These genes, often overlooked by transcriptomic or proteomic approaches unless ontogeny analyses or in-depth venom expression studies are performed, may target unique physiological pathways. Such discoveries could lead to novel therapies for human illnesses including but not limited to cancer.”

"We deciphered numerous genomic attributes of this species including its genetic diversity and failed to find evidence of inbreeding" - lack of inbreeding is never discussed in the context of the heterozygosity results, but is pitched here as a major result of the paper. Did the authors have a priori expectations regarding

inbreeding in this species?

It is true that we did not have a clear expectation of the genomic status. For this reason, we have now changed it to this:

“We deciphered numerous genomic attributes of this species, highlighting its adequate levels of genetic diversity”

Methods:
"Gene counts per gene…" - should this be "Gene expression counts per gene…"?

Reviewer 2 is right, we have included the word expression in this sentence, thank you for pointing it out.

Venom gland RNA-seq data was generated from three animals, but proteomic data was generated from a pool of two other animals. This is not ideal for linking gene expression to venom proteome composition, where you really would want venom collected from the same animals you are getting venom gland RNA from. This is especially true is there is intraspecific variation in venom phenotypes within this species. The latitude and longitude are not provided for the two proteome samples. Were these collected from the same latitude and longitude as the RNA-seq animals?

Intraspecific venom variation has not been described for this species, meaning that reviewer 2 is right and we should consider this. However, we were not able to extract venom from the reference individual and we do not have the exact coordinates for the two proteomic individuals. Although both individuals are from the same country as the reference genome, we have added that possible differences between transcriptomic and proteomic venom expression may rise due to geographical variation.

For analyses of heterozygosity, the authors map wgs data from diverse species against the cerastes reference and call variants. Why was this approach chosen over instead mapping the data for each species to either that species' reference (i.e., C. viridis and N. naja) or a more closely related species for those without a reference? Presumably that would reduce the potential influence of reference bias on these estimates of heterozygosity?

Yes, reviewer 2 is right and we may have reduced the heterozygosity levels of those species. We have reanalyzed each sample mapping it with the closest reference genome possible.

Results:
"Toxin genes usually found in venomous snakes (see proteome results below) were mainly found on macrochromosomes, although major toxin groups were found on microchromosomes (SVMPs, SVSPs and PLA2; Fig. 1)" this feels a bit contradictory. Maybe just can state that toxin genes were found on both macro and microchromosomes?

We have modified this section accordingly. Now it reads:

“Toxin genes were found in both macro- and microchromosomes (Fig. 1).”

"Finally, we also found a battery of 3FTxs and myotoxin-like genes, but they were not represented in our RNA-seq dataset (see below)." The authors do not further discuss this result as implied by "(see below)," unless that was simply referring to subsequent discussion of RNA-seq data. From what I can tell, these are also not present in the proteomic data, correct?

Exactly, we did not detect neither transcripts nor proteomic data for those two gene groups. We have now clarified it in the text.

"The venom gland transcriptome contained a total of 7,237 genes expressed (TPM > 500), including a total of 65 putative toxin genes. Differential gene expression analyses revealed a total of 161 genes (33 putative

toxin genes) that were differentially upregulated (FC > 2 and 1% FDR) in venom glands compared to other tissues (Fig. 3A)." Figure 3A only shows 10 toxin genes with "unique" expression in the venom gland, not the 161 upregulated toxin genes as implied here. The authors should add a heatmap with these 161 genes to the supplement, if not to Figure 3 (guessing it might not fit).

Thanks for pointing that out, we have added now two extra supplementary heatmaps (Fig. S6 and S7), one including the upregulated genes in the venom gland and a second one only focusing on the toxins.

Fig 3: The authors do not discuss the lack of unique/upregulated expression evidence for PLA2s and Disintegrins in Fig 3A, despite their contribution to protein composition in Fig 3B. For disintegrins in particular, they represent a higher proportion of the venom proteome than CTLs and CRISPs, yet there is no evidence presented for high expression in these genes. What do the authors think is going on here? Could this be a technical issue related to the processing of the RNAseq data, perhaps related to the small size of these genes? Alternatively, could this be indicative of a mismatch between venom phenotypes of the animals used to generate transcriptomic versus proteomic data?

We agree with the reviewer that we did not discuss some of the differences between RNA-seq and proteomic datasets, possibly (as suggested) due to individual differences. We have now included it along the text.

In the text, the authors state "These genes, together with other SVMPs, SVSPs, Disintegrins (DISI) and C-type lectins (CTL), were highly expressed in the venom gland and form the core toxic effector components of the venom" but again there is no presented evidence for DISI expression in particular. Are these genes included in the 161 upregulated genes in the venom gland?

This sentence was referring to the highly expressed genes in the venom gland, and as the reviewer mentions, DISI are not there. DISI has been removed from the sentence, thank you for pointing that out.

The authors only present proteomic data in the form of a pie chart of overall composition grouped by toxin family (Fig 3B). Does the proteomic data generated here provide individual gene-level proteomic abundance estimates? If so, this would be valuable to include, especially in support of the authors claims about gene copy number being correlated with protein abundance. For example in Figure 3, SVMP9 and SVMP10, and to a lesser extent SVMP13, are highly expressed and therefore possibly/likely the major contributors to SVMPs in the proteome. Is the SVMP section of the pie chart in Fig 3B dominated by proteins from these 3 genes?

We have now added one supplementary table (Table S4) with the values of figure 3B and a supplementary file including the proteomic abundance estimated at the gene-level. The claim about gene copy number being correlated with protein abundance is discussed below.

"We studied venom evolution within the most abundant toxin groups (i.e., SVMPs and SVSPs, as well as PLA2)." PLA2s are a relatively low proportion of the venom proteome in Fig 3B, and are not present in the expression heatmap in Fig 3A. Why were these chosen for further investigation over CTL, CRISP, DISI, etc.?

The reviewer is correct that the justification for including PLA2s in our comparative analyses is not very robust, as several other toxin families were found to be more abundant. However, genetic and genomic resources for PLA2s within snakes are more abundant than for other toxin families, paving the way to the study of this toxin family.

"The amplification of SVMP copy numbers is consistent with proteomic results, as SVMPs were the second most abundant component…". Related to my comment above, are all/many of these copies expressed in proteomic, or at least transcriptomic, data? As the data is currently presented, it appears that a small number of SVMPs are highly expressed and thus likely contributing to the proteome. This does not support, and might in fact contradict, the authors claim that there is an association with increased copy number and contribution to the proteome.

Regarding this fact, what we meant to say is that the toxin families that have undergone the highest number of gene duplication events throughout their evolution are also the most abundant in the proteome, regardless of whether all the genes in that toxin family are expressed or not. However, we agree with the reviewer that this statement could cause confusion, and therefore we have decided to remove it as well as other references for this claim along the text.

Related to this, and more generally, the authors do not present a convincing argument for the relationship between gene copy number and the resulting percentage of a given toxin gene family in the proteome. If copy number is directly related to the resulting amount of a toxin in the proteome, the authors would need to show that many/all of those copies are expressed in the transcriptomic data, and that proteins produced from those genes are present and contributing to the venom proteome (beyond just the total percentage for the family). Further, making any links between copy number and percent overall composition in the proteome is problematic, because it inherently is impacted by copy number variation and expression of all the other toxin genes. You could, in theory, have copy number expansion in a species where all the genes are expressed and contribute to the proteome, but no overall change in the percent of that toxin family in the proteome if other toxin families have
also expanded and/or are expressed more highly. Related to this, there is currently no obvious baseline to compare against in order to make these claims that expansion has resulted in higher venom proteome composition (i.e., a situation where we have fewer SVMP gene copies and a corresponding lower percentage of SVMP proteins in the venom proteome). This would potentially require comparison across species and/or populations with differing copy number, etc.

Thank you for this explanation, now it is clearer your specific point. We are aware that our data does not support this claim, and for this reason, we have decided to remove it from the manuscript.

My concerns above also apply to the interpretation of SVSP results: "The high number of SVSP genes found (although lower than in Crotalus adamanteus) were in line with the proteomic results, as SVSPs are the most abundant toxin in the proteome (Fig. 3B)." Further, C. adamanteus has a larger number of SVSP genes than C. gasperettii, yet a lower percent composition of SVSPs in the proteome (Margres et al. 2014), emphasizing my concerns about associating copy number and percent composition.

We agree with the reviewer and, as mentioned earlier, we have removed these claims from the manuscript. Thanks again for pointing it out.

Could the two large Group 2 SVSPs in Fig 4E be misannotations of multiple genes? Looking at the adamanteus genes above these, there genes starting and ending at roughly the same position the start and end of these large SVSPs, making me wonder if there are multiple cerastes genes that were annotated as one. In my own experience, I have seen similar situations where FGENESH+ was fed a large region containing multiple genes and annotated multiple genes together as one, so might just be worth double checking that that hasn't happened here. Alternatively, could these be gene fusions? If that's the case, that would presumably complicate the gene tree analyses, correct? i.e., these genes would probably need to excluded from those analyses.

The reviewer is right, thank you very much for spotting this. We have reannotated the region and we have identified the different genes. The different figures, as well as the annotation has been modified accordingly.

Reviewer #3: Dear Authors, thank you for compiling this resource and the manuscript. I apologise for the delay in my review. I have read the manuscript with great interest. I have some major concerns that need be addressed and a lot of minor concerns. Without line numbers, it was difficult to provide comments. I have chosen to write the part of the sentence that my comment refers to for you to consider for improvements.

We thank reviewer 3 for his/her comments of our manuscript. We are sorry for the lack of line numbers, they have been added now. We provide below a point-by-point answer to all his/her questions. We appreciate the thorough review provided, specially the minor concerns.

Major concerns:

- Abstract can include quantitative values for some key results such as the genome size, contiguity (e.g.N50, L90) and quality metrics (e.g. BUSCO) of the genome assembly among other result claims listed in the abstract.

We have now added quantitative metrics for the reference genome within the abstract.

- Venom as the keyword can perhaps be described/defined. Authors interchangeably use "venom", "toxin", "venom toxin", genes coding venom proteins. I strongly suggest the use of consistent terminologies that are well defined in the manuscript.

We have added venom as a keyword and we have carefully revised the manuscript regarding the use of the words venom, toxin, venom toxin and genes coding venom proteins, thanks for pointing that out.

- Methods need elaborate descriptions about reagents, procedures including for library preparations, sequencing machines, library kits and versions, etc. These are relevant for downstream analyses.

These details have been included, specially following the minor concerns suggested by the reviewer below. We hope it is more clear now.

- For all software, list parameters used, even if default, then explicitly state that "default parameters were used". For all software, list version numbers used for analyses.

We have incorporated parameters and version for the softwares.

- Authors are urged to change "macorsynteny" and "microsynteny" terms to chromosome level and local synteny analyses. This is to avoid confusion related to macro/microchromosomes.

We have accordingly modified the text with regard to both terms.

- "Genomic diversity" analyses use cross-species alignments and variant calling using software and methods developed for same species data. This can introduce significant bias in downstream interpretation and use of the variant data (heterozygosity measure may be). I suggest removal of this section because of lack of accuracy.

This concern was also raised by other reviewers, and we have decided to analyze each species using the closest available reference genome. This approach aims to minimize, as much as possible, the expected reduction in heterozygosity levels associated with mapping to a more distantly related species.

- Discussion of new discovery is largely lacking. I would appreciate if authors contextualized their results with other discoveries in the field.

We have have increased that section, now it reads like this:
“Our discovery of a novel SVMP gene in C. gasperettii adds to the growing body of work on the dynamic

evolution of venom systems. Similar gene expansions and duplications have been observed in other species, such as PLA2 toxin-coding genes found in the venom of Azemiops feae (Myers et al., 2022), highlighting the lineage-specific nature of venom evolution. The gene we identified, possibly arising from an SVMP13 duplication, do not share orthology with genes in other species, suggesting the presence of hidden toxin diversity in venom systems"
- Section headings in Results and Discussions can be changed to reflect main findings instead of "transcriptomics" or "genomic diversity".

Titles have been changed, thank you for the suggestion.

- One of the main findings is about SVMP gene family expansion. However, due to the lack of evidence about assembly accuracy in the region, accurate annotation of copies, and the effect of studying "primary assembly" instead of "haplotype assembly" at this region, I am not convinced of claims made in the paper. Appropriate justification is required for this section.

It may be that there was a misunderstanding regarding the used terms. We did work with haplotypic assemblies, as we obtain them directly from the assembler when incorporating the linkage data. When we mention primary assembly is in fact to remove duplicates present in the primary assembly to transfer them to the secondary assembly, but we do not work with an assembly that combines both haplotypes, this has been specified in the text.
Regarding the assembly accuracy of the region is as high as the current technologies allows it, and as proven by the metrics of the genome, our genome shows contiguity metrics similar to other high-quality reference genomes such as the recently published in PNAS by Hogan et al., (2024), with venom-coding genes assembled in single contigs. Regarding the accurate annotation of the copies, as stated in the manuscript we manually curated the venom regions combining both empirical and manual annotations.

- The nomenclature of SVMP genes is confusing. For example, In Figure 4A, they are all labelled as SVMPs with different colours, but then they are labelled as MDCs and MADs in Figure 4b and Supp Figure 6. Please label each gene in each species with consistent names that can reflect orthologous relationship. This is hard to discern, especially without appropriate species labels in Supp Figure 6.

We have modified the supplementary figures, highlighting in bold the genes involved in Fig. 4, so we hope this makes it easier to spot the orthology between groups. Moreover, now the supplementary figures contain appropriate species labels.

- Provide MSA files and trees used to infer evolutionary history. In the absence of the sequence alignments, and raw tree file, I am unable to evaluate this section of the manuscript. Please provide all required details for reviewers and readers.

All the NGS data was uploaded to ncbi and all the non-NGS data was uploaded to GigaScience's internal repository. We will talk with the Editor to solve this issue, raised by several reviewers, we are sorry for that.

??: It is not clear what authors mean by the word, term, phrase. Please correct them to convey accurate meaning using established and accepted scientific terminologies and English conventions.

Minor concerns:

Abstract:
- "compousing" ??
- "highly expressed toxin genes": in what tissues?
- "genome-wide diversity" ??
- "toxin gene families in venomous species" -> "toxin gene families in venomous snake species"

All these suggestions within the abstract have been modified, thanks.

Background:
- "Such advances in sequencing technologies": remove "Such"

We have removed it

- "depending on their type, interactions, and the organism": interactions with what?

The word interaction was changed.

- "proteomic (and transcriptomic) approaches": remove parenthesis

We have removed it.

- "to new therapies for human illnesses including but not": since the title contains "medically important", it would be great to include some specific examples here from the literature.

We have incorporated new literature citing examples about it.

- "However, venomous snakes are one": remove "However"

We have removed it.

- "therefore, the fundamental model system": change "fundamental" to "useful"

We have changed it.

- "of medical importance by the World Health Organization (WHO) due to their": provide citation

We provided a citation.

- "Within venomous snakes, the most medically": restructure the sentence for brevity and clarity.

We have modified the sentence.

- "cytotoxic effects (among others)": remove "(among others)"

We have removed it.

- "conducted using a proteomic approach": clarify what proteomic approach mean here.
- "Hirst et al., (in review);" : remove this citation

We have added it as citation as it is already available.

- "within the Viperidae family posses an available reference": change the word "posses" to something meaningful

We have changed it to contains.

- "Moreover, employing several -omics techniques": be specific about techniques

We have specified the techniques used.

- "We deciphered numerous genomic attributes": be specific

We have changed it to “We deciphered its adequate levels of genetic diversity.”

Methods:
- Describe how blood was extracted from animals with all details including animal handling techniques, body part etc.

We describe it now.

- "was stored in RNAlater until RNA extraction": source for RNAlater

It is from a company, we have specified it

- "We extracted gDNA from the blood of a female individual": provide additional details such as the quantity of blood used, thawing process, qty of reagents, especially elution buffer etc. Manufacturer protocols may be suited best for mammalian blood (humans, mice) without nucleus in RBCs unlike snakes.

No modifications were added to the protocol, but we have clarified it.

- "Then, we sequenced a total of two 8M SMRT HiFi cells, aiming for a ~30x of coverage, at the University of Leiden": provide details of library preparation, sequencing machine etc.

We have provided additional information.

- "(including venom glands, tongue, liver and pancreas, among others": Either list all or refer to the table.

We refer now to the table.

- "RNA libraries were prepared with the VAHTS": Was the library and sequencing strand specific? Provide complete details on these processes.

It was strand specific, now it is specified.

- "8M SMRT HiFi cell containing two Iso-seq HiFi libraries": use correct names of these and also include sequencing machine details.

We have changed the sentence as well as indicate the sequencing details.

- "Quality control on HiFi and Illumina reads was assessed using FastQC": correct the phrasing of this sentence

This has been corrected.

- "To make an initial exploration of the genome, …..we generated a k-mer profile with Meryl": Explicitly state the purpose of this analysis.

We have specified it: "In order to initially explore the genome size, heterozygosity levels and coverage data".

- "Manual curation was performed with Pretext": cite Pretext properly. Explain decisions of this manual curation. i.e. what evidence was used to join or break contigs.

Pretext does not have any scientific publication(or at least we did not find it), but we have provided the website.

- "Then, we ran three iterative rounds of RepeatMasker to annotate the known and unknown elements identified by RepeatModeler and soft-masked the genome for simple repeats": break this sentence into two and explain reasons for running RepeatMasker three times.

This has been specified.

- "We used GeMoMa v.1.9": Include all details about the annotations. This sentence is not sufficient for reproducibility. Were the RNAseq data assembled or provided as raw files to GeMoMa. How were they mapped to the genome assembly f

Details are included now.

- "published: Anolis carolinensis from Alföldi": Remove the word "from" here as citation is sufficient. Provide details of assembly versions, annotation version, database of annotations etc.

From was removed.

- "Crotalus ruber from Hirst et al., (in review)": remove this citation or list it as personal communication

As above, this has been included as the paper has been recently published.

- "We previously quality checked and removed the adapters of the RNA-seq data": remove "previously" and provide details on how adapters were removed from RNAseq data

We removed previously and provided information about how we removed the adapters with fastp.

- "also removed the adapters for the Iso-seq data": Explain how this was performed.

Same as above.

- "We blast our ..": Change all occurrence of "blast" to "BLAST" and specify parameters, if it was BLASTN or BLASTP or something else. This is not clear at all.

We have changed it and specified it.

- "we performed additional annotation steps for venom genes.": Details are not complete for reproducibility. State explicitly what decisions were made and how gene structure was determined. This is the main part of the paper and does require accurate details.

Details are following this sentence, we modified it and we hope now it is more clear.

- "Whole-genome synteny was explored between": synteny by definition refers to being on the same string/chromosome. Therefore whole-genome synteny as a term doesn't make sense given that genome is divided into chromosomes. Revise it to say "chromosomal synteny"

This has been changed.

- "chromosomes assembled in the reverse complement, which were corrected using SAMtools faidx": samtools faidx cannot do this. Explain how this was done.

We used samtools faidx with reverse-complement and mark-strand options.

- "After adapter trimming and quality control, we mapped our RNA-seq reads": how were adapters trimmed and QC implemented.
- "Gene counts per gene": change gene counts to read counts

This has been clarified now.

- "Differential expression analyses were carried out": requires additional details such as filters applied for the count, groups compared, statistical model, multiple testing correction methods.

This has been now added: “Prior to analysis, genes with fewer than 10 counts across all samples were filtered out. For comparisons, we defined two groups: venom glands versus all other tissues. DESeq2 employs a negative binomial generalized linear model to estimate differences in gene expression, and the p-values were adjusted for multiple testing using the Benjamini-Hochberg method to control the false discovery rate (FDR). Genes with an adjusted p-value < 0.01 and a fold change > 2 were considered significantly differentially expressed.”

- "characterize the venom arsenal of Cerastes gasperettii": change the arsenal word.

Changed.

- "Fragmentation spectra were matched against a customized database including the bony vertebrates taxonomy dataset of the NCBI non-redundant database": revise for accuracy

This has been revised.

- "Unmatched MS/MS spectra were de novo sequenced": spectra were sequenced how??"we used blast, incorporating both toxin and non-toxin paralogs": change blast to BLAST and provide additional details about the tool used

This was chaned and we provided additional details about it.

- "Then, we aligned those regions using Mafft (Katoh": provide coordinates of these regions for future research in each assembly

Coordinates are now provided.

- "history for the main groups of toxins (i.e.,": parenthesis is not closed.

We have now closed it.

- "we also included other non-toxin paralogous genes from nontoxic species (for details about this see Supplementary Information": where do I look into the supplementary information? Be very clear. Provide coordinates of regions that were compared.

Supplementary information was provided to GigaScience. We already have told them that reviewers were not able to access the data.

- "When needed, we translated CDS": when was this needed? Explain.

Now it is explained: “When nuclear sequences were obtained”.

- "built a phylogeny for each of the toxin groups using Phyml": I presume that this is done with translated CDS sequences in toxin genomic regions. Please clarify.

Yes, we have clarified it.

- "Heterozygous positions were obtained from bam files with Samtools v1.9": provide details as to how this was done. Samtools doesn't have features to operate at a site level and therefore I am confused.

We used the mpileup function of samtools, now it is clarified.

- "Filtered reads were mapped against the new reference genome of Cerastes gasperettii using the bwa mem algorithm": bwa mem is designed for same species comparisons. Here you have used it for cross-species. Provide justification and perhaps biases it may have introduced for distantly related species.

We have mapped the other species to closer reference genomes and every sampled was handled

independently. This is now clarified.

- "SNP calling was carried out …": This is not appropriate as models assume same species data. You have used cross-species alignments, which can be highly biased.

Same as above, thanks for pointing it out.

Results and Discussion:
- "PacBio HiFi (~40x), Hi-C (~60x) and Illumina data (~78x)": change to number of base pairs. 40x for a genome of 2GB is 80GB data and for genome of 1GB size, it is 40GB data. Before sequencing and assembly, the genome size cannot be known.
This has been changed to: "PacBio HiFi (65 Gbp of data), Hi-C (96 Gbp of data) and Illumina data (135 Gbp of data)".

- "After manual curation, we enhanced the scaffolding parameters of our genome": what was done as manual curation. Please specify.

It is an intrinsic outcome of the manual curation, we have modified it for a better understanding.

- "~228 times more contiguous than the Anolis sagrei genome": how is 228 more measured. How is this useful as a metric without the known ground truth. Assemblies can and do have errors.

It is true that assemblies have errors and they are not complete, but the contiguity value is a comparable metric between genomes, so one genome can be 228x more contiguous than another one, although it is not complete.

- "27,158 different protein-coding genes within our assembly": this seems large compared to other species. Can you elaborate or compare these numbers with other species.

We have recently published a gecko reference genome (Burriel-Carranza et al., 2024 Mol Ecol) with a total of 39,360 protein-coding genes, compared to which other species it is a large number?

- "Toxin genes usually found in venomous snakes (see proteome results below) were mainly found on macrochromosomes, although major toxin groups were found on microchromosomes (SVMPs, SVSPs and PLA2; Fig. 1)." : please revise this statement. Two part of the sentence are saying opposite things. Second provide coordinates of these genes as GFF/BED file as supplementary file with their exon structure annotations for others to reuse this information.

This was modified, as also indicated by another reviewer, coordinates are also provided in a previous comment of the same reviewer.

- "showed a great level of similarity between Cerastes gasperettii and Crotalus adamanteus": provide quantitative metrics for "great" level of similarity.

This sentence has been now modified.

- "we found several fission events in the A. sagrei genome,": Since A. sagrei genome is not contiguous and chromosome scale, you cannot infer fissions as it may be artefact of non-contiguous assembly. If that is not the case, provide evidence of this.

Anolis sagrei genome is at chromosome level as indicated in the publication
https://www.nature.com/articles/s42003-022-04074-5

- "The last four…": Belongs in methods

This has been now moved to methods.

- "Macrosyntenic differences between lizards and snakes": this is very
superficial discussion point. Please remove it or strengthen it with evidence.

We removed it, as it is not the point of the analyses or the paper.

- "Heatmap analyses with the most 2,000": Revise this statement. It doesn't make sense. E.g. Heatmap is a visualisation technique and not analyses method.

Thanks, we have removed it.

- "We studied venom evolution within the most abundant toxin groups": rewrite the sentence for clarity and brevity.

We have modified the sentence.

- "After a thorough manual curation": Explain what was this manual curation process clearly and the purpose of it.

This was explained in materials and methods

- "contiguous tandem repeat SVMPs for": Change "repeat" to "array" because tandem repeat has a different meaning in genomics research context.

Array is more acurate, thanks for the suggestion.

- "flanked by the NEFL and NEFM": Unclear if they are both 5' or 3' of toxin genes. Clarify

This has been clarified.

- "Microsyntenic analyses showed": change to local synteny

This has been chaned along the text.

- "gene copy number variation between": Since these are duplicate copies, clearly state how gene copies were identified. Include details of open reading frames, exon structures, pseudogene status, etc

Gene copies were identified through phylogeny methods, this has been now clarified in the text.

- "we can see an expansion in": Describe number of new copies, their status as intact or not, and sequence similarity between copies. Provide evidence that there is no false duplication due to heterozygous allele collapse in the assembly.

We provide details about it now: "Crotalus adamanteus (22 copies unique to vipers and 10 lineage-specific copies) but also in Cerastes gasperettii (12 copies unique to vipers and one lineage-specific copy)" Regarding the false duplication due to heterozygous alleles, this was already discussed above. During the assembly process, we removed duplicates from the primary assembly.

- "More genomic data will indicate if SVMP12": Did you mean SVMP13?
No, we mean SVMP12, as it is the new discovered gene.

- "This difference may be expected, as PLA2 only represents around 5% of the proteome for Cerastes gasperettii": This is not true. Proteome doesn't equal to genome in some cases and superficial inference such as this is not warranted.

We agree with the reviewer this sentence has been removed.

- For PSMC analyses, please discuss the effect of mutation rate and generation time.

We have added this sentence: “PSMC results may vary depending on the generation time as well as the mutational rate specified. The absence of species-specific data for this analyses may bias our results, although it is a general consensus in the literature when inferring these demographyc analyses in snakes (e.g. Schield et al., 2019).”

Figures:
- Figure 1: Add y-axis scales to the circos plot.

This has been added now.

- Figure 1b legend says it is a linkage map, but looks more like HiC contact map. Please edit.

We have now changed it, thanks.

- Figure 1b legend also says "including the sex chromosomes", which is not consistent with the circos plot.

During the HiC contact map, chromosomes were sorted by size, because it was not until the end that we identified the sexual chromosomes and in the final assembly they were moved to the end, for this reason there is this inconsistency.

- Figure 3A refers to transcriptome and 3b to proteome. Please make this very clear.
We have clarified it, thanks.

- Figure 4A, C and E, label genes consistent with the phylogenetic trees in supplementary figures so readers can know their genomic arrangements.

We have underscored the genes used in Figure 4A, C and E within the supplementary figures, because Figure 4 is already complex and labelling them would make it even more complex.

- Figure S4: Discuss why CG1 sample separates from rest of the samples. Seems like a batch effect.

We have added that information, thank you.

em Editorial Manager®

Cookie Preference Center

We use cookies which are necessary to make our site work. We may also use additional cookies to analyze, improve and personalize our content and your digital experience. For more information, see our Cookie Policy.

You may choose not to allow some types of cookies. However, blocking some types may impact your experience of our site and the services we are able to offer. See the different category headings below to find out more or change your settings.

Allow all

Manage Consent Preferences

Strictly Necessary Cookies

Always active

These cookies are necessary for the website to function and cannot be switched off in our systems. They are usually only set in response to actions made by you which amount to a request for services, such as setting your privacy preferences, logging in or filling in forms. You can set your browser to block or alert you about these cookies, but some parts of the site will not then work. These cookies do not store any personally identifiable information.

Functional Cookies

These cookies enable the website to provide enhanced functionality and personalisation. They may be set by us or by third party providers whose services we have added to our pages. If you do not allow these cookies then some or all of these services may not function properly.

Performance Cookies

These cookies allow us to count visits and traffic sources so we can measure and improve the performance of our site. They help us to know which pages are the most and least popular and see how visitors move around the site.

Targeting Cookies

These cookies may be set through our site by our advertising partners. They may be used by those companies to build a profile of your interests and show you relevant adverts on other sites. If you do not allow these cookies, you will experience less targeted advertising.

Cookie List

Clear

Apply Cancel

Consent Leg.Interest

Confirm my choices

Powered by onetrust
