# Supplementary material for: Chromosome-level reference genome for the medically important Arabian horned viper (Cerastes gasperettii)
Source: Gigascience. 2025 Jun 6;14:giaf030. doi: 10.1093/gigascience/giaf030 (PMC12143202; doi:10.1093/gigascience/giaf030)
Supplement: giaf030_Authors_Response_To_Reviewer_Comments_Revision_1 [file giaf030_authors_response_to_reviewer_comments_revision_1.pdf]

Author's Response To Reviewer Comments

Dear Hongfang Zhang,

Thank you for potentially accepting our manuscript. We have now incorporated the indicated suggestions by the reviewers. Below we provide a point-by-point justification of such changes and we hope that this version is now fit for publication in GigaScience.

Sincerely,
Gabriel Mochales Riaño

Reviewer #1: The author basically addressed my concerns that the manuscript could be published. There are still unidentified border lines in Fig. 1 only, but this could be a typographical problem?

We thank again reviewer one for his/her suggestions. Regarding the border's lines, we think it may be an artifact when the pdf is created, as we do not seen them in our figure.

Reviewer #2: The authors have conducted substantial revisions and I thank them for addressing my concerns. While I am generally satisfied with the revised version of the manuscript (in particular the removal of claims relating gene copy number to composition), I have a few minor, but important suggestions listed below. Most importantly, I think the authors need to be more explicit about the limitations resulting from the RNAseq and proteomic data being collected from separate animals which may or may not exhibit distinct venom phenotypes.

We thank again reviewer 2 for his/her thorough comments and suggestions along the text. We are sorry for not being clear enough regarding the limitations of having RNA-seq and proteomic data from different individuals, we have now included these sentences in line 392, in the section "toxins uniquely expressed in the venom gland" within results and conclusions:

"Transcriptomic and proteomic data was obtained from different individuals which may exhibit phenotypic differences in the venom due to several factors such as local adaptation or geographical isolation. We encourage future research to explore this possibility."

129: "We deciphered its adequate levels of genetic diversity" - I appreciate the authors addressing my concern in the original draft, but feel that this new sentence reads a bit vague. Perhaps something like "We assessed genetic diversity in comparison with other venomous snakes" or something like that?

Thanks for the suggestion, we have now changed it.

Line 396-398: Please be more explicit about the limitations of these comparisons by stating that you do not have gene expression and proteomic data from the same glands.

We have now added a sentence regarding this.

Related to this, I expressed concern in the initial review about the mismatch between RNA and proteomic data in Figure 3 for PLA2 and Disintegrins in particular, as they are large portions of the proteome but are not expressed highly in RNAseq data. Again, I think the authors should be explicit by highlighting this and any other major discrepancies between the RNA and proteomic data that may be driven by not sampling both from the same glands. This is important to highlight and will prevent confusion for the reader.

We have now highlighted these differences, thanks for pointing it out:
"However, transcriptomic and proteomic data for the same individuals should be sampled, as there are

several mismatches between RNA-seq and proteome data (Fig. 3). For example, PLA2 and Disintegrins, which are present in the proteome but they are not part of the transcriptomic results, which may be highlighting venom differences in our samples.”

Reviewer #3: Thank you for revising the manuscript. I have two minor comments remaining.
Thanks again for the suggestions. We provided below an answer to the following questions:

- Line 280: "Unmatched MS/MS spectra were de novo sequenced": this doesn't seem correct. They were not sequenced. They were matched/aligned to other snake toxin genes. Please correct the terminology here.

The MS/MS spectra of peptide ions unmatched in the genomic/transcriptomic reference databases were manually (de novo) sequenced and assigned by BLAST analysis to a known homologue venom toxin.

- Line 289-290: Thank you for providing coordinates. Please indicate the species for which these coordinates are for.

We have now indicated the species, thanks again.

em Editorial Manager®

Cookie Preference Center

We use cookies which are necessary to make our site work. We may also use additional cookies to analyze, improve and personalize our content and your digital experience. For more information, see our Cookie Policy.

You may choose not to allow some types of cookies. However, blocking some types may impact your experience of our site and the services we are able to offer. See the different category headings below to find out more or change your settings.

Allow all

Manage Consent Preferences

Strictly Necessary Cookies

Always active

These cookies are necessary for the website to function and cannot be switched off in our systems. They are usually only set in response to actions made by you which amount to a request for services, such as setting your privacy preferences, logging in or filling in forms. You can set your browser to block or alert you about these cookies, but some parts of the site will not then work. These cookies do not store any personally identifiable information.

Functional Cookies

These cookies enable the website to provide enhanced functionality and personalisation. They may be set by us or by third party providers whose services we have added to our pages. If you do not allow these cookies then some or all of these services may not function properly.

Performance Cookies

These cookies allow us to count visits and traffic sources so we can measure and improve the performance of our site. They help us to know which pages are the most and least popular and see how visitors move around the site.

Targeting Cookies

These cookies may be set through our site by our advertising partners. They may be used by those companies to build a profile of your interests and show you relevant adverts on other sites. If you do not allow these cookies, you will experience less targeted advertising.

Cookie List

Clear

Apply Cancel

Consent Leg.Interest

Confirm my choices

Powered by onetrus
