# Supplementary material for: Chromosome-level reference genome for the medically important Arabian horned viper (Cerastes gasperettii)
Source: Gigascience. 2025 Jun 6;14:giaf030. doi: 10.1093/gigascience/giaf030 (PMC12143202; doi:10.1093/gigascience/giaf030)
Supplement: giaf030_Reviewer_1_Report_original_submission — Jiatang Li -- 8/20/2024 [file giaf030_reviewer_1_report_original_submission.pdf]

Reviewer Report

Title: Chromosome-level reference genome for the medically important Arabian horned viper (Cerastes gasperettii)

Version: Original Submission **Date:** 8/20/2024

Reviewer name: Jiatang Li

Reviewer Comments to Author:

In the manuscript entitled 'Chromosome-level reference genome for the medically important Arabian horned viper (Cerastes gasperettii)', the authors assembled a high-quality chromosome-level reference genome for the Arabian horned viper (Cerastes gasperettii), a special Viperid species, which is an important data resource. Combined with multi omics data, the authors characterized the genome, conducted the analysis of toxin gene family, and identified a novel SVMP gene. The research is with great significance for the revelation of the origin and diversification of snake venom. Overall, I think the science and findings of the study are meaningful and merit publication, but in its current form, there are some issues should be noticed:

1. It should be noted that Fig. 1 and Fig. 2 both have unidentified border lines.
2. In all phylogenetic trees presented by the manuscript, it would be better for authors to indicate all species information.
3. I'm curious if the authors considered period differences in sampling, for example differences in venom glands after venom harvest or in the resting state, which could affect the analysis especially the transcriptome.
4. In the transcriptomics section, the author stated that the batch effect of CG1 was due to the low mapping of that sample to our reference genome. It is a misinterpretation to me as CG1 itself is the genome sequencing sample. The authors should further explain for this.
5. The authors need to ensure that all data generated by the manuscript is accessible and information about the data is not currently available.
6. Please check the references to ensure that the formatting meets the publisher's requirements, e.g., some Latin names of species requiring italics.

Methods

Are the methods appropriate to the aims of the study, are they well described, and are necessary controls included? Choose an item.

Conclusions

Are the conclusions adequately supported by the data shown? Choose an item.

Reporting Standards

Does the manuscript adhere to the journal's guidelines on [minimum standards of reporting?]() Choose an item.

Choose an item.

Statistics

Are you able to assess all statistics in the manuscript, including the appropriateness of statistical tests used? Choose an item.

Quality of Written English

Please indicate the quality of language in the manuscript: Choose an item.

Declaration of Competing Interests

Please complete a declaration of competing interests, considering the following questions:

- Have you in the past five years received reimbursements, fees, funding, or salary from an organisation that may in any way gain or lose financially from the publication of this manuscript, either now or in the future?
- Do you hold any stocks or shares in an organisation that may in any way gain or lose financially from the publication of this manuscript, either now or in the future?
- Do you hold or are you currently applying for any patents relating to the content of the manuscript?
- Have you received reimbursements, fees, funding, or salary from an organization that holds or has applied for patents relating to the content of the manuscript?
- Do you have any other financial competing interests?
- Do you have any non-financial competing interests in relation to this paper?

If you can answer no to all of the above, write 'I declare that I have no competing interests' below. If your reply is yes to any, please give details below.

I declare that I have no competing interests

I agree to the open peer review policy of the journal. I understand that my name will be included on my report to the authors and, if the manuscript is accepted for publication, my named report including any attachments I upload will be posted on the website along with the authors' responses. I agree for my report to be made available under an Open Access Creative Commons CC-BY license (http://creativecommons.org/licenses/by/4.0/). I understand that any comments which I do not wish to be included in my named report can be included as confidential comments to the editors, which will not be published.

Choose an item.

To further support our reviewers, we have joined with Publons, where you can gain additional credit to further highlight your hard work (see: https://publons.com/journal/530/gigascience). On publication of this paper, your review will be automatically added to Publons, you can then choose whether or not to claim your Publons credit. I understand this statement.

Yes Choose an item.
