# Supplementary material for: Chromosome-level reference genome for the medically important Arabian horned viper (Cerastes gasperettii)
Source: Gigascience. 2025 Jun 6;14:giaf030. doi: 10.1093/gigascience/giaf030 (PMC12143202; doi:10.1093/gigascience/giaf030)
Supplement: giaf030_Reviewer_2_Report_original_submission — Blair Perry -- 9/9/2024 [file giaf030_reviewer_2_report_original_submission.pdf]

**Reviewer Report**

**Title: Chromosome-level reference genome for the medically important Arabian horned viper (Cerastes gasperettii)**

**Version: Original Submission** **Date:** 9/9/2024

**Reviewer name: Blair Perry**

**Reviewer Comments to Author:**

Mochales-RiaÃ±o et al. present a high-quality genome assembly for the Arabian horned viper and provide a suite of genomic analyses related to synteny, toxin gene evolution and expression, genomic diversity, and demographic history of this and related species. This species is a valuable addition to existing snake genome resources given its medical significance and the current underrepresentation of genomes for Viperidae. I also appreciate that the authors sequenced the heterogametic sex and successfully assembled both sex chromosomes. I do have a few questions and concerns about the manuscript in its current form that I highlight below. Most notably, I feel that the arguments throughout the manuscript about toxin gene copy number correlating with proteomic abundance to be poorly supported and generally problematic given the data and analyses that the authors present. I suggest that the authors reevaluate these claims, and either provide additional analyses in an effort to support these claims or otherwise remove them from the manuscript, as I don't think they are ultimately crucial to the value of this genome report.
Introduction:
I find the argument being made in the sentence beginning "Previous works have shown that changes in gene regulation" a bit confusing. Rather than this arguing that studying the expression of venom genes is "insufficient," I think that this instead argues that transcriptomic and proteomic data are critical for studying venom in conjunction with annotated genome sequence. You could for example have a species with 20 copies in a particular tandem array, but only two of them are ever expressed at biologically meaningful levels and thus contribute proteins to the excreted venom. Knowing both the total number of copies in the genome and the number that are actually contributing to the venom proteome are both valuable and necessary for understanding the evolution of that gene family, its role and significance in venom phenotypes, etc.
I'm also not sure I follow the logic of the next sentence. Why exactly would the identification of specifically "unexpressed" toxin genes be particularly notable for antivenom, drug discovery, therapeutics, etc.?
"We deciphered numerous genomic attributes of this species including its genetic diversity and failed to find evidence of inbreeding" - lack of inbreeding is never discussed in the context of the heterozygosity results, but is pitched here as a major result of the paper. Did the authors have a priori expectations regarding inbreeding in this species?
Methods:
"Gene counts per gene…" - should this be "Gene expression counts per gene…"?
Venom gland RNA-seq data was generated from three animals, but proteomic data was generated from

a pool of two other animals. This is not ideal for linking gene expression to venom proteome composition, where you really would want venom collected from the same animals you are getting venom gland RNA from. This is especially true is there is intraspecific variation in venom phenotypes within this species. The latitude and longitude are not provided for the two proteome samples. Were these collected from the same latitude and longitude as the RNA-seq animals?

For analyses of heterozygosity, the authors map wgs data from diverse species against the cerastes reference and call variants. Why was this approach chosen over instead mapping the data for each species to either that species' reference (i.e., C. viridis and N. naja) or a more closely related species for those without a reference? Presumably that would reduce the potential influence of reference bias on these estimates of heterozygosity?

Results:

"Toxin genes usually found in venomous snakes (see proteome results below) were mainly found on macrochromosomes, although major toxin groups were found on microchromosomes (SVMPs, SVSPs and PLA2; Fig. 1)" this feels a bit contradictory. Maybe just can state that toxin genes were found on both macro and microchromosomes?

"Finally, we also found a battery of 3FTxs and myotoxin-like genes, but they were not represented in our RNA-seq dataset (see below)." The authors do not further discuss this result as implied by "(see below)," unless that was simply referring to subsequent discussion of RNA-seq data. From what I can tell, these are also not present in the proteomic data, correct?

"The venom gland transcriptome contained a total of 7,237 genes expressed (TPM > 500), including a total of 65 putative toxin genes. Differential gene expression analyses revealed a total of 161 genes (33 putative toxin genes) that were differentially upregulated (FC > 2 and 1% FDR) in venom glands compared to other tissues (Fig. 3A)." Figure 3A only shows 10 toxin genes with "unique" expression in the venom gland, not the 161 upregulated toxin genes as implied here. The authors should add a heatmap with these 161 genes to the supplement, if not to Figure 3 (guessing it might not fit).

Fig 3: The authors do not discuss the lack of unique/upregulated expression evidence for PLA2s and Disintegrins in Fig 3A, despite their contribution to protein composition in Fig 3B. For disintegrins in particular, they represent a higher proportion of the venom proteome than CTLs and CRISPs, yet there is no evidence presented for high expression in these genes. What do the authors think is going on here? Could this be a technical issue related to the processing of the RNAseq data, perhaps related to the small size of these genes? Alternatively, could this be indicative of a mismatch between venom phenotypes of the animals used to generate transcriptomic versus proteomic data?

In the text, the authors state "These genes, together with other SVMPs, SVSPs, Disintegrins (DISI) and C-type lectins (CTL), were highly expressed in the venom gland and form the core toxic effector components of the venom" but again there is no presented evidence for DISI expression in particular. Are these genes included in the 161 upregulated genes in the venom gland?

The authors only present proteomic data in the form of a pie chart of overall composition grouped by toxin family (Fig 3B). Does the proteomic data generated here provide individual gene-level proteomic abundance estimates? If so, this would be valuable to include, especially in support of the authors claims about gene copy number being correlated with protein abundance. For example in Figure 3, SVMP9 and SVMP10, and to a lesser extent SVMP13, are highly expressed and therefore possibly/likely the major contributors to SVMPs in the proteome. Is the SVMP section of the pie chart in Fig 3B dominated by

proteins from these 3 genes?

"We studied venom evolution within the most abundant toxin groups (i.e., SVMPs and SVSPs, as well as PLA2)." PLA2s are a relatively low proportion of the venom proteome in Fig 3B, and are not present in the expression heatmap in Fig 3A. Why were these chosen for further investigation over CTL, CRISP, DISI, etc.?

"The amplification of SVMP copy numbers is consistent with proteomic results, as SVMPs were the second most abundant component…". Related to my comment above, are all/many of these copies expressed in proteomic, or at least transcriptomic, data? As the data is currently presented, it appears that a small number of SVMPs are highly expressed and thus likely contributing to the proteome. This does not support, and might in fact contradict, the authors claim that there is an association with increased copy number and contribution to the proteome.

Related to this, and more generally, the authors do not present a convincing argument for the relationship between gene copy number and the resulting percentage of a given toxin gene family in the proteome. If copy number is directly related to the resulting amount of a toxin in the proteome, the authors would need to show that many/all of those copies are expressed in the transcriptomic data, and that proteins produced from those genes are present and contributing to the venom proteome (beyond just the total percentage for the family). Further, making any links between copy number and percent overall composition in the proteome is problematic, because it inherently is impacted by copy number variation and expression of all the other toxin genes. You could, in theory, have copy number expansion in a species where all the genes are expressed and contribute to the proteome, but no overall change in the percent of that toxin family in the proteome if other toxin families have also expanded and/or are expressed more highly. Related to this, there is currently no obvious baseline to compare against in order to make these claims that expansion has resulted in higher venom proteome composition (i.e., a situation where we have fewer SVMP gene copies and a corresponding lower percentage of SVMP proteins in the venom proteome). This would potentially require comparison across species and/or populations with differing copy number, etc.

My concerns above also apply to the interpretation of SVSP results: "The high number of SVSP genes found (although lower than in Crotalus adamanteus) were in line with the proteomic results, as SVSPs are the most abundant toxin in the proteome (Fig. 3B)." Further, C. adamanteus has a larger number of SVSP genes than C. gasperettii, yet a lower percent composition of SVSPs in the proteome (Margres et al. 2014), emphasizing my concerns about associating copy number and percent composition.

Could the two large Group 2 SVSPs in Fig 4E be misannotations of multiple genes? Looking at the adamanteus genes above these, there genes starting and ending at roughly the same position the start and end of these large SVSPs, making me wonder if there are multiple cerastes genes that were annotated as one. In my own experience, I have seen similar situations where FGENESH+ was fed a large region containing multiple genes and annotated multiple genes together as one, so might just be worth double checking that that hasn't happened here. Alternatively, could these be gene fusions? If that's the case, that would presumably complicate the gene tree analyses, correct? i.e., these genes would probably need to excluded from those analyses.

**Methods**

Are the methods appropriate to the aims of the study, are they well described, and are necessary controls included? Choose an item.

**Conclusions**

Are the conclusions adequately supported by the data shown? Choose an item.

**Reporting Standards**

Does the manuscript adhere to the journal's guidelines on minimum standards of reporting? Choose an item.

Choose an item.

**Statistics**

Are you able to assess all statistics in the manuscript, including the appropriateness of statistical tests used? Choose an item.

**Quality of Written English**

Please indicate the quality of language in the manuscript: Choose an item.

**Declaration of Competing Interests**

Please complete a declaration of competing interests, considering the following questions:

- Have you in the past five years received reimbursements, fees, funding, or salary from an organisation that may in any way gain or lose financially from the publication of this manuscript, either now or in the future?
- Do you hold any stocks or shares in an organisation that may in any way gain or lose financially from the publication of this manuscript, either now or in the future?
- Do you hold or are you currently applying for any patents relating to the content of the manuscript?
- Have you received reimbursements, fees, funding, or salary from an organization that holds or has applied for patents relating to the content of the manuscript?
- Do you have any other financial competing interests?
- Do you have any non-financial competing interests in relation to this paper?

If you can answer no to all of the above, write 'I declare that I have no competing interests' below. If your reply is yes to any, please give details below.

I declare that I have no competing interests.

I agree to the open peer review policy of the journal. I understand that my name will be included on my report to the authors and, if the manuscript is accepted for publication, my named report including any attachments I upload will be posted on the website along with the authors' responses. I agree for my report to be made available under an Open Access Creative Commons CC-BY license (http://creativecommons.org/licenses/by/4.0/). I understand that any comments which I do not wish to be included in my named report can be included as confidential comments to the editors, which will not be published.

Choose an item.

To further support our reviewers, we have joined with Publons, where you can gain additional credit to further highlight your hard work (see: https://publons.com/journal/530/gigascience). On publication of this paper, your review will be automatically added to Publons, you can then choose whether or not to claim your Publons credit. I understand this statement.

Yes Choose an item.
