# Supplementary material for: Chromosome-level reference genome for the medically important Arabian horned viper (Cerastes gasperettii)
Source: Gigascience. 2025 Jun 6;14:giaf030. doi: 10.1093/gigascience/giaf030 (PMC12143202; doi:10.1093/gigascience/giaf030)
Supplement: giaf030_Reviewer_3_Report_original_submission — Hardip Patel -- 9/11/2024 [file giaf030_reviewer_3_report_original_submission.pdf]

Reviewer Report

Title: Chromosome-level reference genome for the medically important Arabian horned viper (Cerastes gasperettii)

Version: Original Submission **Date:** 9/11/2024

Reviewer name: Hardip Patel

Reviewer Comments to Author:

Dear Authors, thank you for compiling this resource and the manuscript. I apologise for the delay in my review. I have read the manuscript with great interest. I have some major concerns that need be addressed and a lot of minor concerns. Without line numbers, it was difficult to provide comments. I have chosen to write the part of the sentence that my comment refers to for you to consider for improvements.

Major concerns:

- Abstract can include quantitative values for some key results such as the genome size, contiguity (e.g.N50, L90) and quality metrics (e.g. BUSCO) of the genome assembly among other result claims listed in the abstract.
- Venom as the keyword can perhaps be described/defined. Authors interchangeably use "venom", "toxin", "venom toxin", genes coding venom proteins. I strongly suggest the use of consistent terminologies that are well defined in the manuscript.
- Methods need elaborate descriptions about reagents, procedures including for library preparations, sequencing machines, library kits and versions, etc. These are relevant for downstream analyses.
- For all software, list parameters used, even if default, then explicitly state that "default parameters were used".
- For all software, list version numbers used for analyses.
- Authors are urged to change "macorsynteny" and "microsynteny" terms to chromosome level and local synteny analyses. This is to avoid confusion related to macro/microchromosomes.
- "Genomic diversity" analyses use cross-species alignments and variant calling using software and methods developed for same species data. This can introduce significant bias in downstream interpretation and use of the variant data (heterozygosity measure may be). I suggest removal of this section because of lack of accuracy.
- Discussion of new discovery is largely lacking. I would appreciate if authors contextualized their results with other discoveries in the field.
- Section headings in Results and Discussions can be changed to reflect main findings instead of "transcriptomics" or "genomic diversity".
- One of the main findings is about SVMP gene family expansion. However, due to the lack of evidence about assembly accuracy in the region, accurate annotation of copies, and the effect of studying "primary assembly" instead of "haplotype assembly" at this region, I am not convinced of claims made in the paper. Appropriate justification is required for this section.
- The nomenclature of SVMP genes is confusing. For example, In Figure 4A, they are all labelled as

SVMPs with different colours, but then they are labelled as MDCs and MADs in Figure 4b and Supp Figure 6. Please label each gene in each species with consistent names that can reflect orthologous relationship. This is hard to discern, especially without appropriate species labels in Supp Figure 6.
- Provide MSA files and trees used to infer evolutionary history. In the absence of the sequence alignments, and raw tree file, I am unable to evaluate this section of the manuscript. Please provide all required details for reviewers and readers.
??: It is not clear what authors mean by the word, term, phrase. Please correct them to convey accurate meaning using established and accepted scientific terminologies and English conventions.
Minor concerns:
Abstract:
- "compousing" ??
- "highly expressed toxin genes": in what tissues?
- "genome-wide diversity" ??
- "toxin gene families in venomous species" -> "toxin gene families in venomous snake species"
Background:
- "Such advances in sequencing technologies": remove "Such"
- "depending on their type, interactions, and the organism": interactions with what?
- "proteomic (and transcriptomic) approaches": remove parenthesis
- "to new therapies for human illnesses including but not": since the title contains "medically important", it would be great to include some specific examples here from the literature.
- "However, venomous snakes are one": remove "However"
- "therefore, the fundamental model system": change "fundamental" to "useful"
- "of medical importance by the World Health Organization (WHO) due to their": provide citation
- "Within venomous snakes, the most medically": restructure the sentence for brevity and clarity.
- "cytotoxic effects (among others)": remove "(among others)"
- "conducted using a proteomic approach": clarify what proteomic approach mean here.
- "Hirst et al., (in review);" : remove this citation
- "within the Viperidae family posses an available reference": change the word "posses" to something meaningful
- "Moreover, employing several -omics techniques": be specific about techniques
- "We deciphered numerous genomic attributes": be specific
Methods:
- Describe how blood was extracted from animals with all details including animal handling techniques, body part etc.
- "was stored in RNAlater until RNA extraction": source for RNAlater
- "We extracted gDNA from the blood of a female individual": provide additional details such as the quantity of blood used, thawing process, qty of reagents, especially elution buffer etc. Manufacturer protocols may be suited best for mammalian blood (humans, mice) without nucleus in RBCs unlike snakes.
- "Then, we sequenced a total of two 8M SMRT HiFi cells, aiming for a âˆ¼30x of coverage, at the University of Leiden": provide details of library preparation, sequencing machine etc.
- "(including venom glands, tongue, liver and pancreas, among others": Either list all or refer to the

table.
- "RNA libraries were prepared with the VAHTS": Was the library and sequencing strand specific? Provide complete details on these processes.
- "8M SMRT HiFi cell containing two Iso-seq HiFi libraries": use correct names of these and also include sequencing machine details.
- "Quality control on HiFi and Illumina reads was assessed using FastQC": correct the phrasing of this sentence
- "To make an initial exploration of the genome, …..we generated a k-mer profile with Meryl": Explicitly state the purpose of this analysis.
- "Manual curation was performed with Pretext": cite Pretext properly. Explain decisions of this manual curation. i.e. what evidence was used to join or break contigs.
- "Then, we ran three iterative rounds of RepeatMasker to annotate the known and unknown elements identified by RepeatModeler and soft-masked the genome for simple repeats": break this sentence into two and explain reasons for running RepeatMasker three times.
- "We used GeMoMa v.1.9": Include all details about the annotations. This sentence is not sufficient for reproducibility. Were the RNAseq data assembled or provided as raw files to GeMoMa. How were they mapped to the genome assembly f
- "published: Anolis carolinensis from AlfÃ¶ldi": Remove the word "from" here as citation is sufficient. Provide details of assembly versions, annotation version, database of annotations etc.
- "Crotalus ruber from Hirst et al., (in review)": remove this citation or list it as personal communication
- "We previously quality checked and removed the adapters of the RNA-seq data": remove "previously" and provide details on how adapters were removed from RNAseq data
- "also removed the adapters for the Iso-seq data": Explain how this was performed.
- "We blast our ..": Change all occurrence of "blast" to "BLAST" and specify parameters, if it was BLASTN or BLASTP or something else. This is not clear at all.
- "we performed additional annotation steps for venom genes.": Details are not complete for reproducibility. State explicitly what decisions were made and how gene structure was determined. This is the main part of the paper and does require accurate details.
- "Whole-genome synteny was explored between": synteny by definition refers to being on the same string/chromosome. Therefore whole-genome synteny as a term doesn't make sense given that genome is divided into chromosomes. Revise it to say "chromosomal synteny"
- "chromosomes assembled in the reverse complement, which were corrected using SAMtools faidx": samtools faidx cannot do this. Explain how this was done.
- "After adapter trimming and quality control, we mapped our RNA-seq reads": how were adapters trimmed and QC implemented.
- "Gene counts per gene": change gene counts to read counts
- "Differential expression analyses were carried out": requires additional details such as filters applied for the count, groups compared, statistical model, multiple testing correction methods.
- "characterize the venom arsenal of Cerastes gasperettii": change the arsenal word.
- "Fragmentation spectra were matched against a customized database including the bony vertebrates taxonomy dataset of the NCBI non-redundant database": revise for accuracy
- "Unmatched MS/MS spectra were de novo sequenced": spectra were sequenced how??

- "we used blast, incorporating both toxin and non-toxin paralogs": change blast to BLAST and provide additional details about the tool used
- "Then, we aligned those regions using Mafft (Katoh": provide coordinates of these regions for future research in each assembly
- "history for the main groups of toxins (i.e.,": parenthesis is not closed. Close it or remove it.
- "we also included other non-toxin paralogous genes from nontoxic species (for details about this see Supplementary Information": where do I look into the supplementary information? Be very clear. Provide coordinates of regions that were compared.
- "When needed, we translated CDS": when was this needed? Explain.
- "built a phylogeny for each of the toxin groups using Phyml": I presume that this is done with translated CDS sequences in toxin genomic regions. Please clarify.
- "Heterozygous positions were obtained from bam files with Samtools v1.9": provide details as to how this was done. Samtools doesn't have features to operate at a site level and therefore I am confused.
- "Filtered reads were mapped against the new reference genome of Cerastes gasperettii using the bwa mem algorithm": bwa mem is designed for same species comparisons. Here you have used it for cross-species. Provide justification and perhaps biases it may have introduced for distantly related species.
- "SNP calling was carried out …": This is not appropriate as models assume same species data. You have used cross-species alignments, which can be highly biased.

Results and Discussion:

- "PacBio HiFi (~40x), Hi-C (~60x) and Illumina data (~78x)": change to number of base pairs. 40x for a genome of 2GB is 80GB data and for genome of 1GB size, it is 40GB data. Before sequencing and assembly, the genome size cannot be known.
- "After manual curation, we enhanced the scaffolding parameters of our genome": what was done as manual curation. Please specify.
- "âˆ¼228 times more contiguous than the Anolis sagrei genome": how is 228 more measured. How is this useful as a metric without the known ground truth. Assemblies can and do have errors.
- "27,158 different protein-coding genes within our assembly": this seems large compared to other species. Can you elaborate or compare these numbers with other species.
- "Toxin genes usually found in venomous snakes (see proteome results below) were mainly found on macrochromosomes, although major toxin groups
- were found on microchromosomes (SVMPs, SVSPs and PLA2; Fig. 1)." : please revise this statement. Two part of the sentence are saying opposite things. Second provide coordinates of these genes as GFF/BED file as supplementary file with their exon structure annotations for others to reuse this information.
- "showed a great level of similarity between Cerastes gasperettii and Crotalus adamanteus": provide quantitative metrics for "great" level of similarity.
- "we found several fission events in the A. sagrei genome,": Since A. sagrei genome is not contiguous and chromosome scale, you cannot infer fissions as it may be artefact of non-contiguous assembly. If that is not the case, provide evidence of this.
- "The last four…": Belongs in methods
- "Macrosyntenic differences between lizards and snakes": this is very superficial discussion point. Please remove it or strengthen it with evidence.

- "Heatmap analyses with the most 2,000": Revise this statement. It doesn't make sense. E.g. Heatmap is a visualisation technique and not analyses method.
- "We studied venom evolution within the most abundant toxin groups": rewrite the sentence for clarity and brevity.
- "After a thorough manual curation": Explain what was this manual curation process clearly and the purpose of it.
- "contiguous tandem repeat SVMPs for": Change "repeat" to "array" because tandem repeat has a different meaning in genomics research context.
- "flanked by the NEFL and NEFM": Unclear if they are both 5' or 3' of toxin genes. Clarify
- "Microsyntenic analyses showed": change to local synteny
- "gene copy number variation between": Since these are duplicate copies, clearly state how gene copies were identified. Include details of open reading frames, exon structures, pseudogene status, etc
- "we can see an expansion in": Describe number of new copies, their status as intact or not, and sequence similarity between copies. Provide evidence that there is no false duplication due to heterozygous allele collapse in the assembly.
- "More genomic data will indicate if SVMP12": Did you mean SVMP13?
- "This difference may be expected, as PLA2 only represents around 5% of the proteome for Cerastes gasperettii": This is not true. Proteome doesn't equal to genome in some cases and superficial inference such as this is not warranted.
- For PSMC analyses, please discuss the effect of mutation rate and generation time.

Figures:
- Figure 1: Add y-axis scales to the circos plot.
- Figure 1b legend says it is a linkage map, but looks more like HiC contact map. Please edit.
- Figure 1b legend also says "including the sex chromosomes", which is not consistent with the circos plot.
- Figure 3A refers to transcriptome and 3b to proteome. Please make this very clear.
- Figure 4A, C and E, label genes consistent with the phylogenetic trees in supplementary figures so readers can know their genomic arrangements.
- Figure S4: Discuss why CG1 sample separates from rest of the samples. Seems like a batch effect.

Methods

Are the methods appropriate to the aims of the study, are they well described, and are necessary controls included? Choose an item.

Conclusions

Are the conclusions adequately supported by the data shown? Choose an item.

Reporting Standards

Does the manuscript adhere to the journal's guidelines on minimum standards of reporting? Choose an item.

Choose an item.

Statistics

Are you able to assess all statistics in the manuscript, including the appropriateness of statistical tests used? Choose an item.

Quality of Written English

Please indicate the quality of language in the manuscript: Choose an item.

Declaration of Competing Interests

Please complete a declaration of competing interests, considering the following questions:

- Have you in the past five years received reimbursements, fees, funding, or salary from an organisation that may in any way gain or lose financially from the publication of this manuscript, either now or in the future?
- Do you hold any stocks or shares in an organisation that may in any way gain or lose financially from the publication of this manuscript, either now or in the future?
- Do you hold or are you currently applying for any patents relating to the content of the manuscript?
- Have you received reimbursements, fees, funding, or salary from an organization that holds or has applied for patents relating to the content of the manuscript?
- Do you have any other financial competing interests?
- Do you have any non-financial competing interests in relation to this paper?

If you can answer no to all of the above, write 'I declare that I have no competing interests' below. If your reply is yes to any, please give details below.

I declare that I have no competing interests

I agree to the open peer review policy of the journal. I understand that my name will be included on my report to the authors and, if the manuscript is accepted for publication, my named report including any attachments I upload will be posted on the website along with the authors' responses. I agree for my report to be made available under an Open Access Creative Commons CC-BY license (http://creativecommons.org/licenses/by/4.0/). I understand that any comments which I do not wish to be included in my named report can be included as confidential comments to the editors, which will not be published.

Choose an item.

To further support our reviewers, we have joined with Publons, where you can gain additional credit to further highlight your hard work (see: https://publons.com/journal/530/gigascience). On publication of this paper, your review will be automatically added to Publons, you can then choose whether or not to claim your Publons credit. I understand this statement.

Yes Choose an item.
